# Dearomative triple elementalization of quinolines driven by visible light

**DOI:** 10.1038/s41467-023-36161-4

**Published:** 2023-02-06

**Authors:** Shiho Ishigaki, Yuki Nagashima, Daiki Yukimori, Jin Tanaka, Takashi Matsumoto, Kazunori Miyamoto, Masanobu Uchiyama, Ken Tanaka

**Affiliations:** 1 Department of Chemical Science and Engineering, Tokyo Institute of Technology, O-okayama, Meguro-ku, Tokyo, 152-8550 Japan; 2 Graduate School of Pharmaceutical Sciences, The University of Tokyo, 7-3-1 Hongo, Bunkyo-ku, Tokyo, 113-0033 Japan; 3 Rigaku Corporation, 3-9-12 Matsubara-cho, Akishima, Tokyo, 196-8666 Japan; 4 Research Initiative for Supra-Materials (RISM), Shinshu University, 3-15-1 Tokida, Ueda, Nagano, 386-8567 Japan

**Keywords:** Synthetic chemistry methodology, Reaction mechanisms, Synthetic chemistry methodology, Stereochemistry, Photochemistry

## Abstract

Organoboron and organosilicon compounds are used not only as synthetic building blocks but also as functional materials and pharmaceuticals, and compounds with multiple boryl and silyl groups are beginning to be used for these purposes. Especially in drug discovery, methodology providing easy stereoselective access to aliphatic nitrogen heterocycles bearing multiple boryl or silyl groups from readily available aromatic nitrogen heterocycles would be attractive. However, such transformations remain challenging, and available reactions have been mostly limited to dearomative hydroboration or hydrosilylation reactions. Here, we report the dearomative triple elementalization (carbo-sila-boration) of quinolines via the addition of organolithium followed by photo-boosted silaboration, affording the desired products with complete chemo-, regio-, and stereoselectivity. The reaction proceeds via the formation of silyl radicals instead of silyl anions. We also present preliminary studies to illustrate the potential of silaboration products as synthetic platforms.

## Abstract (web summary)

Organoboron and organosilicon compounds have potential applications in various fields including materials science and medicinal chemistry. Here the authors report a protocol that enables the dearomative carbo-sila-boration of quinolines.

## Introduction

**Fig. 1:**
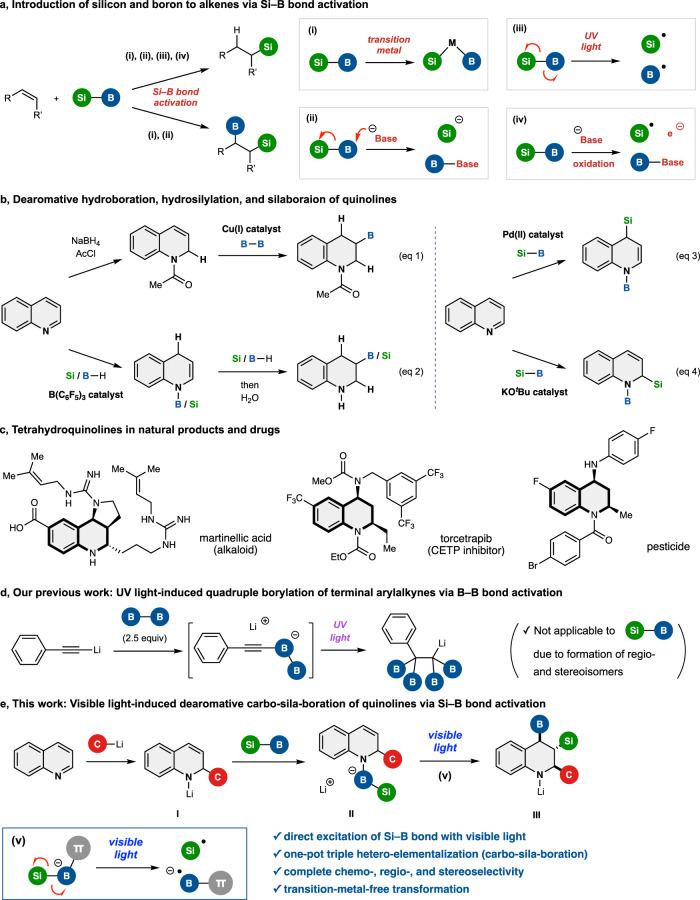
Overview of previous studies and this work. **a** Hydrosilylation and silaboration of alkenes using silylborane (Si–B) via four types of Si–B bond activation. **b** Previous work on dearomative hydroboration, hydrosilylation, or C–Si and N–B bond-forming silaboration of quinolines. **c** 1,2,3,4-Tetrahydroquinoline natural products and drugs. **d** Our previous work: quadruple borylation. **e** This work: dearomative triple elementalization (carbo-sila-boration).

Organoboron and organosilicon compounds are attracting attention as optoelectronic materials^1–4^ and pharmaceuticals^5–8^, in addition to their utility as building blocks in organic synthesis^9–12^, and the discovery of cross-coupling reactions for constructing C(*sp*^2^)–B and C(*sp*^2^)–Si bonds have advanced the use of aryl boron and silicon compounds in these fields^13,14^. In contrast, although the synthesis of aliphatic boron and silicon compounds is relatively easy with classical methods^15,16^, their (stereospecific) transformation remains challenging. Nevertheless, C(*sp*^3^)–B and C(*sp*^3^)–Si bond conversions have begun to be developed, and the products have potential applications in materials science and medicinal chemistry^11,12^. Furthermore, aliphatic compounds bearing both silyl and boryl groups with different reactivity and properties have potential value as synthetic platforms. Silaboration of alkenes with silylboranes (Si–B) is one of the most straightforward methods for their synthesis^17–21^. Si–B bond activation is necessary to realize this reaction, though the covalent hetero-interelement Si–B bond is stable and requires high energy to cleave. So far, four Si–B bond activation methods have been developed (Fig. 1a): (i) transition metal-catalyzed activation^22–24^, (ii) base-mediated activation^25–28^, (iii) photo-excitation by ultraviolet (UV) light^29^, and (iv) oxidative activation by a photoredox catalyst^30,31^. Although the methods involving silyl radical species (iii and iv) are only applicable to hydrosilylation of alkenes, the methods utilizing silyl metal species (i) and silyl anion species (ii) are applicable not only to hydrosilylation but also to silaboration of isolated alkenes.

If dearomative C–Si and C–B bond-forming silaboration could be applied to aromatic nitrogen heterocycles instead of simple alkenes, the resulting aliphatic nitrogen heterocycles would be of great interest in the field of medicinal chemistry^32–36^. However, such transformations are challenging, and available methods have been limited to dearomative hydroboration, hydrosilylation, or C–Si and N–B bond-forming silaboration reactions of aromatic nitrogen heterocycles^37–42^. For example, the construction of the 1,2,3,4-tetrahydroquinoline skeleton, which is a common structure in natural products and pharmaceuticals (Fig. 1c)^43–45^, by reductive hydroboration (Fig. 1b, eqs 1 and 2)^40,41^, hydrosilylation (Fig. 1b, eq 2)^38^, or C–Si and N–B bond-forming silaboration (Fig. 1b, eqs 3 and 4)^37,42^ of quinolines has been reported (Fig. 1b), but the simultaneous introduction of boron and silicon on carbon atoms in this skeleton has not been achieved.

Recently, we developed a UV light-induced B–B bond^46^ (homo-interelement bond) activation method, achieving quadruple borylation of terminal aryl acetylides (Fig. 1d)^47^. In this reaction, the B–B bond was excited and smoothly cleaved under UV irradiation by the ate complexation^48,49^ of diboron (B–B) with light-absorbing aryl acetylene. However, due to the use of highly energetic UV light, the regioselectivity and stereoselectivity cannot be controlled in reactions involving hetero-interelement bonds such as Si–B bonds.

Here, we report the visible-light-induced one-pot dearomative triple elementalization (carbo-sila-boration) of quinolines via (1) the addition of organolithium to quinoline to form lithium anilide **I**^50–53^, (2) ate complexation of **I** with silylborane **II**, and (3) direct Si–B bond activation by visible light irradiation to afford the product **III** (Fig. 1e). This method allows the catalyst-free conversion of readily available quinoline derivatives to 2-alkyl-3-silyl-4-boryl-1,2,3,4-tetrahydroquinoline scaffolds in chemo-, regio-, and stereoselective manner. Experimental and computational studies reveal that this selective reaction proceeds via a Si–B bond activation process, in which visible light excitation of anilide–silylborane ate complex **II** produces a silyl radical rather than a silyl anion. We also present preliminary studies on the chemo- and stereospecific conversions of C–B/C–Si bonds to C–C, C–O, and C–H/D bonds that serve to illustrate the potential of the silaboration products as synthetic platforms.

## Results and discussion

### Optimization of reaction conditions

**Fig. 2:**
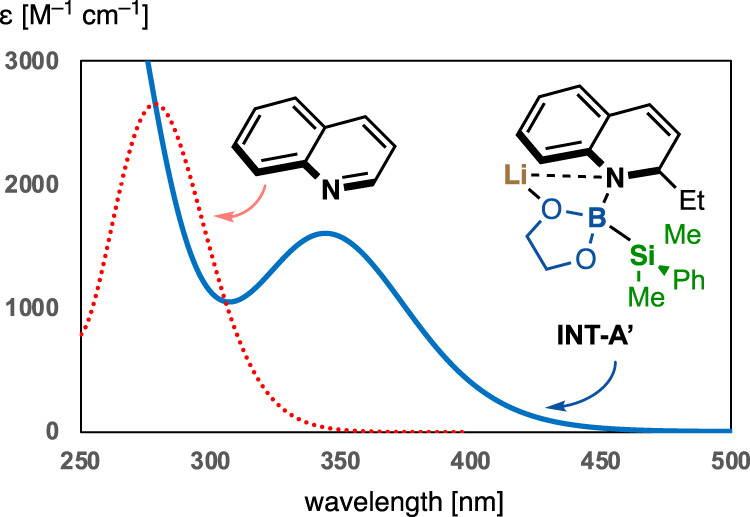
UV/vis absorption spectrum of INT-A’ by TDDFT calculations. TDDFT Calculations were performed at B3LYP/6-31+G* levels of theory.

We commenced our study of the dearomative carbo-sila-boration (C/Si/B) by using 6-methoxyquinoline (**1a**) with a combination of PhMe_2_Si–B(pin) **2a** as a silylborane and ^n^BuLi as an organometal reagent (Table 1). In contrast to our previous reports involving the quadruple borylation of terminal acetylenes^47^, thermal and Hg lamp irradiation (>250 nm, ultraviolet light) conditions, which would activate the Si–B bond of borate intermediate **INT-A** generated from **1a**, **2a**, and ^*n*^BuLi, resulted in either no reaction or complex mixtures (entries 1–3). These results may be attributed to undesired activation (excitation). To overcome this problem, we envisioned that selective excitation of **INT-A** by irradiation at an optimal wavelength would facilitate selective Si–B bond cleavage under mild conditions. Our time-dependent density functional theory (TDDFT) calculations of quinoline and model borate complex **INT-A’** predicted that the longest absorption band of **INT-A’** (366 nm), assigned to the HOMO–LUMO transition, shows a red shift from that of quinoline (289 nm) (Fig. 2). Thus, visible-light irradiation would selectively afford the excited state of ate complex INT-A’* to realize the desired triple elementalization of the quinoline. Pleasingly, the use of a white LED provided the desired carbo-sila-borated (C/Si/B) product (**3aa**) with 65% yield (entry 4). The structure and stereochemistry of **3aa** were unambiguously determined by single-crystal X-ray crystallographic analysis. Notably, the two stereocenters on boron and silane were formed with complete regio- and diastereoselectivity (>99:1), as confirmed by ^1^H-NMR analysis of the crude product.

**Table 1:** Optimization of reaction conditions


Entry	Conditions for Si–B bond activation	Solvent	Yield 3aa (%)
1	Dark, 50 °C	Dioxane	0
2	Dark, 100 °C	Dioxane	n.d.^a^
3	Hg lamp (> 250 nm), r.t.	Dioxane	n.d.^a^
4	White LEDs, r.t.	Dioxane	65
5	Blue LEDs, r.t.	Dioxane	80
6	Dark, r.t.	Dioxane	9
7	Blue LEDs^b^, r.t.	THF	46
8	Blue LEDs^b^, r.t.	Toluene	60
9	Blue LEDs^b^, r.t.	Hexane	39
10^c^	Blue LEDs^b^, r.t.	Dioxane	88

Unless otherwise stated, reactions were performed on a 0.25 mmol (**1a**) scale under argon. *N*–Acetylation using AcCl was performed at 50 °C until the reaction was completed. Yields were determined by ^1^H-NMR analysis.

*pin* pinacolate, *Ac* acetyl.

^a^Not determined, a complicated mixture of products was obtained.

^b^Blue LEDs (wavelength range: 390–480 nm) was used.

^c^3 equiv of **2a** was employed.

We surveyed various types of light sources to facilitate the Si–B bond cleavage process and found that the yield of **3aa** was drastically improved to 80% when blue LEDs were used (entry 5). Importantly, the yield significantly decreased under dark conditions (entry 6). Examination of several solvents revealed dioxane to be much superior to THF, hexane, or toluene (entries 7–9). Increasing the amount of silylborane **2a** (3 equiv) promoted the reaction to afford the desired product in the highest yield of 88% (entry 10). These conditions were found to be optimal.

### Dearomative triple elementalization of quinolines

**Fig. 3:**
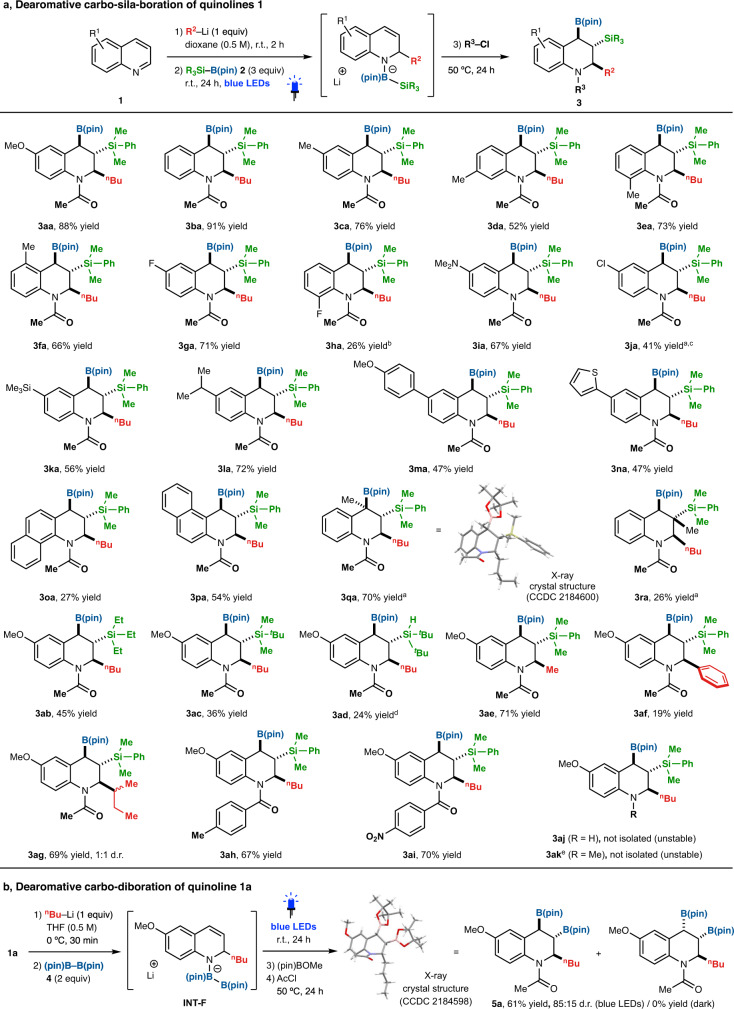
Dearomative triple elementalization of quinolines. **a** Synthesis of carbo-sila-borated tetrahydroquinolines. **b** Synthesis of carbo-diborated tetrahydroquinoline. Reactions were performed on a 0.25 mmol (**1**) scale under argon. *N*–Functionalization using R^3^Cl was performed at 50–80 °C until the reaction was completed. The yields were determined by ^1^H-NMR analysis. ^a^Run for 48 h. ^b^Run for 96 h. ^c^THF was used instead of dioxane as a solvent. ^d^370 nm LEDs were used instead of blue LEDs as a light source. ^e^Iodomethane was used instead of acyl chloride at 80 °C.

With the optimized conditions in hand, we next investigated the substrate scope of this triple elementalization (Fig. 3a). A variety of quinolines could be employed: (1) the position (6-, 7-, 8-, or 9-) of the methyl group on the benzene part had little impact on the reaction (**3ca**, **3da**, **3ea**, and **3fa**); (2) quinolines with electron-donating substituents (OMe, alkyl, amino, and silyl groups; **3aa**, **3ca**, **3ia**, **3ka**, and **3la**) as well as electron-withdrawing substituents (fluoro, chloro, phenyl, and thienyl groups; **3ga**, **3ja**, **3ma**, and **3na**) at the 6-position of the benzene part were efficiently converted to the corresponding carbo-sila-borated products in moderate to excellent yields; (3) π-extended aromatics **1o** and **1p** were also available; (4) 4- and 3-substituents (**1q** and **1r**) on the pyridine part were tolerated, affording 1,2,3,4-tetrahydroquinolines with tetrasubstituted stereocenters, though 2-methylquinoline was unreactive. The stereochemistry of the tertiary boronic ester **3qa** was unambiguously determined by single-crystal X-ray crystallographic analysis. For the silyl substituents, not only the dialkylarylsilyl group but also trialkylsilyl (**3ab** and **3ac**) and hydrosilyl (**3ad**) groups were compatible. In addition, various alkylations, including *n*-butylation (**3aa**), methylation (**3ae**), phenylation (**3af**), and *sec*-butylation (**3ag**) could be employed in the first dearomative alkylation step to afford the corresponding products. The starting quinolines **1** were not recovered in the reactions shown in Fig. 3a. Some *N*-functionalizations of 1,2,3,4-tetrahydroquinolines, such as the acetyl (**3aa**) and the *p*-methyl or *p*-nitrobenzoyl (**3ah** and **3ai**) substituted compounds, were available in one pot without further purification. However, the non-substituted product (**3aj**) obtained without acyl chloride and the *N*-methylated product (**3ak**) synthesized using iodomethane instead of acyl chloride were only detected as major products by ^1^H-NMR and ESI-MS of the crude mixtures and could not be isolated due to their instability. Importantly, all the products listed in Fig. 3a were obtained with complete regio- and diastereoselectivity.

We also investigated a dearomative carbo-diboration reaction (Fig. 3b). The use of bis(pinacolate)diboron (B–B) **4** instead of silylborane **2** gave the diborate intermediate **INT-F**. Blue LED irradiation of **INT-F** led to a carbo-diboration reaction, giving carbo-diboration product **5a** with good diastereoselectivity (85:15) as confirmed by ^1^H NMR analysis of the crude reaction mixture (Fig. 3b). The stereochemistry of the major diastereomer of **5a** was confirmed by X-ray crystallography. These reactions were completely shut down in the dark, indicating that the present selective photoexcitation strategy is also essential for the B–B bond activation.

### Silaborated tetrahydroquinolines as synthetic platforms

**Fig. 4:**
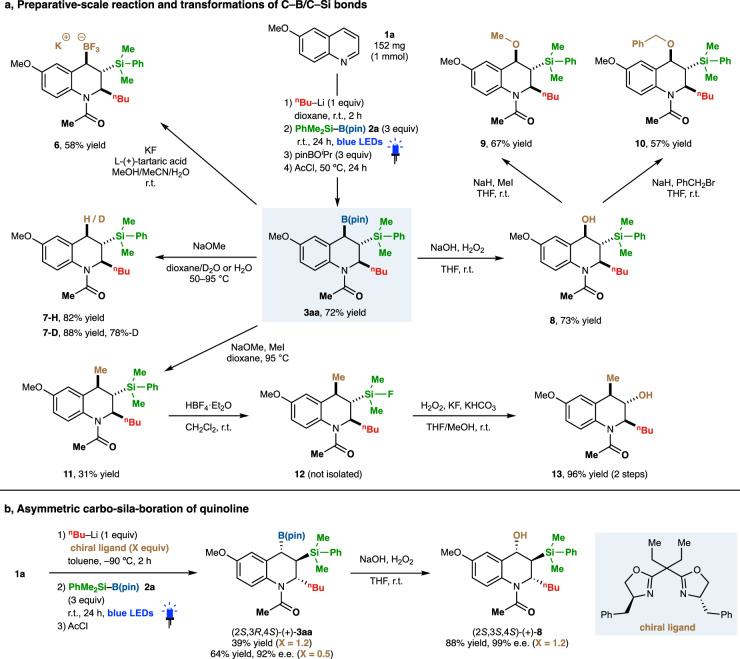
Silaborated tetrahydroquinolines as synthetic platforms and an asymmetric synthesis. **a** Use of silaborated tetrahydroquinolines as synthetic platforms. **b** Asymmetric synthesis of 3-silyl-4-boryl-1,2,3,4-tetrahydroquinoline. Yields were determined by ^1^H-NMR analysis.

We have also obtained a variety of highly functionalized 1,2,3,4-tetrahydroquinoline derivatives by preparative-scale synthesis and chemo- and stereospecific transformations of 2-alkyl-3-silyl-4-boryl-1,2,3,4-tetrahydroquinoline **3aa** (Fig. 4a). The dearomative triple elementalization of 6-methoxyquinoline (**1a**, 152 mg, 1 mmol) using an additional electrophile [(pin)BO^*i*^Pr] smoothly proceeded to give the desired 1,2,3,4-tetrahydroquinoline **3aa** in 72% yield. The pinacol group on the boryl moiety of **3aa** could be easily removed by KF to give trifluoroborate salt **6** in good yield^54^. For selective transformation of the boryl group, hydrodeboration or deuterodeboration proceeded under basic conditions using NaOMe to afford the corresponding products **7-H** (C/Si/H) and **7-D** (C/Si/D). The direct oxidation of **3aa** afforded alcohol **8** (C/Si/O), which was further converted to methyl ether **9** and benzyl ether **10**. Alkylation using MeI and NaOMe provided methylation product **11** (C/Si/C) with retention of the stereochemistry. Transformation of the silyl group was also achieved: the highly sterically hindered C–Si bond of **11** was smoothly oxidized to a C–O bond via fluorosilane intermediate **12** under the standard conditions of Tamao–Fleming oxidation to afford the corresponding alcohol **13** (C/O/C). Furthermore, the present photo-boosted dearomative triple elementalization and subsequent derivatization could be combined with the asymmetric alkylation of quinolines reported by the Alexakis group^55^, affording enantioenriched 2-alkyl-3-silyl-4-boryl-1,2,3,4-tetrahydroquinoline (2*S*,3*R*,4*S*)-(+)-**3aa** and 1,2,3,4-tetrahydroquinoline-4-ol (2*S*,3*S*,4*S*)-(+)-**8** with excellent enantioselectivity (up to 99% ee, Fig. 4b). These preparative-scale reactions and product transformations suggest that silaborated 1,2,3,4-tetrahydroquinoline derivatives have considerable potential as synthetic platforms.

### Experimental and theoretical mechanistic studies

**Fig. 5:**
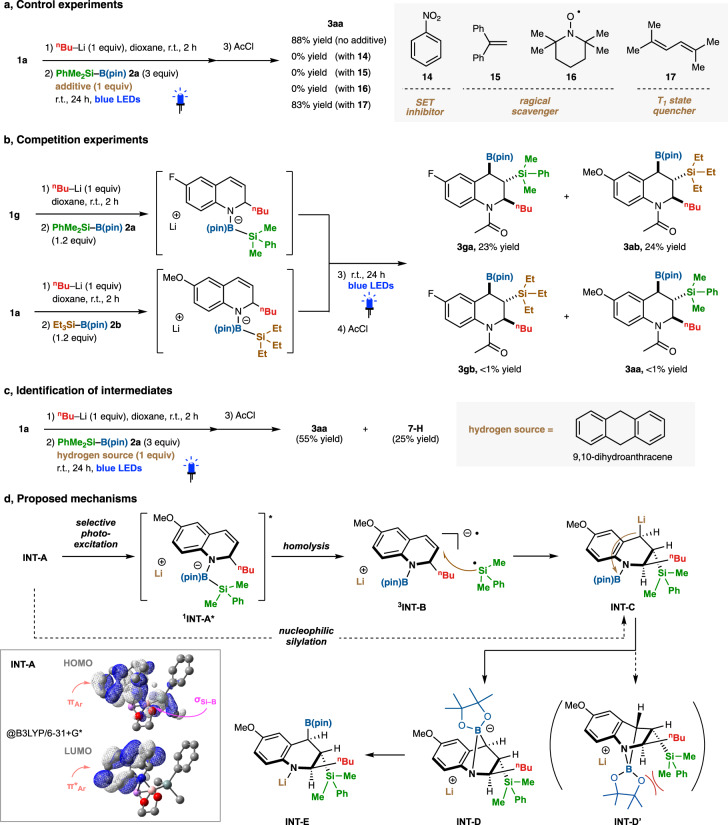
Experimental mechanistic studies. **a** Control experiments. **b** Competition experiments. **c** Identification of intermediates. **d** Proposed mechanisms. Yields were determined by ^1^H-NMR analysis.

Next, we sought to acquire mechanistic insights into the triple elementalization reaction. Firstly, control experiments were performed as shown in Fig. 5a. The reaction was inhibited by a single-electron-transfer (SET) inhibitor [nitrobenzene (**14**)] and by radical scavengers [1,2-diphenylethylene (**15**) and (2,2,6,6-tetramethylpiperidin-1-yl)oxy (TEMPO, **16**)], but not by an excited triplet-state quencher **17**. These results indicate that this reaction involves both excited-state species and free radical species. We also identified the adducts of the in-situ-generated silyl radical^56^ with TEMPO **16**^57^ and with aliphatic alkene (*tert*-butyl 4-methylenepiperidine-1-carboxylate, **S1**) by means of GCMS or ESI-MS measurements, as shown in Supplementary Figs. 5, 6. Competition experiments using two types of combinations of silylboranes (**2a** and **2b**) and quinolines (**1a** and **1g**) resulted in no detection of the cross-reaction products (**3gb** and **3aa**), which suggests that the borate intermediate undergoes intramolecular reaction (Fig. 5b). In addition, as depicted in Fig. 5c, the addition of 9,10-dihydroanthracene as a hydrogen source before the light irradiation afforded not only the desired product (**3aa**) but also 2-alkyl-3-silyl-1,2,3,4-tetrahydroquinoline **7-H** as the hydrogenated intermediate in 25% yield. This result indicates that the present triple elementalization consists of the following three events; (1) dearomative alkylation, (2) photo-induced intramolecular silylmetalation of 1,2-dihydroquinoline, and (3) intramolecular borylation of the benzyl anion species.

Based on the experimental studies, a plausible mechanism is illustrated in Fig. 5d. As already mentioned, nucleophilic silylation from the borate intermediate **INT-A** did not proceed effectively. Instead, **INT-A** could be selectively excited by photo-irradiation to a singlet excited state (S_1_; ^**1**^INT-A*), which enables cleavage (homolysis) of the Si–B bond to proceed smoothly, generating both silyl radical and radical anion species ^**3**^**INT-B**. Indeed, TD-DFT calculation of **INT-A** indicates that the charge-transfer character from the HOMO composed of the σ orbital of the Si–B bond and the π orbital of the 1,2-dihydroquinoline moiety to the π* orbital of the 1,2-dihydroquinoline moiety (LUMO) is predominant in the visible absorption range. Then, radical coupling yields the corresponding silylmetalated intermediate **INT-C**. Finally, the boryl group transfer from the nitrogen atom to the carbon atom via bicyclo intermediate **INT-D** affords the desired triple elementalization (C/Si/B) product **INT-E**. The diastereoselectivity of the boryl and silyl groups would be determined by steric hindrance in **INT-D’**, resulting in the anti-configuration of these groups.

**Fig. 6:**
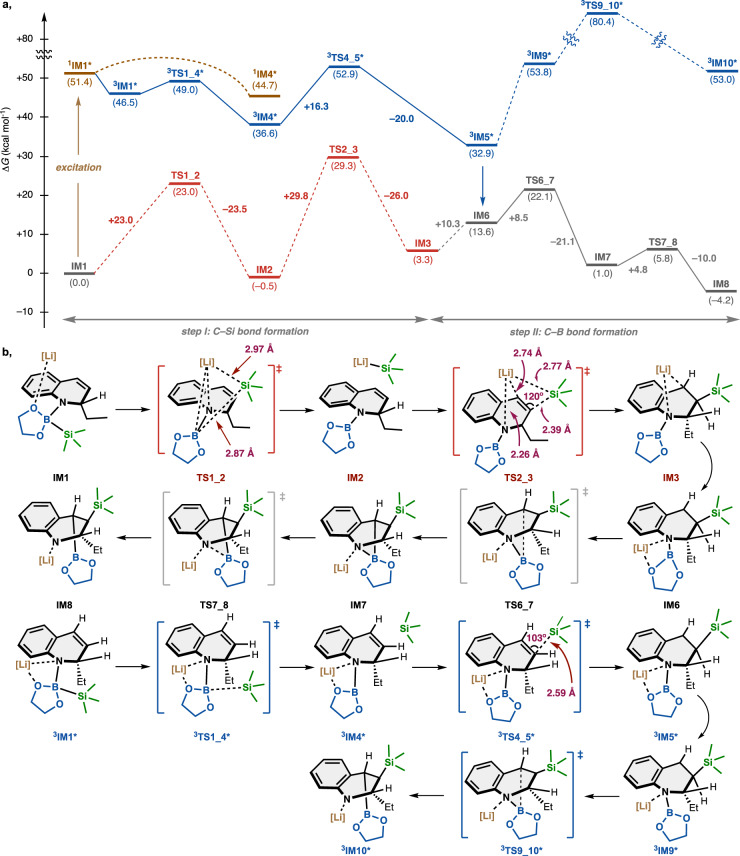
Theoretical mechanistic studies. **a** Energy diagram using Gibbs free energies. **b** Chemical structures of intermediates and transition states. Gibbs free energy changes are shown in kcal mol^–1^. DFT Calculations were performed at the (U)B3LYP/6-31+G*&PCM(1,4-dioxane)//(U)B3LYP/6-31+G* level of theory. [Li] = Li(OMe_2_)_2_. IM intermediate, TS transition state.

To gain detailed insight into the proposed mechanisms, model calculations using borate complex **IM1**, which is generated from **1b**, **2a**, and ethyllithium (EtLi) coordinated with two dimethyl ethers (model solvent), were performed as shown in Fig. 6. Borate complex **IM1** facilitates cleavage of the Si–B bond to generate silyllithium (silyl anion) species **IM2**, which is stabilized by the π orbital of the styrene unit and the lone pair of the nitrogen atom. Then, the addition of this silyllithium species to the styrene moiety affords the corresponding silylmetalated intermediate **IM3**. However, these events both suffer from high activation energies (+23.0 and +29.8 kcal mol^–1^, respectively), mainly due to the stability of the Si–B bond (**TS1_2**) and the steric bulkiness of the quinoline skeleton and the silyllithium species (**TS1_2** and **TS2_3**). In contrast, our TDDFT calculation of **IM1** in the excited S_1_ state indicates that a large energy gain would facilitate smooth cleavage of the Si–B bond to form ^**3**^IM4* via ^**3**^IM1* with a small activation energy of 2.5 kcal mol^–1^. The resulting silyl atom shows the nature of a “silyl radical.” The subsequent silyl radical addition to the styrene moiety (^**3**^IM4* → ^**3**^IM5*) requires a smaller activation energy (16.3 kcal mol^−1^) than the corresponding silyl anion addition (**IM2** → **IM3**), resulting in the silylmetalated intermediate ^**3**^IM5*. The structure of ^**3**^TS4_5* clearly shows that the steric interaction between the quinoline skeleton and the silyl substituent is smaller than that of **TS2_3**. These steps complete the C(*sp*^3^)–Si bond formation, leading to silylmetalated intermediate **IM6**, which is in equilibrium with the intermediate **IM3**.

In addition, a reaction pathway for C(*sp*^3^)–B bond formation was also identified by DFT calculations. The transfer of the boryl group from the nitrogen atom to the carbon atom (**IM6** → **IM8**) involves two transition states (**TS6_7** and **TS7_8**) for C(*sp*^3^)–B bond formation and N–B bond cleavage, respectively, both of which are kinetically and thermodynamically favorable (∆*G*^‡^ < 20 kcal/mol and ∆*G* < 0 kcal/mol) in the ground state. On the other hand, the C(*sp*^3^)–B bond formation in the triplet state (^**3**^IM5* → ^**3**^IM9* → ^**3**^IM10*) is kinetically and thermodynamically unfavored. These calculations are in good agreement with the experimental results, and thus we can conclude that in situ generations of radical species from the excited borate complex facilitates the present hetero-elementalization reaction.

### Dearomative triple elementalization of anthracene and phenanthrene

**Fig. 7:**
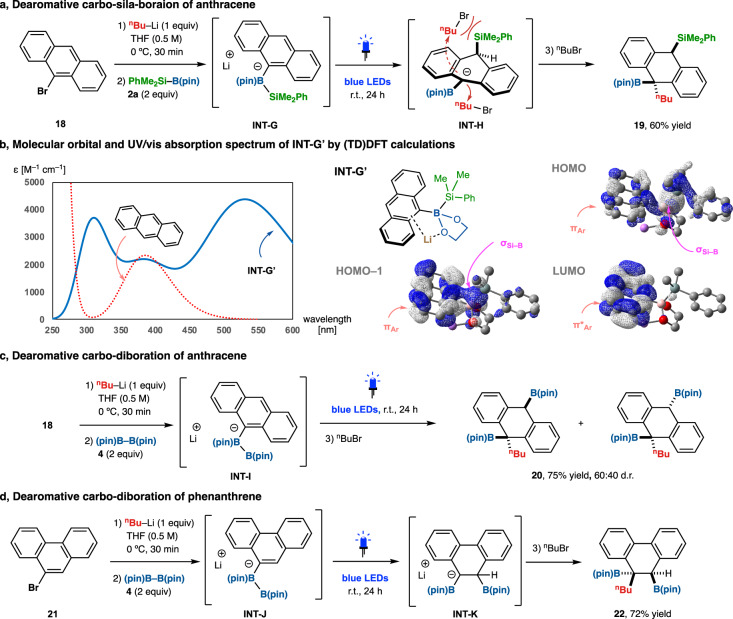
Dearomative triple elementalization of anthracene and phenanthrene. **a** Synthesis of carbo-sila-borated dihydroanthracene. **b** Molecular orbital and UV/vis absorption spectrum of **INT-G’** by (TD)DFT calculations at the B3LYP/6-31+G* level of theory. **c** Synthesis of carbo-diborated dihydroanthracene. **d** Synthesis of carbo-sila-borated dihydrophenanthrene. Reactions were performed on a 0.25 mmol (**18** or **21**) scale under argon. Yields were determined by ^1^H-NMR analysis.

Finally, based on the mechanisms proposed in Fig. 5d, we investigated another type of dearomative triple elementalization. The use of 9-bromoanthracene (**18**) instead of quinoline (**1**) yielded borate intermediate **INT-G** via a halogen–lithium exchange reaction using ^*n*^BuLi (Fig. 7a). As in the case of quinoline, TDDFT calculations for anthracene and the model borate complex **INT-G’** predicted that the longest and the second longest absorption bands of **INT-G’** (532 and 396 nm) are due to the HOMO–LUMO and HOMO-1–LUMO transitions, respectively, showing redshifts from anthracene (385 nm) (Fig. 7b). Both the transitions show charge-transfer character from the HOMO or the HOMO-1, consisting of the σ orbital of the Si–B bond and the π orbital of the anthracene moiety, to the π* orbital (LUMO) of the anthracene moiety. Gratifyingly, irradiation of **INT-G** with blue LEDs followed by alkylation of **INT-H** with *n*-butyl bromide led to the carbo-sila-boration reaction, affording 9-silyl-10-alkyl-boryl-9,10-dihydroanthracene **19** in 60% yield with complete regio- and stereoselectivity. The diastereoselectivity is expected to be determined by the electrophilic addition of *n*-butyl bromide at the sterically unhindered side, resulting in the anti-configuration of the *n*-butyl and silyl groups. Using bis(pinacolato)diboron (**4**, B–B) instead of silylborane **2a**, carbo-diboration of anthracene via borate complex **INT-I** afforded 9-boryl-10-alkyl-boryl-9,10-dihydroanthracene **20** in 75% yield and a diastereomeric ratio of 60:40 as confirmed by ^1^H NMR analysis of the crude product (Fig. 7c). Dearomative carbo-diboration of 9-bromophenanthrene (**21**) instead of **18** also proceeded to give 9-boryl-10-alkyl-boryl-9,10-dihydrophenanthrene **22** in 72% yield via borate complex **INT-J** (Fig. 7d). However, dearomative carbo-diboration and carbo-sila-boration of 1-bromonaphthalene did not proceed, probably due to the high aromaticity of the naphthalene ring. Thus, the present Si–B/B–B bond photoactivation strategy may be useful for the functionalization of various polycyclic aromatic hydrocarbons.

In conclusion, we have accomplished chemo-, regio-, and stereoselective dearomative triple elementalization (carbo-sila-boration) of quinolines by the addition of organolithium followed by photo-boosted silaboration. Experimental and computational studies indicate that the selective photoexcitation of borate complexes results in smooth cleavage of the Si–B bond to form silyl radicals rather than silyl anions, enabling carbo-sila-boration (C/Si/B) to occur. Although chemo- and stereoselective transformations of adjacent boron–silicon functional groups are still under study, carbo-sila-borated tetrahydroquinolines are expected to be versatile synthetic platforms; for example, the conversion of boryl and silyl groups yields carbo-oxy-silylation (C/Si/O), carbo-sila-deuteration (C/Si/D), dicarbo-silylation (C/Si/C), and dicarbo-oxidation (C/O/C) products in a regio- and stereoselective manner.

## Methods

### General procedure for dearomative carbo-sila-boration of quinolones

Quinoline **1** (0.25 mmol) was charged in a dried Schlenk tube and dissolved in dry dioxane (0.50 mL). To the mixture was added organolithium (0.25 mmol; 2.55 M in hexane solution) at 0 °C. The solution was stirred for 2 h, and then silylborane **2** (0.75 mmol) was added at room temperature. The reaction tube was sealed and irradiated with blue LEDs equipped with a cooling fan at room temperature for 24 hours. *N*-Acylation reagent (1.0–1.5 mmol) was then added to the mixture at room temperature and the resulting mixture was stirred at 50 °C for 24 h. The reaction was quenched with water (2 mL) and the mixture was extracted with ethyl acetate (10 mL × 3). The ethyl acetate layers were combined and dried over MgSO_4_, and the solvent was removed under reduced pressure. The residue was purified by preparative thin-layer chromatography.

## Supplementary information

Supplementary Information

Description of Additional Supplementary Files

Supplementary Data 1

## Data Availability

The crystallographic data generated in this study have been deposited in the Cambridge Crystallographic Data Centre under accession code CCDC 2184597 (**3aa**), CCDC 2184600 (**3qa**), and CCDC 2184598 (**5a**). Cartesian coordinates of intermediates and transition states are available in Supplementary Data 1. All of the other data supporting the findings of this study are provided in the main text or the Supplementary Information.
