# Supplementary material for: Dearomative triple elementalization of quinolines driven by visible light
Source: Nat Commun. 2023 Feb 6;14:652. doi: 10.1038/s41467-023-36161-4 (PMC9902486; doi:10.1038/s41467-023-36161-4)
Supplement: Supplementary file 1 — Supplementary Information [file 41467_2023_36161_MOESM1_ESM.pdf]

Supplementary Information

Dearomative triple elementalization of quinolines driven by visible light

Shiho Ishigaki,[†] Yuki Nagashima,*[,†,‡] Daiki Yukimori,[‡] Jin Tanaka,[†] Takashi Matsumoto,[§] Kazunori Miyamoto,[‡] Masanobu Uchiyama*[,‡,⊥], and Ken Tanaka*[,†]

[†] Department of Chemical Science and Engineering, Tokyo Institute of Technology, O-okayama, Meguro-ku, Tokyo 152-8550, Japan
[‡] Graduate School of Pharmaceutical Sciences, The University of Tokyo, 7-3-1 Hongo, Bunkyo-ku, Tokyo 113-0033, Japan
[§] Rigaku Corporation, 3-9-12 Matsubara-cho, Akishima, Tokyo 196-8666, Japan
[⊥] Research Initiative for Supra-Materials (RISM), Shinshu University, 3-15-1 Tokida, Ueda, Nagano 386-8567, Japan

Table of Contents

1. General 2
2. Experimental Section 4
   2.1. Dearomative triple elementalization of quinolines (Fig. 2) 4
   2.2. Silaborated tetrahydroquinolines as synthetic platform and its asymmetric synthesis (Fig. 3) 13
   2.3. Dearomative triple elementalization of anthracene and phenanthrene (Fig. 5) 17
3. Computational Details 19
4. Single Crystal X-ray Structure 21
5. HPLC, GCMS, and ESI-MS Data 24
6. Copies of NMR Spectra 29
7. Supplementary References 71

1. General

Instrumentation.

Photoreactions were performed in a Schlenck tube using a blue LED light (Kessil A160WE TUNA Blue) or Hg lamp (YMC-P-0066). Normal-phase column chromatography was performed with YAMAZEN YFLC AI-580. Preparative thin-layer chromatography was performed with silica gel 60 from Merck. Preparative recycling gel permeation chromatography (GPC) was performed with JAI LC-5060 / JAIGEL-2HR columns × 2 (eluent: $CHCl_3$).

^{1}H, ^{13}C, ^{19}F, and ^{11}B NMR spectra were collected on a Bruker AVANCE III HD 400 spectrometer or Bruker AVANCE III HD 500 spectrometer at ambient temperature. Chemical shifts are expressed in δ (ppm) values and coupling constants are expressed in hertz (Hz). All ^{1}H NMR experiments are reported in δ units, parts per million (ppm), and were measured relative to the signals for residual chloroform (7.26 ppm). All ^{13}C NMR spectra are reported in ppm relative to deuterochloroform (77.16 ppm) and were obtained with ^{1}H decoupling. The following abbreviations are used: s = singlet, d = doublet, t = triplet, m = multiplet, and bs = broad singlet. EI mass spectra with gas clomatography were measured on an Agilent 5977B spectrometer. ESI mass spectra (HRMS analyses) were measured on a Bruker micrOTOF-II spectrometer. IR spectra were obtained on a JASCO FT/IR-4700 spectrometer. Melting points were determined with a Mettler Toledo MP50 One Click Melting Point System and uncorrected. HPLC analyses were carried out on a Jasco LC-2000Plus Series system using Daicel CHIRALPAK® columns (internal diameter 4.6 mm, column length 250 mm, and particle size 3 or 5 μm) or SUMICHIRAL OA-3100 (internal diameter 4.6 mm, column length 250 mm, and particle size 5 μm). Optical rotation data were obtained with a Jasco P-2200 digital polarimeter at the sodium D line (589 nm) at ambient temperature.

Materials.

Unless otherwise noted, materials were purchased from Aldrich Inc., FUJIFILM Wako Pure Chemical Co., Tokyo Kasei Co., and other commercial suppliers and were used after appropriate purification. Anhydrous solvents were purchased from Kanto Chemical Co. Ltd. (Dimethylphenylsilyl)boronic acid pinacol ester [$PhMe_2$Si–B(pin)] (**2a**) was purchased from Tokyo Kasei Co., and stored under argon atmosphere. Other silylboranes including Et_3Si–B(pin) **2b**,[1] ^{t}BuMe_2Si–B(pin) **2c**,[1] and ^{t}Bu$_2$HSi–B(pin) **2d**[1] were prepared according to the below procedure and stored under argon atmosphere. All other chemicals were of reagent grade and used as received. Air- and moisture-sensitive manipulations were performed with standard Schlenk techniques under argon atmosphere.

Synthesis of Et_3Si–B(pin) 2b:

To a dried Schlenk tube were added $B_2(pin)_2$ (1.33 g, 5.13 mmol), triethylsilane (3.28 mL, 2.06. mmol), $[Ir(cod)(OMe)]_2$ (16.9 mg, 0.0256 mmol), 4,4’-di-*tert*-butyl-2,2’-bipyridil (13.9 mg, 0.0517 mmol), and cyclohexane (4.2 mL). The Schlenk tube was heated to 80 °C for 18 hours. The mixture was cooled down to room temperature and dried in vacuo. The crude mixture was purified by a silica gel column chromatography using hexane/AcOEt to give **2b** (0.468 g, 1.9 mmol, 92% isolated yield) as a colorless oil.

Synthesis of ^{t}BuMe$_2$Si–B(pin) 2c:

To a dried Schlenk tube were added $B_2(pin)_2$ (3.17 g, 12.5 mmol), *tert*-butyldimethylsilane (581.4 mg, 5.0 mmol), Pt/C (31.7 mg, 0.15 mmol), and cyclohexane (5 mL). The Schlenk tube was heated to 80 °C for 18 hours. The mixture was cooled down to room temperature and was diluted with $CHCl_3$, and the white precipitate was filtered. The crude mixture was purified by a silica gel column chromatography using hexane/AcOEt to give **2c** (0.41 g, 1.7 mmol, 34% isolated yield) as a white solid.

Synthesis of ^{t}Bu$_2$HSi–B(pin) 2d:

To a dried Schlenk tube were added B_2(pin)$_2$ (1.02 g, 4.00 mmol), di-*tert*-butylsilane (0.4 mL, 2.00 mmol), [Ir(cod)(OMe)]$_2$ (6.7 mg, 0.01 mmol), 4,4'-di-*tert*-butyl-2,2'-bipyridil (5.4 mg, 0.02 mmol), and cyclohexane (4.0 mL). The Schlenk tube was heated to 80 °C for 18 hours. The mixture was cooled down to room temperature and dried in vacuo. The crude mixture was purified by a silica gel column chromatography using hexane/AcOEt to give **2d** (0.950 g, 3.52 mmol, 88% isolated yield) as a colorless oil.

2. Experimental Section

2.1. Dearomative triple elementalization of quinolines (Fig. 2)

Dearomative carbo-sila-boration of quinolines (Fig. 2a)

Typical Procedure A:

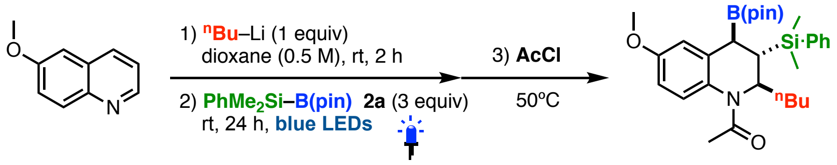

1a **3aa** (*rac*)

6-Methoxyquinoline **1a** (0.25 mmol) was charged in a dried Schlenk tube and dissolved in dry dioxane (0.5 mL, 0.5 M). To the mixture was added ^{n}BuLi (0.10 mL, 0.25 mmol; 2.55 M in hexane solution) at 0 ºC, and the solution was stirred for 2 hours. To the mixture was successively added (dimethylphenylsilyl)boronic acid pinacol ester [$PhMe_2Si$–B(pin)] (**2a**) (0.197 g, 0.75 mmol) at room temperature. After the reaction tube was sealed and irradiated for 24 hours with blue LEDs equipped with a cooling fan at room temperature. To the mixture was successively added acetyl chloride (0.1 mL) at room temperature and the mixture was stirred at 50°C for 24 hours. The reaction was quenched by H_2O (2 mL) and extracted with AcOEt (10 mL × 3). The AcOEt layers were combined and dried over $MgSO_4$, and the solvent was removed under reduced pressure. The residue was purified by a preparative TLC (n–hexane/AcOEt/toluene/dichloromathane = 3:1:3:3) to give **3aa** (0.075 g, 0.15 mmol, 58% isolated yield, 88% NMR yield) as a white solid.

1-((2*S*,3*R*,4*S*)-2-butyl-3-(dimethyl(phenyl)silyl)-6-methoxy-4-(4,4,5,5-tetramethyl-1,3,2-dioxaborolan-2-yl)-3,4-dihydroquinolin-1(2*H*)-yl)ethan-1-one (racemic mixture of (2*S*,3*R*,4*S*) and (2*R*,3*S*,4*R*)) (3aa)
Using the **Typical Procedure A**, the titled compound was obtained as a white solid. **mp** 89.2–91.0 ºC. **^{1}H NMR ($CDCl_3$, 500 MHz):** 7.56 (dd, *J* = 6.4, 3.0 Hz, 2H), 7.32-7.31 (m, 3H), 6.85-6.83 (m, 2H), 6.66 (dd, *J* = 2.5, 8.6 Hz, 1H), 5.12-5.09 (m, 1H), 3.77 (s, 3H), 2.45 (d, *J* = 5.5 Hz, 1H), 2.00 (s, 3H), 1.54 (dd, *J* = 5.5, 2.0 Hz, 1H), 1.22 (s, 6H), 1.21 (s, 6H), 1.17-1.04 (m, 6H), 0.74 (t, *J* = 6.9 Hz, 3H), 0.35 (s, 3H), 0.28 (s, 3H). **^{13}C NMR ($CDCl_3$, 125 MHz):** 169.0, 156.7, 138.0, 135.0, 134.3, 130.7, 128.9, 127.7, 126.0, 113.4, 111.2, 83.8, 55.3, 50.1, 35.1, 28.3, 28.2, 25.2, 24.9, 23.2, 22.4, 14.0, -3.74, -4.60. The carbon directly attached to the boron atom was not detected, likely due to quadrupolar relaxation. **^{11}B NMR ($CDCl_3$, 160 MHz):** 33.7. $C_{30}H_{44}BNO_4Si$. **HRMS (pos. ESI) *m*/*z*:** calcd for $[M+Na]^+$ 544.3030, found 544.3053. **ATR-FTIR (neat) ν:** 2954, 1647, 1500, 1326, 1251, 1141, 813, 733, 700, 424, 409 cm^{-1}. The relative configuration was determined by X-ray structural analysis.

1-((2*S*,3*R*,4*S*)-2-butyl-3-(dimethyl(phenyl)silyl)-4-(4,4,5,5-tetramethyl-1,3,2-dioxaborolan-2-yl)-3,4-dihydroquinolin-1(2*H*)-yl)ethan-1-one (racemic mixture of (2*S*,3*R*,4*S*) and (2*R*,3*S*,4*R*)) (3ba)

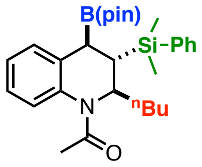

Using the **Typical Procedure A**, the titled compound (0.091 g, 0.19 mmol, 74% isolated yield, 91% NMR yield) was obtained as a colorless oil. **^{1}H NMR ($CDCl_3$, 400 MHz):** 7.57-7.55 (m, 2H), 7.36-7.29 (m, 3H), 7.26-7.24 (m, 1H), 7.13-7.07 (m, 2H), 7.00-6.89 (m, 1H), 5.22-5.06 (m, 1H), 2.50 (d, *J* = 3.8 Hz, 1H), 2.02 (s, 3H), 1.66-1.60 (m, 1H), 1.22 (s, 12H). 1.19-1.02 (m, 6H), 0.74 (t, *J* = 6.4 Hz, 3H), 0.34 (s, 3H), 0.27 (s, 3H).**^{13}C NMR ($CDCl_3$, 100 MHz):** 169.1, 137.9, 137.5, 134.3, 133.6, 129.0, 128.8, 127.7, 125.2, 125.1, 124.9, 83.8, 50.1, 35.1, 28.4, 28.1, 25.1, 24.9, 23.4, 22.4, 14.0, -3.70, -4.57. The carbon directly attached to the boron atom was not detected, likely due to quadrupolar relaxation. **^{11}B NMR ($CDCl_3$, 160 MHz):** 33.3. $C_{29}H_{42}BNO_3Si$. **HRMS (pos. ESI) *m*/*z*:** calcd for $[M+Na]^+$ 514.2925 found 514.2925.

1-((2*S*,3*R*,4*S*)-2-butyl-3-(dimethyl(phenyl)silyl)-6-methyl-4-(4,4,5,5-tetramethyl-1,3,2-dioxaborolan-2-

yl)-3,4-dihydroquinolin-1(2*H*)-yl)ethan-1-one (racemic mixture of (2*S*,3*R*,4*S*) and (2*R*,3*S*,4*R*)) (3ca)

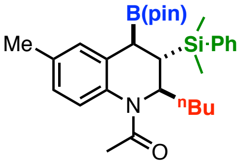

Using the **Typical Procedure A**, the titled compound (0.081 g, 0.16 mmol, 64% isolated yield, 76% NMR yield) was obtained as a colorless oil. **^{1}H NMR ($CDCl_3$, 400 MHz):** 7.58-7.54 (m, 2H), 7.35-7.30 (m, 3H), 7.06 (s, 1H), 6.90 (dt, *J* = 0.8, 8.0 Hz ,1H), 6.81 (d, *J* = 8.0 Hz, 1H), 5.14-5.06 (m, 1H), 2.44 (d, *J* = 5.5 Hz, 1H), 2.29 (s, 3H), 2.01 (s, 3H), 1.55-1.51 (m, 1H), 1.22 (s, 6H), 1.21 (s, 6H), 1.20-1.10 (m, 6H), 0.74 (t, *J* = 6.6 Hz, 3H), 0.35 (s, 3H), 0.27 (s, 3H). **^{13}C NMR ($CDCl_3$, 100 MHz):** 169.1, 138.0, 135.0, 134.4, 134.3, 133.5, 129.2, 128.9, 127.7, 125.8, 125.0, 83.7, 50.1, 35.1, 28.3, 28.2, 25.1, 24.8, 23.3, 22.3, 21.0, 14.0, -3.83, -4.59. The carbon directly attached to the boron atom was not detected, likely due to quadrupolar relaxation. **^{11}B NMR ($CDCl_3$, 160 MHz):** 33.8. $C_{30}H_{44}BNO_3Si$. **HRMS (pos. ESI) *m/z*:** calcd for $[M+Na]^+$ 528.3081, found 528.3076.

1-((2*S*,3*R*,4*S*)-2-butyl-3-(dimethyl(phenyl)silyl)-7-methyl-4-(4,4,5,5-tetramethyl-1,3,2-dioxaborolan-2-yl)-3,4-dihydroquinolin-1(2*H*)-yl)ethan-1-one (racemic mixture of (2*S*,3*R*,4*S*) and (2*R*,3*S*,4*R*)) (3da)

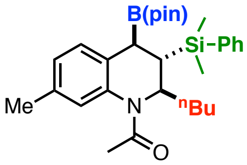

Using the **Typical Procedure A**, the titled compound (0.051 g, 0.10 mmol, 40% isolated yield, 52% NMR yield) was obtained as a colorless oil. **^{1}H NMR ($CDCl_3$, 400 MHz):** 7.59-7.54 (m, 2H), 7.35-7.29 (m, 3H), 7.12 (d, *J* = 7.8 Hz, 1H), 6.89 (d, *J* = 7.9 Hz, 1H), 6.72-6.71 (m, 1H), 5.16-5.06 (m, 1H), 2.44-2.43 (m, 1H), 2.29 (s, 3H), 2.03 (s, 3H), 1.55-1.54 (m, 1H), 1.21 (s, 6H), 1.20 (s, 6H), 1.19-1.05 (m, 6H), 0.75 (t, *J* = 5.6 Hz, 3H), 0.33 (s, 3H), 0.26 (s, 3H). **^{13}C NMR ($CDCl_3$, 100 MHz):** 169.1, 138.0, 137.3, 137.3, 134.6, 134.3, 130.3, 128.9, 128.5, 127.7, 125.8, 83.7, 50.0, 35.0, 28.4, 28.0, 25.0, 24.8, 23.4, 22.4, 21.1, 14.0, -3.73, -4.59. The carbon directly attached to the boron atom was not detected, likely due to quadrupolar relaxation. **^{11}B NMR ($CDCl_3$, 160 MHz):** 32.9. $C_{30}H_{44}BNO_3Si$. **HRMS (pos. ESI) *m/z*:** calcd for $[M+Na]^+$ 528.3081, found 528.3074.

1-((2*S*,3*R*,4*S*)-2-butyl-3-(dimethyl(phenyl)silyl)-8-methyl-4-(4,4,5,5-tetramethyl-1,3,2-dioxaborolan-2-yl)-3,4-dihydroquinolin-1(2*H*)-yl)ethan-1-one (racemic mixture of (2*S*,3*R*,4*S*) and (2*R*,3*S*,4*R*)) (3ea)

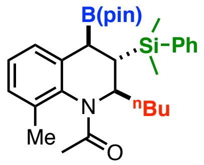

Using the **Typical Procedure A**, the titled compound (0.051 g, 0.10 mmol, 40% isolated yield, 73% NMR yield) was obtained as a colorless oil. **^{1}H NMR ($CDCl_3$, 400 MHz):** 7.65-7.58 (m, 2H), 7.37-7.32 (m, 3H), 7.09 (m, 3H), 5.08-5.01 (m, 1H), 2.20 (s, 3H), 2.04 (d, *J* = 12.2 Hz, 1H), 1.81 (s, 3H), 1.32 (d, *J* = 8.0 Hz, 1H), 1.28 (s, 6H), 1.27 (s, 6H), 1.15-0.73 (m, 6H), 0.66 (t, *J* = 7.0 Hz, 3H), 0.43 (s, 3H), 0.42 (s, 3H). **^{13}C NMR ($CDCl_3$, 100 MHz):** 170.3, 139.8, 138.5, 138.1, 134.4, 134.3, 129.0, 128.4, 127.7, 126.2, 124.0, 84.0, 53.4, 36.3 32.6, 28.8, 26.2, 25.1, 22.4, 21.9, 17.7, 13.9, -2.79, -4.06. The carbon directly attached to the boron atom was not detected, likely due to quadrupolar relaxation. **^{11}B NMR ($CDCl_3$, 160 MHz):** 33.4. $C_{30}H_{44}BNO_3Si$. **HRMS (pos. ESI) *m/z*:** calcd for $[M+Na]^+$ 528.3081, found 528.3080.

1-((2*S*,3*R*,4*S*)-2-butyl-3-(dimethyl(phenyl)silyl)-5-methyl-4-(4,4,5,5-tetramethyl-1,3,2-dioxaborolan-2-yl)-3,4-dihydroquinolin-1(2*H*)-yl)ethan-1-one (racemic mixture of (2*S*,3*R*,4*S*) and (2*R*,3*S*,4*R*)) (3fa)

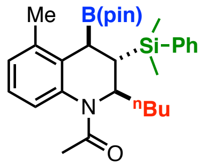

Using the **Typical Procedure A** [**1f** (0.10 mmol), **2a** (0.30 mmol), ^{n}BuLi (0.10 mmol), and dioxane (0.3 mL) were used.], the titled compound (0.013 g, 0.025 mmol, 25% isolated yield, 66% NMR yield) was obtained as a colorless oil. **^{1}H NMR ($CDCl_3$, 400 MHz):** 7.56-7.48 (m, 2H), 7.32-7.27 (m, 3H), 6.99-6.90 (m, 2H), 6.76-6.62 (m, 1H), 5.34-4.90 (m, 1H). 2.71-2.63 (m, 1H), 2.21 (s, 3H), 1.89 (s, 3H), 1.70-1.64 (m, 1H), 1.38-1.21 (m, 6H), 1.21 (s, 6H), 1.18 (s, 6H), 0.80 (t, *J* = 7.0 Hz, 3H), 0.27 (s, 3H), 0.23 (s, 3H). **^{13}C NMR ($CDCl_3$, 100 MHz):** 169.7, 137.8, 136.6, 135.9, 135.8, 134.2, 128.9, 127.7, 127.6, 126.3, 124.0, 83.4, 34.6, 28.81, 28.79, 27.2, 24.8, 24.8, 24.0, 22.6, 20.3, 14.1, -3.01, -4.51. The carbon directly attached to the boron atom was not detected, likely due to quadrupolar relaxation. **^{11}B NMR ($CDCl_3$, 160 MHz):** 33.3. $C_{30}H_{44}BNO_3Si$. **HRMS

(pos. ESI) ***m/z*****:** calcd for $[M+Na]^+$ 528.3081, found 528.3112.

1-((2*S*,3*R*,4*S*)-2-butyl-3-(dimethyl(phenyl)silyl)-6-fluoro-4-(4,4,5,5-tetramethyl-1,3,2-dioxaborolan-2-yl)-3,4-dihydroquinolin-1(2*H*)-yl)ethan-1-one (racemic mixture of (2*S*,3*R*,4*S*) and (2*R*,3*S*,4*R*)) (3ga)

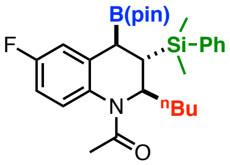

Using the **Typical Procedure A**, the titled compound (0.082 g, 0.16 mmol, 64% isolated yield, 71% NMR yield) was obtained as a colorless oil. **^{1}H NMR ($CDCl_3$, 400 MHz):** 7.57-7.54 (m, 2H), 7.35-7.29 (m, 3H), 7.14-7.05 (m, 1H), 7.04-7.02 (m, 1H), 6.92-7.78 (m, 1H), 5.16-5.09 (m, 1H), 2.50-2.47 (m, 1H), 2.00 (d, *J* = 16.4 Hz, 3H), 1.26-1.24 (m, 1H), 1.21 (s, 12H), 1.16-1.01(m, 6H), 0.74 (t, *J* = 5.1 Hz, 3H), 0.34 (s, 3H), 0.27 (s, 3H). **^{13}C NMR ($CDCl_3$, 100 MHz):** 169.1, 169.0, 159.8 (d, *J* = 243.6Hz), 137.7, 135.6 (d, *J* = 9.3 Hz), 134.2, 133.4, 127.7, 126.1 (d, *J* = 8.3 Hz), 125.1, 115.5 (d, *J* = 22.8 Hz), 112.1 (d, *J* = 22.8 Hz), 84.0, 83.8, 50.0, 34.9, 28.3, 27.8, 25.0, 24.8, 23.4, 23.3, 22.3, 14.0, -3.63, -4.70. The carbon directly attached to the boron atom was not detected, likely due to quadrupolar relaxation. **^{19}F NMR ($CDCl_3$, 376 MHz): −**117.4. **^{11}B NMR ($CDCl_3$, 160 MHz):** 32.3. $C_{29}H_{41}BFNO_3Si$. **HRMS (pos. ESI)** ***m/z*****:** calcd for $[M+Na]^+$ 532.2831, found 532.2851.

1-((2*S*,3*R*,4*S*)-2-butyl-3-(dimethyl(phenyl)silyl)-8-fluoro-4-(4,4,5,5-tetramethyl-1,3,2-dioxaborolan-2-yl)-3,4-dihydroquinolin-1(2*H*)-yl)ethan-1-one (racemic mixture of (2*S*,3*R*,4*S*) and (2*R*,3*S*,4*R*)) (3ha)

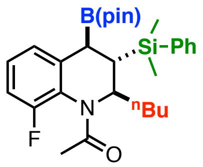

Using the **Typical Procedure A** (reaction time = 96 h), the titled compound (0.024 g, 0.048 mmol, 19% isolated yield, 26% NMR yield) was obtained as a colorless oil. **^{1}H NMR ($CDCl_3$, 400 MHz):** 7.60-7.57 (m, 2H), 7.37-7.31 (m, 3H), 7.12-7.05 (m, 2H), 6.97-6.91 (m, 1H), 5.11-5.04 (m, 1H), 2.35 (d, *J* = 8.1 Hz, 1H), 1.92 (m, 3H), 1.43-1.38 (m, 1H), 1.24 (s, 6H), 1.23 (s, 6H), 1.19-0.91 (m, 6H), 0.70 (t, *J* = 6.9 Hz, 3H), 0.38 (s, 3H), 0.35 (s, 3H). **^{13}C NMR ($CDCl_3$, 100 MHz):** 169.9, 156.5 (d, *J* = 248.0 Hz), 138.7, 137.7, 134.4, 129.1, 127.8, 126.7 (d, *J* = 11.5 Hz), 126.2 (d, *J* = 8.4 Hz), 123.2 (d, *J* = 3.1 Hz), 113.3 (m, *J* = 20.8 Hz), 84.0, 50.9, 36.0, 30.3, 28.0, 25.5, 25.0, 22.2, 21.8, 21.7, 13.9, -3.98, -4.15. **^{19}F NMR ($CDCl_3$, 376 MHz): −**121.9. **^{11}B NMR ($CDCl_3$, 160 MHz):** 33.8. $C_{29}H_{41}BFNO_3Si$. **HRMS (pos. ESI)** ***m/z*****:** calcd for $[M+Na]^+$ 532.2831, found 532.2851.

1-((2*S*,3*R*,4*S*)-2-butyl-3-(dimethyl(phenyl)silyl)-6-(dimethylamino)-4-(4,4,5,5-tetramethyl-1,3,2-dioxaborolan-2-yl)-3,4-dihydroquinolin-1(2*H*)-yl)ethan-1-one (racemic mixture of (2*S*,3*R*,4*S*) and (2*R*,3*S*,4*R*)) (3ia)

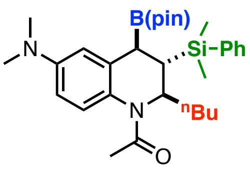

Using the **Typical Procedure A** [**1i** (0.10 mmol), **2a** (0.30 mmol), ^{n}BuLi (0.10 mmol), and dioxane (0.3 mL) were used.], the titled compound (0.0075 g, 0.014 mmol, 14% isolated yield, 67% NMR yield) was obtained as a colorless oil. **^{1}H NMR ($CDCl_3$, 400 MHz):** 7.60-7.55 (m, 2H), 7.35-7.30 (m, 3H), 6.80 (d, *J* = 8.7 Hz, 1H), 6.65 (d, *J* = 1.8 Hz, 1H), 6.60 (dd, *J* = 5.9, 11.0 Hz, 1H), 5.12-5.04 (m, 1H), 2.92 (s, 6H), 2.42 (d, *J* = 6.2 Hz, 1H), 2.02 (s, 3H), 1.50 (dd, *J* = 6.0, 2.2 Hz, 1H), 1.22 (s, 6H), 1.20 (s, 6H), 1.18-1.03 (m, 6H), 0.73 (t, *J* = 3.1 Hz, 3H) , 0.36 (s, 3H), 0.28 (s, 3H). **^{13}C NMR ($CDCl_3$, 100 MHz):** 169.0, 148.1, 138.2, 134.7, 134.3, 128.9, 127.7, 127.5, 126.0, 112.3, 109.7, 83.6, 50.2, 40.8, 35.2, 28.4, 28.3, 25.3, 25.0, 23.2, 22.4, 14.0, -3.84, -4.53. The carbon directly attached to the boron atom was not detected, likely due to quadrupolar relaxation. **^{11}B NMR ($CDCl_3$, 160 MHz):** 33.8. $C_{31}H_{47}BN_2O_3Si$. **HRMS (pos. ESI)** ***m/z*****:** calcd for $[M+Na]^+$ 557.3347, found 557.3360.

1-((2*S*,3*R*,4*S*)-2-butyl-6-chloro-3-(dimethyl(phenyl)silyl)-4-(4,4,5,5-tetramethyl-1,3,2-dioxaborolan-2-yl)-3,4-dihydroquinolin-1(2H)-yl)ethan-1-one (racemic mixture of (2*S*,3*R*,4*S*) and (2*R*,3*S*,4*R*)) (3ja)

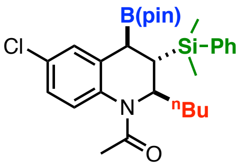

Using the **Typical Procedure A** (reaction time = 48 h, THF was used instead of dioxane as a solvent.), the titled compound (0.016 g, 0.030 mmol, 12% isolated yield, 41% NMR yield) was obtained as a colorless oil. **^{1}H NMR (CDCl$_3$, 400 MHz):** 7.75-7.71 (m, 1H), 7.56-7.53 (m, 2H), 7.52-7.49 (m, 1H), 7.32-7.31 (m, 3H), 6.96-6.87 (m, 1H), 5.18-5.00 (m, 1H), 2.48-2.47 (m, 1H), 2.00 (s, 3H), 1.36-1.34 (m, 1H), 1.32 (s, 6H), 1.30 (s, 6H), 1.19-1.01 (m, 6H), 0.73 (t, *J* = 6.7 Hz, 3H), 0.33 (s, 3H), 0.27 (s, 3H). **^{13}C NMR (CDCl$_3$, 100 MHz):** 169.2, 137.8, 135.8, 134.3, 133.9, 132.2, 131.4, 129.0, 127.7, 127.6, 124.6, 83.7, 83.6, 50.3, 35.4, 28.4, 25.1, 24.9, 23.3, 22.7, 22.3, 14.0, -4.04, -4.54. The carbon directly attached to the boron atom was not detected, likely due to quadrupolar relaxation. **^{11}B NMR (CDCl$_3$, 160 MHz):** 33.3. $C_{29}H_{41}BClNO_3Si$. **HRMS (pos. ESI) *m*/*z*:** calcd for $[M+Na]^+$ 548.2535 found 548.2528.

1-((2*S*,3*R*,4*S*)-2-butyl-3-(dimethyl(phenyl)silyl)-4-(4,4,5,5-tetramethyl-1,3,2-dioxaborolan-2-yl)-6-(trimethylsilyl)-3,4-dihydroquinolin-1(2H)-yl)ethan-1-one (racemic mixture of (2*S*,3*R*,4*S*) and (2*R*,3*S*,4*R*)) (3ka)

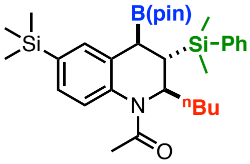

Using the **Typical Procedure A** [**1k** (0.10 mmol), **2a** (0.30 mmol), ^{n}BuLi (0.10 mmol), and dioxane (0.3 mL) were used.], the titled compound (0.023 g, 0.041 mmol, 41% isolated yield, 56% NMR yield) was obtained as a colorless oil. **^{1}H NMR (CDCl$_3$, 400 MHz):** 7.60-7.54 (m, 2H), 7.42-7.37 (m, 1H), 7.36-7.29 (m, 3H), 7.22 (d, *J* = 7.9 Hz, 1H), 6.89 (d, *J* = 7.3 Hz, 1H), 5.20-5.05 (m, 1H), 2.50 (d, *J* = 3.4 Hz, 1H), 2.05 (s, 3H), 1.67-1.60 (m, 1H), 1.23 (s, 6H). 1.21 (s, 6H), 1.20-1.02 (m, 6H), 0.75 (t, *J* = 6.1 Hz, 3H), 0.34 (s, 3H), 0.27 (s, 3H), 0.25 (s, 9H). **^{13}C NMR (CDCl$_3$, 100 MHz):** 169.1, 138.1, 138.0, 136.5, 134.3, 134.0, 132.6, 130.0, 128.9, 127.7, 124.5, 83.7, 50.1, 35.2, 28.4, 27.9, 25.2, 24.9, 23.4, 22.4, 21.6, 14.0, -1.00, -3.75, -4.57. **^{11}B NMR (CDCl$_3$, 160 MHz):** 32.1. $C_{32}H_{50}BNO_3Si_2$. **HRMS (pos. ESI) *m*/*z*:** calcd for $[M+Na]^+$ 586.3320, found 586.3303.

1-((2*S*,3*R*,4*S*)-2-butyl-3-(dimethyl(phenyl)silyl)-6-isopropyl-4-(4,4,5,5-tetramethyl-1,3,2-dioxaborolan-2-yl)-3,4-dihydroquinolin-1(2H)-yl)ethan-1-one (racemic mixture of (2*S*,3*R*,4*S*) and (2*R*,3*S*,4*R*)) (3la)

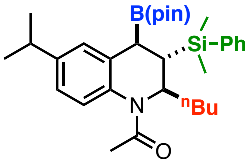

Using the **Typical Procedure A**, the titled compound (0.065 g, 0.16 mmol, 65% isolated yield, 72% NMR yield) was obtained as a colorless oil. **^{1}H NMR (CDCl$_3$, 400 MHz):** 7.60-7.53 (m, 2H), 7.35-7.30 (m, 3H), 7.14-7.10 (m, 1H), 6.94 (dd, *J* = 8.1, 1.8 Hz, 1H), 6.83 (d, *J* = 8.1 Hz, 1H), 5.17-5.04 (m, 1H), 2.90-2.77 (m, 1H), 2.45 (d, *J* = 5.4 Hz, 1H), 2.03 (s, 3H), 1.74-1.68 (m, 1H), 1.24 (m, 3H), 1,23 (s, 6H), 1,22 (m, 3H), 1.21 (s, 6H), 1.19-1.03 (m, 6H), 0.74 (t, *J* = 6.7 Hz, 3H), 0.35 (s, 3H), 0.27 (s, 3H). **^{13}C NMR (CDCl$_3$, 100 MHz):** 169.1, 145.4, 138.0, 135.2, 134.3, 133.5, 128.9, 127.7, 126.4, 125.0, 123.4, 83.7, 50.1, 35.2, 33.7, 28.4, 28.2, 25.2, 24.9, 24.2, 23.8, 23.3, 22.3, 14.0, -3.82, -4,55. The carbon directly attached to the boron atom was not detected, likely due to quadrupolar relaxation. **^{11}B NMR (CDCl$_3$, 160 MHz):** 32.2. $C_{32}H_{48}BNO_3Si$. **HRMS (pos. ESI) *m*/*z*:** calcd for $[M+Na]^+$ 536.3394, found 556.3414.

1-((2*S*,3*R*,4*S*)-2-butyl-3-(dimethyl(phenyl)silyl)-6-(4-methoxyphenyl)-4-(4,4,5,5-tetramethyl-1,3,2-dioxaborolan-2-yl)-3,4-dihydroquinolin-1(2*H*)-yl)ethan-1-one (racemic mixture of (2*S*,3*R*,4*S*) and (2*R*,3*S*,4*R*)) (3ma)

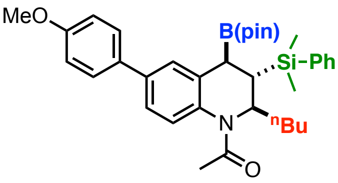

Using the **Typical Procedure A** [**1m** (0.10 mmol), **2a** (0.30 mmol), ^{n}BuLi (0.10 mmol), and dioxane (0.3 mL) were used.], the titled compound (0.017 g, 0.028 mmol, 28% isolated yield, 47% NMR yield) was obtained as a colorless oil. **^{1}H NMR (CDCl$_3$, 400 MHz):** 7.60-7.53 (m, 2H), 7.52-7.50 (m, 1H), 7.50-7.45 (m, 2H), 7.37-7.30 (m, 3H), 7.28 (ddd, *J* = 0.8, 2.1, 8.3 Hz, 1H), 6.98-6.92 (m, 3H), 5.27-5.00 (m, 1H), 3.84 (s, 3H), 2.55 (d, *J* = 3.5 Hz, 1H), 2.07 (s, 3H), 1.71-1.64 (m, 1H), 1.23 (s, 12H), 1.22-1.01 (m, 6H), 0.76 (t, *J* = 6.0, 3H), 0.36 (s, 3H), 0.28 (s, 3H). **^{13}C NMR (CDCl$_3$, 100**

MHz): 169.1, 159.0, 137.9, 137.2, 136.2, 134.3, 133.6, 133.3, 128.9, 127.8, 127.7, 127.1, 125.4, 123.4, 114.2, 83.8, 55.3, 50.1, 35.1, 28.4, 28.0, 25.1, 24.9, 23.5, 22.4, 14.0, -3.67, -4.63. The carbon directly attached to the boron atom was not detected, likely due to quadrupolar relaxation. **^{11}B NMR (CDCl$_3$, 160 MHz):** 32.8. $C_{36}H_{48}BNNaO_4Si$. **HRMS (pos. ESI) *m/z*:** calcd for [M+Na]$^+$ 620.3364 found 620.3364.

1-((2*S*,3*R*,4*S*)-2-butyl-3-(dimethyl(phenyl)silyl)-4-(4,4,5,5-tetramethyl-1,3,2-dioxaborolan-2-yl)-6-(thiophen-3-yl)-3,4-dihydroquinolin-1(2*H*)-yl)ethan-1-one (racemic mixture of (2*S*,3*R*,4*S*) and (2*R*,3*S*,4*R*)) (3na)

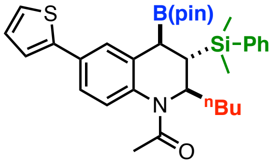

Using the **Typical Procedure A** [**1n** (0.10 mmol), **2a** (0.30 mmol), ^{n}BuLi (0.10 mmol), and dioxane (0.3 mL) were used.], the titled compound (0.013 g, 0.023 mmol, 23% isolated yield, 47% NMR yield) was obtained as a colorless oil. **^{1}H NMR (CDCl$_3$, 400 MHz):** 7.59-7.53 (m, 3H), 7.39-7.30 (m, 7H), 7.00-6.89 (m, 1H), 5.21-5.08 (m, 1H), 2.61-2.51 (m, 1H), 2.06 (s, 3H), 1.67-1.61 (m, 1H), 1.23 (s, 12H), 1.21-1.05 (m, 6H), 0.76 (t, *J* = 6.2 Hz, 3H), 0.35 (s, 3H), 0.27 (s, 3H). **^{13}C NMR (CDCl$_3$, 100 MHz):** 169.1, 142.0, 137.9, 136.5, 134.3, 133.6, 132.3, 129.0, 127.7, 127.0, 126.2, 126.0, 125.4, 123.2, 119.6, 83.8, 50.1, 35.0, 28.4, 27.8, 25.1, 24.9, 23.5, 22.4, 14.0, -3.62, -4.71. The carbon directly attached to the boron atom was not detected, likely due to quadrupolar relaxation. **^{11}B NMR (CDCl$_3$, 160 MHz):** 34.2. $C_{33}H_{44}BNNaO_3SSi$. **HRMS (pos. ESI) *m/z*:** calcd for [M+Na]$^+$ 596.2802, found 596.2796.

1-((1*S*,2*R*,3*S*)-3-butyl-2-(dimethyl(phenyl)silyl)-1-(4,4,5,5-tetramethyl-1,3,2-dioxaborolan-2-yl)-2,3-dihydrobenzo[f]quinolin-4(1*H*)-yl)ethan-1-one (racemic mixture of (1*S*,2*R*,3*S*) and (1*R*,2*S*,3*R*)) (3oa)

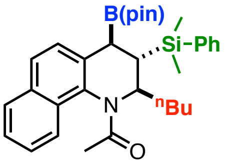

Using the **Typical Procedure A**, the titled compound (0.064 g, 0.12 mmol, 47% isolated yield, 54% NMR yield) was obtained as a white solid. **mp** 133.3–134.2 °C. **^{1}H NMR (CDCl$_3$, 400 MHz):** 7.81-7.75 (m, 2H), 7.59-7.53 (m, 3H), 7.48-7.36 (m, 3H), 7.34-7.27 (m, 2H), 7.14-7.00 (m, 1H), 5.41-5.00 (m, 1H), 3.20-3,13 (m, 1H), 1.94 (s, 3H), 1.79-1.67 (m, 1H), 1.44-1.20 (m, 6H), 1.17 (s, 6H), 1.09 (s, 6H), 0.79 (t, *J* = 6.7 Hz, 3H), 0.33 (s, 3H), 0.30 (s, 3H). **^{13}C NMR (CDCl$_3$, 100 MHz):** 169.6, 137.6, 134.3, 133.0, 133.0, 132.4, 131.0, 129.0, 128.4, 127.7, 125.8, 125.1, 124.8, 124.7, 124.0, 83.4, 49.6, 34.3, 26.9, 24.8, 24.3, 22.6, 19.0, 14.1, -3.10, -4.70. The carbon directly attached to the boron atom was not detected, likely due to quadrupolar relaxation. **^{11}B NMR (CDCl$_3$, 160 MHz):** 31.5. $C_{33}H_{44}BNO_3Si$. **HRMS (pos. ESI) *m/z*:** calcd for [M+Na]$^+$ 564.3081, found 564.3055.

1-((2*S*,3*R*,4*S*)-2-butyl-3-(dimethyl(phenyl)silyl)-4-(4,4,5,5-tetramethyl-1,3,2-dioxaborolan-2-yl)-3,4-dihydrobenzo[h]quinolin-1(2*H*)-yl)ethan-1-one (racemic mixture of (2*S*,3*R*,4*S*) and (2*R*,3*S*,4*R*)) (3pa)

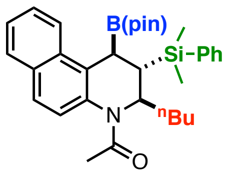

Using the **Typical Procedure A** [**1p** (0.10 mmol), **2a** (0.30 mmol), ^{n}BuLi (0.10 mmol), and dioxane (0.3 mL) were used.], the titled compound (0.012 g, 0.023 mmol, 23% isolated yield, 27% NMR yield) was obtained as a colorless oil. **^{1}H NMR (CDCl$_3$, 400 MHz):** 7.81 (d, *J* = 7.8 Hz, 1H), 7.75 (d, *J* = 8.2 Hz, 1H), 7.65 (dd, *J* = 9.0, 16.7 Hz, 2H), 7.63 (d, *J* = 3.4 Hz, 1H), 7.49-7.44 (m, 1H), 7.42 (d, *J* = 8.4 Hz, 2H), 7.37-7.33 (m, 3H), 5.29-5.19 (m, 1H), 2.40 (d, *J* = 9.5 Hz, 1H), 1.67 (s, 3H), 1.40 (dd, *J* = 4.0, 9.5 Hz, 1H), 1.27 (s, 6H), 1.27 (s, 6H), 1.20-0.84 (m, 6H), 0.63 (t, *J* = 7.3 Hz, 3H), 0.45 (s, 3H), 0.42 (s, 3H). **^{13}C NMR (CDCl$_3$, 100 MHz):** 170.9, 138.1, 135.2, 134.5, 134.4, 132.6, 130.4, 129.0, 128.3, 127.7, 126.3, 126.1, 125.5, 125.2, 122.6, 84.0, 53.1, 36.2, 31.8, 29.2, 25.8, 25.1, 23.6, 22.3, 13.9, -3.44, -4.00. The carbon directly attached to the boron atom was not detected, likely due to quadrupolar relaxation. **^{11}B NMR (CDCl$_3$, 160 MHz):** 33.8. $C_{33}H_{44}BNO_3Si$. **HRMS (pos. ESI) *m/z*:** calcd for [M+Na]$^+$ 564.3081, found 564.3109.

1-((2*S*,3*R*,4*S*)-2-butyl-3-(dimethyl(phenyl)silyl)-4-methyl-4-(4,4,5,5-tetramethyl-1,3,2-dioxaborolan-2-yl)-3,4-dihydroquinolin-1(2H)-yl)ethan-1-one (racemic mixture of (2*S*,3*R*,4*S*) and (2*R*,3*S*,4*R*)) (3qa)

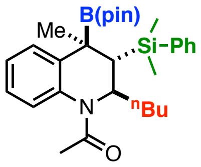

Using the **Typical Procedure A** (reaction time = 48 h), the titled compound (0.054 g, 0.11 mmol, 44% isolated yield, 70% NMR yield) was obtained as a as a white solid. **mp** 138.7-139.4 °C. **^{1}H NMR ($CDCl_3$, 400 MHz):** 7.64-7.57 (m, 2H), 7.39-7.31 (m, 3H), 7.23-7.19 (m, 1H), 7.17-7.12 (m, 2H), 7.00-6.92 (m, 1H), 5.13-4.98 (m, 1H), 2.04 (s, 3H), 1.60 (d, *J* = 4.4 Hz, 1H), 1.40 (s, 6H), 1.31 (s, 6H), 1.25 (s, 3H), 1.04-0.75 (m, 6H), 0.58 (t, *J* = 6.9 Hz, 3H), 0.55 (s, 3H), 0.45 (s, 3H). **^{13}C NMR ($CDCl_3$, 100 MHz):** 169.0, 143.5, 139.2, 138.3, 134.2, 128.9, 127.7, 127.0, 126.3, 126.1, 126.0, 84.2, 51.7, 37.0, 36.5, 27.7, 26.2, 25.4, 23.6, 22.9, 22.1, 13.9, -0.90, -2.82. The carbon directly attached to the boron atom was not detected, likely due to quadrupolar relaxation. **^{11}B NMR ($CDCl_3$, 160 MHz):** 32.9. $C_{30}H_{44}BNO_3Si$. **HRMS (pos. ESI) *m*/*z*:** calcd for $[M+Na]^+$ 528.3081, found 528.3076. The relative configuration was determined by X-ray structural analysis.

1-((2*S*,3*R*,4*S*)-2-butyl-3-(dimethyl(phenyl)silyl)-3-methyl-4-(4,4,5,5-tetramethyl-1,3,2-dioxaborolan-2-yl)-3,4-dihydroquinolin-1(2*H*)-yl)ethan-1-one (racemic mixture of (2*S*,3*R*,4*S*) and (2*R*,3*S*,4*R*)) (3ra)

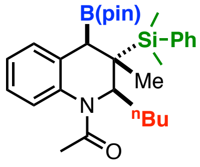

Using the **Typical Procedure A** (reaction time = 48 h), the titled compound (0.033 g, 0.065 mmol, 26% isolated yield, 26% NMR yield) was obtained as a colorless oil. **^{1}H NMR ($CDCl_3$, 400 MHz):** 7.56-7.52 (m, 2H), 7.30-7.24 (m, 3H), 7.21-7.14 (m, 1H), 7.11-7.03 (m, 2H), 6.94-6.87 (m, 1H), 5.07-4.96 (m, 1H), 2.83 (s, 1H), 1.89 (s, 3H), 1.26 (s, 6H), 1.25 (s, 6H), 1.15-1.01 (m, 6H), 0.98 (s, 3H), 0.69 (t, *J* = 6.6 Hz, 3H), 0.36 (s, 3H), 0.35 (s, 3H). **^{13}C NMR ($CDCl_3$, 100 MHz):** 169.1, 137.8, 137.4, 135.1, 134.8, 134.7, 128.8, 127.5, 125.2, 125.1, 124.8, 83.7, 54.4, 29.1, 28.6, 28.3, 25.2, 25.0, 23.5, 22.7, 18.5, 13.9, -4.40, -4.42. The carbon directly attached to the boron atom was not detected, likely due to quadrupolar relaxation. **^{11}B NMR ($CDCl_3$, 160 MHz):** 33.3. $C_{30}H_{44}BNO_3Si$. **HRMS (pos. ESI) *m*/*z*:** calcd for $[M+Na]^+$ 528.3081, found 528.3080.

1-((2*S*,3*R*,4*S*)-2-butyl-6-methoxy-4-(4,4,5,5-tetramethyl-1,3,2-dioxaborolan-2-yl)-3-(triethylsilyl)-3,4-dihydroquinolin-1(2*H*)-yl)ethan-1-one (racemic mixture of (2*S*,3*R*,4*S*) and (2*R*,3*S*,4*R*)) (3ab)

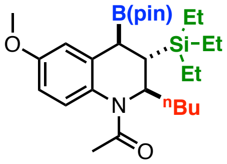

Using the **Typical Procedure A** [**1a** (0.10 mmol), **2b** (0.30 mmol), ^{n}BuLi (0.10 mmol), and dioxane (0.3 mL) were used.], the titled compound (0.0053 g, 0.011 mmol, 11% isolated yield, 45% NMR yield) was obtained as a colorless oil. **^{1}H NMR ($CDCl_3$, 400 MHz):** 6.89 (d, *J* = 8.7 Hz, 1H), 6.81 (d, *J* = 2.1 Hz, 1H), 6.69-6.65 (m, 1H), 5.08-5.00 (m, 1H), 3.78 (s, 3H), 2.48 (d, *J* = 4.2 Hz, 1H), 2.14 (s, 3H), 1.41-1.37 (m, 1H), 1.28 (s, 12H), 1.26-1.11 (m, 6H), 0.93 (t, *J* =7.9 Hz, 9H), 0.80 (t, *J* =7.0 Hz, 3H), 0.59 (q, *J* = 7.9 Hz, 6H). **^{13}C NMR ($CDCl_3$, 100 MHz):** 168.9, 156.6, 134.6, 130.5, 125.9, 113.7, 111.1, 83.8, 55.3, 50.1, 35.0, 28.6, 25.2, 24.8, 24.8, 23.5, 22.5, 14.1, 7.72, 2.54. The carbon directly attached to the boron atom was not detected, likely due to quadrupolar relaxation. **^{11}B NMR ($CDCl_3$, 160 MHz):** 30.1. $C_{28}H_{48}BNO_4Si$. **HRMS (pos. ESI) *m*/*z*:** calcd for $[M+Na]^+$ 524.3343, found 524.3301.

1-((2*S*,3*R*,4*S*)-2-butyl-3-(tert-butyldimethylsilyl)-6-methoxy-4-(4,4,5,5-tetramethyl-1,3,2-dioxaborolan-2-yl)-3,4-dihydroquinolin-1(2*H*)-yl)ethan-1-one (racemic mixture of (2*S*,3*R*,4*S*) and (2*R*,3*S*,4*R*)) (3ac)

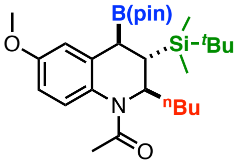

Using the **Typical Procedure A** [**1a** (0.10 mmol), **2c** (0.30 mmol), ^{n}BuLi (0.10 mmol), and dioxane (0.3 mL) were used.], the titled compound (0.0068 g, 0.014 mmol, 14% isolated yield, 36% NMR yield) was obtained as a colorless oil. **^{1}H NMR ($CDCl_3$, 400 MHz):** 6.87-6.86 (m, 1H), 6.82 (d, *J* = 8.8 Hz, 1H), 6.66 (ddd, *J* = 0.8, 2.9, 8.8 Hz, 1H), 5.04-5.02 (m, 1H), 3.79 (s, 3H), 2.58-2.55 (m, 1H), 2.17 (s, 3H), 1.60-1.57 (m, 1H), 1.35-1.10 (m, 6H), 1.26 (s, 12H), 0.95 (s, 9H), 0.80 (t, *J* = 7.0 Hz, 3H), 0.05 (s, 3H), -0.19 (s, 3H). **^{13}C NMR ($CDCl_3$, 100 MHz):** 168.8, 156.3, 133.4, 130.0, 125.5, 113.9, 111.2, 83.8, 55.3, 50.7, 34.6, 28.5, 27.5, 25.0, 25.0, 23.9, 23.7, 22.6, 17.5, 14.1, -6.38, -7.41. The carbon directly attached to the boron atom was not detected, likely due to quadrupolar relaxation. **^{11}B NMR ($CDCl_3$, 160 MHz):** 33.9. $C_{28}H_{48}BNO_4Si$. **HRMS (pos. ESI) *m*/*z*:** calcd for $[M+Na]^+$ 524.3343, found 524.3338.

1-((2*S*,3*R*,4*S*)-2-butyl-3-(di-tert-butylsilyl)-6-methoxy-4-(4,4,5,5-tetramethyl-1,3,2-dioxaborolan-2-yl)-3,4-dihydroquinolin-1(2*H*)-yl)ethan-1-one (racemic mixture of (2*S*,3*R*,4*S*) and (2*R*,3*S*,4*R*)) (3ad)

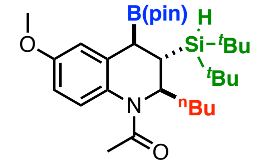

Using the **Typical Procedure A** [**1a** (0.10 mmol), **2d** (0.30 mmol), ^{n}BuLi (0.10 mmol), and dioxane (0.3 mL) were used. 370 nm LED was used for the light source.], the titled compound (0.0059 g, 0.011 mmol, 11% isolated yield, 24% NMR yield) was obtained as a colorless oil. **^{1}H NMR ($CDCl_3$, 400 MHz):** 6.93 (d, *J* = 8.4 Hz, 1H), 6.78-6.70 (m, 2H), 5.29-5.21 (m, 1H), 3.79 (s, 3H), 3.49-3.46 (m, 1H), 2.44 (d, *J* = 12.9 Hz, 1H), 2.03 (s, 3H), 1.40 (s, 6H), 1.38 (s, 6H), 1.32-0.94 (m, 1H), 1.32-0.94 (m, 6H), 1,18 (s, 9H), 1.09 (s, 9H), 0.80 (t, *J* = 7.0 Hz, 3H). **^{13}C NMR ($CDCl_3$, 100 MHz):** 169.3, 157.6, 139.6, 131.8, 126.7, 112.3, 111.3, 83.9, 55.4, 51.4, 37.4, 30.3, 30.2, 29.9, 28.3, 25.8, 25.4, 22.8, 22.5, 21.1, 19.6, 14.1. The carbon directly attached to the boron atom was not detected, likely due to quadrupolar relaxation. **^{11}B NMR ($CDCl_3$, 160 MHz):** 35.0. $C_{28}H_{48}BNO_4Si$. **HRMS (pos. ESI) *m*/*z*:** calcd for $[M+Na]^+$ 552.3656, found 552.3638.

1-((2*S*,3*R*,4*S*)-3-(dimethyl(phenyl)silyl)-6-methoxy-2-methyl-4-(4,4,5,5-tetramethyl-1,3,2-dioxaborolan-2-yl)-3,4-dihydroquinolin-1(2*H*)-yl)ethan-1-one (racemic mixture of (2*S*,3*R*,4*S*) and (2*R*,3*S*,4*R*)) (3ae)

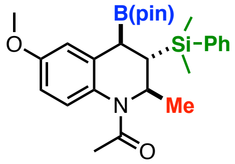

Using the **Typical Procedure A** (MeLi in 3.1 M in diethoxymethane solution was used instead of ^{n}BuLi), the titled compound (0.063 g, 0.13 mmol, 52% isolated yield, 71% NMR yield) was obtained as a yellow oil. **^{1}H NMR ($CDCl_3$, 400 MHz):** 7.59-7.52 (m, 2H), 7.38-7.29 (m, 3H), 6.88-6.80 (m, 2H), 6.70-6.63 (m, 1H), 5.40-5.21 (m, 1H), 3.77 (s, 3H), 2.45 (d, *J* = 3.6 Hz, 1H), 2.00 (s, 3H), 1.53-1.45 (m, 1H), 1.22 (s, 12H), 0.92 (d, *J* = 5.8 Hz, 3H), 0.34 (s, 3H), 0.28 (s, 3H). **^{13}C NMR ($CDCl_3$, 100 MHz):** 168.6, 156.7, 137.8, 135.0, 134.2, 130.5, 129.0, 127.7, 126.0, 113.5, 111.2, 83.9, 55.3, 46.1, 29.9, 25.2, 24.9, 23.2, 21.5, -3.62, -4.47. The carbon directly attached to the boron atom was not detected, likely due to quadrupolar relaxation. **^{11}B NMR ($CDCl_3$, 160 MHz):** 33.7. $C_{27}H_{38}BNO_4Si$. **HRMS (pos. ESI) *m*/*z*:** calcd for $[M+Na]^+$ 502.2561, found 502.2555.

1-((2*S*,3*R*,4*S*)-3-(dimethyl(phenyl)silyl)-6-methoxy-2-phenyl-4-(4,4,5,5-tetramethyl-1,3,2-dioxaborolan-2-yl)-3,4-dihydroquinolin-1(2*H*)-yl)ethan-1-one (racemic mixture of (2*S*,3*R*,4*S*) and (2*R*,3*S*,4*R*)) (3af)

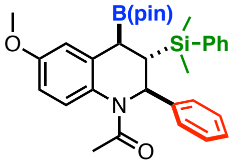

Using the **Typical Procedure A** (PhLi in 1.6 M dibutyl ether solution was used instead of ^{n}BuLi), the titled compound (0.016 g, 0.030 mmol, 12% isolated yield, 19% NMR yield) was obtained as a colorless oil. **^{1}H NMR ($CDCl_3$, 400 MHz):** 7.30-7.10 (m, 10H), 6.90 (d, *J* = 8.5 Hz, 1H), 6.70 (dd, *J* = 2.8, 8.6 Hz, 1H), 6.65 (d, *J* = 2.8, 1H), 5.69 (d, *J* = 12.8 Hz, 1H), 3.81 (s, 3H), 2.19 (d, *J* = 2.7 Hz, 1H), 1.98 (s, 3H), 1.55-1.49 (m, 1H), 1.20 (s, 6H), 1.19 (s, 6H), 0.46 (s, 3H), 0.10 (s, 3H). **^{13}C NMR ($CDCl_3$, 100 MHz):** 169.2, 157.4, 144.2, 143.2, 138.3, 134.0, 131.1, 128.7, 128.3, 128.0, 127.4, 127.1, 126.9, 111.3, 110.8, 83.8, 60.3, 55.4, 38.8, 25.0, 24.9, 23.8, -2.48, -3.55. The carbon directly attached to the boron atom was not detected, likely due to quadrupolar relaxation. **^{11}B NMR ($CDCl_3$, 160 MHz):** 34.4. $C_{32}H_{40}BNO_4Si$. **HRMS (pos. ESI) *m*/*z*:** calcd for $[M+Na]^+$564.2717, found 564.2713.

1-((2*S*,3*R*,4*S*)-2-((*R*)-sec-butyl)-3-(dimethyl(phenyl)silyl)-6-methoxy-4-(4,4,5,5-tetramethyl-1,3,2-dioxaborolan-2-yl)-3,4-dihydroquinolin-1(2*H*)-yl)ethan-1-one (racemic mixture of (2*S*,3*R*,4*S*) and (2*R*,3*S*,4*R*)) & 1-((2*S*,3*R*,4*S*)-2-((*S*)-sec-butyl)-3-(dimethyl(phenyl)silyl)-6-methoxy-4-(4,4,5,5-tetramethyl-1,3,2-dioxaborolan-2-yl)-3,4-dihydroquinolin-1(2*H*)-yl)ethan-1-one (racemic mixture of (2*S*,3*R*,4*S*) and (2*R*,3*S*,4*R*)), mixture (3ag)

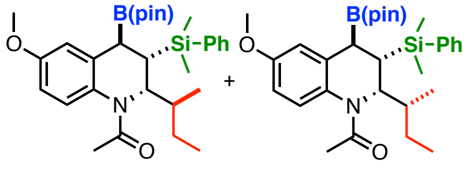

Using the **Typical Procedure A**, the mixture of the titled compounds (0.0078 g, 0.15 mmol, 60% isolated yield, 69% NMR yield) was obtained as a colorless oil. **^{1}H NMR ($CDCl_3$, 400 MHz):** **Compound 1:** 7.60-7.53 (m, 2H), 7.34-7.30 (m, 3H), 6.87-6.81 (m,

2H), 6.68-6.61 (m, 1H), 4.80 (d, *J* = 10.0 Hz, 1H), 3.75 (s, 3H), 2.59-2.55 (m, 1H), 1.99 (s, 3H), 1.94-1.90 (m, 1H), 1.29-0.92 (m, 4H), 1.19 (s, 6H), 1.17 (s, 6H), 0.84-0.74 (m, 3H), 0.70-0.64 (m, 3H), 0.26 (s, 3H), 0.25 (s, 3H). **Compound 2:** 7.60-7.53 (m, 2H), 7.34-7.30 (m, 3H), 6.87-6.81 (m, 2H), 6.68-6.61 (m, 1H), 4.75 (d, *J* = 10.0 Hz, 1H), 3.76 (s, 3H), 2.55-2.52 (m, 1H), 2.01 (s, 3H), 1.94-1.90 (m, 1H), 1.29-0.92 (m, 4H), 1.20 (s, 12H), 0.84-0.74 (m, 3H), 0.70-0.64 (m, 3H), 0.34 (s, 6H).**^{13}C NMR ($CDCl_3$, 100 MHz):** 169.1, 169.1, 156.5, 138.1, 138.1, 134.4, 134.4, 134.3, 134.1, 130.8, 130.7, 128.9, 128.1, 127.7, 127.6, 126.0, 125.9, 113.5, 113.4, 111.2, 111.2, 83.7, 83.7, 55.2, 54.8, 54.0, 36.3, 36.2, 26.0, 24.9, 24.9, 24.8, 23.8, 23.5, 23.3, 23.3, 15.9, 14.6, 11.1, 11.0, -3.54, -3.58, -5.50, -5.25. The carbon directly attached to the boron atom was not detected, likely due to quadrupolar relaxation. **^{11}B NMR ($CDCl_3$, 160 MHz):** 34.0. $C_{30}H_{44}BNO_4Si$. **HRMS (pos. ESI) *m/z*:** calcd for $[M+Na]^+$ 544.3030, found 544.3033.

((2*S*,3*R*,4*S*)-2-butyl-3-(dimethyl(phenyl)silyl)-6-methoxy-4-(4,4,5,5-tetramethyl-1,3,2-dioxaborolan-2-yl)-3,4-dihydroquinolin-1(2H)-yl)(*p*-tolyl)methanone (racemic mixture of (2*S*,3*R*,4*S*) and (2*R*,3*S*,4*R*)) (3ah)

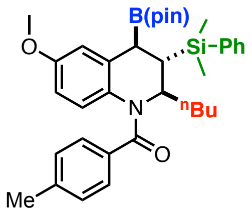

Using the **Typical Procedure A** (4-methylbenzoyl chloride was used instead of AcCl at 80 ºC), the titled compound (0.056 g, 0.094 mmol, 38% isolated yield, 67% NMR yield) was obtained as a colorless oil. **^{1}H NMR ($CDCl_3$, 400 MHz):** 7.64-7.56 (m, 2H), 7.32-7.31 (m, 3H), 7.13-7.05 (m, 2H), 6,96 (d, *J* = 7.7 Hz, 2H), 6.83 (d, *J* = 1.9 Hz, 1H), 6.38-6.22 (m, 2H), 5.18-5.05 (m, 1H), 3.71 (s, 3H), 2.58 (d, *J* = 7.0 Hz, 1H), 2.29 (s. 3H), 1.63 (d, *J* = 6.6 Hz, 1H), 1.28 (s, 6H), 1.25 (s, 6H), 1.20-0.99 (m, 6H), 0.73 (t, *J* = 6.71 Hz, 3H), 0.37 (s, 6H). **^{13}C NMR ($CDCl_3$, 100 MHz):** 168.4, 156.5, 139.8, 137.6, 134.9, 134.4, 133.5, 132.1, 129.1, 129.0, 128.3, 127.8, 127.5, 112.7, 111.0, 83.9, 55.2, 51.3, 35.8, 29.0, 28.1, 25.4, 25.1, 22.3, 21.4, 14.0, -3.97, -4.66. The carbon directly attached to the boron atom was not detected, likely due to quadrupolar relaxation. **^{11}B NMR ($CDCl_3$, 160 MHz):** 32.5. $C_{36}H_{48}BNO_4Si$. **HRMS (pos. ESI) *m/z*:** calcd for $[M+Na]^+$ 620.3343 found 620.3342.

((2*S*,3*R*,4*S*)-2-butyl-3-(dimethyl(phenyl)silyl)-6-methoxy-4-(4,4,5,5-tetramethyl-1,3,2-dioxaborolan-2-yl)-3,4-dihydroquinolin-1(2*H*)-yl)(4-nitrophenyl)methanone (racemic mixture of (2*S*,3*R*,4*S*) and (2*R*,3*S*,4*R*)) (3ai)

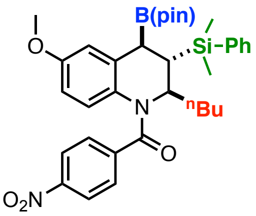

Using the **Typical Procedure A** (4-nitrobenzoyl chloride was used instead of AcCl at 80 ºC), the titled compound (0.090 g, 0.143 mmol, 58% isolated yield, 70% NMR yield) was obtained as a yellow oil. **^{1}H NMR ($CDCl_3$, 400 MHz):** 7.27 (d, *J* = 8.8 Hz, 2H), 7.59-7.57 (m, 2H), 7.28-7.27 (m, 3H), 7.16 (d, *J* = 8.7 Hz, 2H), 6.86 (dd, *J* = 2.7, 1.0 Hz, 1H), 6.28 (dd, J = 8.7, 2.4 Hz, 1H), 6.12 (d, *J* = 8.7 Hz, 1H), 5.15-5.12 (m, 1H), 3.70 (s, 3H), 2.63 (d, *J* = 7.0 Hz, 1H), 1.63 (dd. *J* = 7.1, 1.5 Hz, 1H), 1.31 (s, 6H), 1.28 (s, 6H), 1.24-1.05 (m, 6H), 0.75 (t, *J* = 7.04 Hz, 3H), 0.42 (s, 6H), 0.39 (s. 6H). **^{13}C NMR ($CDCl_3$, 100 MHz):** 166.0, 157.2, 148.0, 142.6, 137.0, 135.3, 134.5, 130.5, 129.8, 129.3, 127.9, 127.4, 122.9, 113.5, 111.0, 84.1, 55.2, 51.7, 35.7, 29.0, 28.1, 25.4, 25.1, 22.3, 14.0, -3.95, -4.31. The carbon directly attached to the boron atom was not detected, likely due to quadrupolar relaxation. **^{11}B NMR ($CDCl_3$, 160 MHz):** 33.8. $C_{35}H_{48}BN_2O_6Si$. **HRMS (pos. ESI) *m/z*:** calcd for $[M+Na]^+$ 651.3038 found 651.3059.

(2*S*,3*R*,4*S*)-2-butyl-3-(dimethyl(phenyl)silyl)-6-methoxy-1-methyl-4-(4,4,5,5-tetramethyl-1,3,2-dioxaborolan-2-yl)-1,2,3,4-tetrahydroquinoline (racemic mixture of (2*S*,3*R*,4*S*) and (2*R*,3*S*,4*R*)) (3ak)

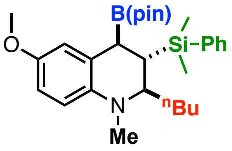

Using the **Typical Procedure A** (iodomethane was used instead of AcCl at 80 ºC), the titled compound was obtained as a crude mixture but could not be isolated due to the instability. **^{1}H NMR ($CDCl_3$, 400 MHz):** 7.62-7.59 (m, 3H), 7.53-7.49 (m, 2H), 6.66 (dd, *J* = 2.9, 0.9 Hz, 1H), 6.60 (dq, *J* = 8.8, 3.0, 0.5 Hz, 1H), 6.30 (d, *J* = 8.8 Hz, 1H), 3.73 (s, 3H), 3.03-2.99 (m, 1H), 2.73 (s, 3H), 2.50-2.48 (m, 1H), 1.78-1.77 (m, 1H), 1.23-1.22 (s, 6H),

1.20 (s, 6H), 1.20 (s, 6H), 0.83 (t, J = 7.2 Hz, 3H), 0.12 (s, 6H), 0.091 (s, 6H). $C_{29}H_{44}BNO_3Si$. **HRMS (pos. ESI) *m/z*:** calcd for $[M+Na]^+$ 516.3076 found 516.3027.

Dearomative carbo-diboration of quinolines (Fig. 2b)

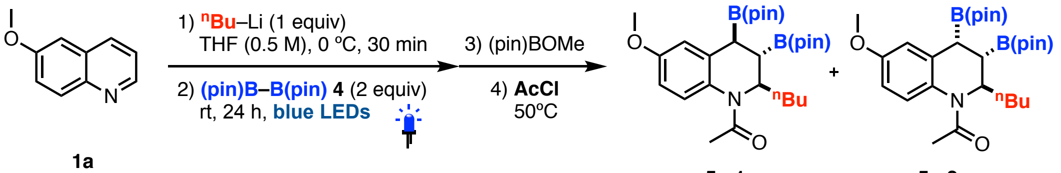

1a **5a-1** **5a-2**

6-Methoxyquinoline **1a** (0.25 mmol) was charged in a dried Schlenk tube, and dissolved in dry THF (0.5 mL, 0.5 M). To the mixture was added ^{n}BuLi (0.10 mL, 0.25 mmol; 2.55 M in hexane solution) at 0 °C, and the solution was stirred for 30 min. To the mixture was successively added $B_2(pin)_2$ (**4**) (0.127 g, 0.50 mmol) at 0 °C. After the reaction tube was sealed and irradiated for 24 hours with Blue LEDs equipped with a cooling fan at room temperature. To the mixture was successively added (pin)BOMe (0.05 mL) and acetyl chloride (0.1 mL) at room temperature and the mixture was stirred at 50 °C for 30 min. The reaction was quenched by H_2O (2 mL), and extracted with AcOEt (10 mL × 3). The AcOEt layers were combined and dried over $MgSO_4$, and the solvent was removed under reduced pressure. The residue was purified by silica gel column chromatography using hezane/AcOEt and preparative recycling gel permeation chromatography using $CHCl_3$ to give **5a** (**5a-1**; 0.0201 g, 0.039 mmol, 16% isolated yield, **5a-2**; 0.0032 g, 0.0062 mmol, 2.5% isolated yield, 61% and dr = 85:15, determined by ^{1}H NMR) as a white solid.

1-((2*S*,3*R*,4*R*)-2-butyl-6-methoxy-3,4-bis(4,4,5,5-tetramethyl-1,3,2-dioxaborolan-2-yl)-3,4-dihydroquinolin-1(2*H*)-yl)ethan-1-one (racemic mixture of (2*S*,3*R*,4*R*) and (2*R*,3*S*,4*S*)) (5a-1)
^{1}H NMR ($CDCl_3$, 500 MHz): 6.93 (d, J = 8.5 Hz, 1H), 6.90 (d, J = 2.2 Hz, 1H), 6.71 (dd, J = 8.5, 2.2 Hz, 1H), 4.92-4.88 (m, 1H), 3.79 (s, 3H), 2.16 (d, J = 12.3 Hz, 1H), 1.97 (s, 3H), 1.59 (s 1H), 1.35 (s, 6H), 1.33 (s, 6H), 1.26 (s, 12H), 1.22-1.08 (m, 6H), 0.82 (t, J = 7.0 Hz, 3H). **^{13}C NMR ($CDCl_3$, 125 MHz):** 169.8, 157.5, 139.8, 132.3, 127.0, 112.5, 110.9, 83.8, 83.5, 55.3, 53.0, 36.2, 28.1, 25.4, 24.9, 24.8, 24.7, 22.8, 22.6, 14.0. The carbon directly attached to the boron atom was not detected, likely due to quadrupolar relaxation. **^{11}B NMR ($CDCl_3$, 160 MHz):** 33.9. $C_{28}H_{45}B_2NO_6$. **HRMS (pos. ESI) *m/z*:** calcd for $[M+Na]^+$ 536.3331, found 536.3317. Relative configuration was speculated by X-ray structural analysis.

1-((2*S*,3*R*,4*S*)-2-butyl-6-methoxy-3,4-bis(4,4,5,5-tetramethyl-1,3,2-dioxaborolan-2-yl)-3,4-dihydroquinolin-1(2*H*)-yl)ethan-1-one (racemic mixture of (2*S*,3*R*,4*S*) and (2*R*,3*S*,4*R*)) (5a-2)
^{1}H NMR ($CDCl_3$, 500 MHz): 6.92 (d, J = 8.4 Hz ,1H), 6.69-6.65 (m, 2H), 4.92 (dt, J = 11.0, 5.7 Hz,1H), 3.78 (s, 3H), 2.43 (d, J = 4.1, 1H), 2.02 (s, 3H), 1.59 (m 1H), 1.40-1.33 (m, 2H), 1.28 (s, 6H), 1.27 (s, 6H), 1.24-1.22 (m, 2H), 1.16 (s, 6H), 1.15 (s, 6H), 1.12-1.10 (m, 2H), 0.82 (t, J = 6.7 Hz, 3H). **^{13}C NMR ($CDCl_3$, 125 MHz):** 170.0, 157.4, 143.2, 131.2, 127.2, 111.5, 83.8, 83.4, 55.3, 54.3, 36.3, 27.7, 25.4, 25.1, 24.7, 24.4, 23.8, 23.0, 14.0. The carbon directly attached to the boron atom was not detected, likely due to quadrupolar relaxation. **^{11}B NMR ($CDCl_3$, 160 MHz):** 33.5. $C_{28}H_{45}B_2NO_6$. **HRMS (pos. ESI) *m/z*:** calcd for $[M+Na]^+$ 536.3331, found 536.3314.

2.2. Silaborated tetrahydroquinolines as synthetic platform and its asymmetric synthesis (Fig. 3)

Potassium ((2*S*,3*R*,4*S*)-1-acetyl-2-butyl-3-(dimethyl(phenyl)silyl)-6-methoxy-1,2,3,4-tetrahydroquinolin-4-yl)trifluoroborate (racemic mixture of (2*S*,3*R*,4*S*) and (2*R*,3*S*,4*R*)) (6)

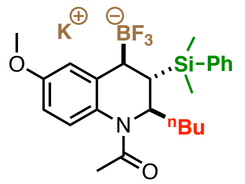

The racemic mixture of 1-(2-butyl-3-(dimethyl(phenyl)silyl)-6-methoxy-4-(4,4,5,5-tetramethyl-1,3,2-dioxaborolan-2-yl)-3,4-dihydroquinolin-1(2*H*)-yl)ethan-1-one **3a** (0.052 g, 0.10 mmol) was added methanol (2 mL) and acetonitrile (0.4 mL), followed by KF (23.2 mg, 0.40 mmol) in H_2O (0.1 mL) at room temperature. The mixture was stirred until boronic ester **3a** was fully dissolved (ca. 1 min). Then, L-(+)-tartaric acid (30.8 mg, 0.20 mmol) was dissolved into THF (0.3 mL), the solution was added to the rapidly stirring biphasic mixture (~ 1000 rpm) over a period of 5 min, and a white precipitate was formed. After being stirred for 2 min, the mixture was diluted with acetonitrile (1 mL). After being stirred for further 2 min, the mixture was diluted with acetonitrile (1 mL) and the white precipitate was filtered. The filtrate was rinsed with further portions of acetonitrile (5 mL × 3) and dried in vacuo to give **6** (0.0029 g, 0.058 mmol, 58% isolated yield) as a white solid. **mp** 240 ºC decomposition. **^{1}H NMR** and **^{13}C NMR** could not be measured due to the low solubility. **HRMS (neg. ESI) *m/z*:** calcd for $[M-K]^-$ 462.2248, found 462.2250. **ATR-FTIR (neat) ν:** 3312, 1576, 1395, 1261, 1208, 1131, 1064, 903, 841, 788, 670, 617, 572, 521, 477, 424, 411 cm^{-1}.

1-((2*S*,3*R*,4*S*)-2-butyl-3-hydroxy-6-methoxy-3,4-dihydroquinolin-1(2*H*)-yl-4-*d*)ethan-1-one (racemic mixture of (2*S*,3*R*,4*S*) and (2*R*,3*S*,4*R*)) (7-D)

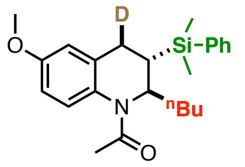

The racemic mixture of 1-(2-butyl-3-(dimethyl(phenyl)silyl)-6-methoxy-4-(4,4,5,5-tetramethyl-1,3,2-dioxaborolan-2-yl)-3,4-dihydroquinolin-1(2*H*)-yl)ethan-1-one **3a** (0.0103 g, 0.020 mmol) was charged in a dried Schlenk tube, and dissolved in dry dioxane (0.5 mL). To the mixture was added NaOMe (3.2 mg, 0.060 mmol) and D_2O (0.1 mL) at room temperature, and the solution was stirred for 17 hours at 95 ºC. The reaction was quenched by H_2O (2 mL) and extracted with AcOEt (10 mL × 3). The AcOEt layers were combined and dried over $MgSO_4$, and the solvent was removed under reduced pressure. The residue was purified by a preparative TLC (n–hexane/AcOEt = 2:1) to give **7-D** (0.0038 g, 0.0096 mmol, 48% isolated yield, 88% NMR yield) as a white solid. **mp** 80.6-82.9 ºC. **^{1}H NMR ($CDCl_3$, 400 MHz):** 7.60-7.51 (m, 2H), 7.42-7.34 (m, 3H), 6.96 (d, *J* = 8.5 Hz, 1H), 6.73 (dd, *J* = 2.7, 8.4 Hz, 1H), 6.67 (d, *J* = 2.6 Hz, 1H), 5.08-4.99 (m, 1H), 3.79 (s, 3H), 2.18 (dd, *J* = 13.8, 4.6 Hz, 1H), 1.98 (s, 3H), 1.22-0.85 (m, 1H), 1.22-0.85 (m, 6H), 0.74 (t, *J* = 6.9 Hz, 3H), 0.40 (s, 3H), 0.39 (s, 3H). **^{13}C NMR ($CDCl_3$, 100 MHz):** 169.7, 157.9, 139.8, 137.4, 133.9, 131.6, 129.2, 127.9, 127.2, 111.9, 111.5, 55.4, 51.9, 37.2, 32.3, 32.2, 28.1, 22.8, 22.4, 14.0, -4.52, -5.22. $C_{24}H_{32}DNO_2Si$. **HRMS (pos. ESI) *m/z*:** calcd for $[M+Na]^+$ 419.2241, found 419.2246.

1-((2*S*,3*R*)-2-butyl-3-(dimethyl(phenyl)silyl)-6-methoxy-3,4-dihydroquinolin-1(2H)-yl)ethan-1-one (racemic mixture of (2*S*,3*R*) and (2*R*,3*S*)) (7-H)

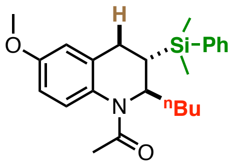

The racemic mixture of 1-(2-butyl-3-(dimethyl(phenyl)silyl)-6-methoxy-4-(4,4,5,5-tetramethyl-1,3,2-dioxaborolan-2-yl)-3,4-dihydroquinolin-1(2*H*)-yl)ethan-1-one **3a** (0.0258 g, 0.05 mmol) was charged in a dried Schlenk tube, and dissolved in dry dioxane (0.5 mL). To the mixture was added KOMe (13.0 mg, 0.18 mmol) at room temperature, and the solution was stirred for 17 hours at 50 ºC. The reaction was quenched by NH_4Cl (2 mL) and extracted with AcOEt (10 mL × 3). The AcOEt layers were combined and dried over $MgSO_4$, and the solvent was removed under reduced pressure. The residue was purified by a preparative TLC (n–hexane/AcOEt = 2:1) to give **7-H** (0.015 g, 0.038 mmol, 76% isolated yield, 82% NMR yield) as a white solid. **mp** 61.1-62.2 ºC. **^{1}H NMR ($CDCl_3$, 400 MHz):** 7.60-7.51 (m, 2H), 7.42-7.34 (m, 3H), 6.96 (d, *J* = 8.5 Hz, 1H), 6.73 (dd, *J* = 2.7, 8.4 Hz, 1H), 6.67 (d, *J* = 2.6 Hz, 1H), 5.08-4.99 (m, 1H), 3.79 (s,

3H), 2.35 (dd, J = 14.1, 4.6 Hz, 1H), 2.19 (td, J = 13.7, 4.6 Hz, 1H), 1.98 (s, 3H), 1.22-0.85 (m, 1H), 1.22-0.85 (m, 6H), 0.74 (t, J = 6.9 Hz, 3H), 0.40 (s, 3H), 0.39 (s, 3H). **^{13}C NMR ($CDCl_3$, 100 MHz):** 169.7, 157.9, 139.8, 137.4, 133.9, 131.6, 129.2, 127.9, 127.2, 111.9, 111.5, 55.4, 51.9, 37.2, 32.3, 32.2, 28.1, 22.8, 22.4, 14.0, -4.52, -5.22. $C_{24}H_{33}NO_2Si$. **HRMS (pos. ESI) *m/z*:** calcd for $[M+Na]^+$ 418.2178, found 418.2185.

1-((2*S*,3*S*,4*S*)-2-butyl-3-(dimethyl(phenyl)silyl)-4-hydroxy-6-methoxy-3,4-dihydroquinolin-1(2H)-yl)ethan-1-one (racemic mixture of (2*S*,3*S*,4*S*) and (2*R*,3*R*,4*R*)) (8)

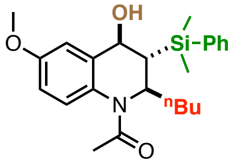

The racemic mixture of 1-(2-butyl-3-(dimethyl(phenyl)silyl)-6-methoxy-4-(4,4,5,5-tetramethyl-1,3,2-dioxaborolan-2-yl)-3,4-dihydroquinolin-1(2*H*)-yl)ethan-1-one **3a** (0.0522 g, 0.10 mmol) was charged in a dried Schlenk tube, and dissolved in THF (0.5 mL). To the mixture was added NaOH (8.9 mg, 0.060 mmol) and aq. H_2O_2 (0.33 mL, 0.06 mmol; 0.3 M) at 0 °C, and the solution was stirred for 30 minutes at room temperature. The reaction was quenched by H_2O (2 mL) and extracted with AcOEt (10 mL × 3). The AcOEt layers were combined and dried over $MgSO_4$, and the solvent was removed under reduced pressure. The residue was purified by a preparative TLC (n–hexane/AcOEt = 2:1) to give **8** (0.029 g, 0.071 mmol, 71% isolated yield, 73% NMR yield) as a white solid. **mp** 113.7–115.5 °C. **^{1}H NMR ($CDCl_3$, 400 MHz):** 7.67-7.58 (m, 2H), 7.43-7.34 (m, 3H), 7.00 (d, J = 2.8 Hz, 1H), 6.95 (d, J = 8.5 Hz, 1H), 6.77 (dd, J = 2.8, 8.5 Hz, 1H), 5.09-5.00 (m, 1H), 4.38 (dd, J = 6.1, 10.8 Hz, 1H), 3.80 (s, 3H), 2.00 (s, 3H), 1.27-0.90 (m, 1H), 1.27-0.90 (m, 6H), 0.78 (t, J = 7.0 Hz, 3H), 0.51 (s, 3H), 0.47 (s, 3H). **^{13}C NMR ($CDCl_3$, 100 MHz):** 169.3, 158.3, 141.2, 137.5, 134.1, 129.6, 128.8, 128.2, 126.8, 112.5, 107.7, 68.1, 55.5, 50.1, 41.3, 37.4, 28.0, 22.6, 22.3, 14.0, -3.65, -4.48. $C_{24}H_{33}NO_3Si$. **HRMS (pos. ESI) *m/z*:** calcd for $[M+Na]^+$ 434.2127, found 434.2122.

1-((2*S*,3*S*,4*S*)-2-butyl-3-(dimethyl(phenyl)silyl)-4,6-dimethoxy-3,4-dihydroquinolin-1(2*H*)-yl)ethan-1-one (racemic mixture of (2*S*,3*S*,4*S*) and (2*R*,3*R*,4*R*)) (9)

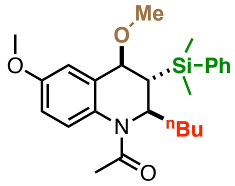

The racemic mixture of 1-(2-butyl-3-(dimethyl(phenyl)silyl)-4-hydroxy-6-methoxy-3,4-dihydroquinolin-1(2H)-yl)ethan-1-one **8** (0.0206 g, 0.050 mmol) was charged in a dried Schlenk tube, and dissolved in dry THF (1.7 mL). To the mixture was added NaH (1.7 mg, 0.070 mmol) and MeI (0.25 mL) at 0 °C, and the solution was kept at 0 ℃ for 1 hour. The solution was then allowed to warm to room temperature and stirred for 6 h. The reaction was quenched by H_2O (2 mL) and extracted with AcOEt (10 mL × 3). The AcOEt layers were combined and dried over $MgSO_4$, and the solvent was removed under reduced pressure. The residue was purified by a preparative TLC (n–hexane/AcOEt = 2:1) to give **9** (0.0117 g, 0.028 mmol, 55% isolated yield, 67% NMR yield) as a colorless oil. **^{1}H NMR ($CDCl_3$, 400 MHz):** 7.66-7.56 (m, 2H), 7.41-7.33 (m, 3H), 7.00 (d, J = 8.5 Hz, 1H), 6.89 (d, J = 2.5 Hz, 1H), 6.77 (dd, J = 2.8, 8.5 Hz, 1H), 5.10-4.98 (m, 1H), 3.83 (s, 3H), 3.74 (d, J = 11.1 Hz, 1H), 3.35 (s, 3H), 2.02 (s, 3H), 1.11-0.77 (m, 1H), 1.11-0.77 (m, 6H), 0.70 (t, J = 7.0 Hz, 3H), 0.46 (s, 3H), 0.42 (s, 3H). **^{13}C NMR ($CDCl_3$, 100 MHz):** 169.2, 158.2, 139.7, 137.7, 134.2, 129.5, 129.1, 127.8, 127.1, 112.1, 108.2, 78.0, 58.8, 55.5, 50.6, 39.3, 36.8, 28.1, 22.7, 22.3, 14.0, -2.99, -4.02. $C_{25}H_{35}NO_3Si$. **HRMS (pos. ESI) *m/z*:** calcd for $[M+Na]^+$ 448.2284, found 448.2262.

1-((2*S*,3*S*,4*S*)-4-(benzyloxy)-2-butyl-3-(dimethyl(phenyl)silyl)-6-methoxy-3,4-dihydroquinolin-1(2H)-yl)ethan-1-one (racemic mixture of (2*S*,3*S*,4*S*) and (2*R*,3*R*,4*R*)) (10)

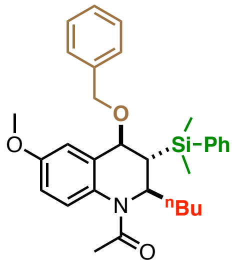

The racemic mixture of 1-(2-butyl-3-(dimethyl(phenyl)silyl)-4-hydroxy-6-methoxy-3,4-dihydroquinolin-1(2H)-yl)ethan-1-one **8** (0.0206 g, 0.050 mmol) was charged in a dried Schlenk tube, and dissolved in dry THF (1.7 mL). To the mixture was added NaH (1.7 mg, 0.070 mmol) and BnBr (0.25 mL) at 0 °C, and the solution was kept at 0 ℃ for 1 hour. The solution was then allowed to warm to room temperature and stirred for 6 h. The reaction was quenched by H_2O (2 mL) and extracted with AcOEt (10 mL × 3). The AcOEt layers were combined and dried over $MgSO_4$, and the solvent was

removed under reduced pressure. The residue was purified by a preparative TLC (n–hexane/AcOEt = 2:1) to give **10** (0.0148 g, 0.030 mmol, 59% isolated yield, 65% NMR yield) as a colorless oil. **^{1}H NMR ($CDCl_3$, 400 MHz):** 7.60-7.58 (m, 2H), 7.37-7.28 (m, 8H), 6.97 (d, *J* = 8.54, 1H), 6.87 (dd, *J* = 2.6, 1H), 6.77 (dd, *J* = 8.5, 2.8 Hz, 1H). 5.12 (m, 1H), 4.53 (d, *J* = 11.3 Hz, 1H), 4.32 (d, *J* = 11.3 Hz, 1H), 4.05 (d, *J* = 10.9 Hz, 1H), 3.74 (s, 3H), 2.05 (s, 3H), 1.14-0.85 (m, 1H), 1.14-0.85 (m, 6H), 0.71 (t, *J* = 6.87, 3H), 0.44 (s, 3H), 0.40 (s, 3H). **^{13}C NMR ($CDCl_3$, 100 MHz):** 169.3, 158.1, 139.4, 137.7, 134.2, 129.5, 129.4, 129.1, 128.4, 128.3, 127.9, 127.9, 127.7, 127.6, 127.4, 127.0, 112.4, 108.6, 76.1, 72.6, 55.4, 50.7, 38.9, 36.7, 28.1, 22.8, 22.3, 14.0, -3.00, -3.73. $C_{31}H_{39}NO_3Si$. **HRMS (pos. ESI) *m/z*:** calcd for $[M+Na]^+$ 524.2591, found 524.2617.

1-((2*S*,3*S*,4*S*)-2-butyl-3-(dimethyl(phenyl)silyl)-6-methoxy-4-methyl-3,4-dihydroquinolin-1(2*H*)-yl)ethan-1-one (racemic mixture of (2*S*,3*S*,4*S*) and (2*R*,3*R*,4*R*)) (11)

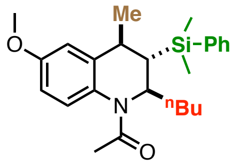

The racemic mixture of 1-(2-butyl-3-(dimethyl(phenyl)silyl)-6-methoxy-4-(4,4,5,5-tetramethyl-1,3,2-dioxaborolan-2-yl)-3,4-dihydroquinolin-1(2*H*)-yl)ethan-1-one **3a** (0.0522 g, 0.10 mmol) was charged in a dried Schlenk tube, and dissolved in dry dioxane (0.5 mL). To the mixture was added NaOMe (16.2 mg, 0.30 mmol) and MeI (0.5 mL) at room temperature, and the solution was stirred for 24 hours at 95 ºC. The reaction was quenched by H_2O (2 mL) and extracted with AcOEt (10 mL × 3). The AcOEt layers were combined and dried over $MgSO_4$, and the solvent was removed under reduced pressure. The residue was purified by a preparative TLC (n–hexane/AcOEt = 2:1) to give **11** (0.0387 g, mixture with **5-H**, purity 38 wt%, 0.031 mmol, 31% isolated yield) as a colorless oil. **^{1}H NMR ($CDCl_3$, 400 MHz):** 7.59-7.52 (m, 2H), 7.38-7.35 (m, 3H), 6.96-6.93 (m, 1H), 6.75-6.71 (m, 2H), 5.10-5.00 (m, 1H), 3.81 (s, 3H), 2.40-2.33 (m, 1H), 1.99 (s, 3H), 1.16 (d, *J* = 6.8 Hz, 3H), 1.17-0.86 (m, 6H), 0.76-0.71 (m, 3H), 0.62 (dd, *J* = 11.4, 5.9 Hz, 1H), 0.45 (s, 3H), 0.41 (s, 3H). **^{13}C NMR ($CDCl_3$, 100 MHz):** 169.6, 158.2, 143.3, 133.9, 133.7, 131.3, 129.0, 127.8, 126.7, 110.4, 110.2, 51.9, 40.2, 39.2, 37.1, 32.1, 28.2, 22.6, 22.3, 17.4, 14.0, -3.22, -3.62. $C_{25}H_{35}NO_2Si$. **HRMS (pos. ESI) *m/z*:** calcd for $[M+Na]^+$ 432.2235, found 432.2322.

1-((2*S*,3*R*,4*S*)-2-butyl-3-hydroxy-6-methoxy-4-methyl-3,4-dihydroquinolin-1(2H)-yl)ethan-1-one (racemic mixture of (2*S*,3*R*,4*S*) and (2*R*,3*S*,4*R*)) (13)

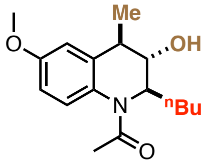

The racemic mixture of 1-(2-butyl-3-(dimethyl(phenyl)silyl)-6-methoxy-4-methyl-3,4-dihydroquinolin-1(2*H*)-yl)ethan-1-one **11** (0.0342 g, purity 38 wt% including **7-H**, 0.031 mmol) was charged in a dried Schlenk tube, and dissolved in dry CH_2Cl_2 (3.0 mL). To the mixture was added $HBF_4 Et_2O$ (1.9 mL, 1.2 mmol) at 0 °C, and then allowed to warm to room temperature and stirred for 5 h. The reaction was quenched by $NaHCO_3$ (2 mL) and extracted with AcOEt (10 mL × 3). The AcOEt layers were combined and dried over $MgSO_4$, and the solvent was removed under reduced pressure to give crude product of racemic mixture of 1-(2-butyl-3-(fluorodimethylsilyl)-6-methoxy-4-methyl-3,4-dihydroquinolin-1(2*H*)-yl)ethan-1-one **12**. Then, the crude product **12** was charged in a dried Schlenk tube and dissolved in THF (0.2 mL) and MeOH (0.2 mL). To the mixture was added KF (20 mg, 0.33 mmol), $KHCO_3$ (83.4 mg, 0.83 mmol), and aq. H_2O_2 (0.16 mL, 0.17 mmol; 0.3 M) at 0 °C, and the solution was kept at 0 °C for 1.5 h. The solution was then allowed to warm to room temperature and stirred for 2 h. The reaction was quenched by H_2O (2 mL) and extracted with AcOEt (10 mL × 3). The AcOEt layers were combined and dried over $MgSO_4$, and the solvent was removed under reduced pressure. The residue was purified by a preparative TLC (n–hexane/AcOEt = 1:1) to give **13** (0.0088 g, 0.030 mmol, 96% isolated 2 steps yield) as a colorless oil. **^{1}H NMR ($CDCl_3$, 400 MHz):** 7.01 (d, *J* = 8.5 Hz, 1H), 6.84-6.80 (m, 1H), 6.80-6.73 (m, 1H), 4.69-4.66 (m, 1H), 3.84 (s, 3H), 3.11-3.04 (m, 1H), 2.57-2.46 (m, 1H), 2.06 (s, 3H), 1.42 (d, *J* = 7.0 Hz, 3H), 1.36-1.20 (m, 6H), 0.84 (t, *J* = 6.9 Hz, 3H). **^{13}C NMR ($CDCl_3$, 100 MHz):** 170.0, 158.3, 137.8, 131.4, 126.4, 111.4, 111.1, 80.9, 60.1, 55.5, 38.6, 33.9, 27.8, 22.6, 22.2, 14.0, 13.1. $C_{17}H_{25}NO_3$. **HRMS (pos. ESI) *m/z*:** calcd for $[M+Na]^+$ 314.1732, found 314.1704.

1-((2*R*,3*S*,4*R*)-2-butyl-3-(dimethyl(phenyl)silyl)-6-methoxy-4-(4,4,5,5-tetramethyl-1,3,2-dioxaborolan-2-yl)-3,4-dihydroquinolin-1(2*H*)-yl)ethan-1-one ((+)-3aa)

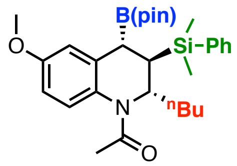

6-Methoxyquinoline **1a** (0.10 mmol) and (4*S*,4'*S*)-2,2'-(pentane-3,3-diyl)bis(4-benzyl-4,5-dihydrooxazole) (chiral ligand, 0.020 g, 0.05 mmol) were charged in a dried Schlenk tube and dissolved in dry toluene (0.05 mL, 0.2 M). To the mixture was added ^{n}BuLi (0.10 mL, 0.25 mmol; 2.55 M in hexane solution) at –90 ºC, and the solution was stirred for 2 hours. To the mixture was successively added (dimethylphenylsilyl)boronic acid pinacol ester [PhMe$_2$Si–B(pin)] (**2a**) (0.078 g, 0.30 mmol) at –90 ºC. After the reaction tube was sealed and irradiated for 24 hours with Blue LEDs equipped with a cooling fan at room temperature. To the mixture was successively added acetyl chloride (0.1 mL) at room temperature and the mixture was stirred at 50°C for 30 min. The reaction was quenched by H_2O (2 mL), and extracted with AcOEt (10 mL × 3). The AcOEt layers were combined and dried over $MgSO_4$, and the solvent was removed under reduced pressure. The residue was purified by a preparative TLC (n–hexane/AcOEt/toluene/dichloromathane = 3:1:3:3) and preparative recycling gel permeation chromatography using $CHCl_3$ to give **(+)-3aa** (0.014 g, 0.026 mmol, 26% isolated yield, 64% NMR yield) as a white solid.

[α]$^{25}_{D}$ +59.4° (c 0.0032, $CHCl_3$, 92% ee). **^{1}H NMR (CDCl$_3$, 400 MHz):** 7.56 (dd, *J* = 6.4, 3.0 Hz, 2H), 7.32-7.31 (m, 3H), 6.85-6.83 (m, 2H), 6.66 (dd, *J* = 2.5, 8.6 Hz, 1H), 5.12-5.09 (m, 1H), 3.77 (s, 3H), 2.45 (d, *J* = 5.5 Hz, 1H), 2.00 (s, 3H), 1.54 (dd, *J* = 5.5, 2.0 Hz, 1H), 1.22 (s, 6H), 1.21 (s, 6H), 1.17-1.04 (m, 6H), 0.74 (t, *J* = 6.9 Hz, 3H), 0.35 (s, 3H), 0.28 (s, 3H). Other Analyses were performed by using racemic **3aa** shown in Page S-3.

1-((2*S*,3*S*,4*S*)-2-butyl-3-(dimethyl(phenyl)silyl)-4-hydroxy-6-methoxy-3,4-dihydroquinolin-1(2H)-yl)ethan-1-one (racemic mixture of (2*S*,3*S*,4*S*) and (2*R*,3*R*,4*R*)) ((+)-8)

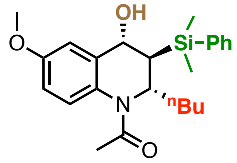

6-Methoxyquinoline **1a** (0.10 mmol) and (4*S*,4'*S*)-2,2'-(pentane-3,3-diyl)bis(4-benzyl-4,5-dihydrooxazole) (chiral ligand, 0.047 g, 0.12 mmol) were charged in a dried Schlenk tube and dissolved in dry toluene (0.30 mL, 0.3 M). To the mixture was added ^{n}BuLi (0.10 mL, 0.25 mmol; 2.55 M in hexane solution) at –90 ºC, and the solution was stirred for 2 hours. To the mixture was successively added (dimethylphenylsilyl)boronic acid pinacol ester [PhMe$_2$Si–B(pin)] (**2a**) (0.078 g, 0.30 mmol) at –90 ºC. After the reaction tube was sealed and irradiated for 24 hours with Blue LEDs equipped with a cooling fan at room temperature. To the mixture was successively added acetyl chloride (0.1 mL) at room temperature and the mixture was stirred at 50°C for 30 min. The reaction was quenched by H_2O (2 mL), and extracted with AcOEt (10 mL × 3). The AcOEt layers were combined and dried over $MgSO_4$, and the solvent was removed under reduced pressure. The residue was roughly purified by a preparative TLC to give **(+)-3aa** (0.0084 g, 0.016 mmol, 16% isolated yield, 39% NMR yield).

The given **(+)-3aa** (0.0084 g, 0.016 mmol) was charged in a dried Schlenk tube, and dissolved in THF (0.5 mL). To the mixture was added NaOH (1.4 mg, 0.001 mmol) and aq. H_2O_2 (0.053 mL, 0.001 mmol; 0.3 M) at 0 ºC, and the solution was stirred for 30 minutes at room temperature. The reaction was quenched by H_2O (2 mL) and extracted with AcOEt (10 mL × 3). The AcOEt layers were combined and dried over $MgSO_4$, and the solvent was removed under reduced pressure. The residue was purified by a preparative TLC (n–hexane/AcOEt = 2:1) to give **(+)-8** (0.0054 g, 0.011 mmol, 71% isolated yield, 88% NMR yield) as a white solid.

[α]$^{25}_{D}$ +103.3° (c 0.0027, $CHCl_3$, 99% ee). **^{1}H NMR (CDCl$_3$, 400 MHz):** 7.67-7.58 (m, 2H), 7.43-7.34 (m, 3H), 7.00 (d, *J* = 2.8 Hz, 1H), 6.95 (d, *J* = 8.5 Hz, 1H), 6.77 (dd, *J* = 2.8, 8.5 Hz, 1H), 5.09-5.00 (m, 1H), 4.38 (dd, *J* = 6.1, 10.8 Hz, 1H), 3.80 (s, 3H), 2.00 (s, 3H), 1.27-0.90 (m, 1H), 1.27-0.90 (m, 6H), 0.78 (t, *J* = 7.0 Hz, 3H), 0.51 (s, 3H), 0.47 (s, 3H). Other Analyses were performed by using racemic **8** shown in Page S-13.

2.3. Dearomative triple elementalization of anthracene and phenanthrene (Fig. 5)

Typical Procedure B:

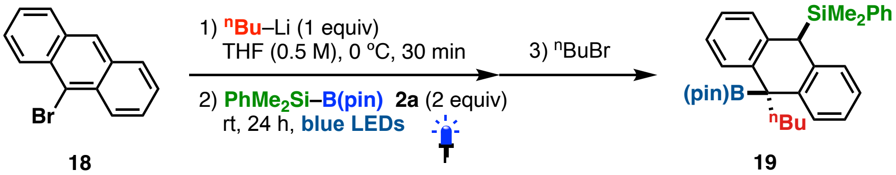

18 **19**

9-Bromoanthracene **18** (0.25 mmol) was charged in a dried Schlenk tube and dissolved in dry dioxane (0.5 mL, 0.5 M). To the mixture was added ^{n}BuLi (0.10 mL, 0.25 mmol; 2.55 M in hexane solution) at –78 °C, and the solution was stirred at 0 °C for 30 min. To the mixture was successively added (dimethylphenylsilyl)boronic acid pinacol ester [PhMe$_2$Si–B(pin)] (**2a**) (0.131 g, 0.50 mmol) at room temperature. After the reaction tube was sealed and irradiated for 24 hours with blue LEDs equipped with a cooling fan at room temperature. To the mixture was successively added n-butylbromide (0.10 mL) at room temperature and the mixture was stirred for 1 hours. The reaction was quenched by H_2O (2 mL) and extracted with AcOEt (10 mL × 3). The AcOEt layers were combined and dried over $MgSO_4$, and the solvent was removed under reduced pressure. The residue was purified by a silica gel column chromatography using hexane/AcOEt and preparative recycling gel permeation chromatography using $CHCl_3$ to give **19** (0.039 g, 0.079 mmol, 32% isolated yield, 60% NMR yield) as a yellow oil.

((9*S*,10*S*)-10-butyl-10-(4,4,5,5-tetramethyl-1,3,2-dioxaborolan-2-yl)-4a,9,9a,10-tetrahydroanthracen-9-yl)dimethyl(phenyl)silane (racemic mixture of (9*S*,10*S*) and (9*R*,10*R*)) (19)

Using the **Typical Procedure B**, the titled compound was obtained as a yellow oil. **^{1}H NMR ($CDCl_3$, 500 MHz):** 7.48-7.46 (m, 2H), 7.37-7.29 (m, 5H), 7.05 (dt, *J* = 15.0, 1.3 Hz, 2H), 6.93 (dt, *J* = 14.8, 1.3 Hz, 2H), 6.74 (d, *J* = 7.6 Hz, 2H), 4.05 (s, 1H), 2.19-2.15 (m, 2H), 1.21 (s, 12H), 1.00 (tt, *J* = 7.4, 7.4 Hz, 2H), 0.60 (t, *J* = 7.4 Hz, 3H), 0.46-0.40 (m, 2H), 0.00 (s, 6H). **^{13}C NMR ($CDCl_3$, 125 MHz):** 139.5, 137.1, 135.4, 134.2, 128.8, 127.8, 127.6, 127.5, 124.7, 124.6, 83.5, 41.3, 38.3, 30.9, 25.5, 24.5, 22.9, 13.9, -3.61. The carbon directly attached to the boron atom was not detected, likely due to quadrupolar relaxation. **^{11}B NMR ($CDCl_3$, 160 MHz):** 33.3. $C_{32}H_{41}BO_2Si$. **HRMS (pos. ESI) *m*/*z*:** calcd for $[M+Na]^+$ 519.2867, found 519.2895. **ATR-FTIR (neat) ν:** 2977, 1361, 1317, 1144, 1112, 849, 811, 774, 434, 424 cm^{-1}. The relative configuration was assigned by NOESY experiments. The NOE was observed between the benzyl proton (δ 4.04 ppm) and the butyl group protons (δ 2.19-2.15, 0.60 ppm). These results indicate that the silyl group and boryl group are *syn*-configuration.

2,2'-((9*S*,10*S*)-9-butyl-4a,9,9a,10-tetrahydroanthracene-9,10-diyl)bis(4,4,5,5-tetramethyl-1,3,2-dioxaborolane) (racemic mixture of (9*S*,10*S*) and (9*R*,10*R*)) (20-1)

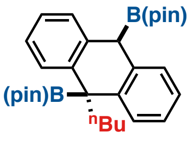

Using the **Typical Procedure B** [using $B_2(pin)_2$ **4** instead of silylborane], the titled compound was obtained as a colorless oil. **^{1}H NMR ($CDCl_3$, 500 MHz):** 7.28 (d, *J* = 7.4 Hz, 2H), 7.15-7.08 (m, 4H), 4.01 (s, 1H), 2.18 (t, *J* = 8.2 Hz, 2H), 1.30-1.24 (m, 2H) 1.17 (s, 12H), 1.15 (s, 12H), 1.08-1.03 (m, 2H), 0.64 (t, *J* = 7.3 Hz, 3H). **^{13}C NMR ($CDCl_3$, 125 MHz):** 138.1, 135.0, 128.1, 128.0, 125.5, 125.1, 83.6, 83.5, 41.0, 26.0, 24.6, 24.5, 23.1, 14.1. The carbon directly attached to the boron atom was not detected, likely due to quadrupolar relaxation. **^{11}B NMR ($CDCl_3$, 160 MHz):** 33.1. $C_{30}H_{42}B_2O_4$. **HRMS (pos. ESI) *m*/*z*:** calcd for $[M+Na]^+$ 511.3167, found 511.3164. **ATR-FTIR (neat) ν:** 2977, 2363, 1370, 1318, 1272, 1144, 970, 851, 761, 671, 472, 409 cm^{-1}. The relative configuration was assigned by NOESY experiments. The NOE was observed between the benzyl proton (δ 4.01 ppm) and the butyl group protons (δ 2.18, 0.64 ppm). These results indicate that the two boryl groups are *syn*-configuration.

2,2'-((9*R*,10*S*)-9-butyl-4a,9,9a,10-tetrahydroanthracene-9,10-diyl)bis(4,4,5,5-tetramethyl-1,3,2-dioxaborolane) (racemic mixture of (9*R*,10*S*) and (9*S*,10*R*)) (20-2)

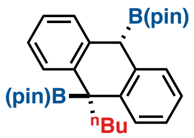

Using the **Typical Procedure B** [using $B_2(pin)_2$ **4** instead of silylborane], the titled compound was obtained as a colorless oil. **^{1}H NMR ($CDCl_3$, 500 MHz):** 7.17-7.15 (m, 4H), 7.12-7.07 (m, 4H), 4.03 (s, 1H), 2.05-2.02 (m, 2H), 1.22 (s, 12H), 1.13 (s, 12H), 1.08, (m, 2H), 0.98-0.91 (m, 2H), 0.70 (t, *J* = 7.3 Hz, 3H). **^{13}C NMR ($CDCl_3$, 125 MHz):** 138.1, 135.5, 128.4, 128.2, 125.2, 125.1, 83.5, 42.5, 27.1, 24.5, 24.4, 23.4, 14.2. The carbon directly attached to the boron atom was not detected, likely due to quadrupolar relaxation. **^{11}B NMR ($CDCl_3$, 160 MHz):** 32.9. $C_{30}H_{42}B_2O_4$. **HRMS (pos. ESI) *m*/*z*:** calcd for $[M+Na]^+$ 511.3167, found 511.3167. **ATR-FTIR (neat) ν:** 2978, 1370, 1318, 1271, 1144, 969, 851, 732, 423 cm^{-1}. The relative configuration was assigned by NOESY experiments. The NOE was not observed between the benzyl proton (δ 4.03 ppm) and the butyl group protons (δ 2.05-2.02, 0.70 ppm). These results indicate that the two boryl groups are *anti*-configuration.

2,2'-((9*S*,10*S*)-9-butyl-9,10-dihydrophenanthrene-9,10-diyl)bis(4,4,5,5-tetramethyl-1,3,2-dioxaborolane) (racemic mixture of (9*S*,10*S*) and (9*R*,10*R*)) (22)

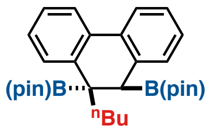

Using the **Typical Procedure B** [using $B_2(pin)_2$ **4** instead of silylborane and 9-bromophenanthrene **21** instead of **18**], the titled compound was obtained as a colorless oil. **^{1}H NMR ($CDCl_3$, 500 MHz):** 7.93-7.86 (m, 1H), 7.69-7.59 (m, 2H), 7.22-7.13 (m, 5H), 2.79 (s, 1H), 1.46-1.36 (m, 2H), 1.35 (s, 6H), 1.33 (s, 6H), 1.28-1.21 (m, 2H), 1.04-0.99 (m, 2H), 0.88 (s, 6H), 0.82 (s, 6H), 0.69 (t, *J* = 7.3 Hz, 3H). **^{13}C NMR ($CDCl_3$, 125 MHz):** 143.0, 137.9, 134.3, 134.1, 128.5, 127.1, 126.7, 125.6, 123.9, 123.2, 83.3, 82.7, 36.5, 30.9, 29.0, 25.2, 24.9, 24.6, 24.4, 23.9, 23.1, 14.0. The carbon directly attached to the boron atom was not detected, likely due to quadrupolar relaxation. **^{11}B NMR ($CDCl_3$, 160 MHz):** 33.8. $C_{30}H_{42}B_2O_4$. **HRMS (pos. ESI) *m*/*z*:** calcd for $[M+Na]^+$ 511.3211, found 511.3167. The relative configuration was assigned by NOESY experiments. The NOE was not observed between the benzyl proton (δ 2.79 ppm) and the butyl group protons (δ 1.46-1.36, 0.69 ppm). These results indicate that the two boryl groups are *anti*-configuration.

3. Computational Details

All calculations were carried with the Gaussian 16 program package.[2] The hybrid density functional method based on (U)B3LYP with a standard 6-31+g* basis set was used for geometry optimizations.[3] The solvation effect was examined by performing single-point self-consistent reaction field (SCRF) calculations[4] based on the polarizable continuum model (PCM) for gas-phase optimized structures. Excitation wavelengths and oscillator strengths were obtained at the density functional level using time-dependent perturbation theory (TD-DFT) approach. The vibrational frequencies were computed at the same level to check whether each optimized structure is an energy minimum (no imaginary frequency) or a transition state (one imaginary frequency) and to evaluate its zero-point vibrational energy. Intrinsic reaction coordinates (IRC) were calculated to confirm the connection between the transition states and the reactants/products.[5]

Supplementary Table 1. Sum of electronic and thermal free energies (*G*) and imaginary frequencies for the optimized strucutures in Fig. 6.

	G (hartree) [@6-31+g*]	*G* (hartree) [@6-31+g*&PCM(dioxane)]	imaginary frequency (cm^{-1}) [@6-31+g*]
**IM1**	-1461.640994	-1461.648023	None
**TS1_2**	-1461.607740	-1461.611311	-125.2802
**IM2**	-1461.641900	-1461.648869	None
**TS2_3**	-1461.594117	-1461.601273	-178.7448
**IM3**	-1461.637384	-1461.642723	None
**^{1}IM1***	-1461.560040	-1461.566095	None
**^{3}IM1***	-1461.560844	-1461.569981	None
**^{3}IM1_4***	-1461.556683	-1461.562902	None
**^{1}IM4***	-1461.566691	-1461.576817	None
**^{3}IM4***	-1461.571954	-1461.58272	None
**^{3}TS4_5***	-1461.550262	-1461.556643	-161.9661
**^{3}IM5***	-1461.587000	-1461.588565	None
**IM6**	-1461.619642	-1461.626354	None
**TS6_7**	-1461.605107	-1461.6128	-116.9176
**IM7**	-1461.636697	-1461.646356	None
**TS7_8**	-1461.630875	-1461.638637	-191.0117
**IM8**	-1461.648369	-1461.654645	None
**^{3}IM9***	-1461.562544	-1461.56235	None
**^{3}TS9_10***	-1461.512641	-1461.519903	-332.8678
**^{3}IM10***	-1461.561456	-1461.56358	None
**IM11**	-1525.148074	-1525.152084	None
**IM12**	-1398.124669	-1398.130168	None

Supplementary Table 2. TD-DFT vertical one-electron excitations.

Compound	excited state	Energy (eV)	Wavelength (nm)	oscillator strength (*f*)	description [Relative major contribution on the excited state is listed.]
**quinoline**	1	4.2940	288.74	0.0016	HOMO–2 → LUMO (0.70224)
	2	4.4136	280.92	0.0443	HOMO → LUMO (0.68333)
	3	4.5414	273.01	0.0214	HOMO–1 → LUMO (0.55684)
	4	5.2400	236.61	0.0000	HOMO–2 → LUMO+1 (0.70328)
	5	5.8026	213.67	0.1407	HOMO → LUMO+2 (0.51507)
**Model INT-A (INT-A')**	1	3.3913	365.60	0.0032	HOMO → LUMO (0.70570)
	2	3.5863	345.72	0.0348	HOMO–1 → LUMO (0.69520)
	3	3.9307	315.43	0.0029	HOMO → LUMO+1 (0.69650)
	4	4.1230	300.71	0.0040	HOMO–1 → LUMO+1 (0.69580)
	5	4.3624	284.21	0.0096	HOMO → LUMO+2 (0.70570)
**anthracene**	1	3.2148	385.66	0.0580	HOMO → LUMO (0.69997)
	2	3.8496	322.07	0.0002	HOMO–1 → LUMO (0.50438)
	3	4.4987	275.60	0.0000	HOMO–2 → LUMO (0.51391)
	4	4.8334	256.52	0.0000	HOMO → LUMO+2 (0.50765)
	5	4.9973	248.10	0.0000	HOMO → LUMO+3 (0.69998)
**Model INT-G (INT-G')**	1	2.3325	531.55	0.1080	HOMO → LUMO (0.69635)
	2	3.1319	395.88	0.0371	HOMO–1 → LUMO (0.69048)
	3	3.2877	377.12	0.0081	HOMO → LUMO+1 (0.70188)
	4	3.4823	356.04	0.0134	HOMO → LUMO+2 (0.65418)
	5	3.9443	314.34	0.0090	HOMO–1 → LUMO+2 (0.40591)

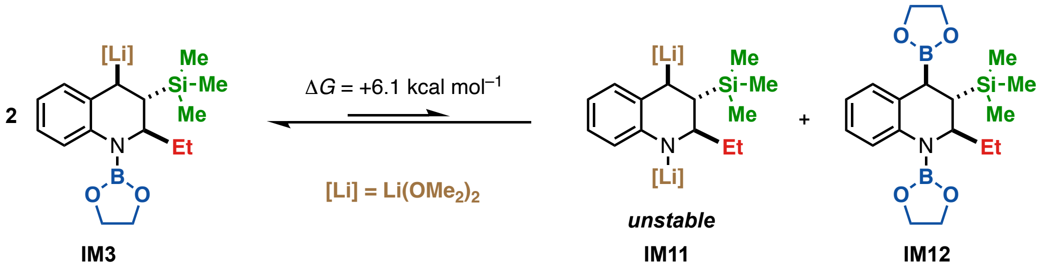

Supplementary Fig 1. Gibbs energy change upon intermolecular borylation calculated at the M06/6-31+g*+PCM (dioxane)//M06/6-31+g* level of theory

4. Single Crystal X-ray Structures

Supplementary Table 3. Crystal data and data collection parameters of **3aa** (***rac***).

CCDC	CCDC2184597
Empirical formula	$C_{30}H_{44}BNO_4Si$
Formula weight	521.56
Temperature/K	293(2)
Crystal system	monoclinic
Space group	$P2_1/n$
a/Å	18.7201(4)
b/Å	8.80841(13)
c/Å	20.2837(4)
α/°	90
β/°	117.040(2)
γ/°	90
Volume/Å^3	2979.07(11)
Z	4
ρ_{calc}/gcm^{-3}	1.163
μ/mm^{-1}	0.957
F(000)	1128.0
Crystal size/mm^3	0.386 × 0.273 × 0.174
Radiation	CuKα (λ = 1.54184)
2Θ range for data collection/°	5.334 to 148.576
Index ranges	$-23 \leq h \leq 22, -10 \leq k \leq 10, -20 \leq l \leq 25$
Reflections collected	18006
Independent reflections	5911 [R_{int} = 0.0343, R_{sigma} = 0.0285]
Data/restraints/parameters	5911/845/420
Goodness-of-fit on F^2	1.045
Final *R* indexes [I>=2σ (I)]	R_1 = 0.0450, wR_2 = 0.1211
Final *R* indexes [all data]	R_1 = 0.0478, wR_2 = 0.1236
Largest diff. peak/hole / e Å^{-3}	0.36/-0.37

Supplementary Table 4. Crystal data and data collection parameters of **3qa** (***rac***).

CCDC	CCDC2184600
Empirical formula	$C_{30}H_{44}BNO_3Si$
Formula weight	505.56
Temperature/K	293
Crystal system	triclinic

Space group	*P*-1
a/Å	10.2379(3)
b/Å	10.4920(2)
c/Å	15.8099(4)
α/°	71.191(2)
β/°	86.420(2)
γ/°	68.426(2)
Volume/Å^3	1491.89(7)
Z	2
ρ_{calc}/g cm^{-3}	1.125
μ/mm^{-1}	0.108
F(000)	548.0
Crystal size/mm^3	0.407 × 0.184 × 0.134
Radiation	MoKα (λ = 0.71073)
2Θ range for data collection/°	4.286 to 61.106
Index ranges	-14 ≤ h ≤ 14, -14 ≤ k ≤ 14, -22 ≤ l ≤ 22
Reflections collected	27549
Independent reflections	8616 [R_{int} = 0.0304, R_{sigma} = 0.0346]
Data/restraints/parameters	8616/0/334
Goodness-of-fit on F^2	1.096
Final *R* indexes [I>=2σ (I)]	R_1 = 0.0544, wR_2 = 0.1444
Final *R* indexes [all data]	R_1 = 0.0808, wR_2 = 0.1658
Largest diff. peak/hole / e Å^{-3}	0.38/-0.40

Supplementary Table 5. Crystal data and data collection parameters of **5a-1** (***rac***).

CCDC	CCDC2184598
Empirical formula	$C_{28}H_{45}B_2NO_6$
Formula weight	513.27
Temperature/K	293
Crystal system	triclinic
Space group	*P*-1
a/Å	10.6317(9)
b/Å	11.7599(9)
c/Å	13.0757(11)
α/°	109.070(7)
β/°	93.189(7)
γ/°	107.176(7)
Volume/Å^3	1455.1(2)

Z	2
ρ_{calc}/g cm^{-3}	1.171
μ/mm^{-1}	0.636
F(000)	556.0
Crystal size/mm^3	0.5 × 0.4 × 0.2
Radiation	CuKα (λ = 1.54184)
2Θ range for data collection/°	22.924 to 102.818
Index ranges	$-10 \leq h \leq 9$, $-8 \leq k \leq 11$, $-12 \leq l \leq 10$
Reflections collected	3076
Independent reflections	2540 [R_{int} = 0.0195, R_{sigma} = 0.0491]
Data/restraints/parameters	2540/0/345
Goodness-of-fit on F^2	1.098
Final R indexes [I>=2σ (I)]	R_1 = 0.0555, wR_2 = 0.1482
Final R indexes [all data]	R_1 = 0.0680, wR_2 = 0.1583
Largest diff. peak/hole / e Å^{-3}	0.22/-0.20

X-ray crystal structure analysis of 5a-1

Single clear light colorless needle-shaped crystals of **5a-1** (***rac***) were obtained by recrystallisation from heaxane/AcOEt at 0 ºC. A suitable crystal was selected and mounted on a suitable support on an XtaLAB Synergy, Single source at home/near, HyPix diffractometer. The crystal was kept at a steady T = 293 K during data collection. The structure was solved with the "What is This?" program[6a] and the ShelXT 2018/1[6b] structure solution program using the Intrinsic Phasing solution method and using Olex2[7] as the graphical interface. The model was refined with version 2018/1 of ShelXL 2018/1[5b] using Least Squares minimization.

5. HPLC, GCMS, and ESI-MS Data

a.

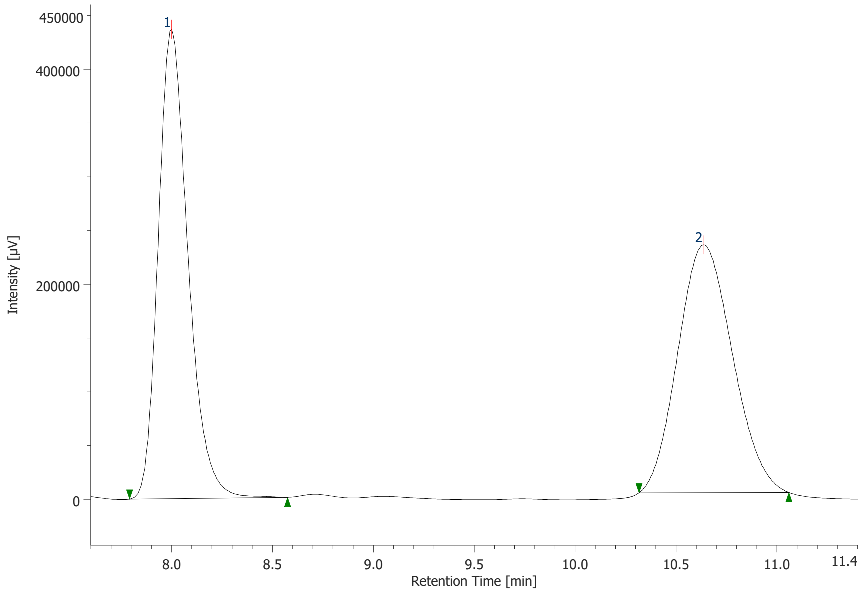

Peak No.	Retention Time (min)	Area (%)
1	8.000	49.332
2	10.633	50.668

b.

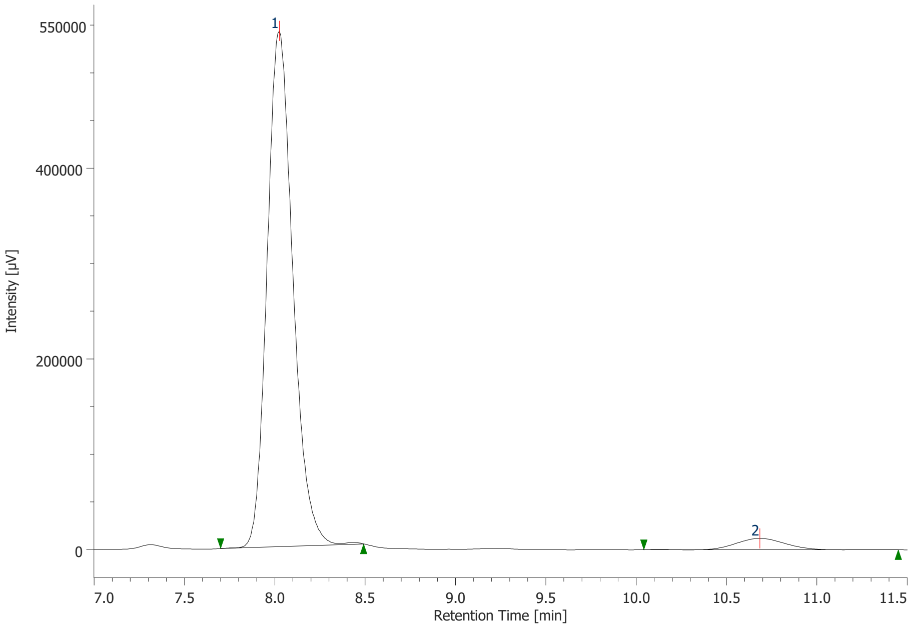

Peak No.	Retention Time (min)	Area (%)
1	8.025	95.978
2	10.683	4.022

Supplementary Fig 2. HPLC chart of 1-((2*R*,3*S*,4*R*)-2-butyl-3-(dimethyl(phenyl)silyl)-6-methoxy-4-(4,4,5,5-tetramethyl-1,3,2-dioxaborolan-2-yl)-3,4-dihydroquinolin-1(2*H*)-yl)ethan-1-one ((+)-3aa). a. HPLC chart of the racemic mixture of **3aa**. **b.** HPLC chart of **(+)-3aa.**

a.

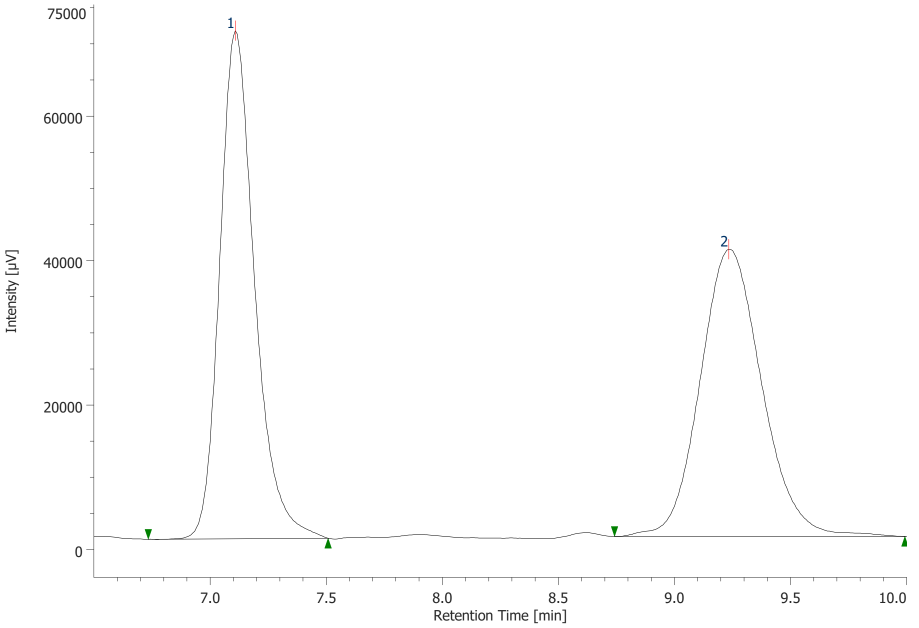

Peak No.	Retention Time (min)	Area (%)
1	7.008	50.028
2	9.233	49.974

b.

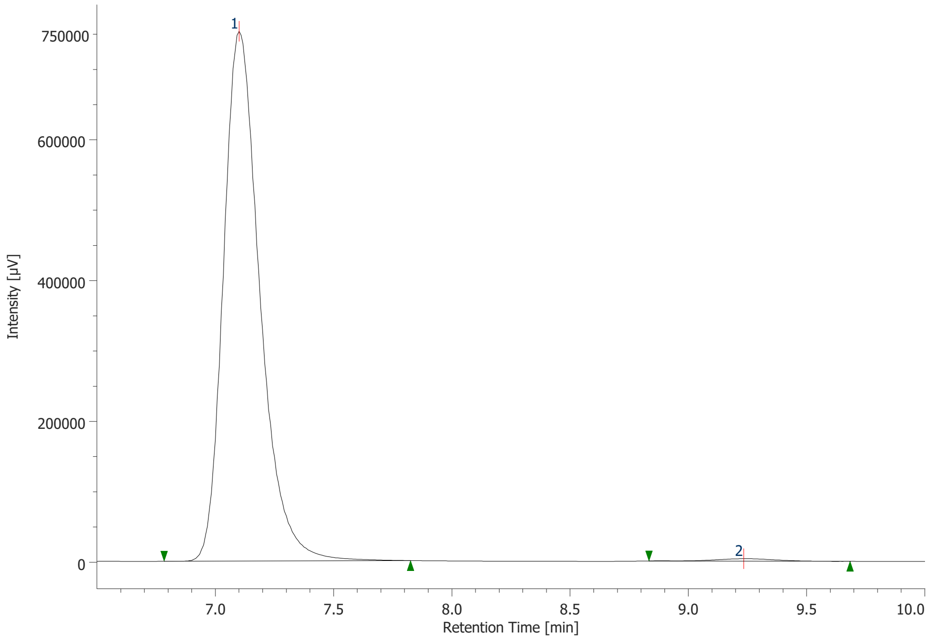

Peak No.	Retention Time (min)	Area (%)
1	7.100	99.288
2	9.233	0.712

Supplementary Fig 3. HPLC chart of 1-((2*S*,3*S*,4*S*)-2-butyl-3-(dimethyl(phenyl)silyl)-4-hydroxy-6-methoxy-3,4-dihydroquinolin-1(2*H*)-yl)ethan-1-one ((+)-8). a. HPLC chart of the racemic mixture of **8**. **b.** HPLC chart of **(+)-8.**

a.

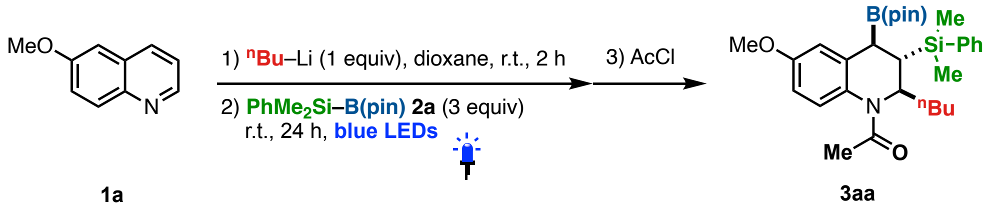

1a **3aa**

b.

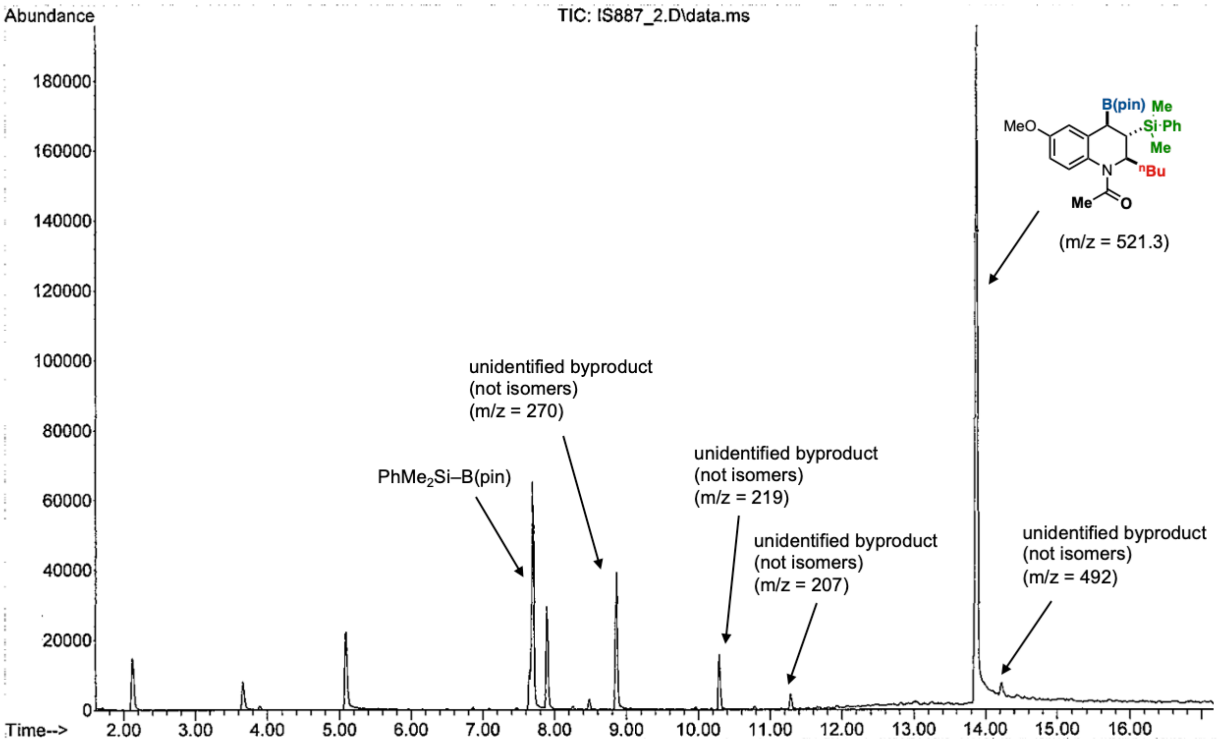

c.

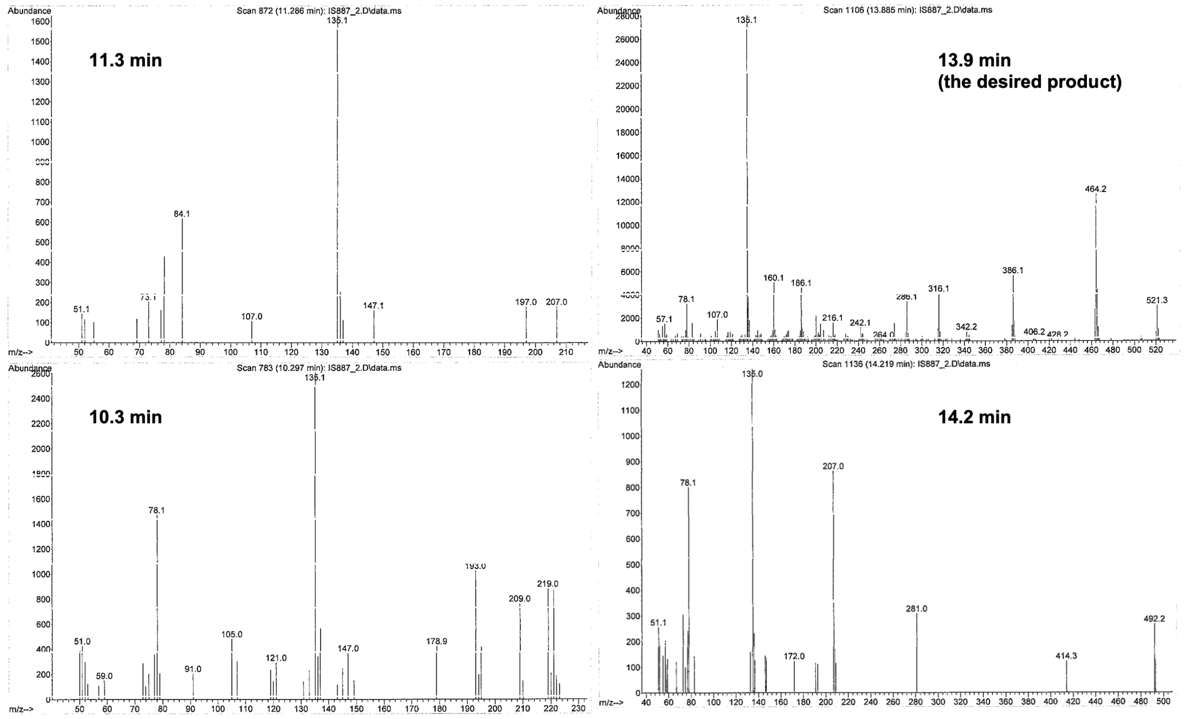

Supplementary Fig 4. GCMS spectra of the crude product of 3aa. a. Reaction scheme of synthesis of **3aa. b.** GC chart of the crude mixture of **3aa. c.** MS chart of the peaks on GC at 10.3, 11.3, 13.9, and 14.2 min.

a.

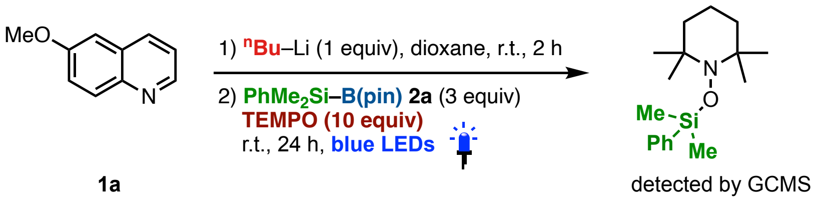

b.

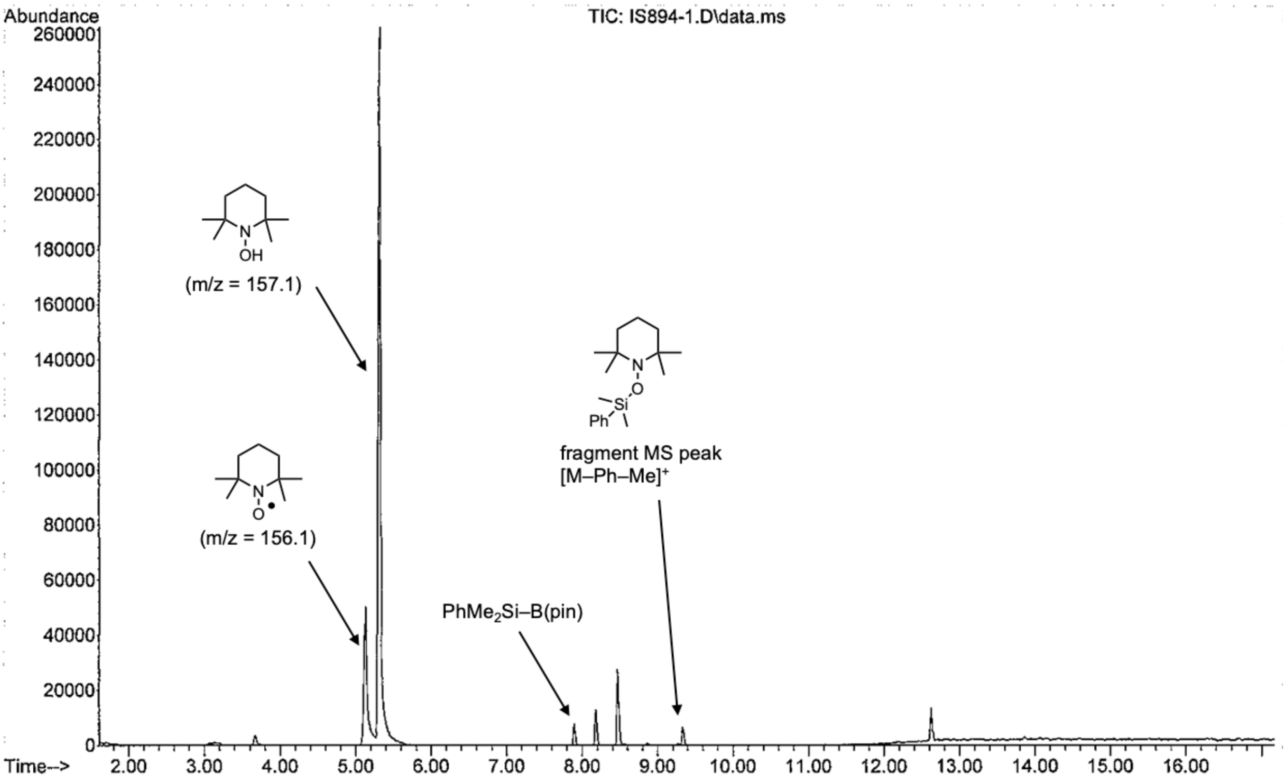

c.

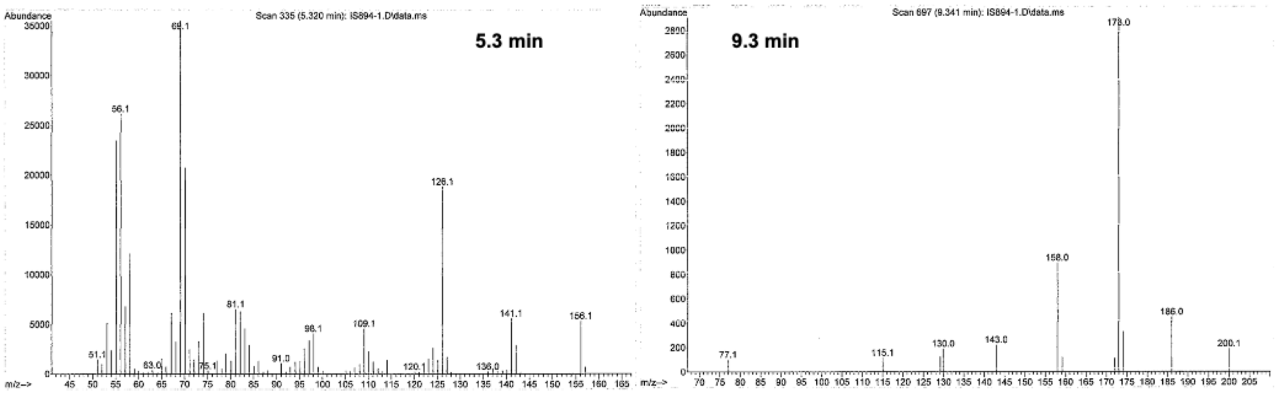

Supplementary Fig 5. GCMS spectra of the additive experiment with TEMPO. a. Reaction scheme of the additive experiment with TEMPO. **b.** GC chart of the crude mixture. **c.** MS chart of the peaks on GC at 5.3 and 9.3 min.

a.

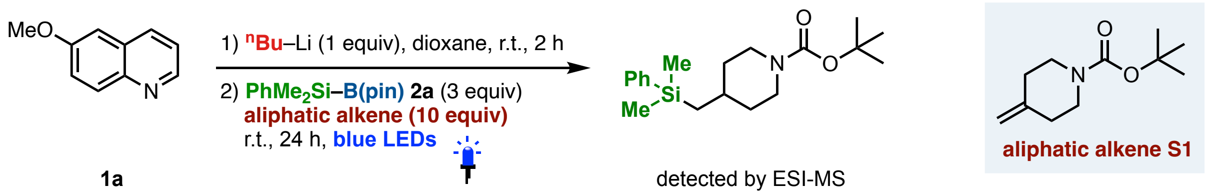

b.

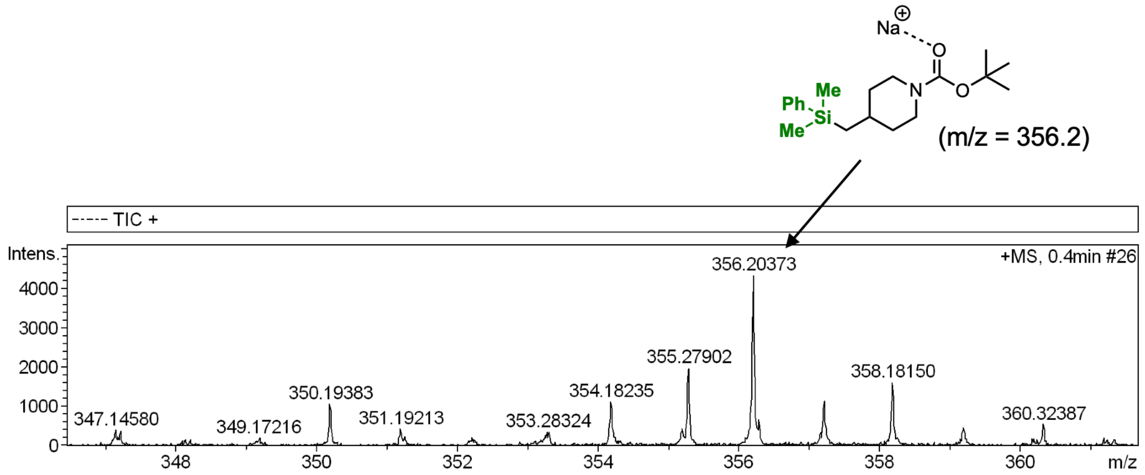

Supplementary Fig 6. ESI-MS spectra of the additive experiment with aliphatic alkene. a. Reaction scheme of the additive experiment with aliphatic alkene **S1**. **b.** ESI-MS chart of the crude mixture.

6. Copies of NMR Spectra

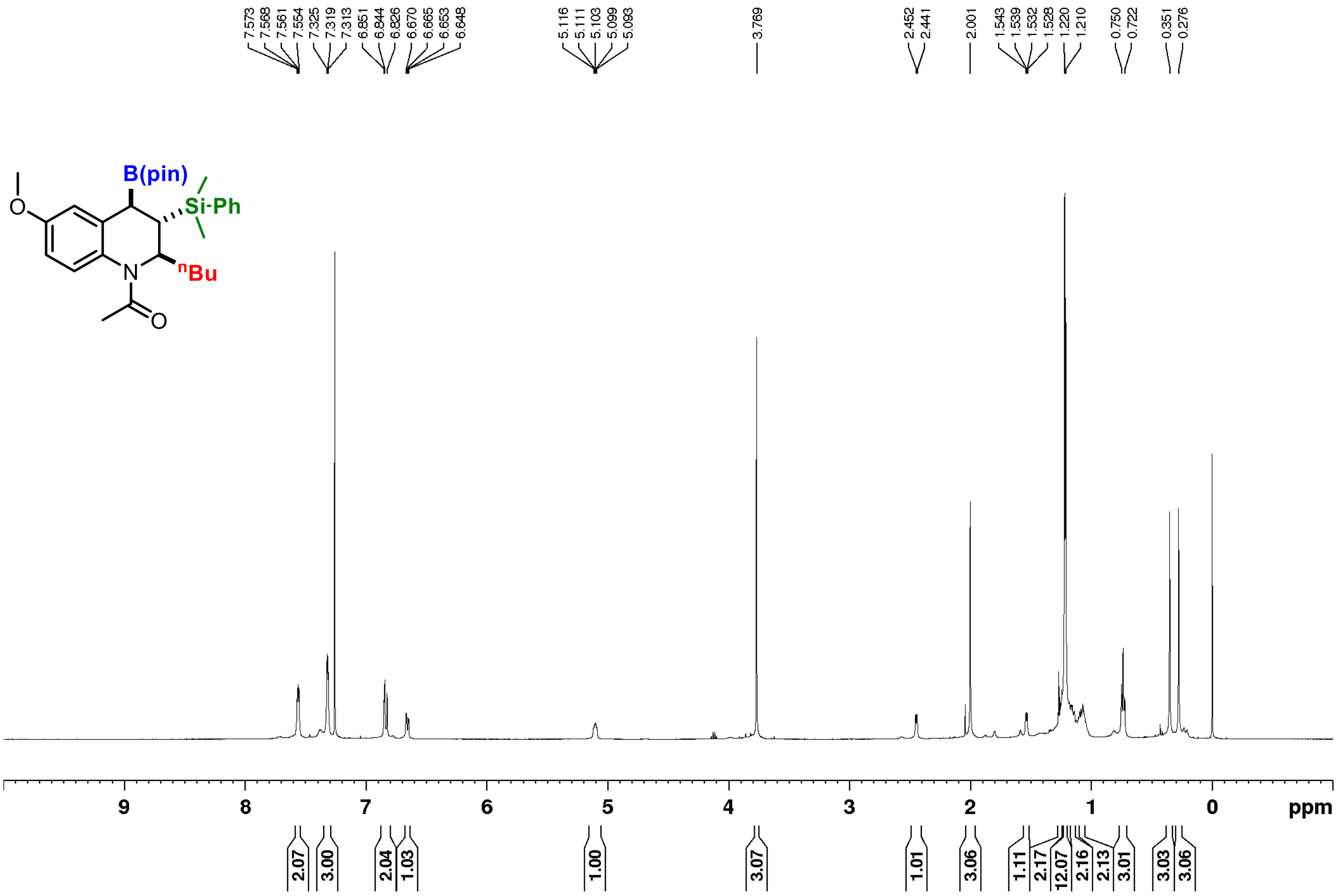

Supplementary Fig 7. ¹H NMR spectrum (500 MHz, $CDCl_3$, r.t.) of 3aa.

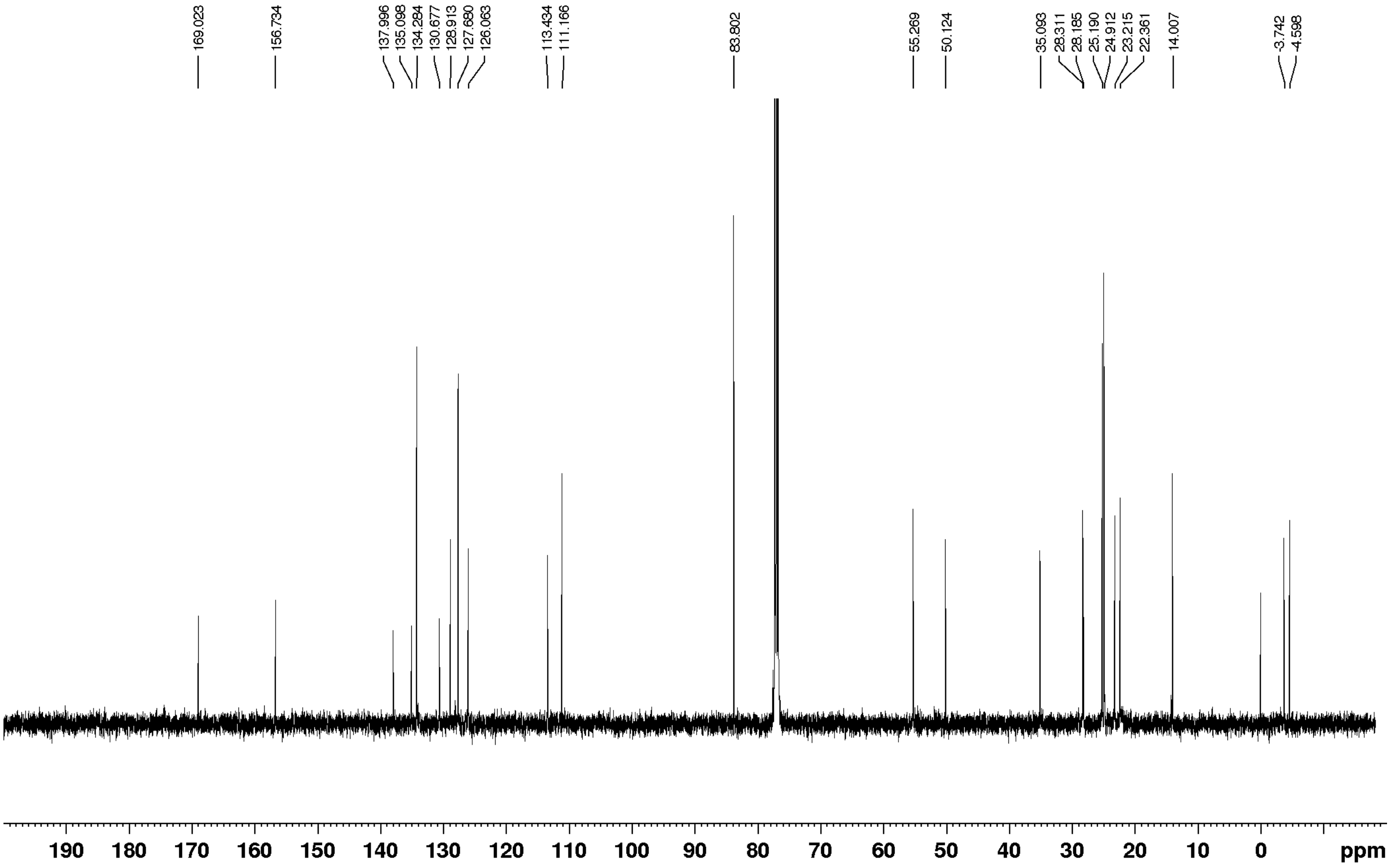

Supplementary Fig 8. ¹³C NMR spectrum (125 MHz, $CDCl_3$, r.t.) of 3aa.

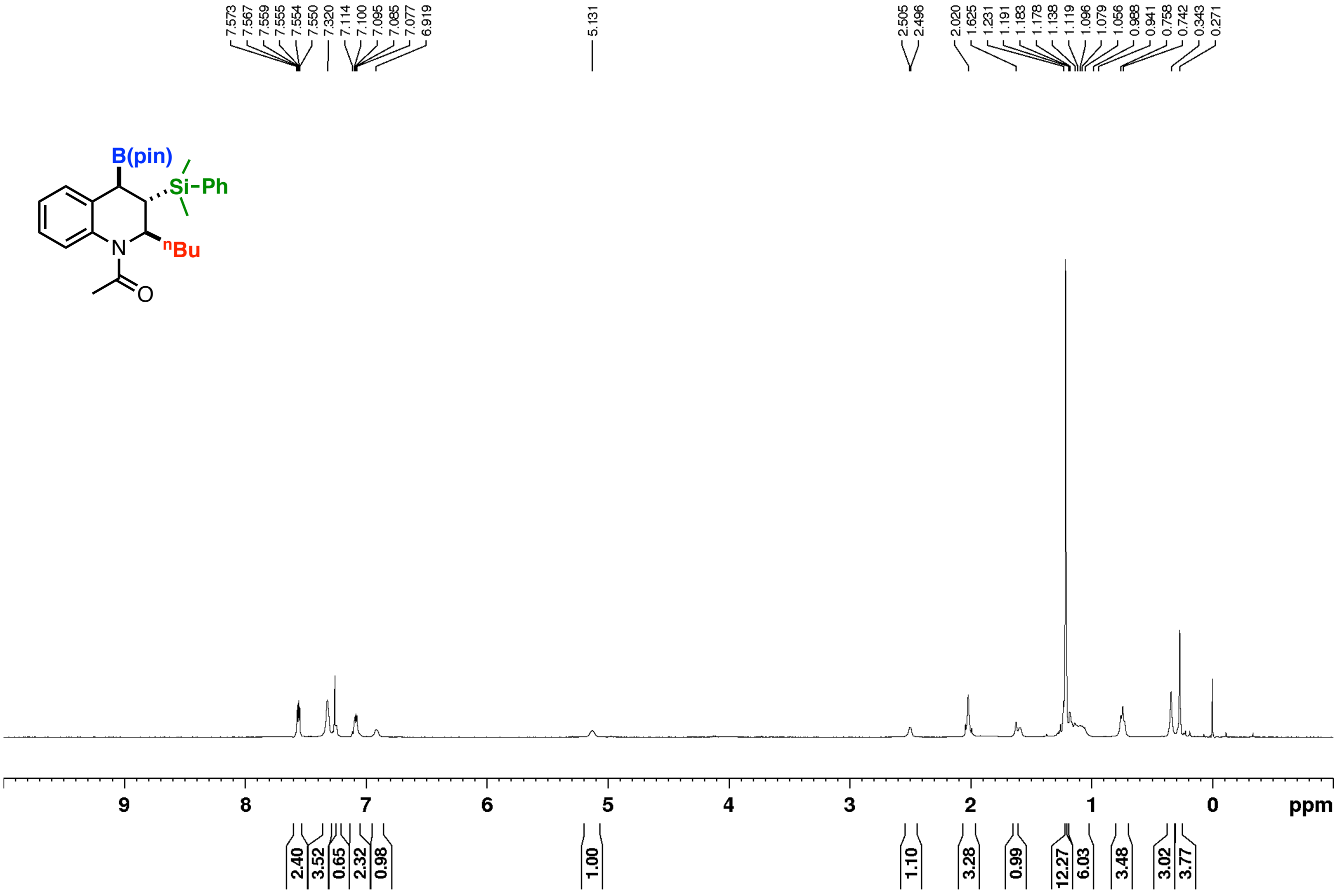

Supplementary Fig 9. ^{1}**H NMR spectrum (400 MHz, CDCl$_3$, r.t.) of 3ba**.

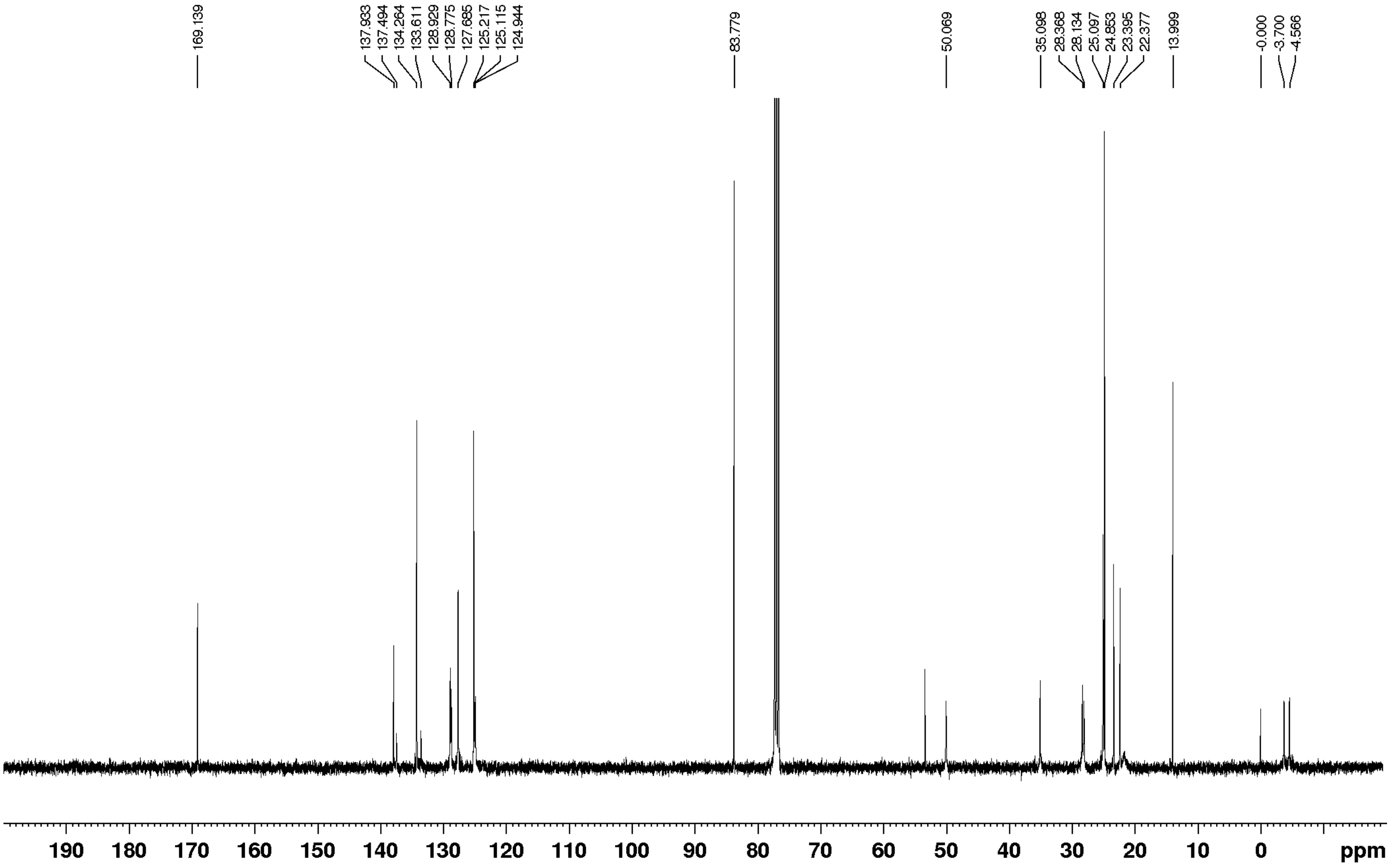

Supplementary Fig 10. ^{13}**C NMR spectrum (100 MHz, CDCl$_3$, r.t.) of 3ba**.

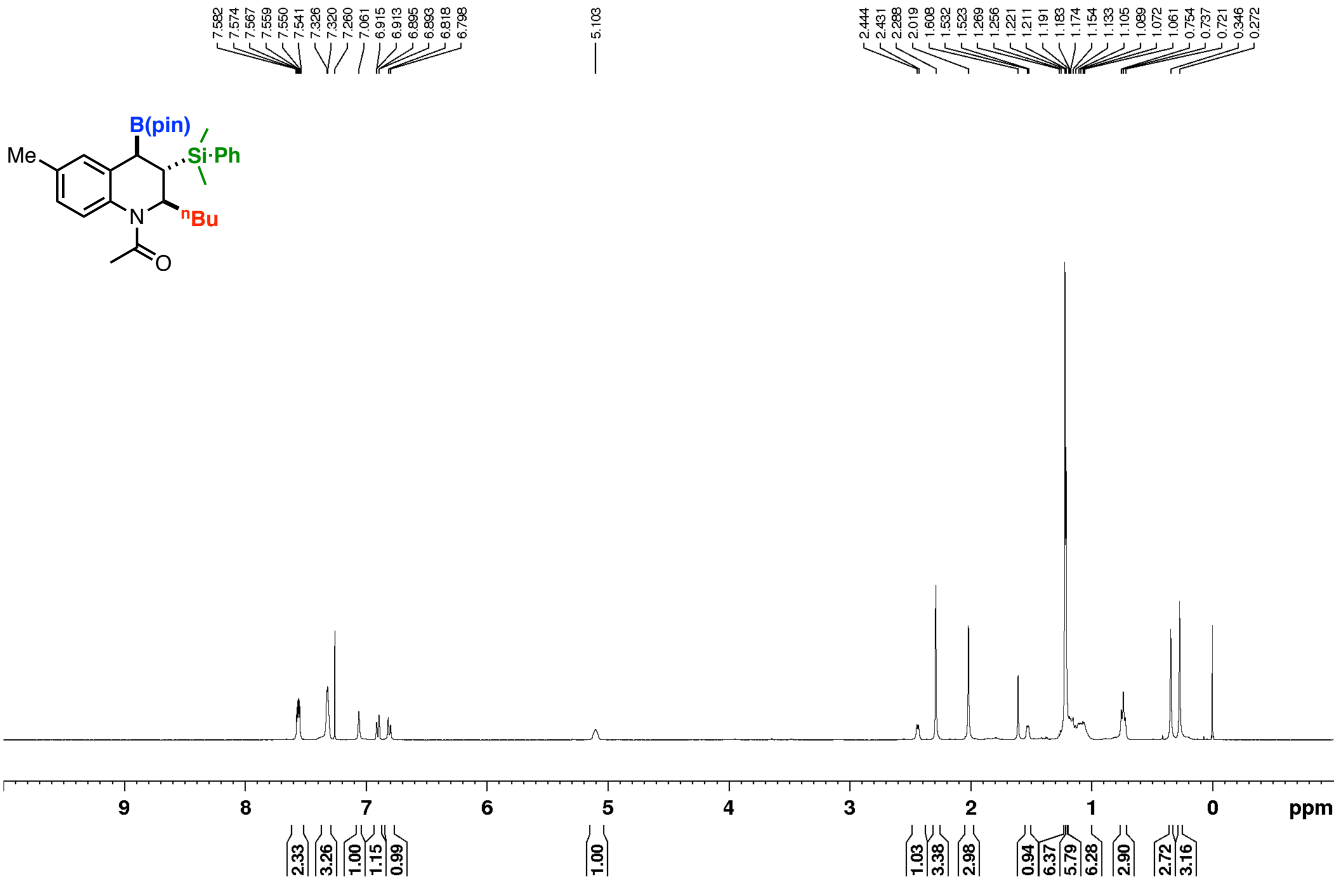

Supplementary Fig 11. **^{1}H NMR spectrum (400 MHz, $CDCl_3$, r.t.) of 3ca**.

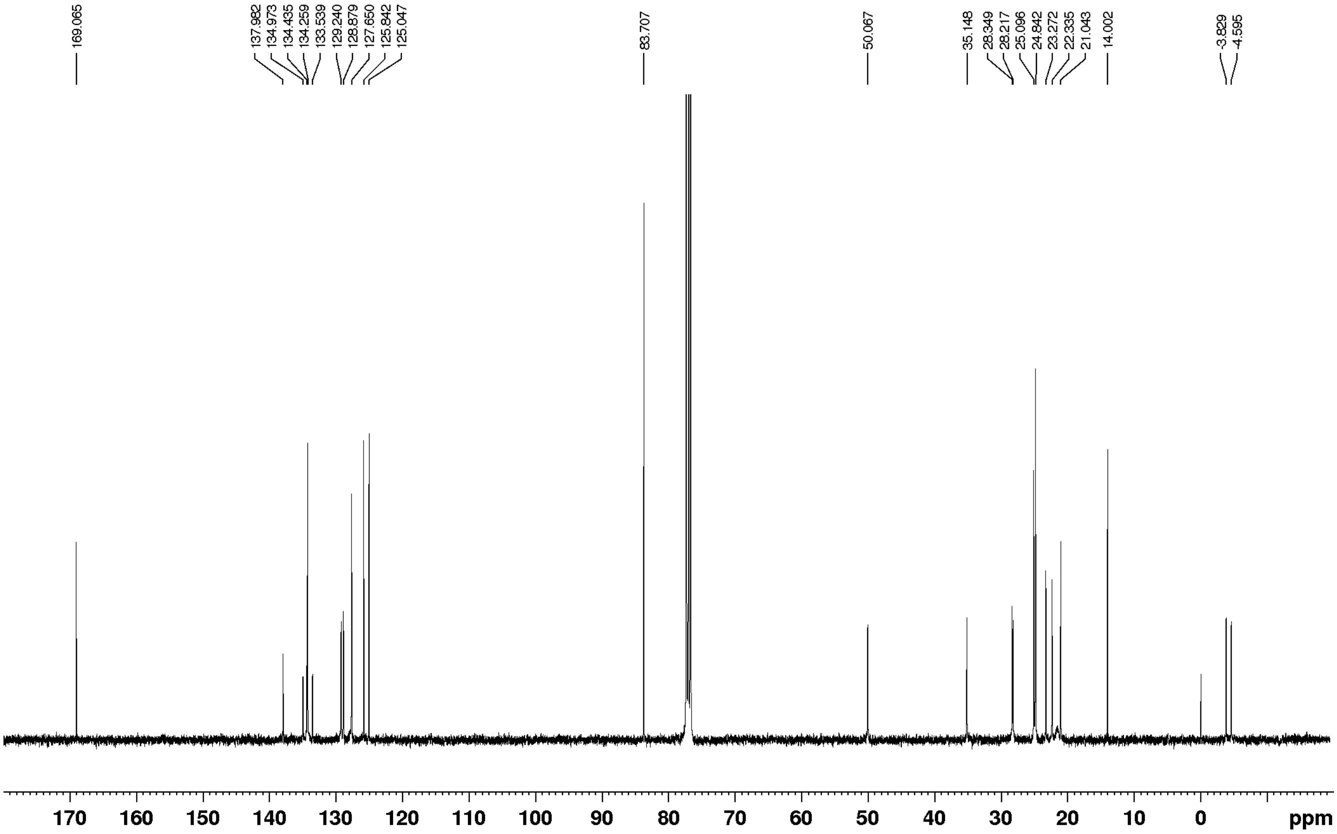

Supplementary Fig 12. **^{13}C NMR spectrum (100 MHz, $CDCl_3$, r.t.) of 3ca**.

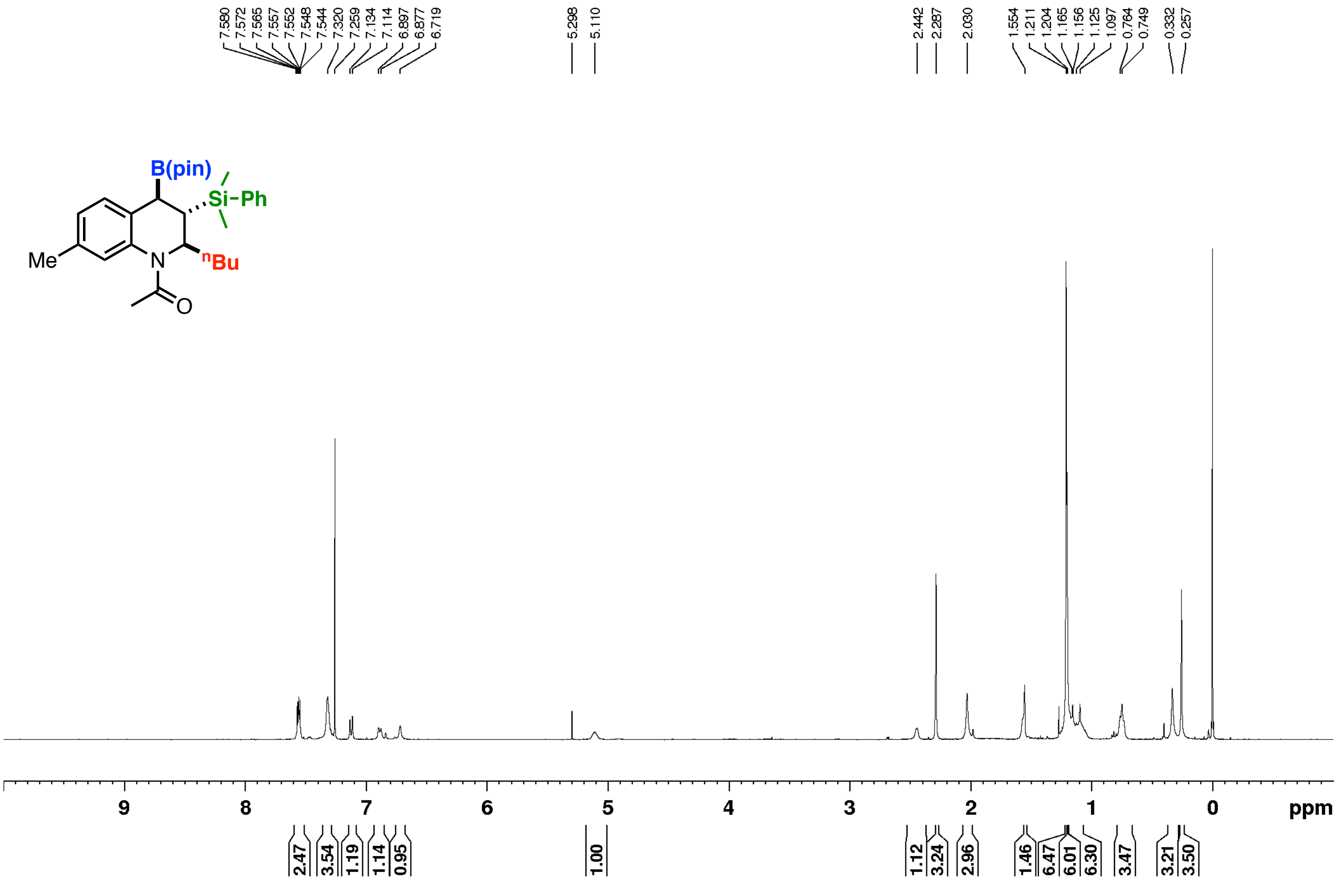

Supplementary Fig 13. ^{1}H NMR spectrum (400 MHz, $CDCl_3$, r.t.) of 3da.

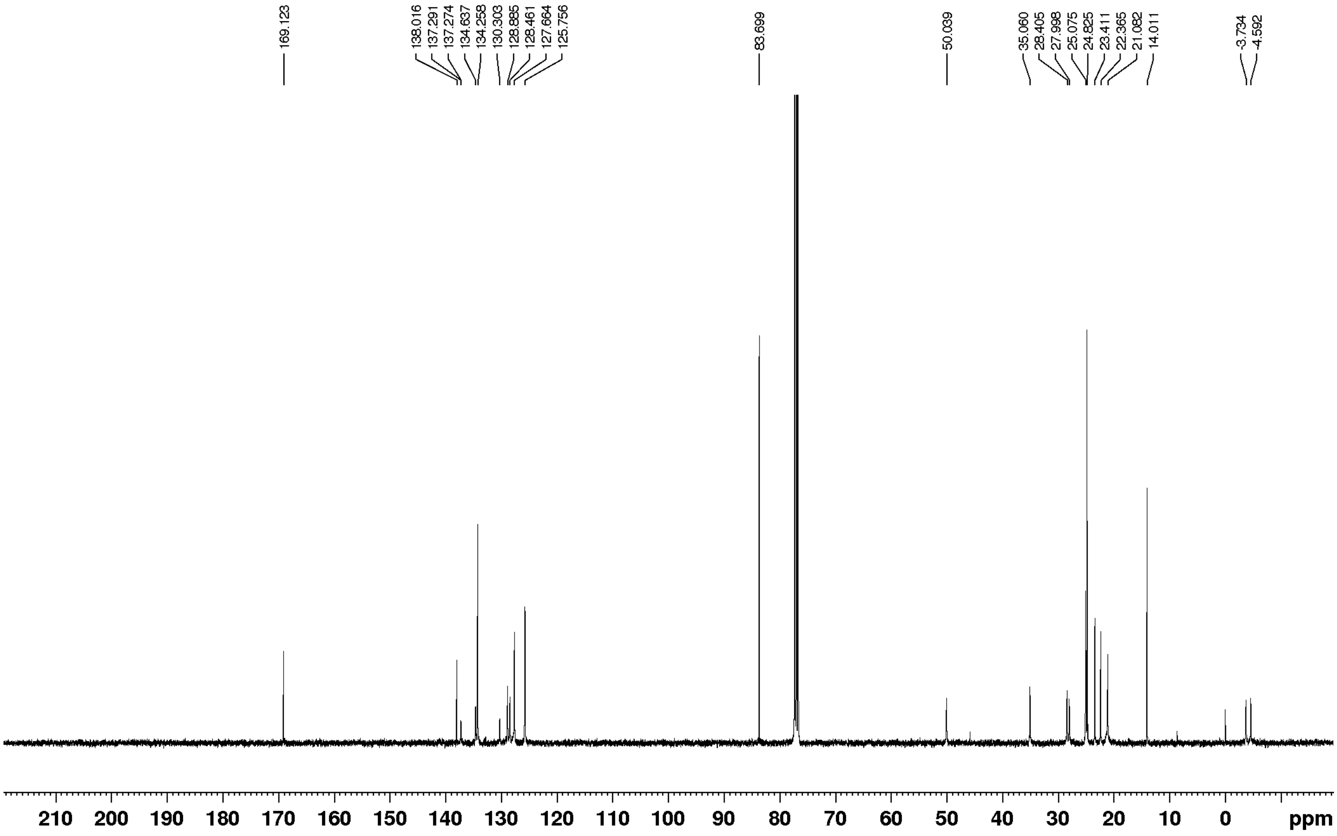

Supplementary Fig 14. ^{13}C NMR spectrum (100 MHz, $CDCl_3$, r.t.) of 3da.

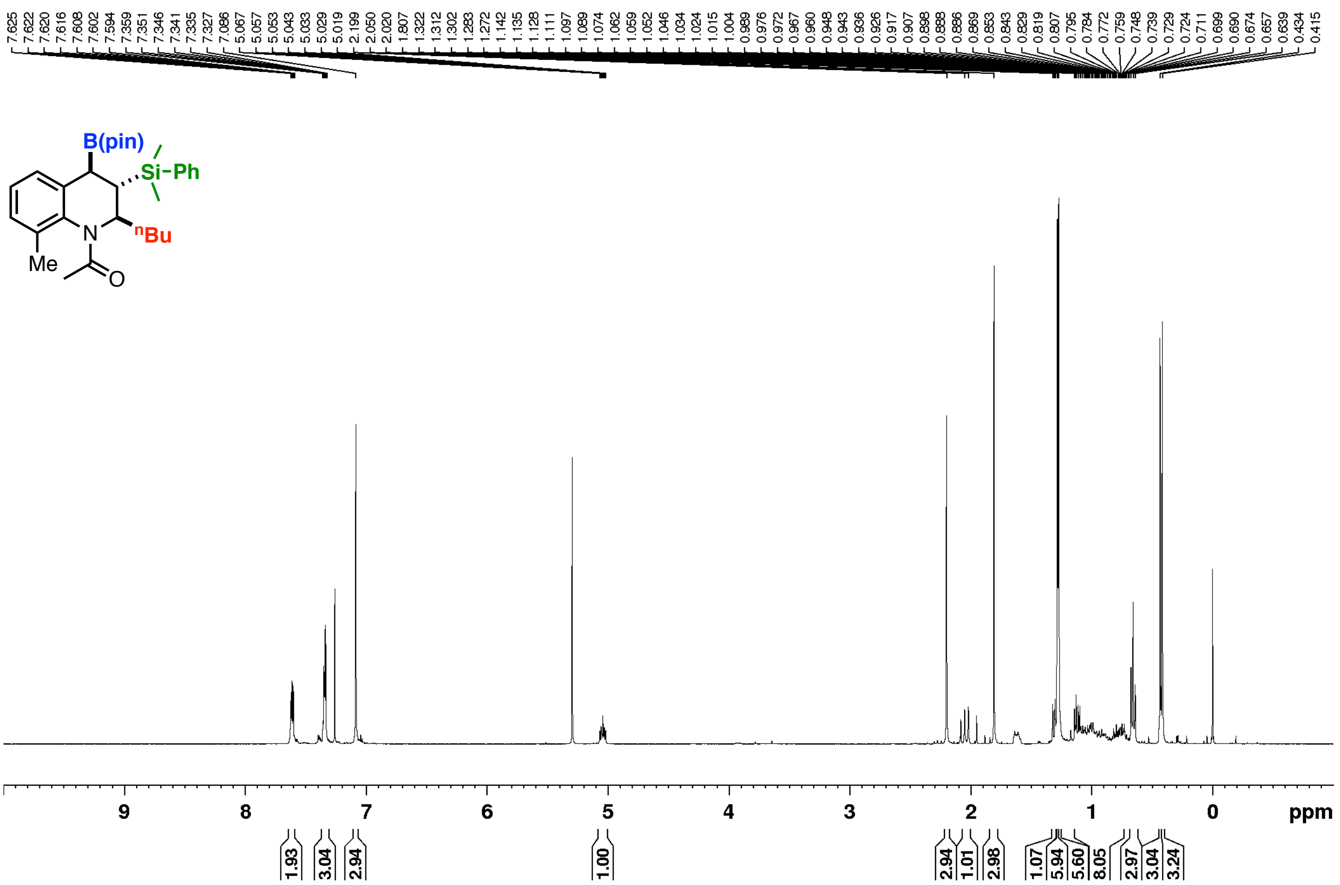

Supplementary Fig 15. **^{1}H NMR spectrum (400 MHz, $CDCl_3$, r.t.) of 3ea**.

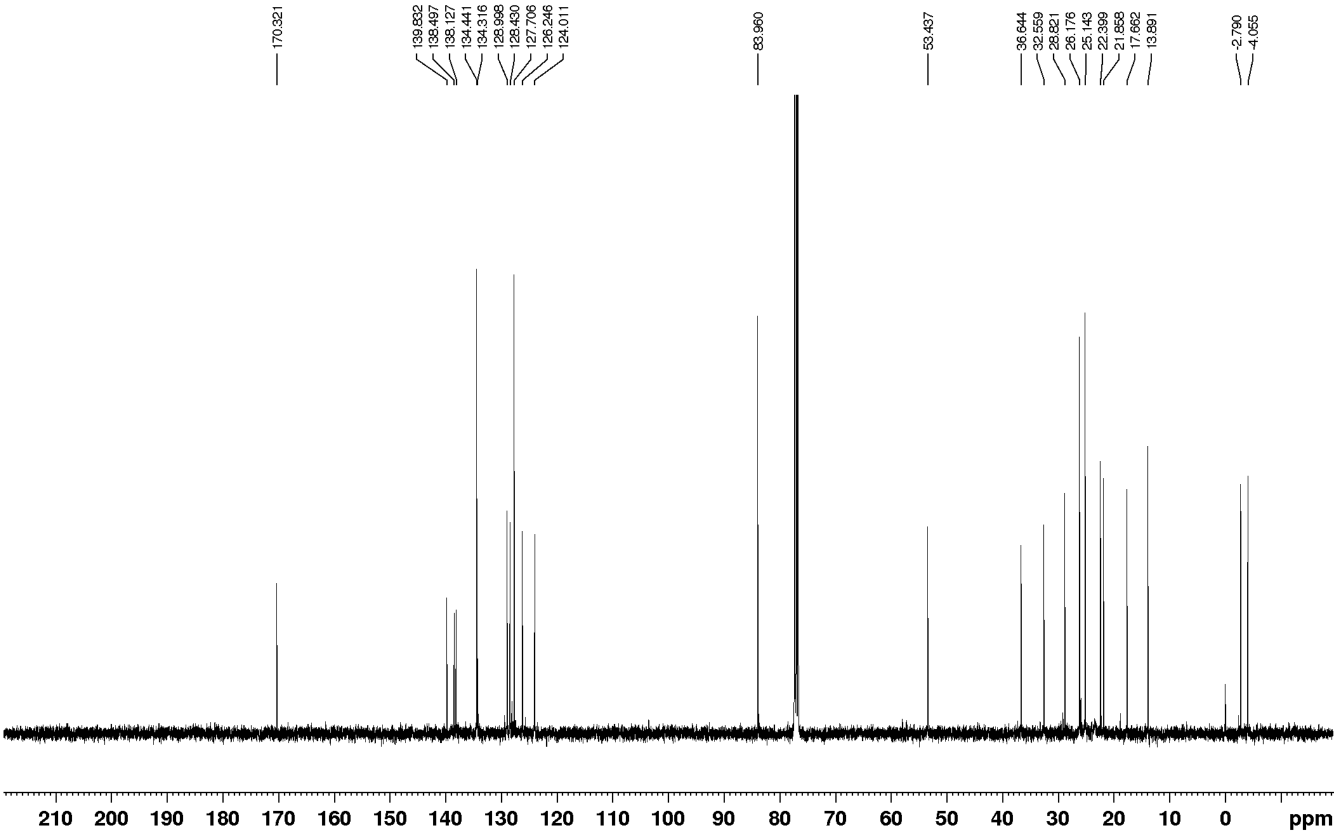

Supplementary Fig 16. **^{13}C NMR spectrum (100 MHz, $CDCl_3$, r.t.) of 3ea**.

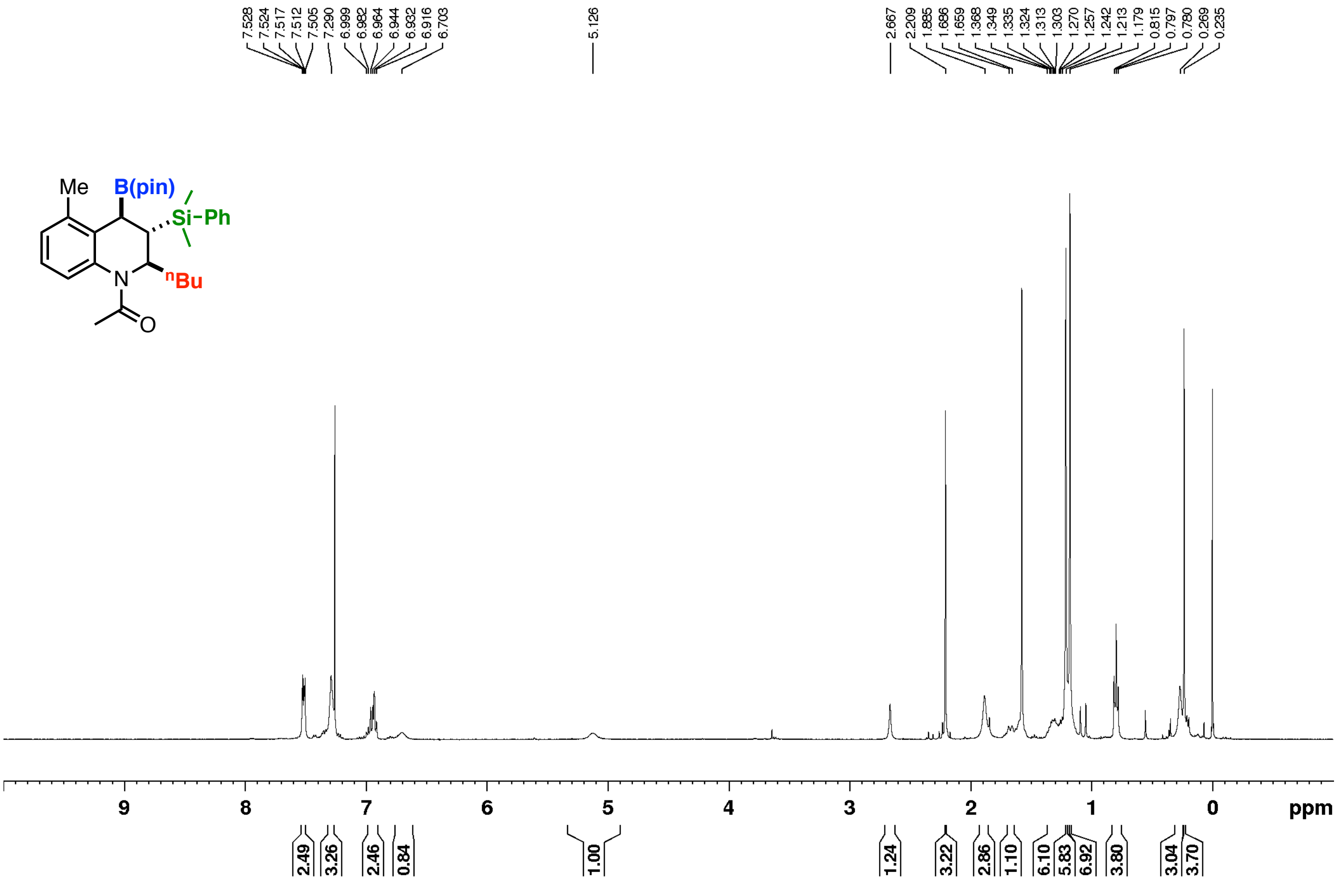

Supplementary Fig 17. ^{1}H NMR spectrum (400 MHz, $CDCl_3$, r.t.) of 3fa.

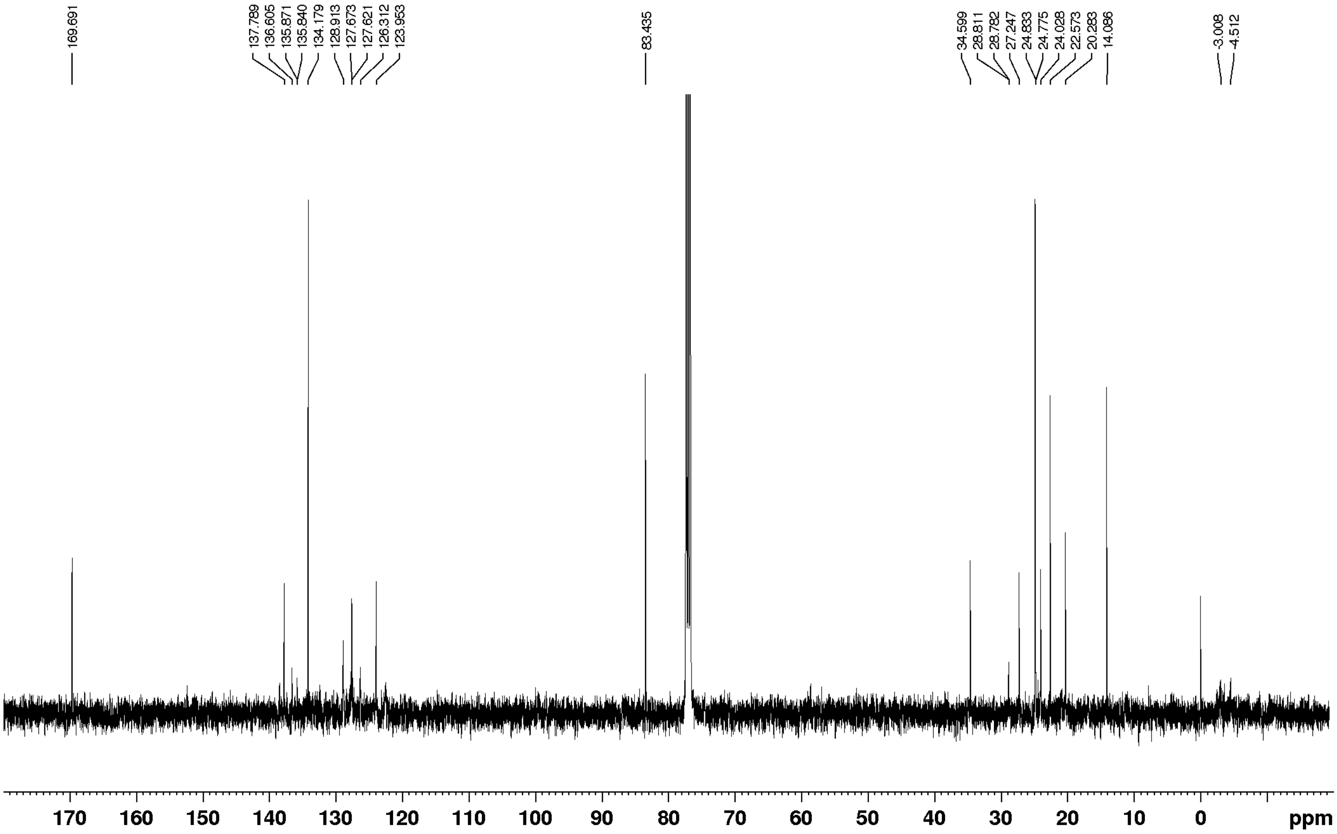

Supplementary Fig 18. ^{13}C NMR spectrum (100 MHz, $CDCl_3$, r.t.) of 3fa.

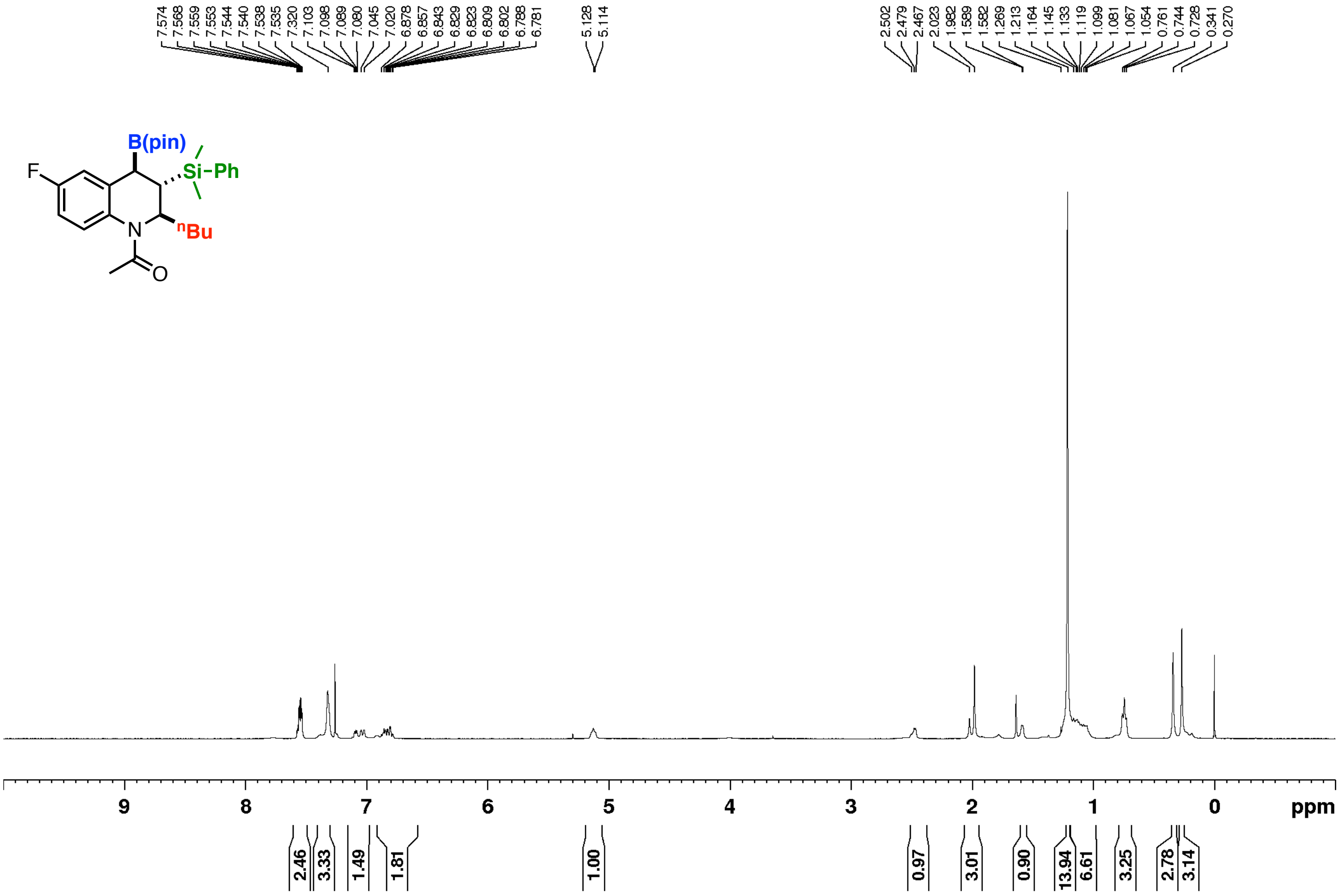

Supplementary Fig 19. **^{1}H NMR spectrum (400 MHz, $CDCl_3$, r.t.) of 3ga**.

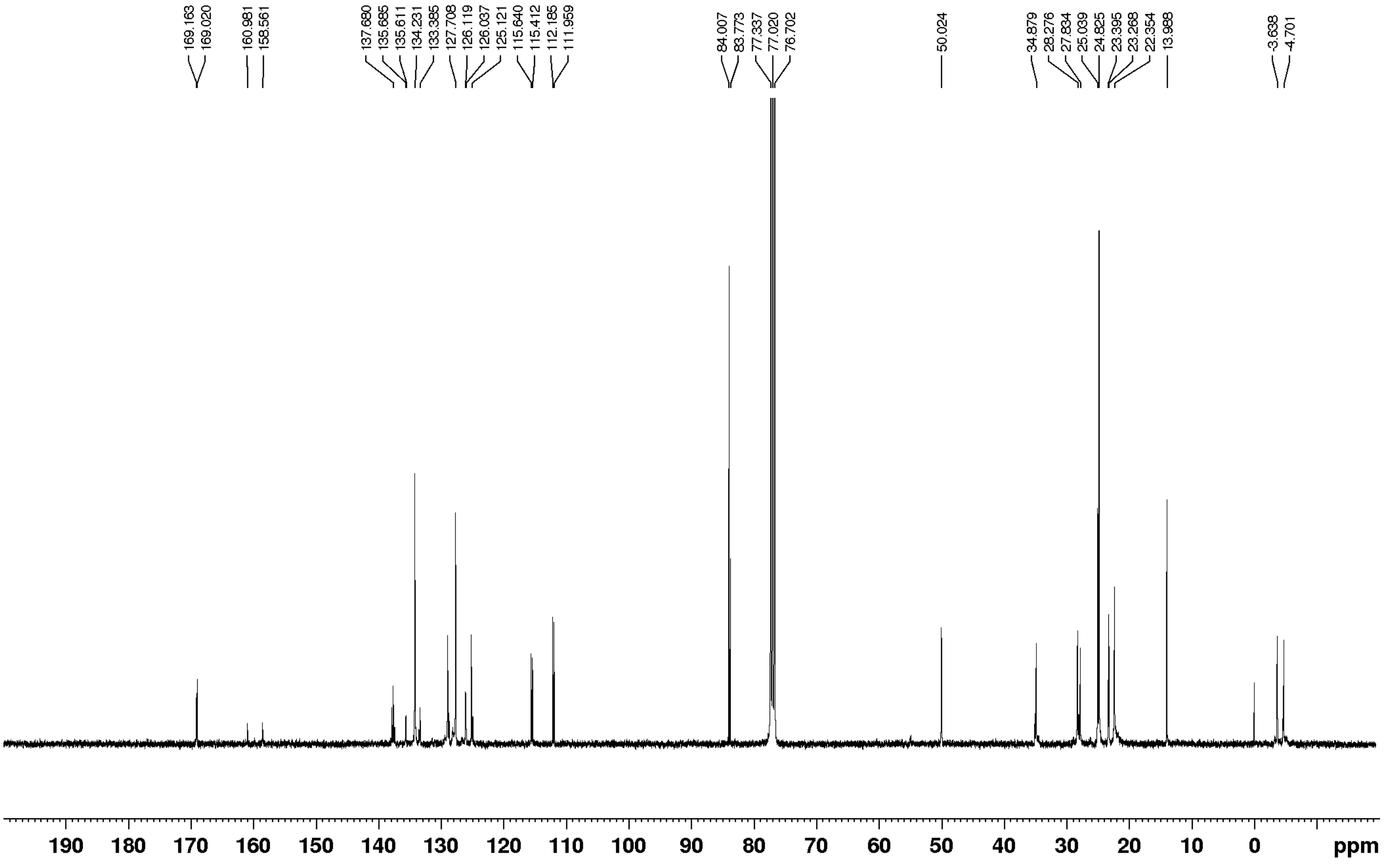

Supplementary Fig 20. **^{13}C NMR spectrum (100 MHz, $CDCl_3$, r.t.) of 3ga**.

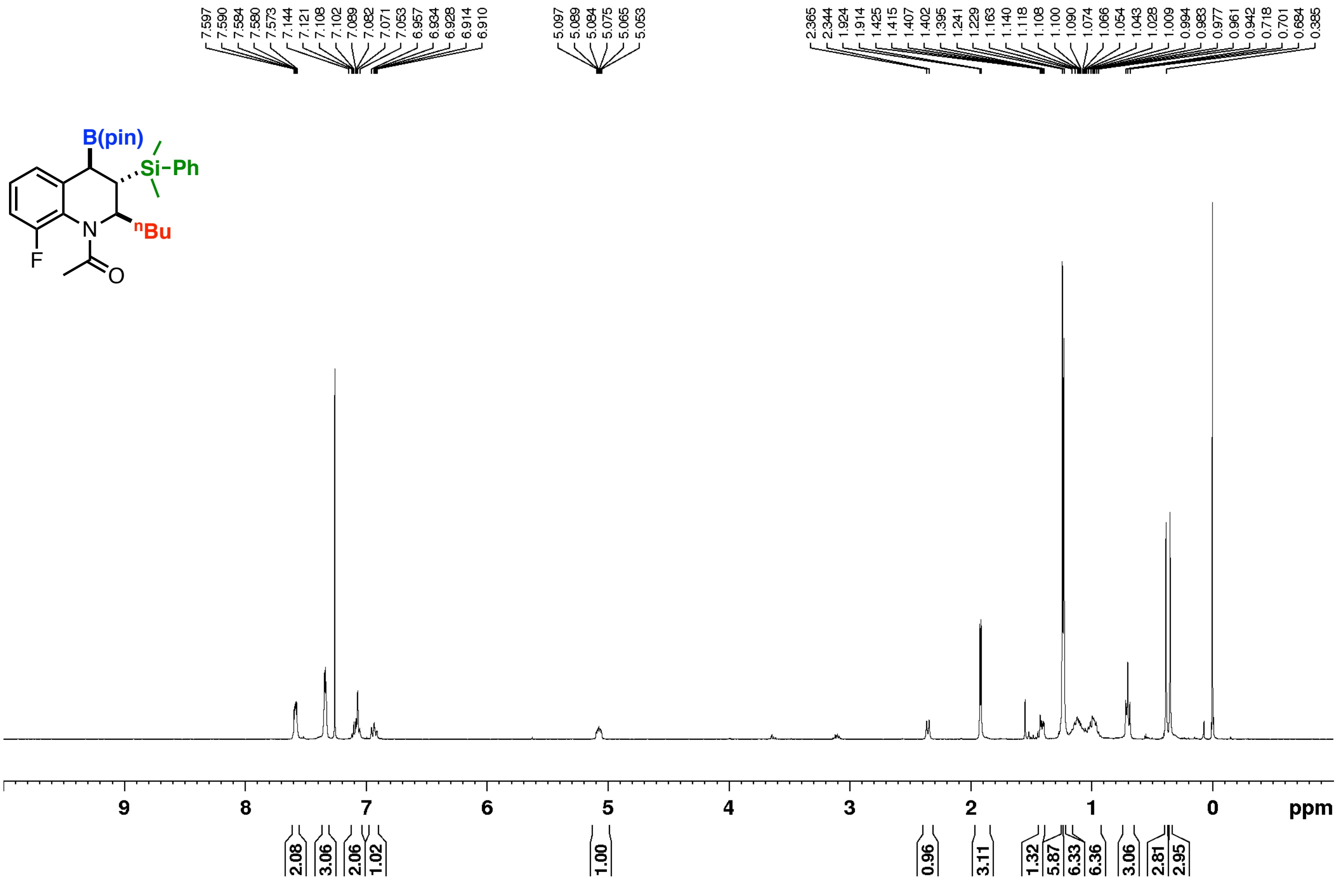

Supplementary Fig 21. ^{1}**H NMR spectrum (400 MHz, $CDCl_3$, r.t.) of 3ha**.

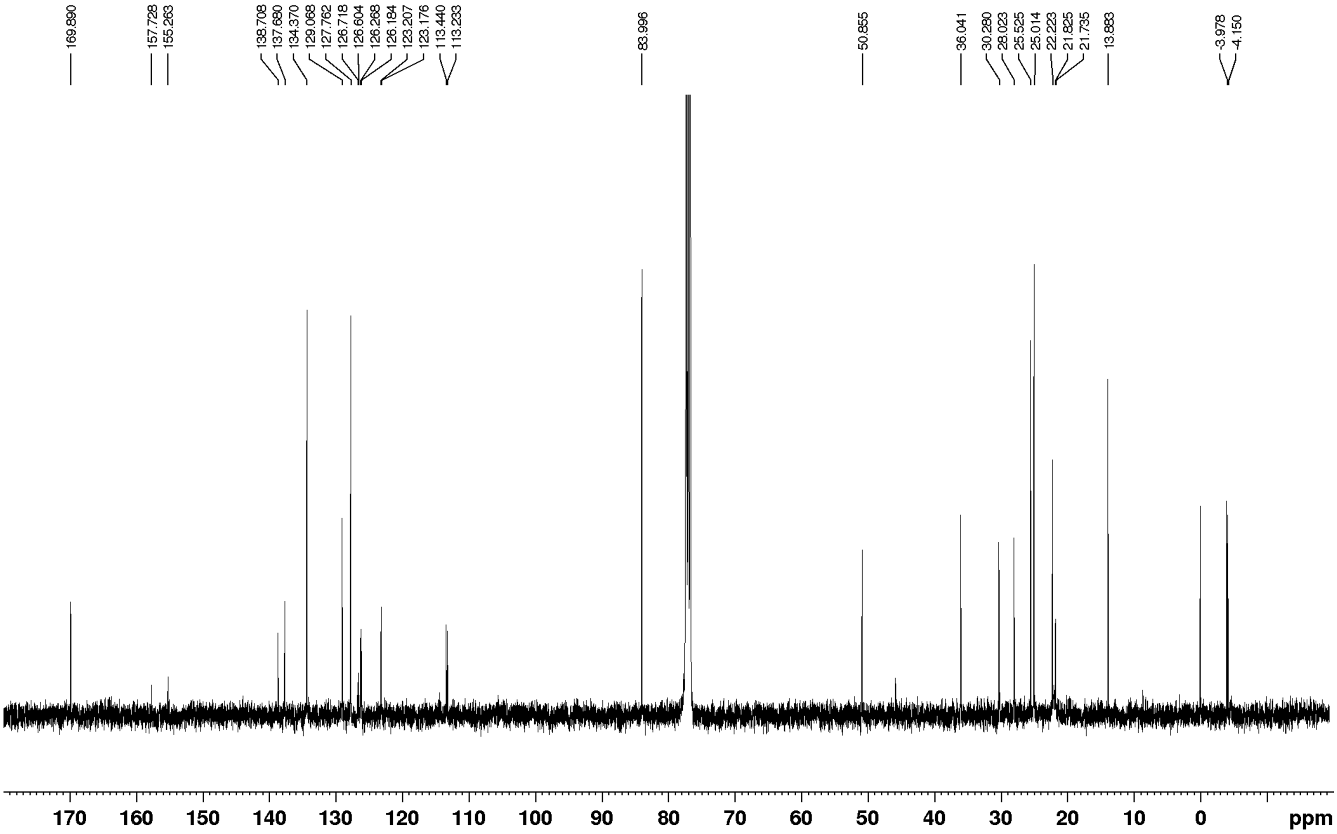

Supplementary Fig 22. ^{13}**C NMR spectrum (100 MHz, $CDCl_3$, r.t.) of 3ha**.

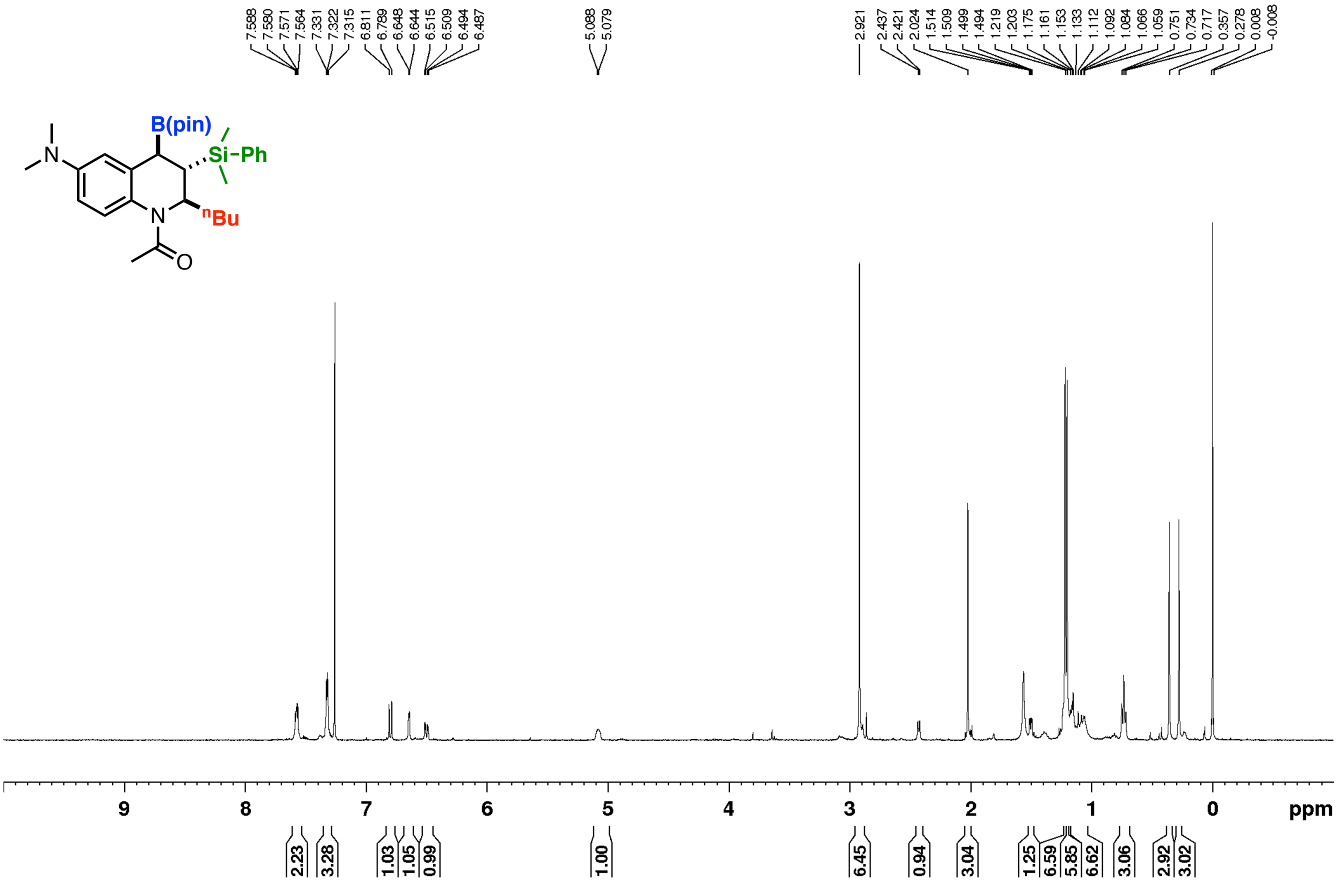

Supplementary Fig 23. ^{1}**H NMR spectrum (400 MHz, CDCl$_3$, r.t.) of 3ia**.

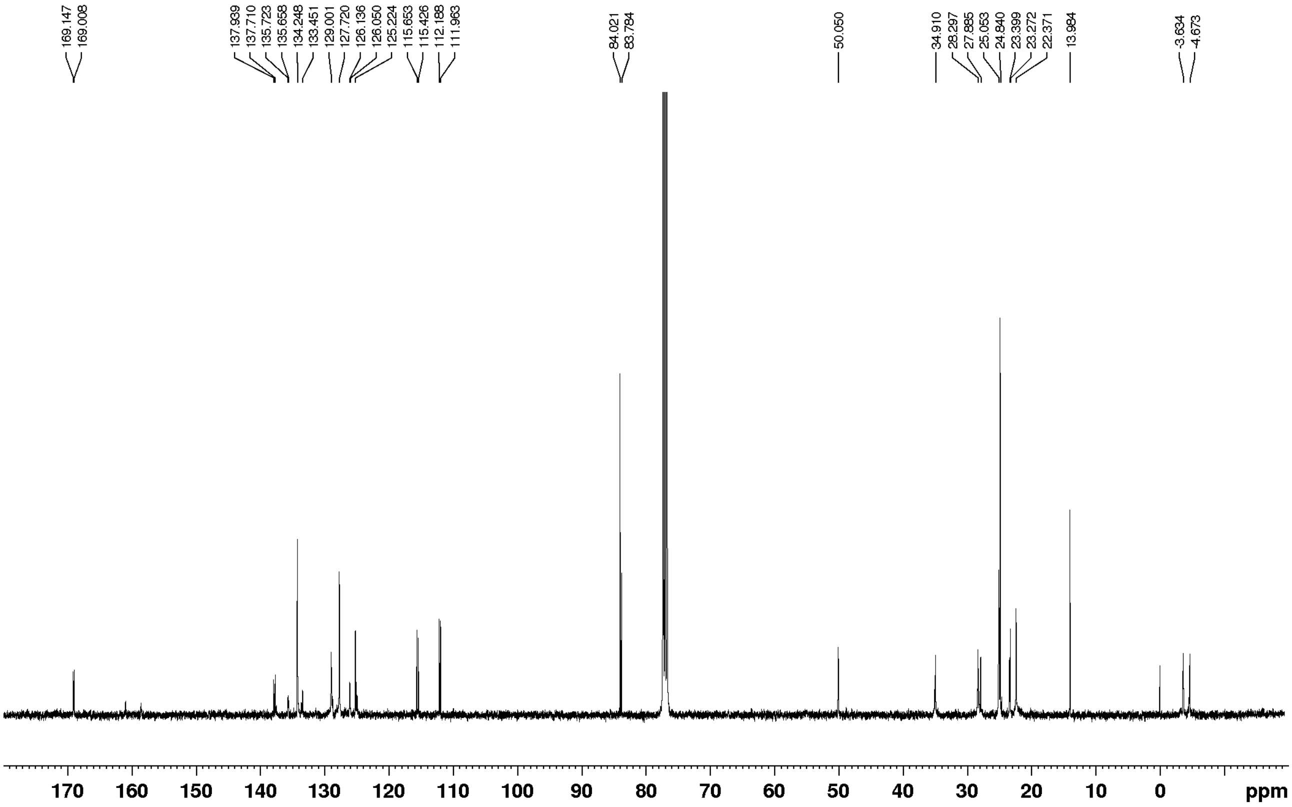

Supplementary Fig 24. ^{13}**C NMR spectrum (100 MHz, CDCl$_3$, r.t.) of 3ia**.

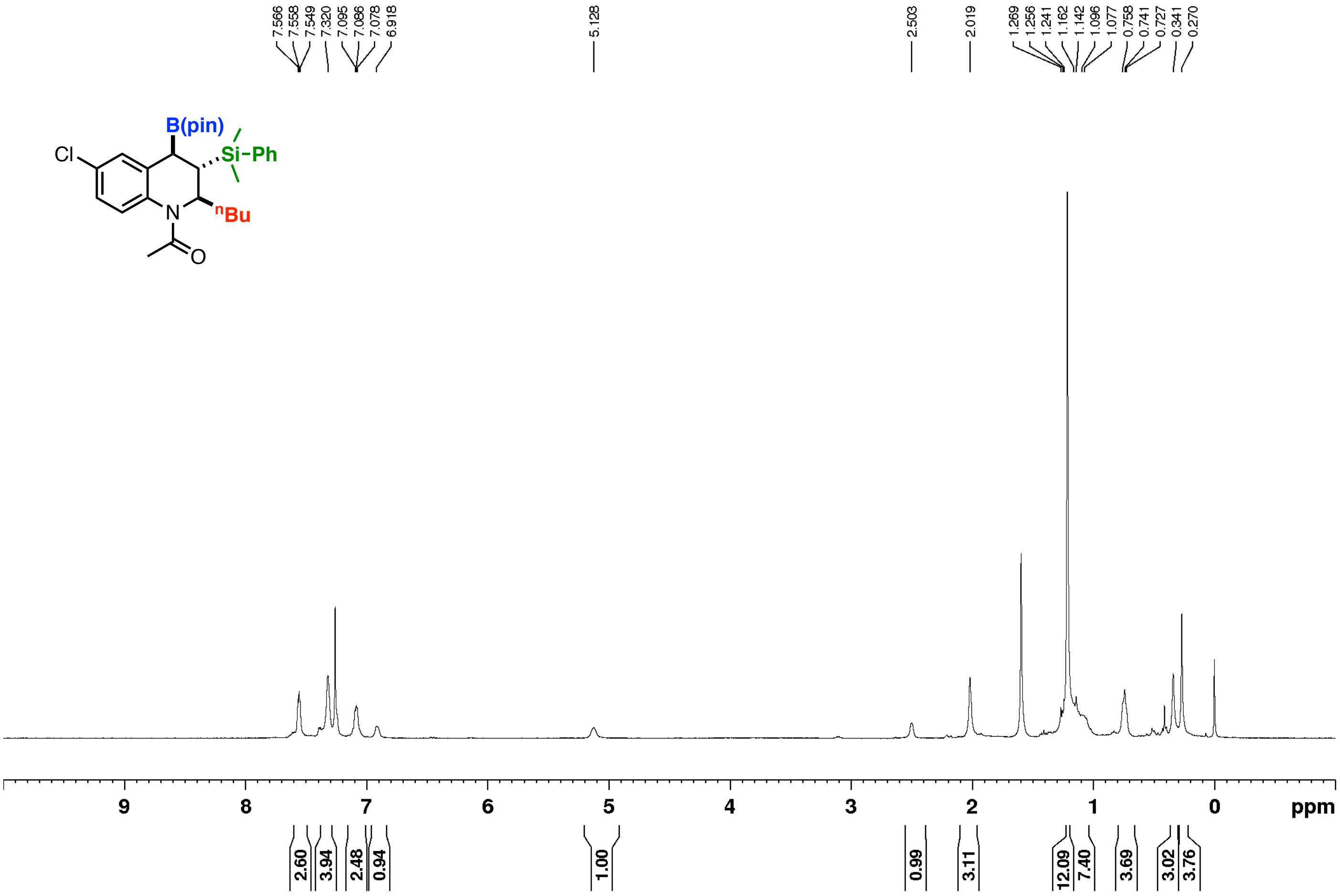

Supplementary Fig 25. **^{1}H NMR spectrum (400 MHz, $CDCl_3$, r.t.) of 3ja**.

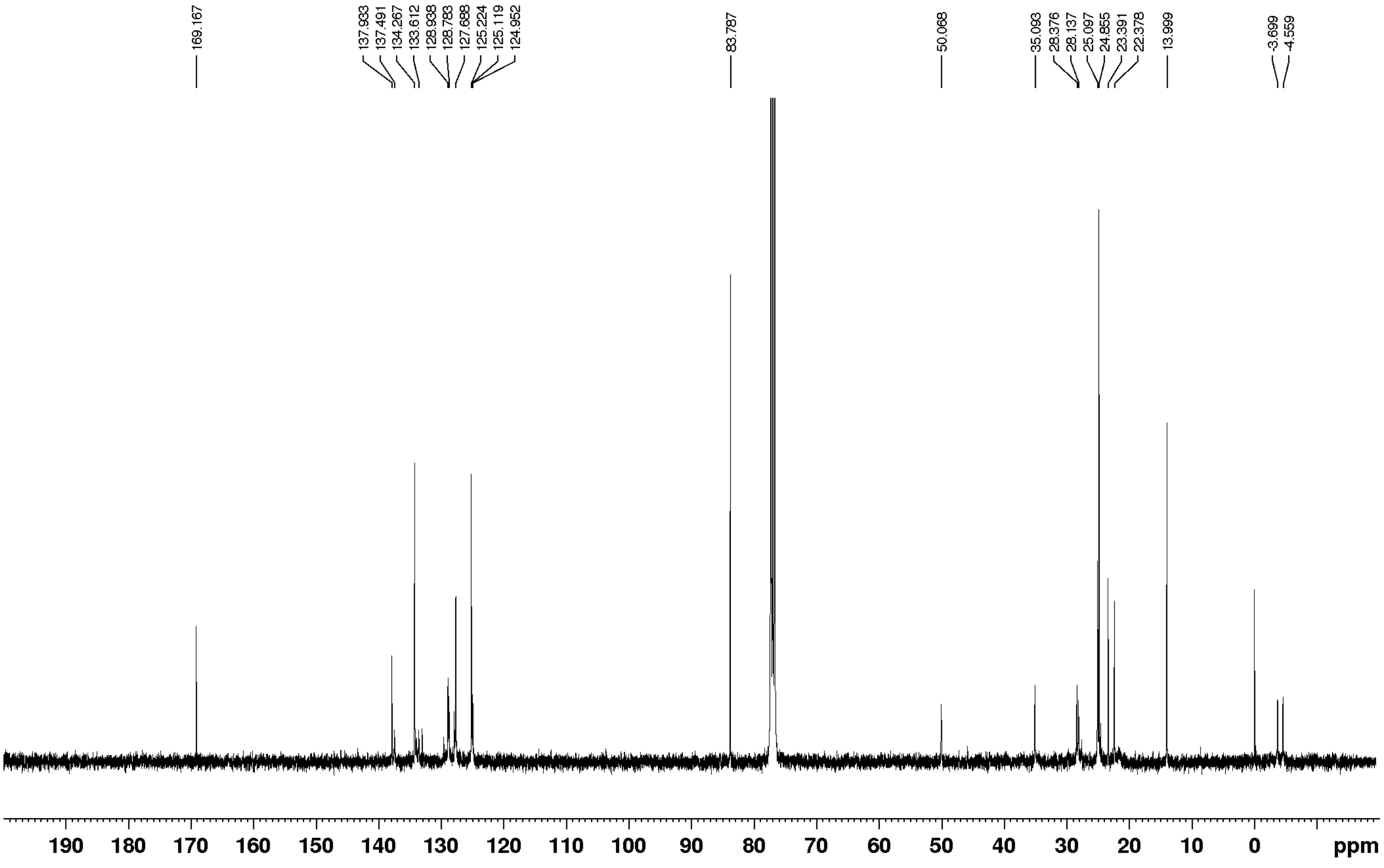

Supplementary Fig 26. **^{13}C NMR spectrum (100 MHz, $CDCl_3$, r.t.) of 3ja**.

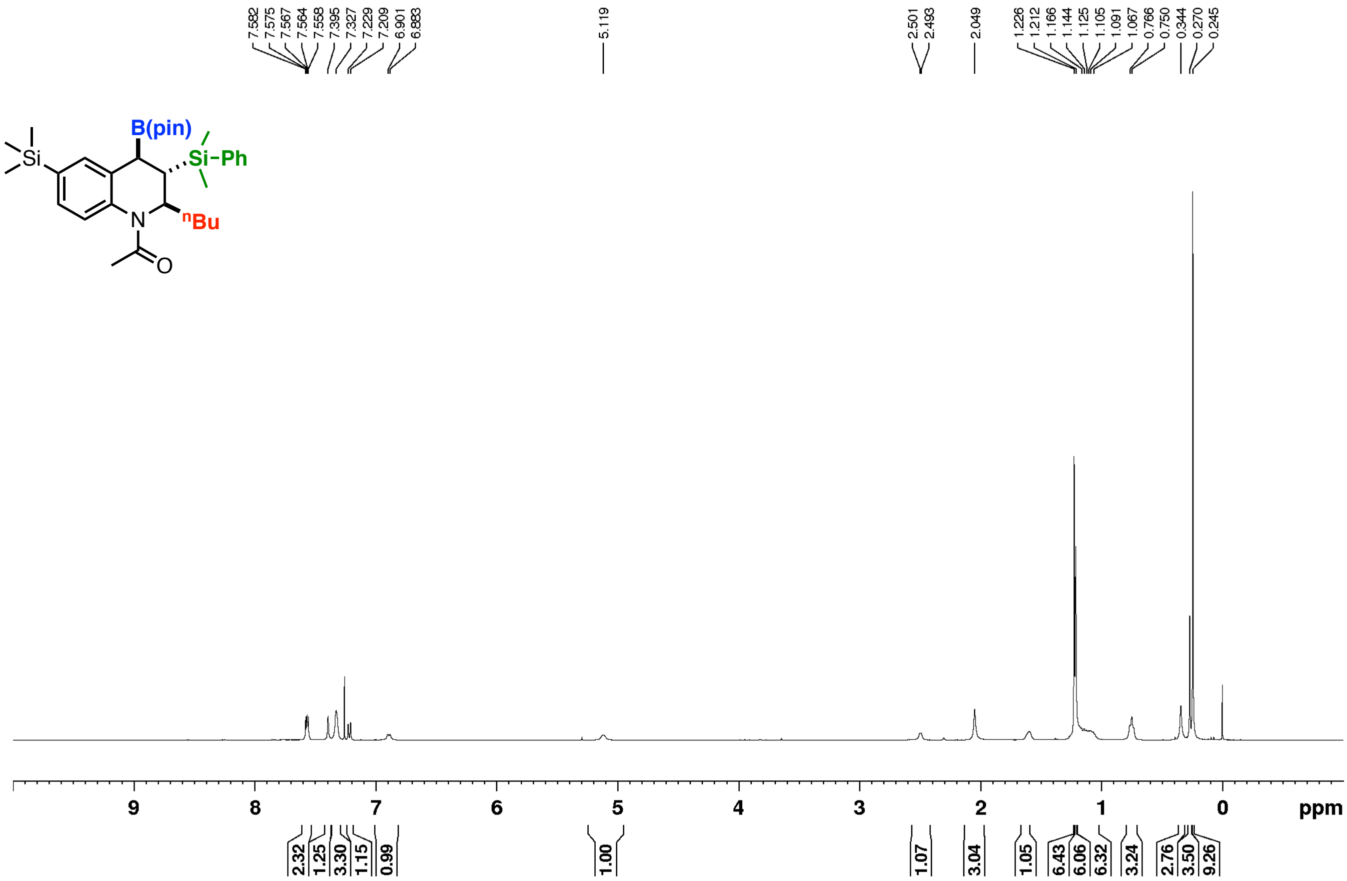

Supplementary Fig 27. ^{1}H NMR spectrum (400 MHz, $CDCl_3$, r.t.) of 3ka.

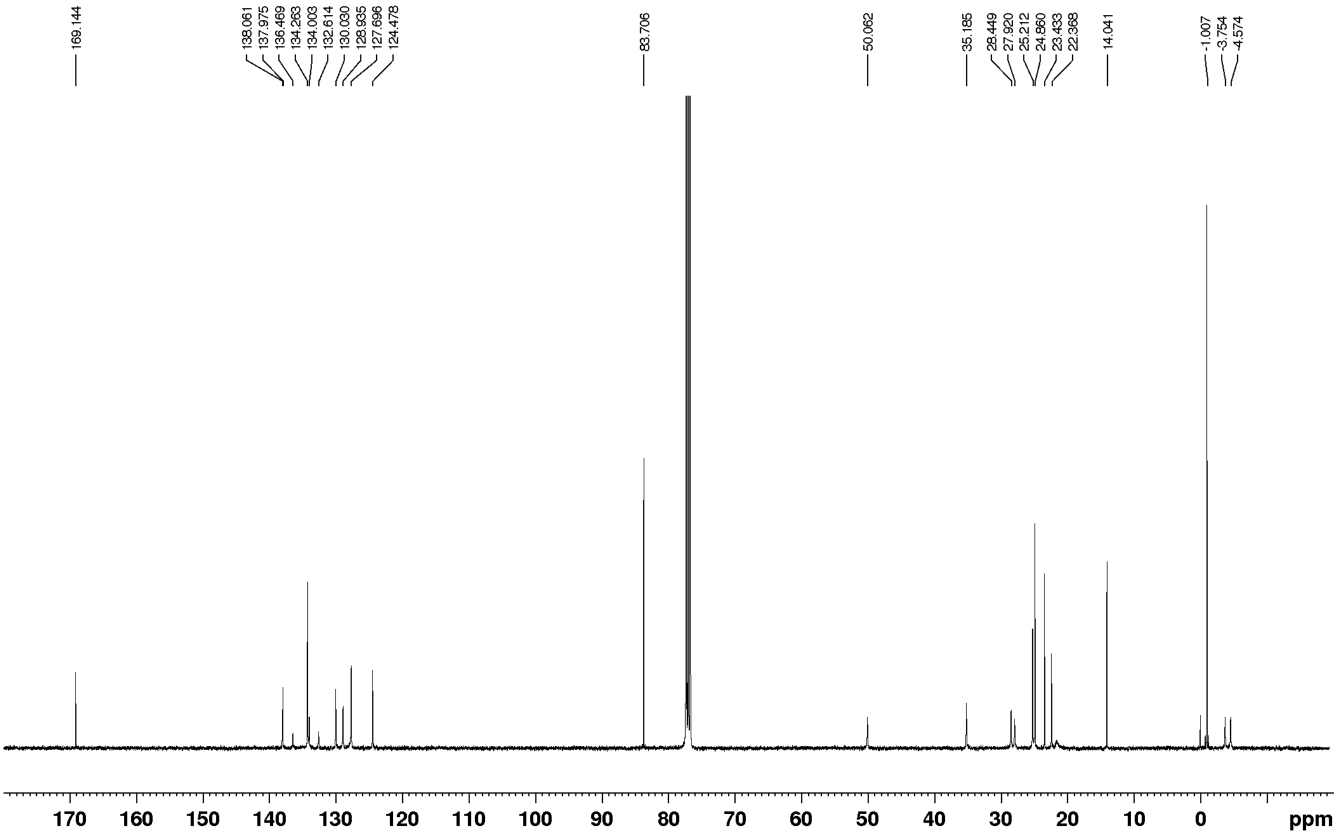

Supplementary Fig 28. ^{13}C NMR spectrum (100 MHz, $CDCl_3$, r.t.) of 3ka.

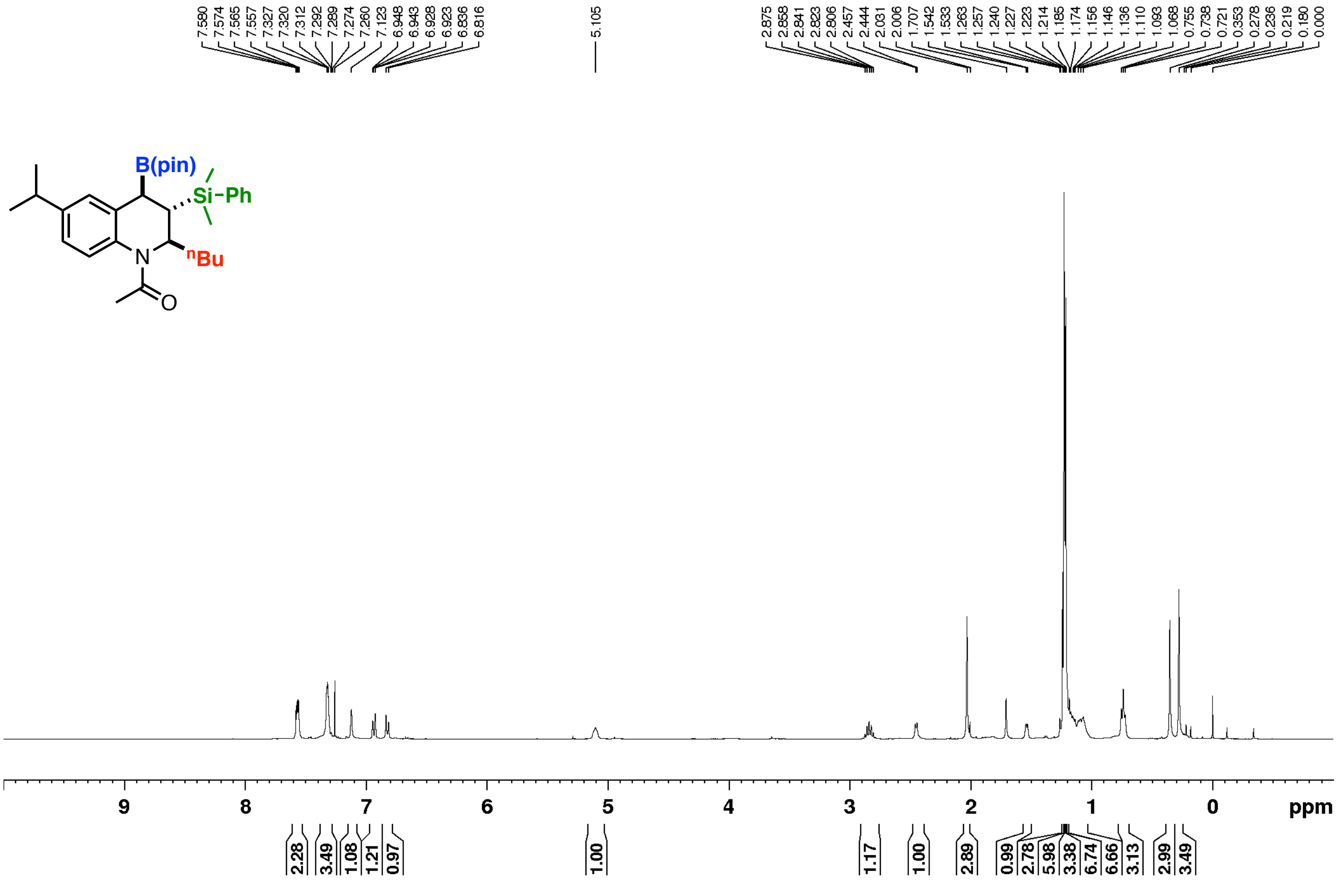

Supplementary Fig 29. ^{1}**H NMR spectrum (400 MHz, CDCl$_3$, r.t.) of 3la**.

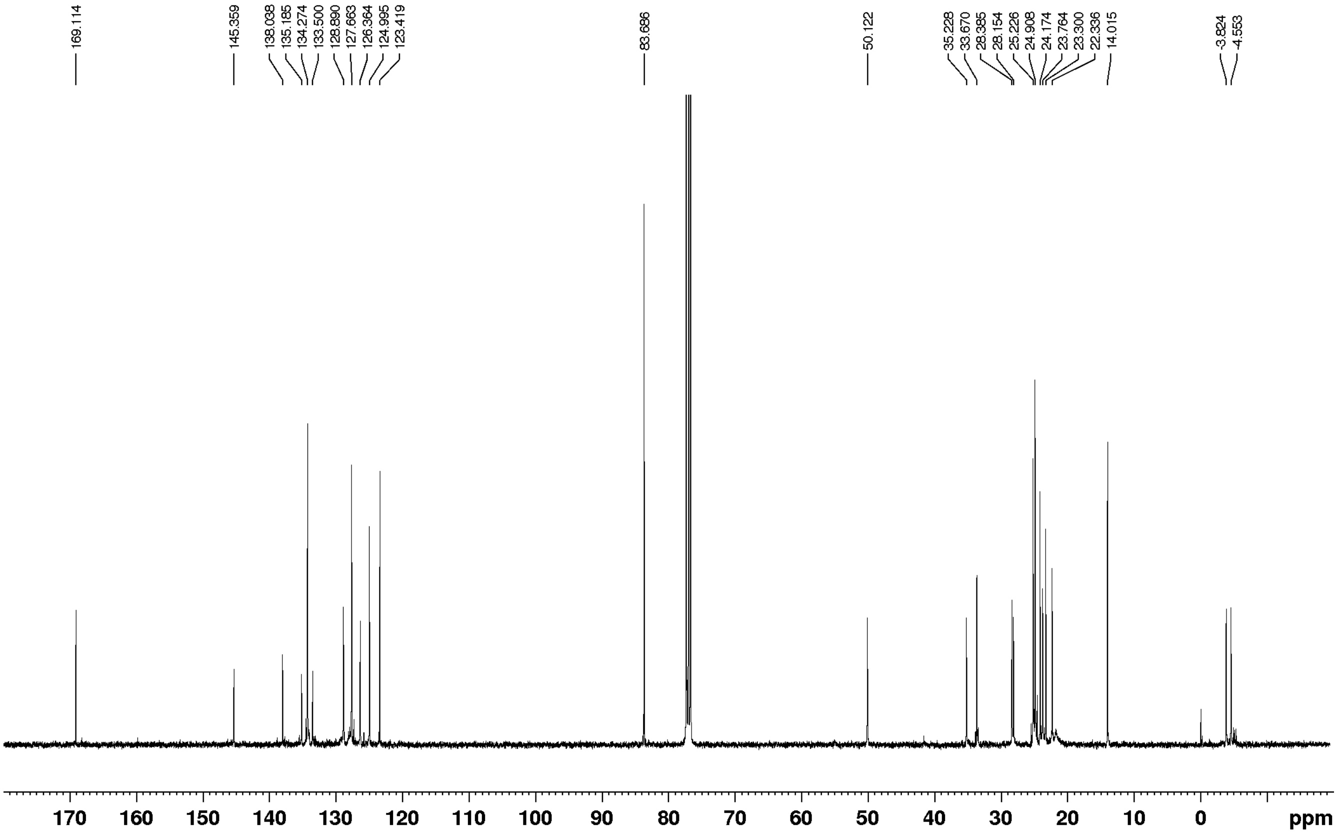

Supplementary Fig 30. ^{13}**C NMR spectrum (100 MHz, CDCl$_3$, r.t.) of 3la**.

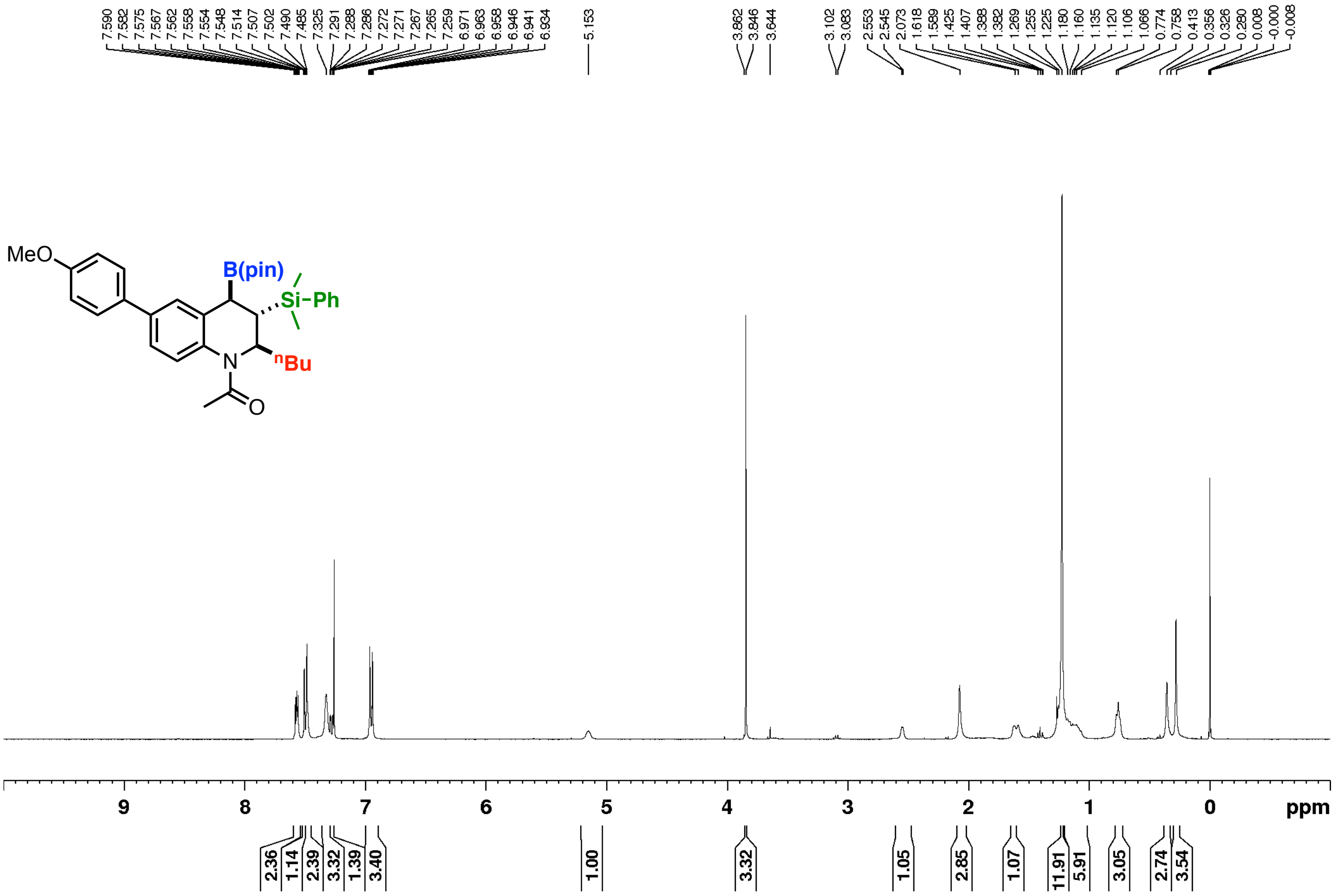

Supplementary Fig 31. ^{1}H **NMR spectrum (400 MHz,** $CDCl_3$**, r.t.) of 3ma**.

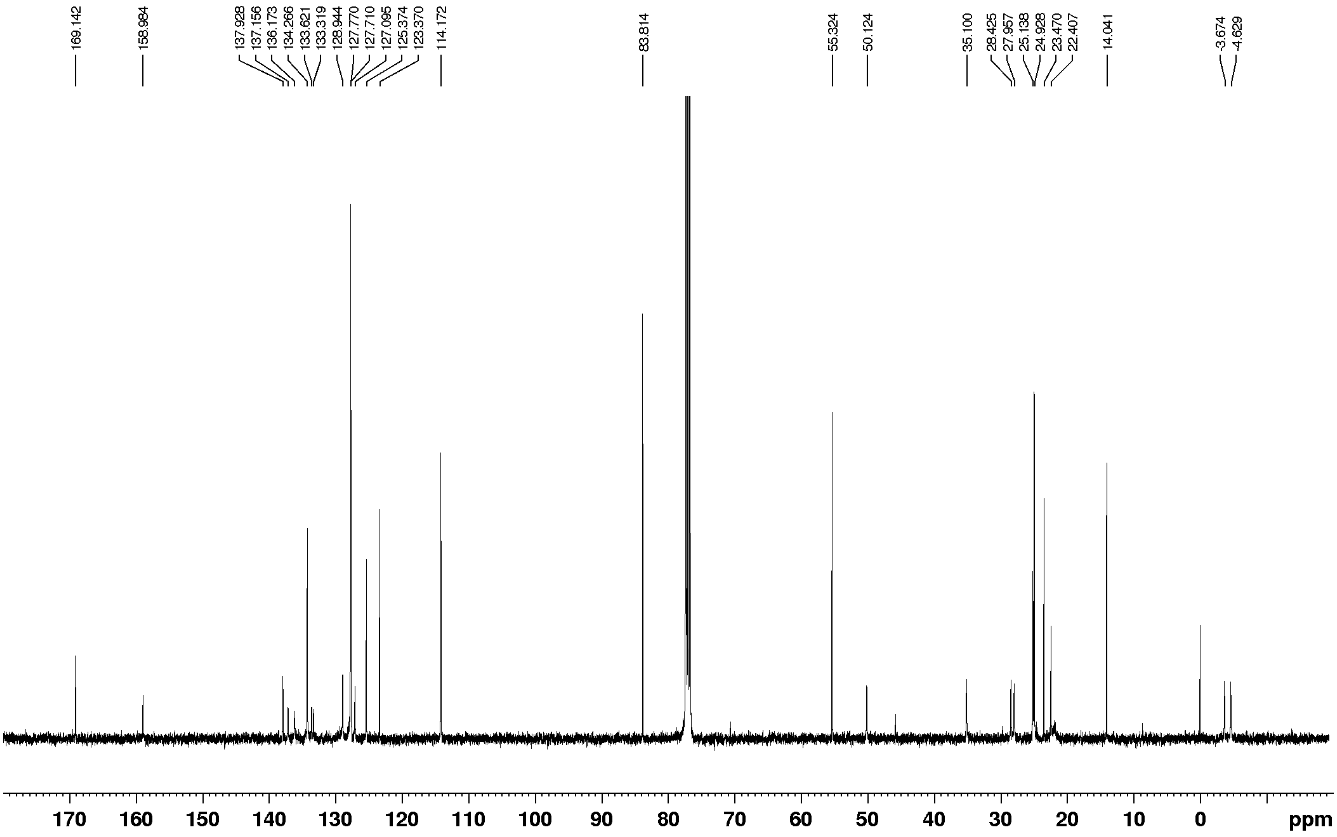

Supplementary Fig 32. ^{13}C **NMR spectrum (100 MHz,** $CDCl_3$**, r.t.) of 3ma**.

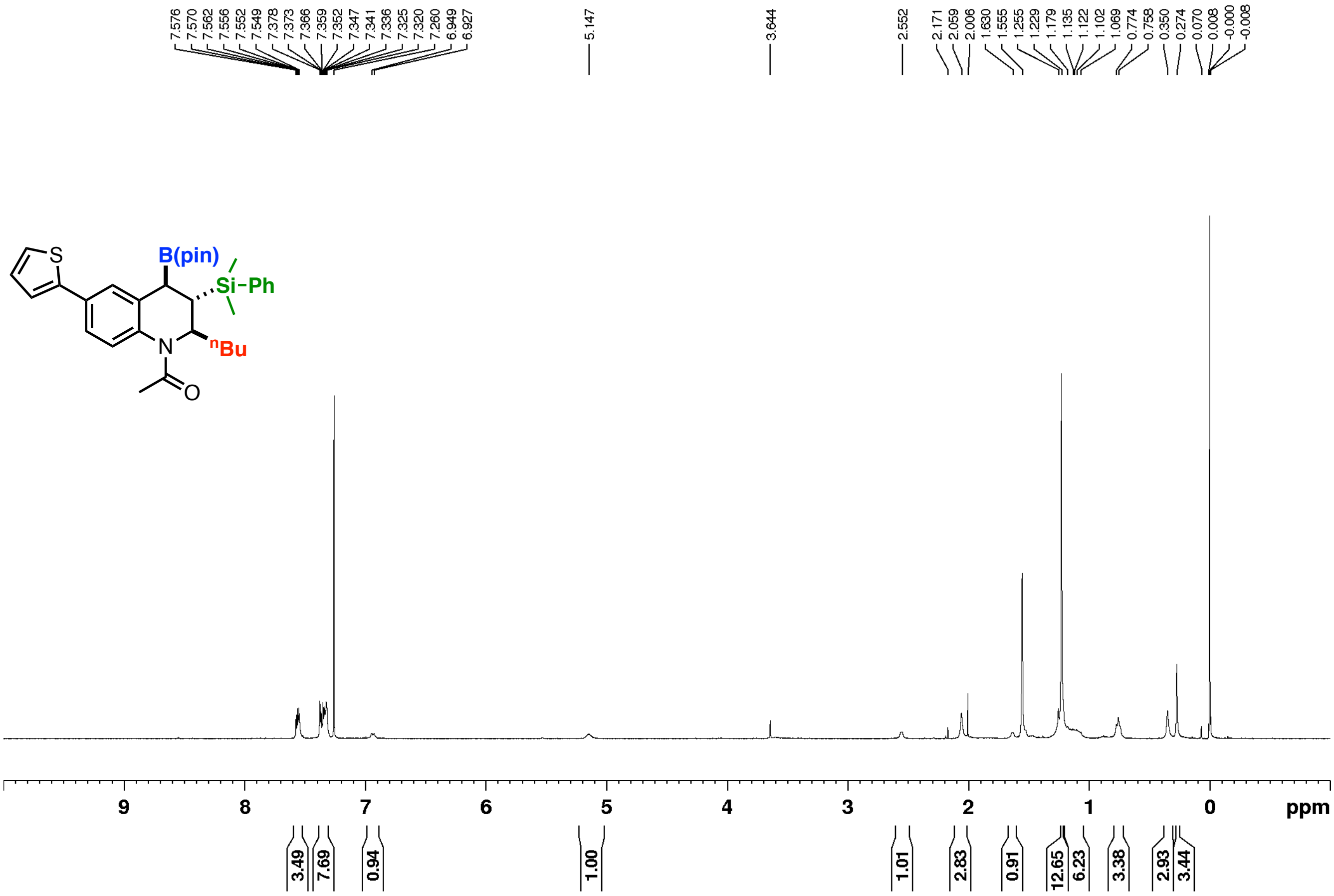

Supplementary Fig 33. ^{1}**H NMR spectrum (400 MHz, CDCl$_3$, r.t.) of 3na**.

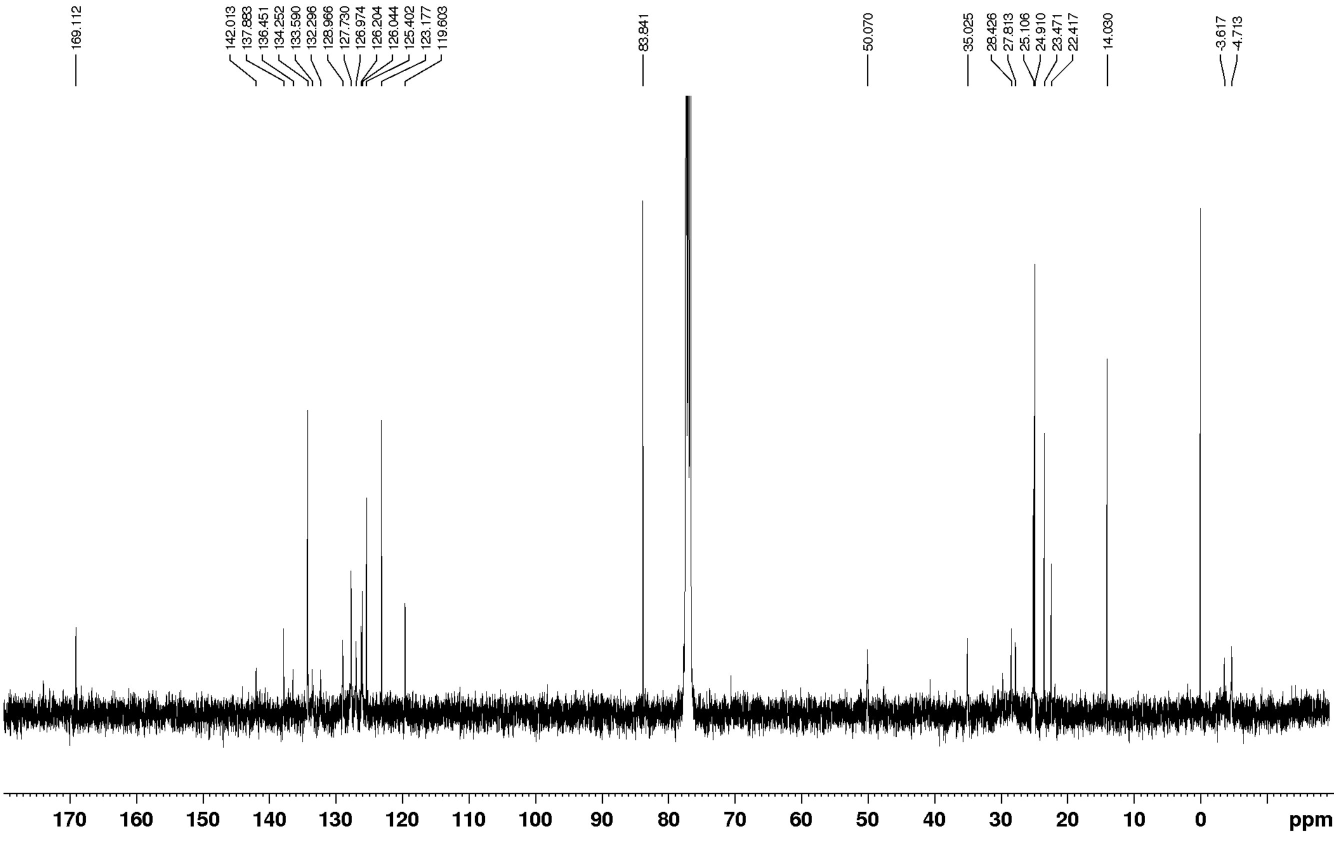

Supplementary Fig 34. ^{13}**C NMR spectrum (100 MHz, CDCl$_3$, r.t.) of 3na**.

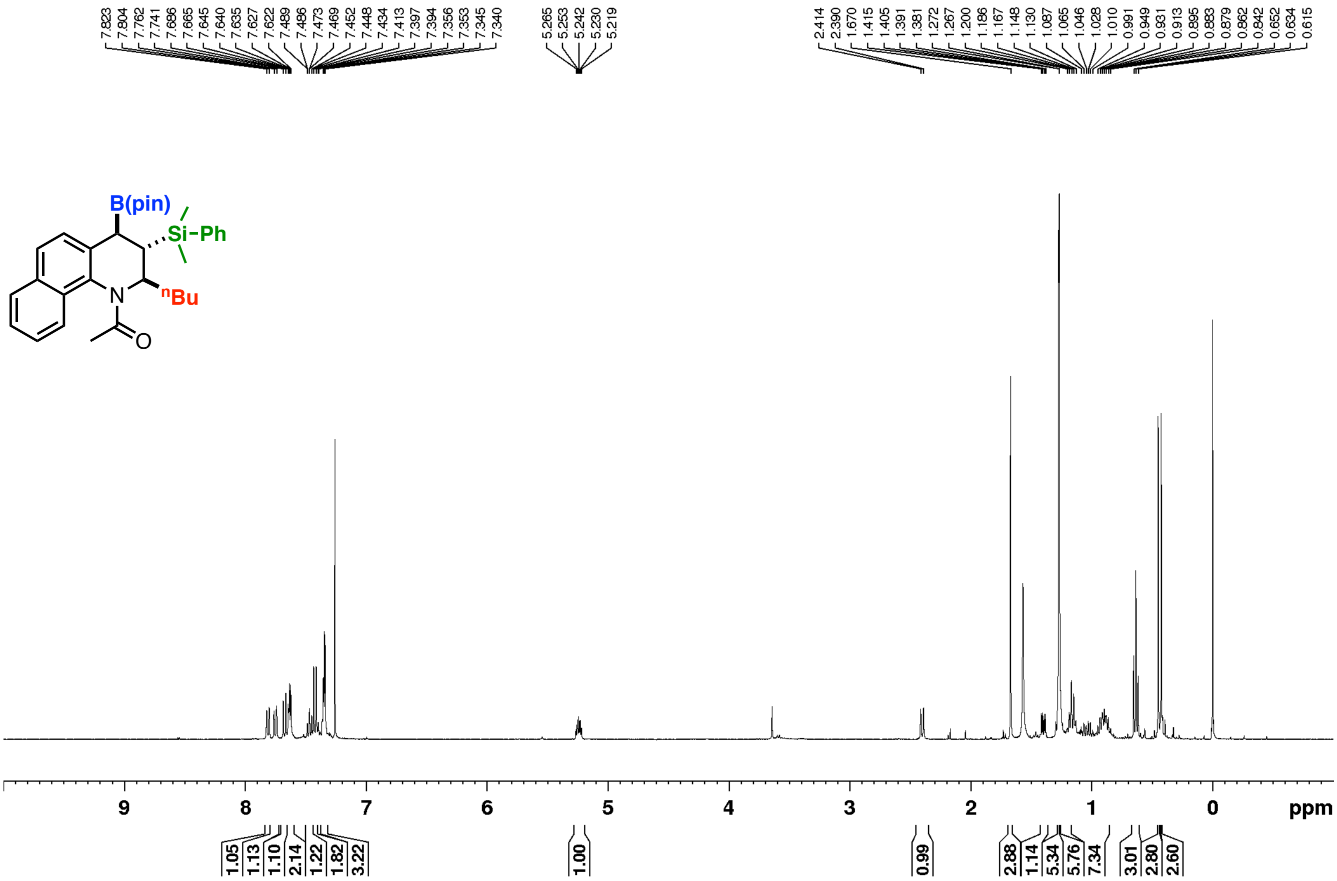

Supplementary Fig 35. ^{1}H NMR spectrum (400 MHz, $CDCl_3$, r.t.) of 3oa.

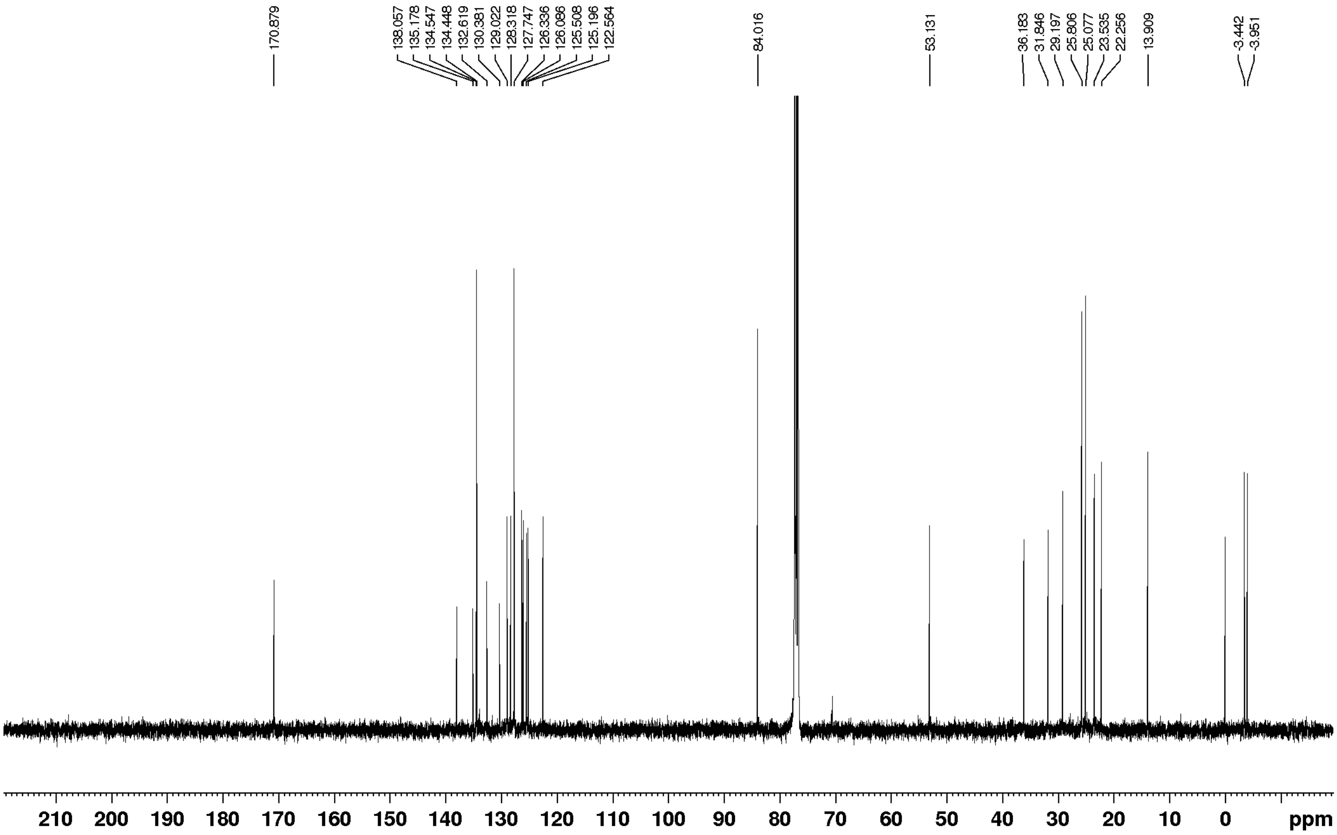

Supplementary Fig 36. ^{13}C NMR spectrum (100 MHz, $CDCl_3$, r.t.) of 3oa.

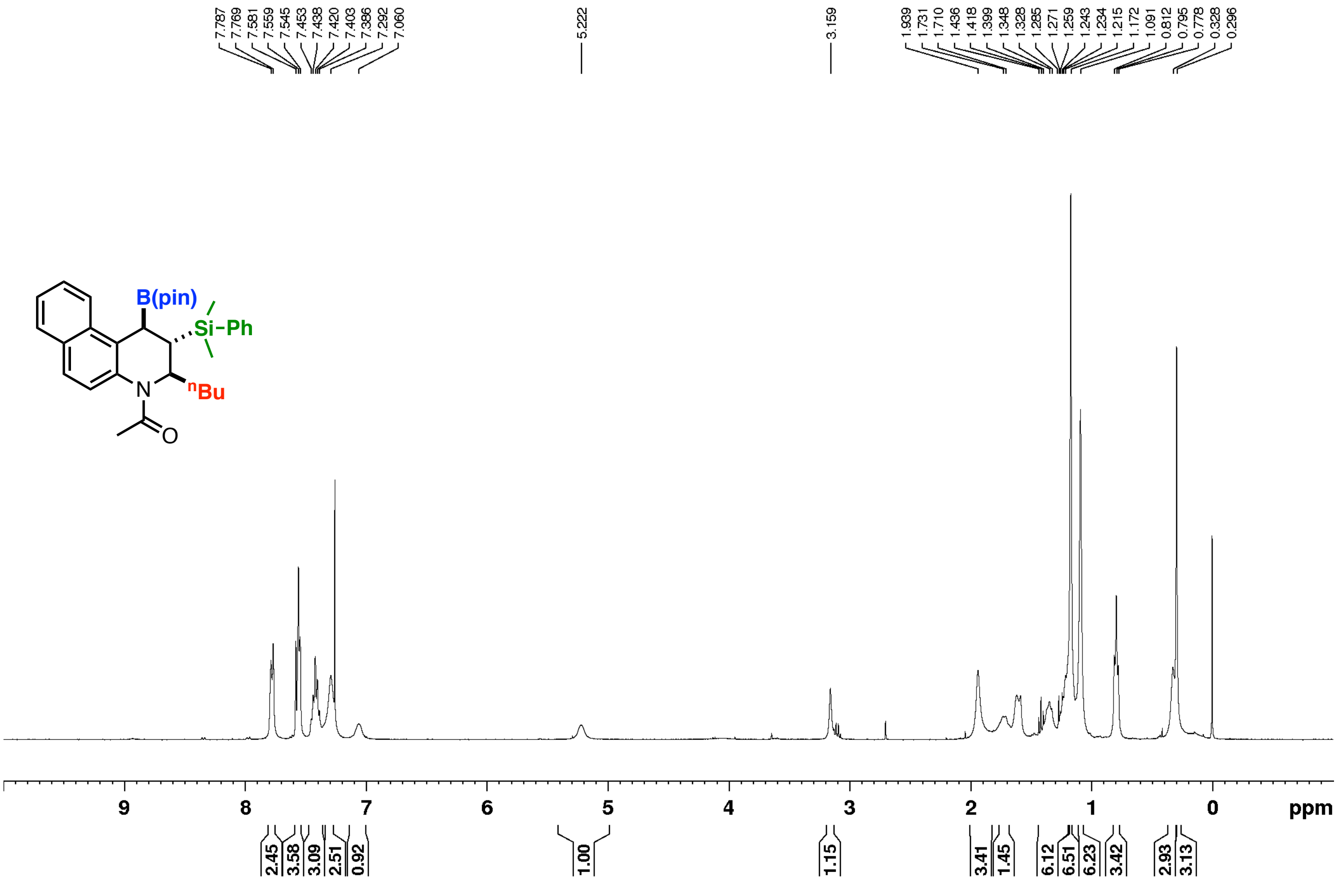

Supplementary Fig 37. **^{1}H NMR spectrum (400 MHz, $CDCl_3$, r.t.) of 3pa**.

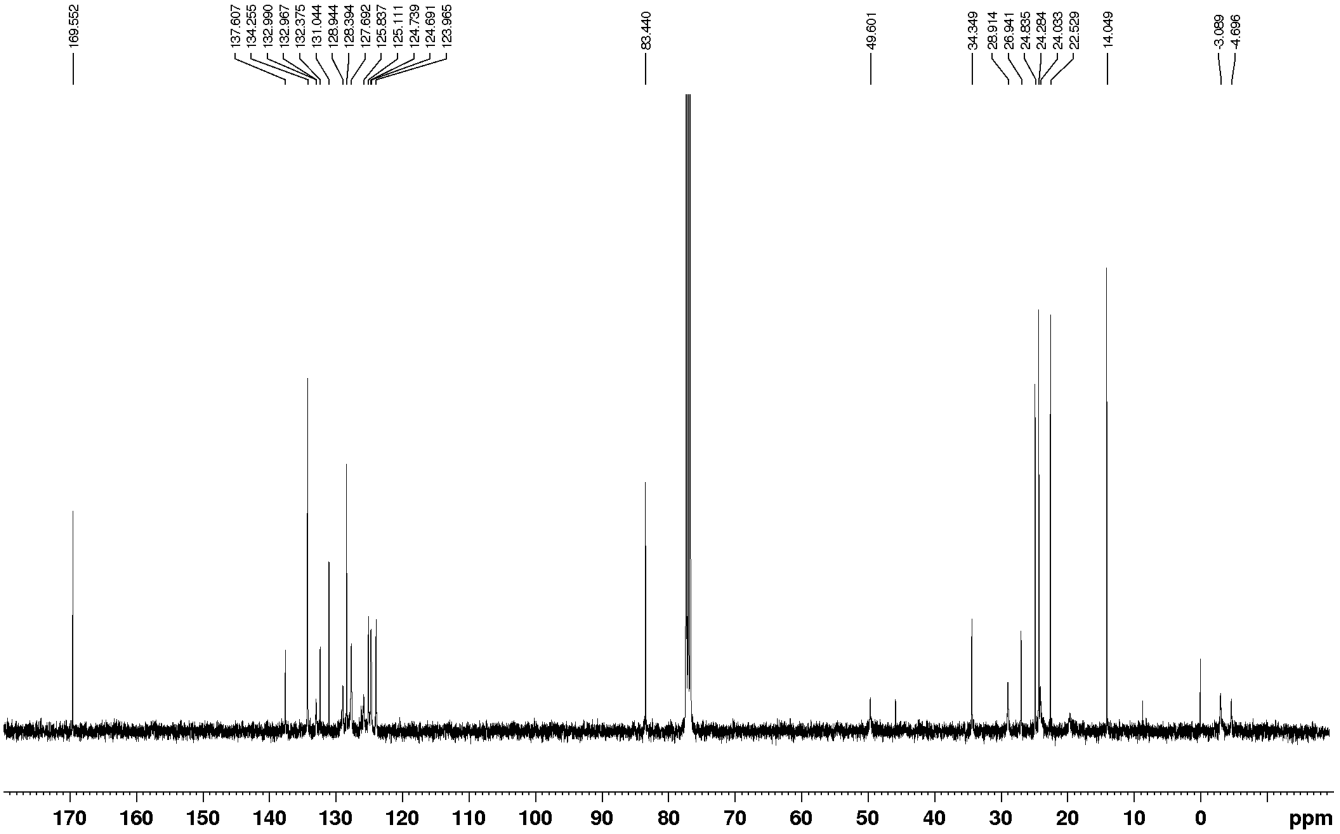

Supplementary Fig 38. **^{13}C NMR spectrum (100 MHz, $CDCl_3$, r.t.) of 3pa**.

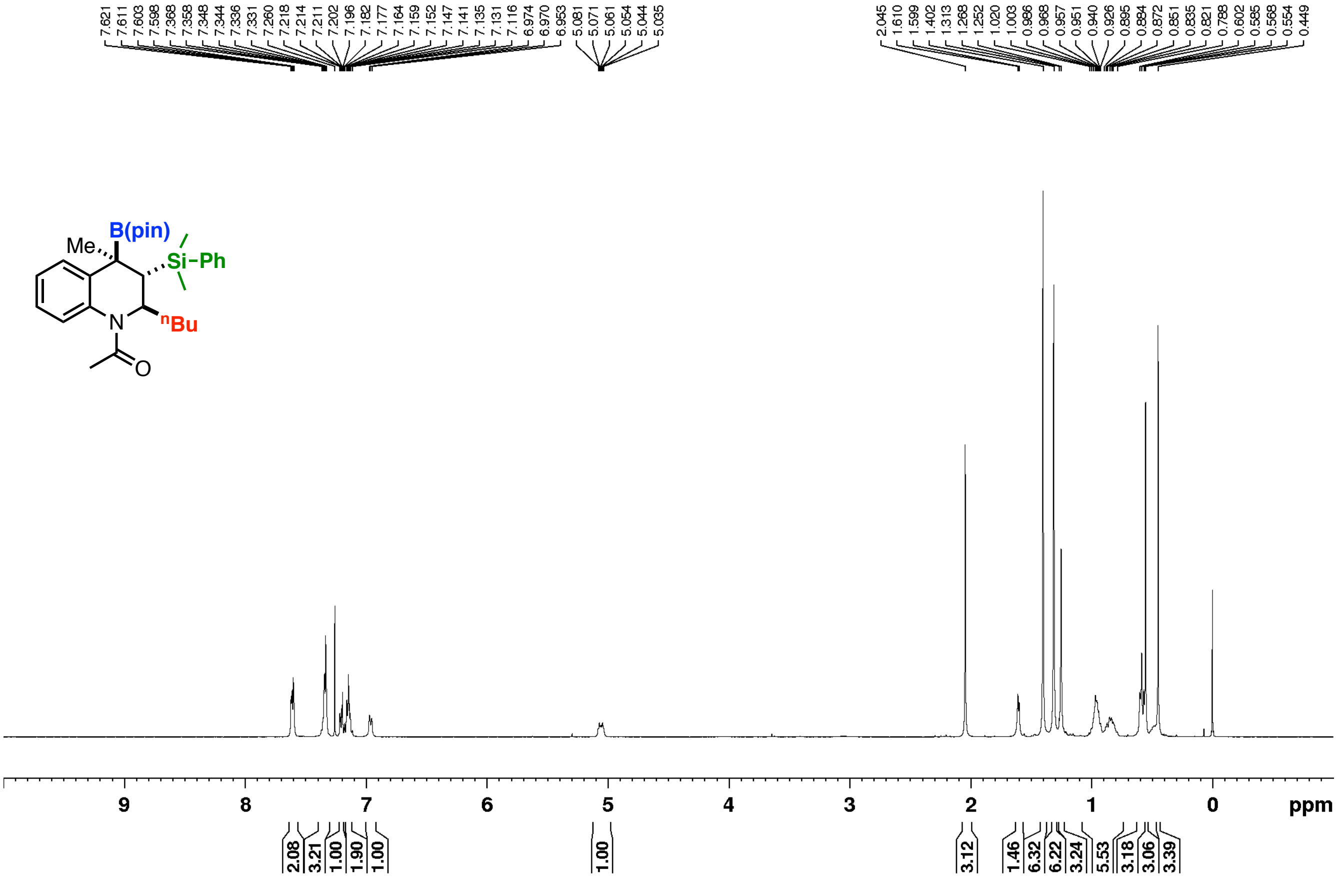

Supplementary Fig 39. **^{1}H NMR spectrum (400 MHz, $CDCl_3$, r.t.) of 3qa**.

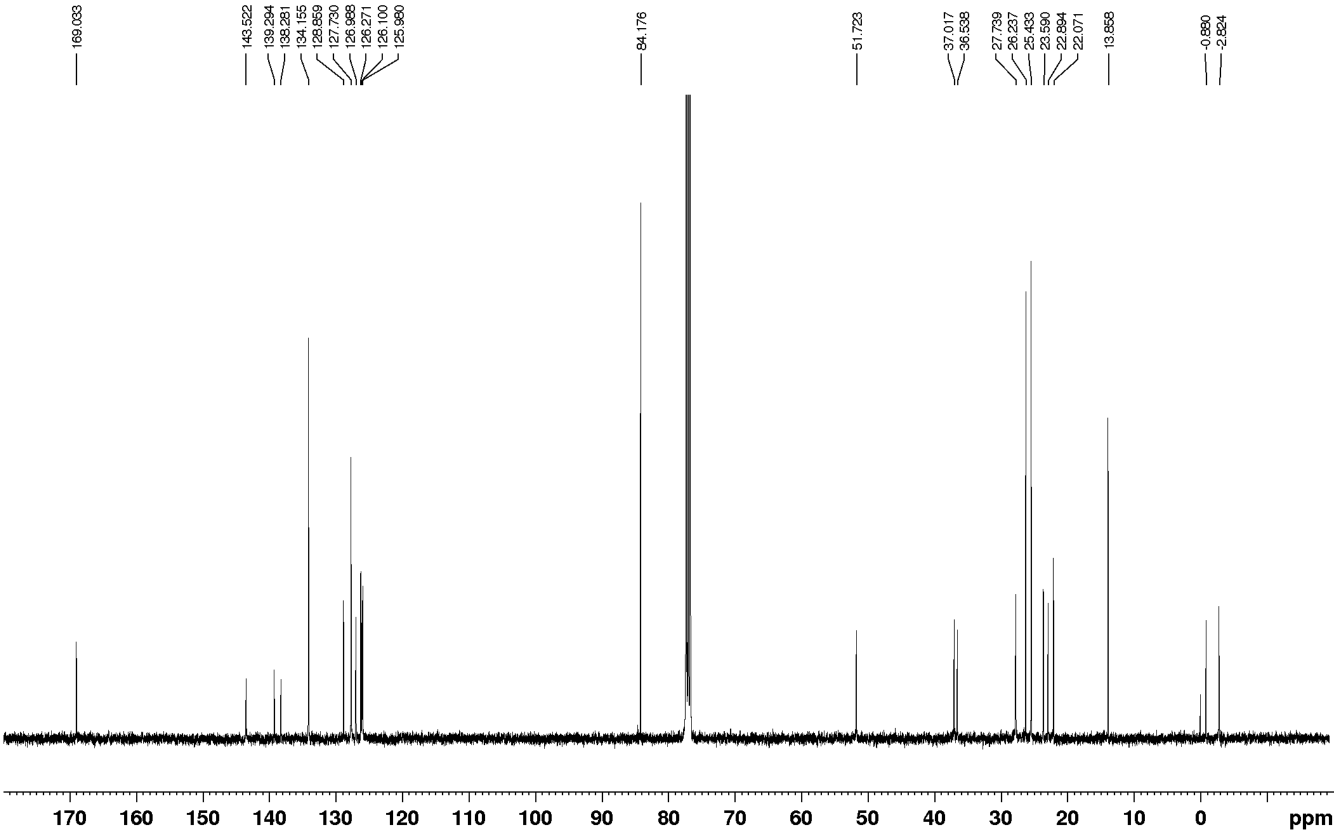

Supplementary Fig 40. **^{13}C NMR spectrum (100 MHz, $CDCl_3$, r.t.) of 3qa**.

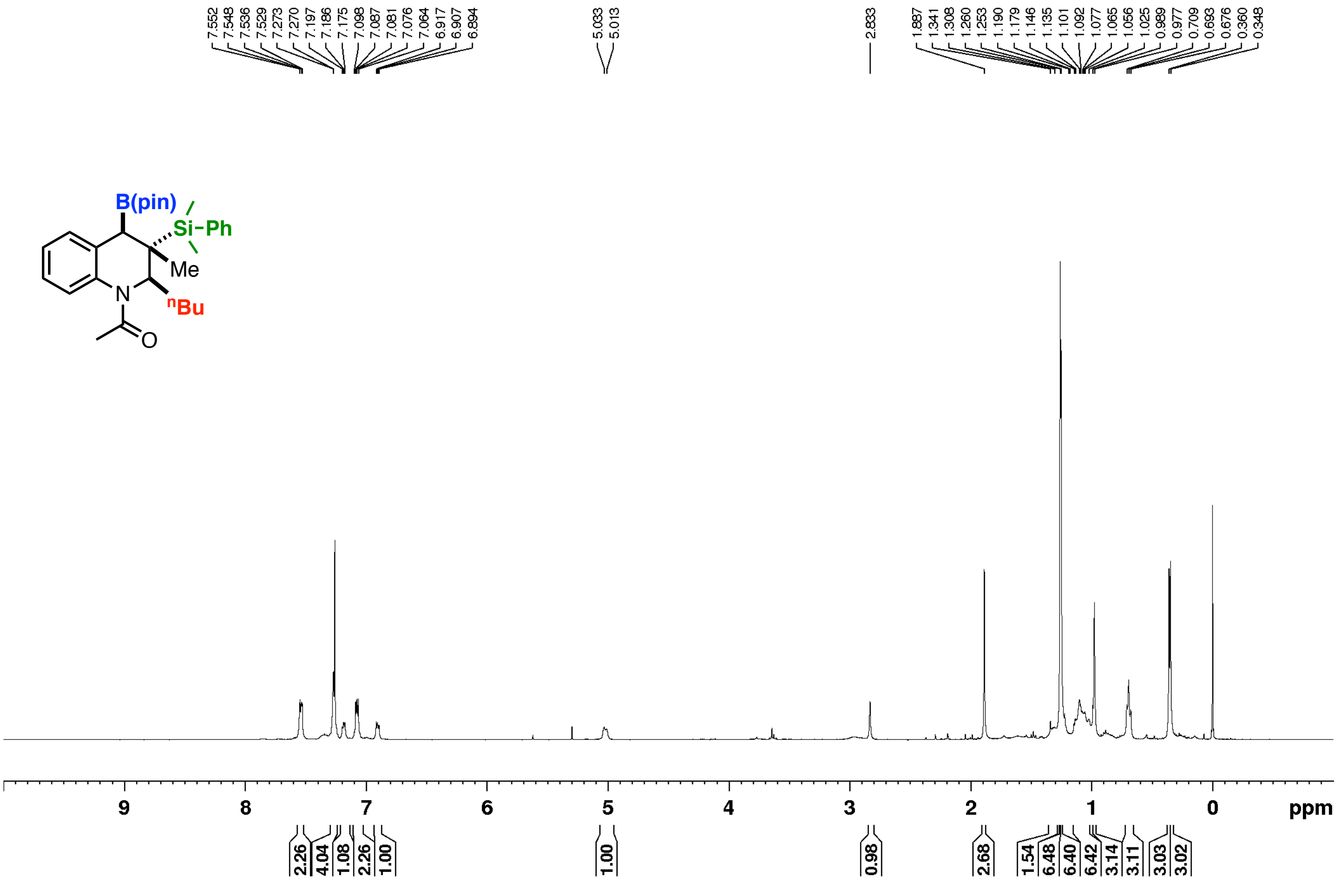

Supplementary Fig 41. ^{1}**H NMR spectrum (400 MHz,** $CDCl_3$**, r.t.) of 3ra**.

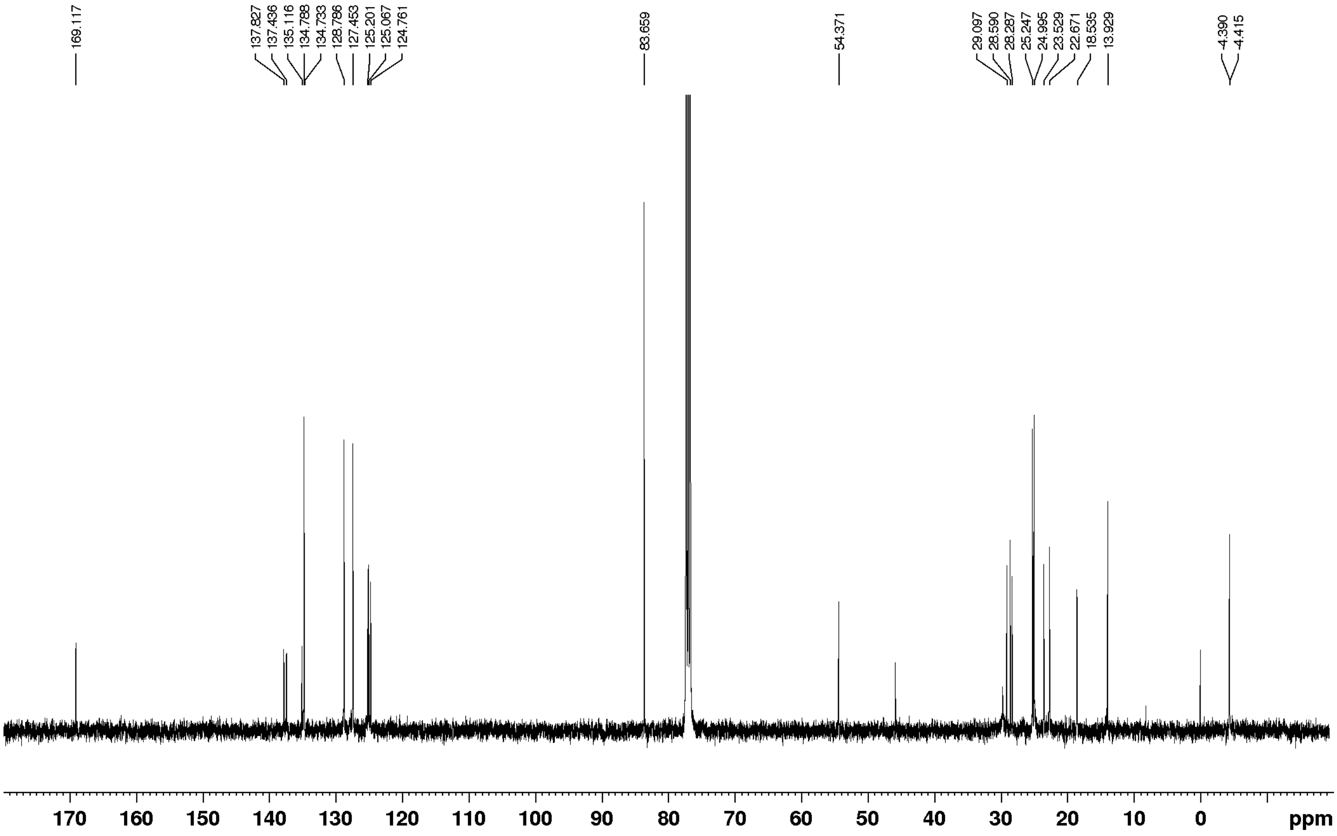

Supplementary Fig 42. ^{13}**C NMR spectrum (100 MHz,** $CDCl_3$**, r.t.) of 3ra**.

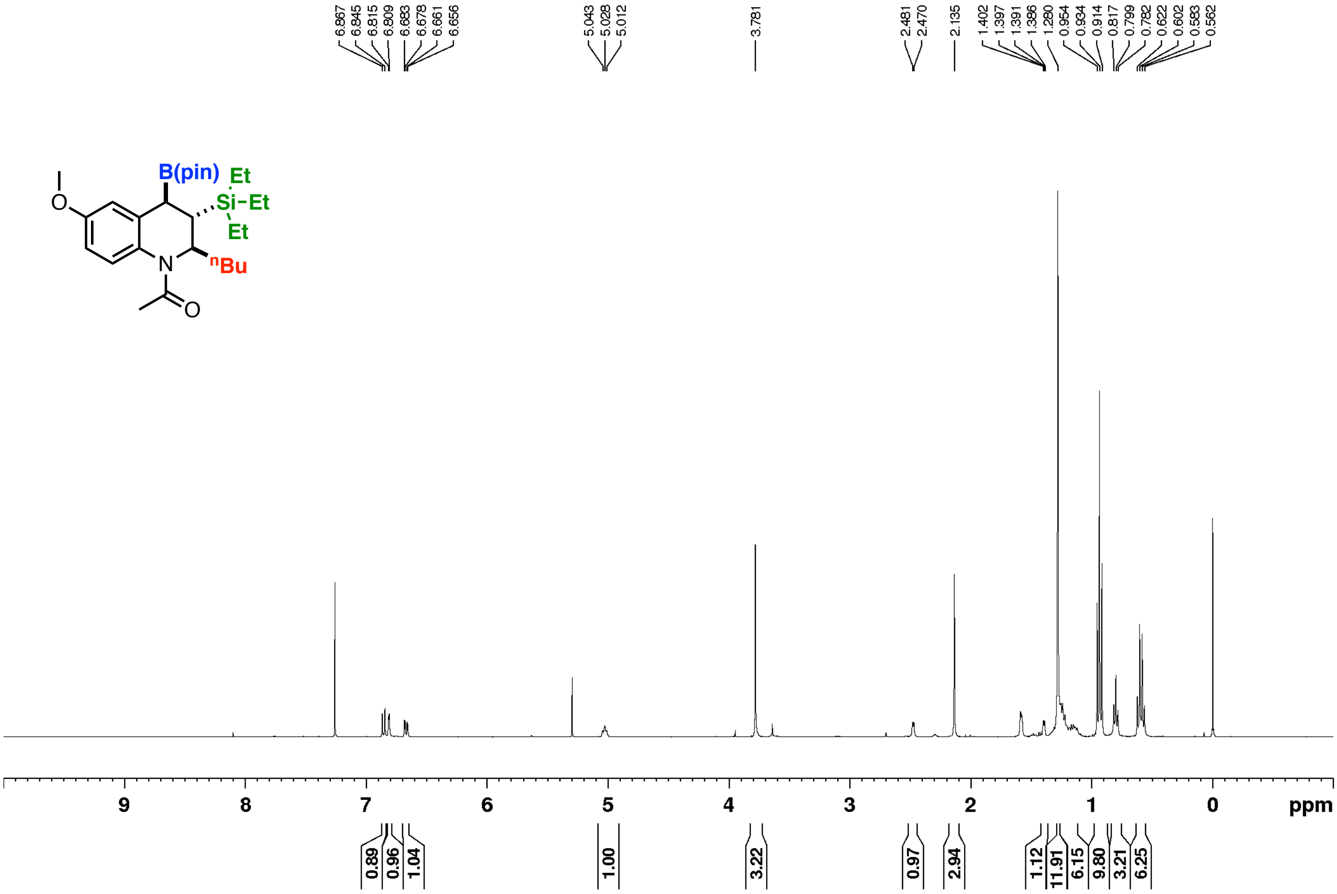

Supplementary Fig 43. ^{1}**H NMR spectrum (400 MHz,** $CDCl_3$**, r.t.) of 3ab**.

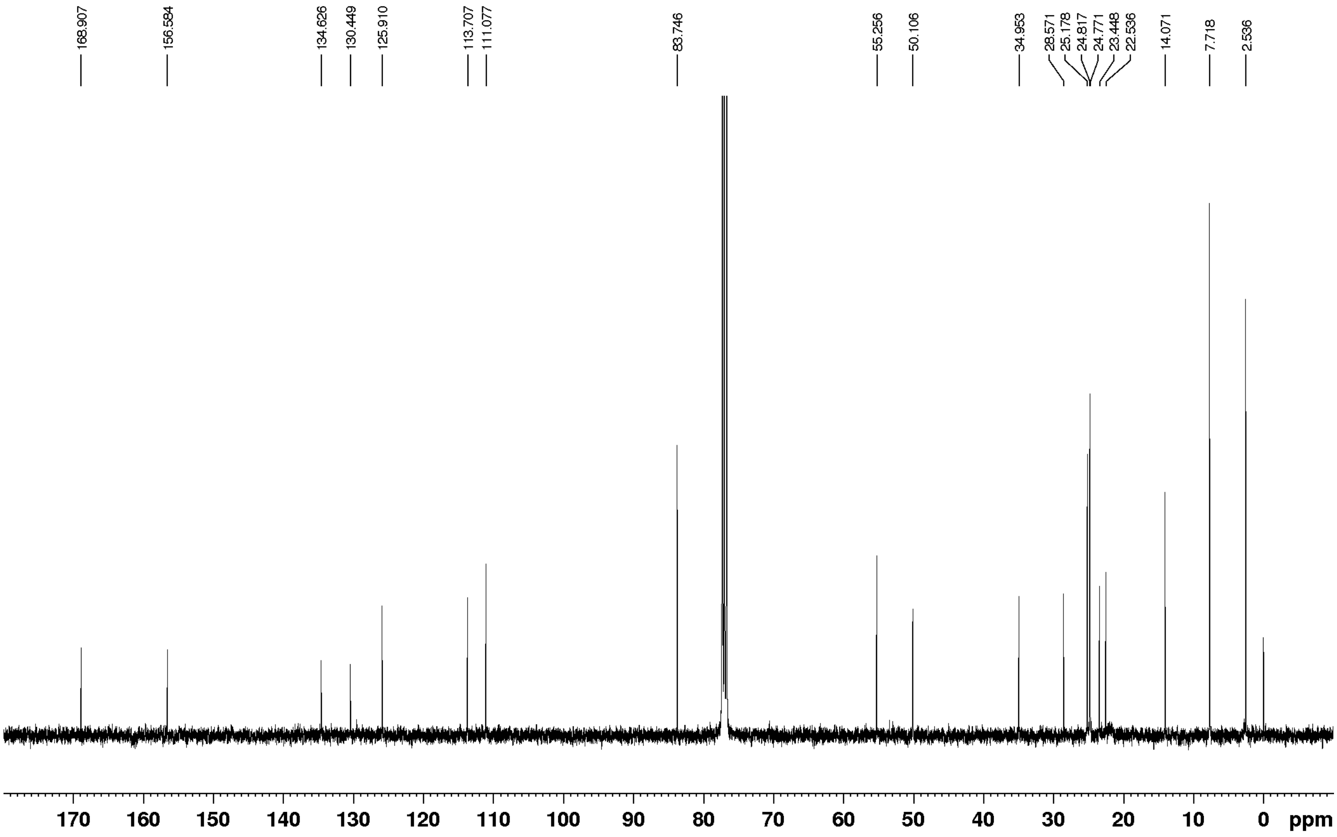

Supplementary Fig 44. ^{13}**C NMR spectrum (100 MHz,** $CDCl_3$**, r.t.) of 3ab**.

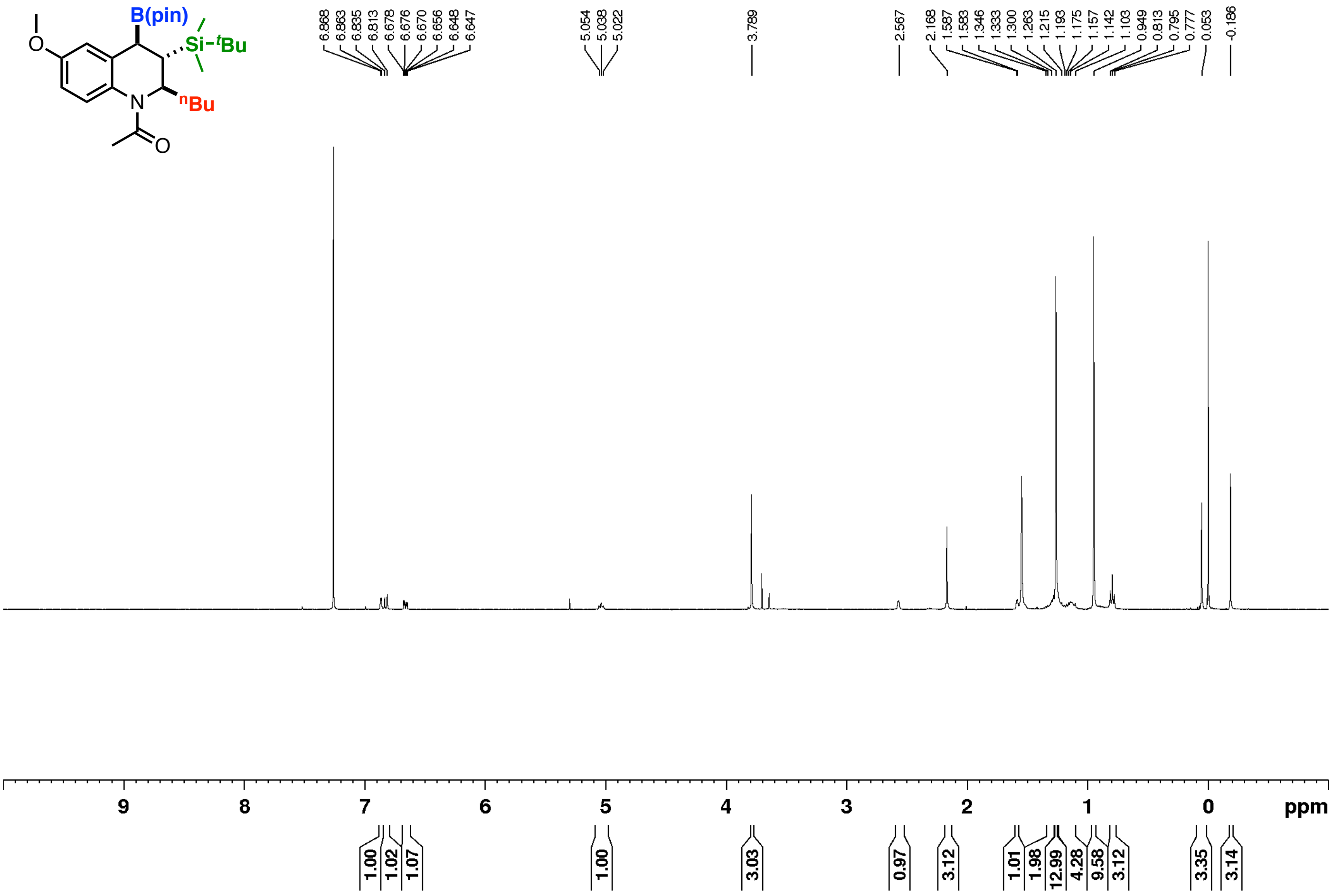

Supplementary Fig 45. **^{1}H NMR spectrum (400 MHz, $CDCl_3$, r.t.) of 3ac**.

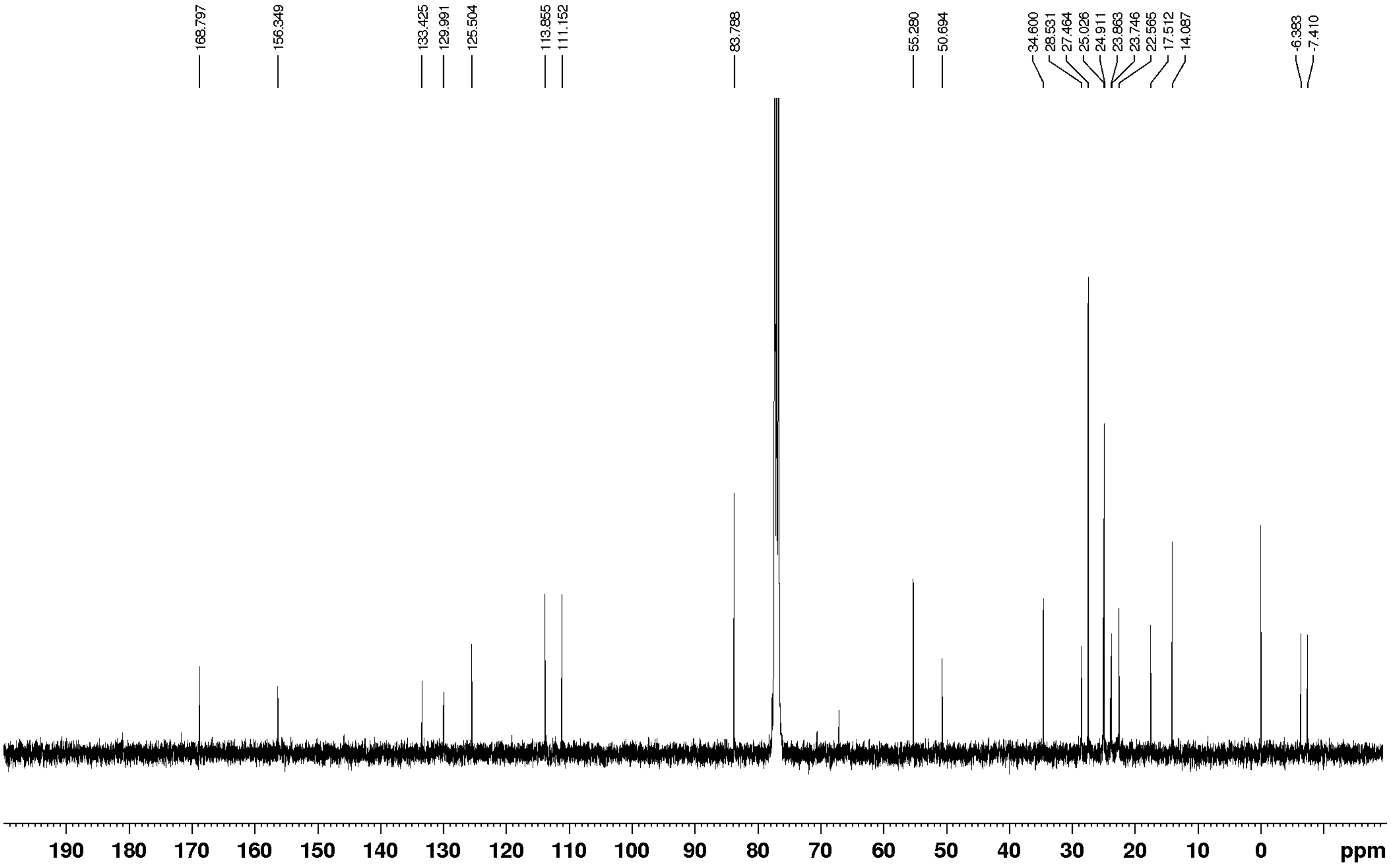

Supplementary Fig 46. **^{13}C NMR spectrum (100 MHz, $CDCl_3$, r.t.) of 3ac**.

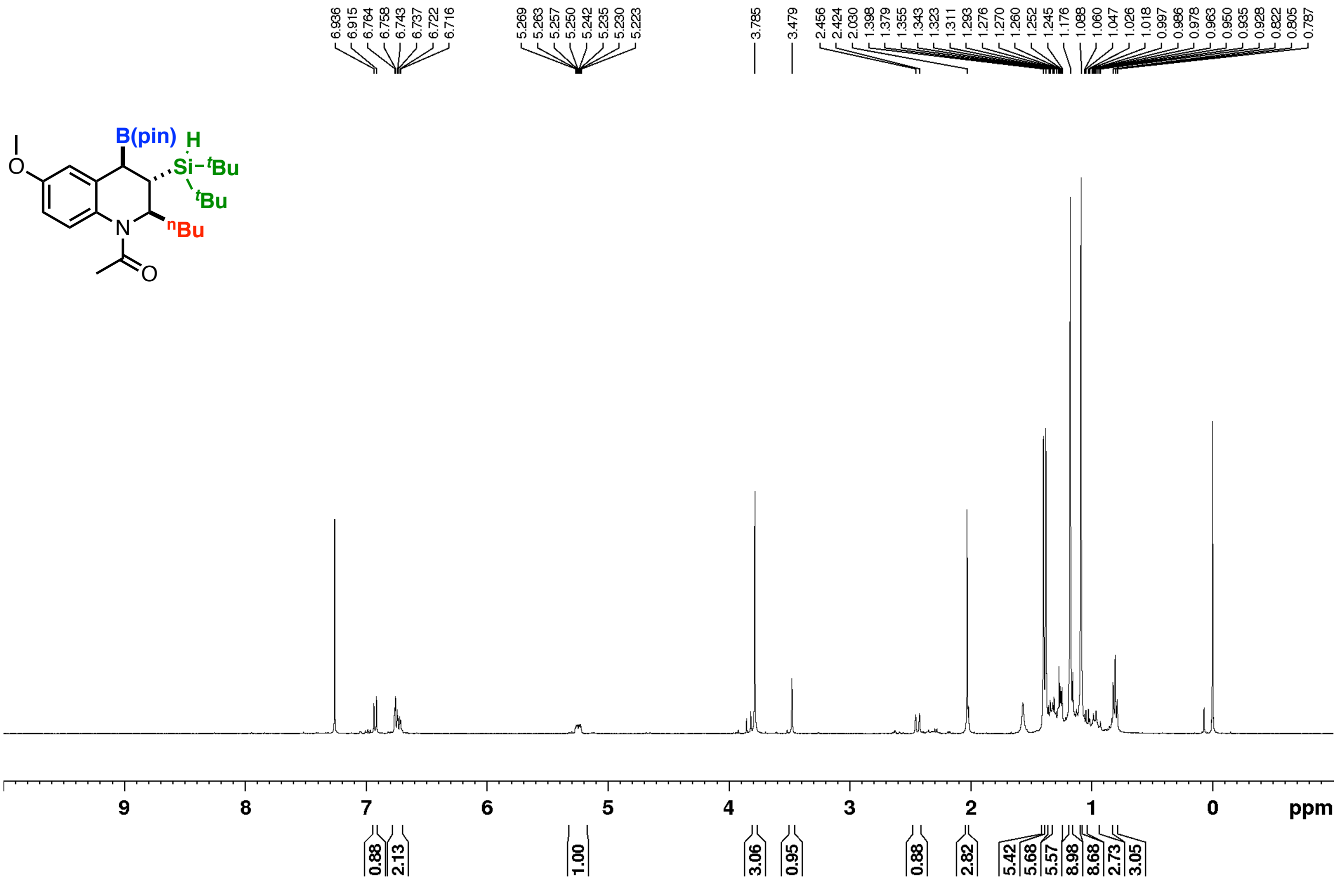

Supplementary Fig 47. **^{1}H NMR spectrum (400 MHz, $CDCl_3$, r.t.) of 3ad**.

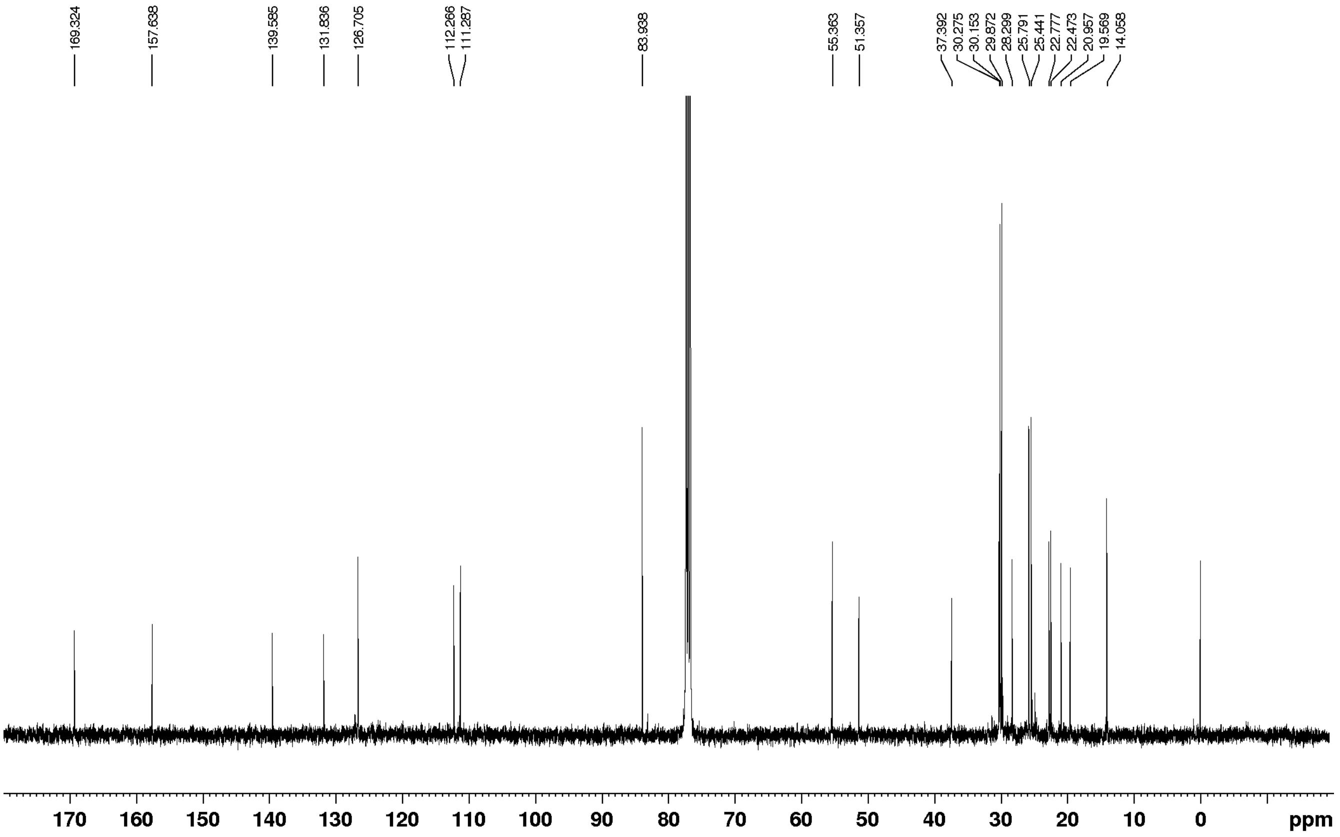

Supplementary Fig 48. **^{13}C NMR spectrum (100 MHz, $CDCl_3$, r.t.) of 3ad**.

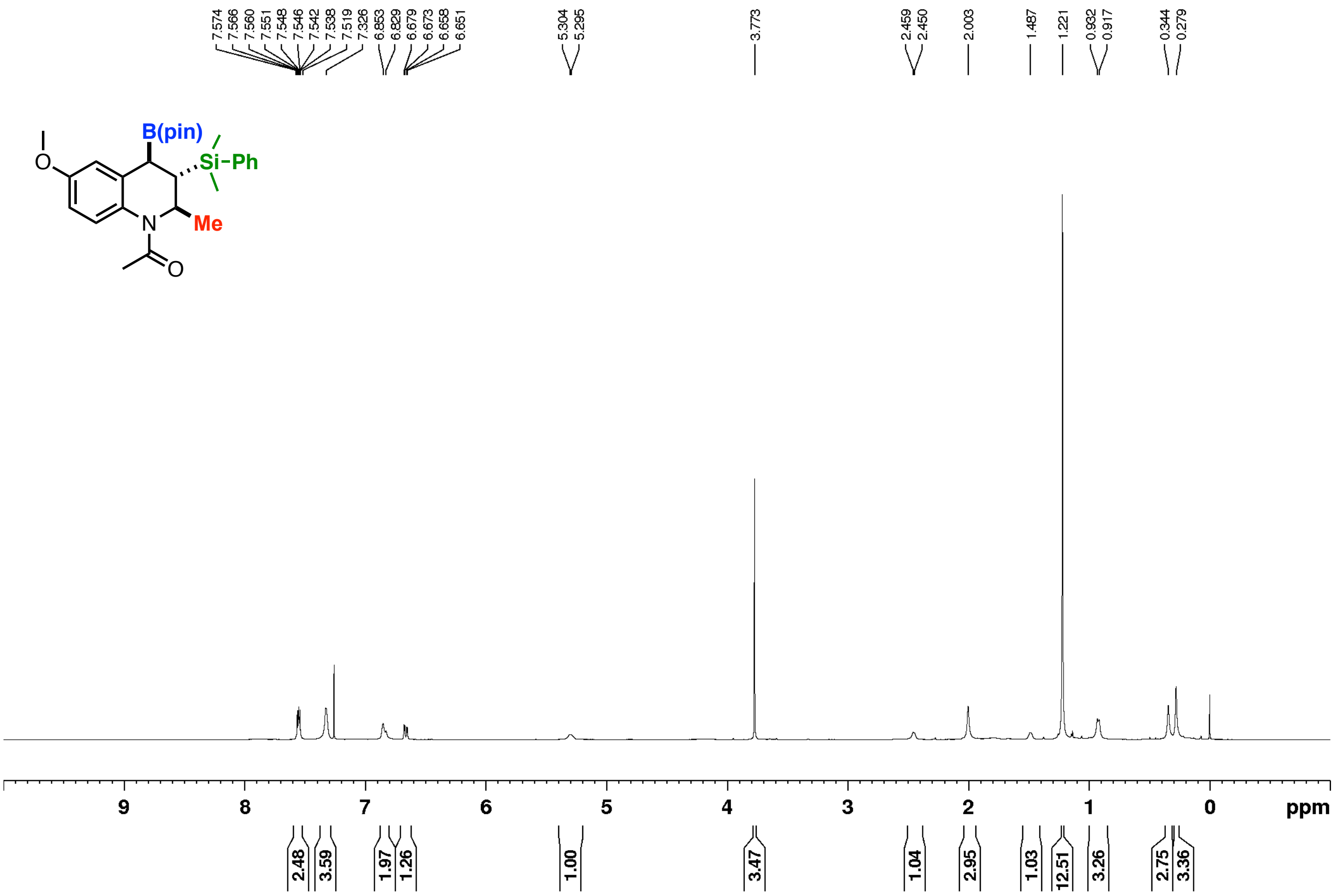

Supplementary Fig 49. **^{1}H NMR spectrum (400 MHz, $CDCl_3$, r.t.) of 3ae**.

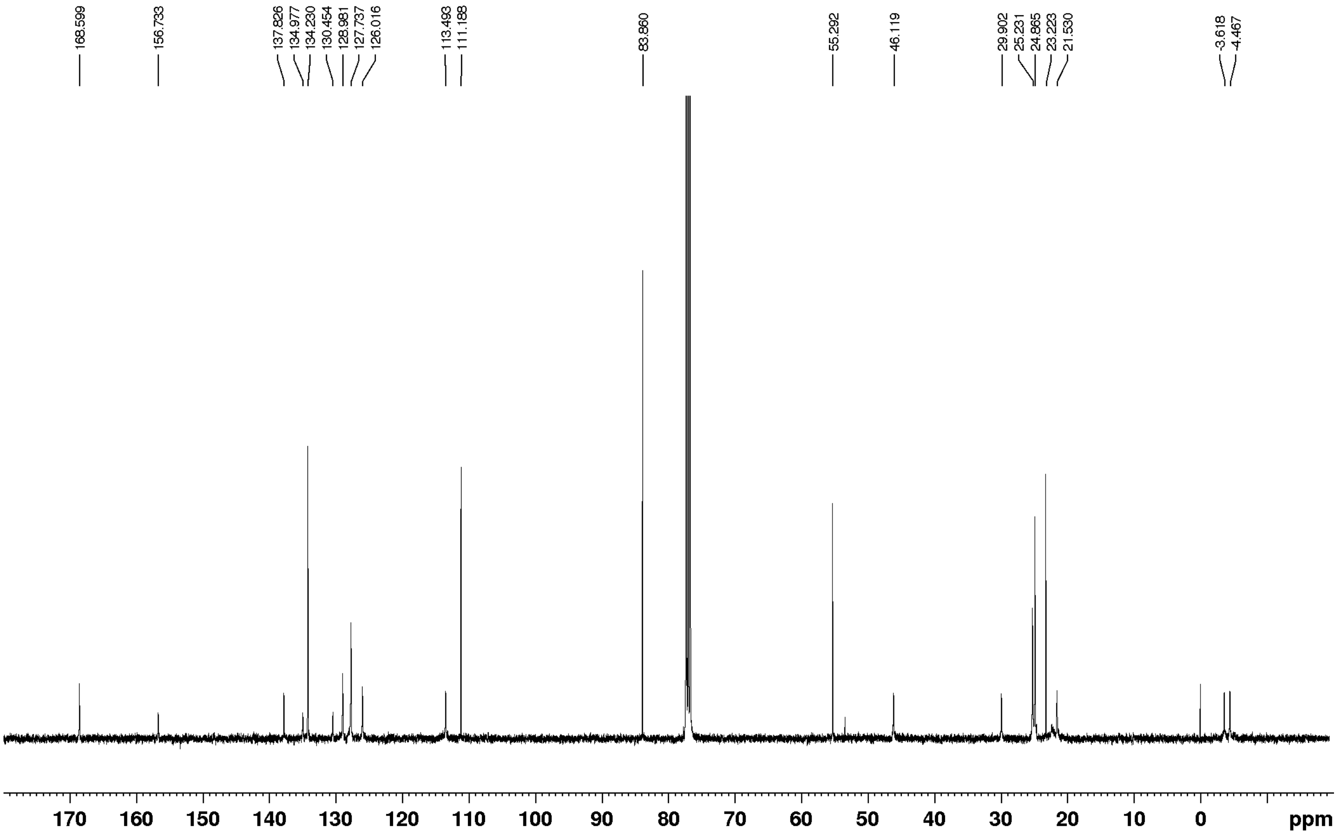

Supplementary Fig 50. **^{13}C NMR spectrum (100 MHz, $CDCl_3$, r.t.) of 3ae**.

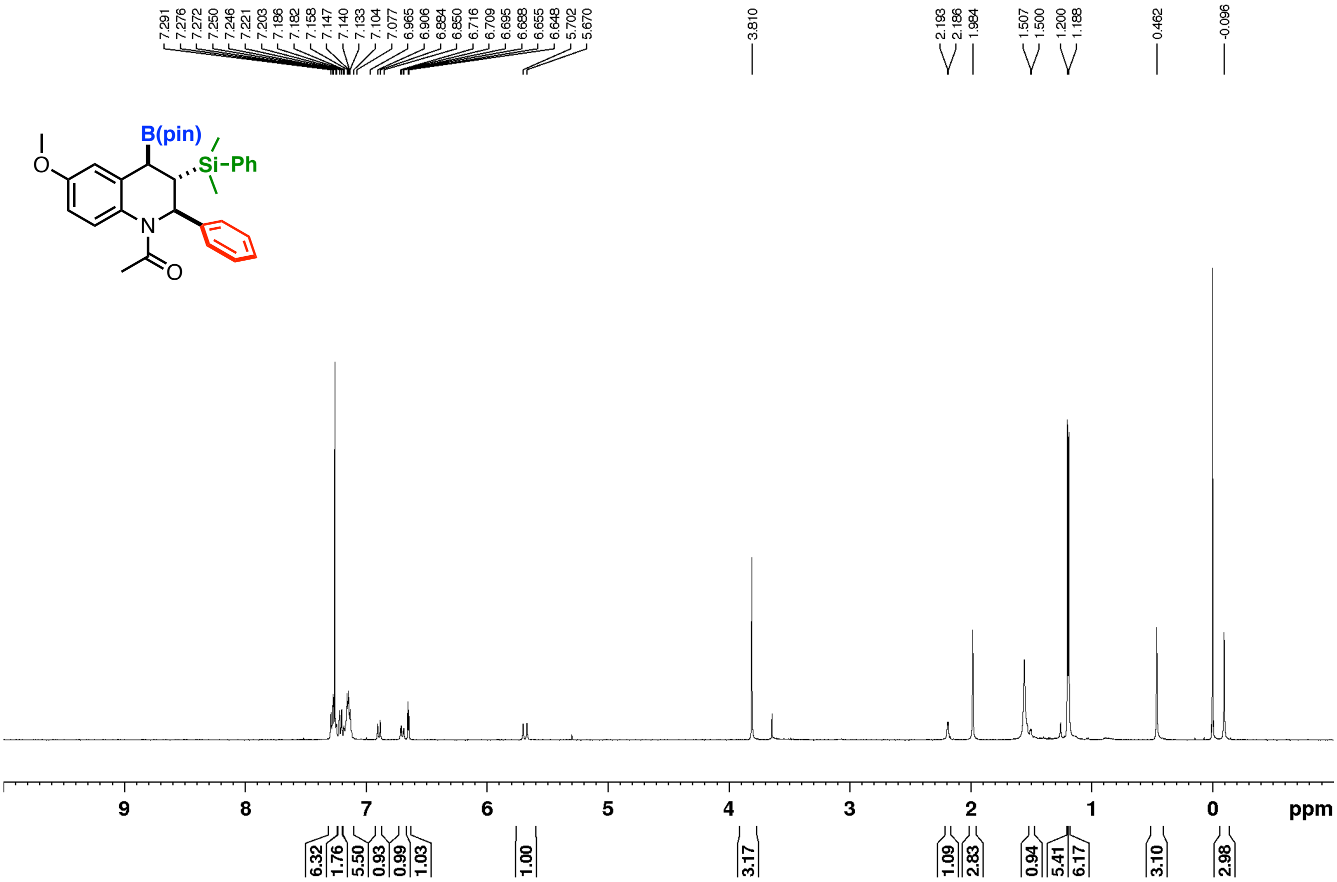

Supplementary Fig 51. ^{1}H **NMR spectrum (400 MHz,** $CDCl_3$**, r.t.) of 3af**.

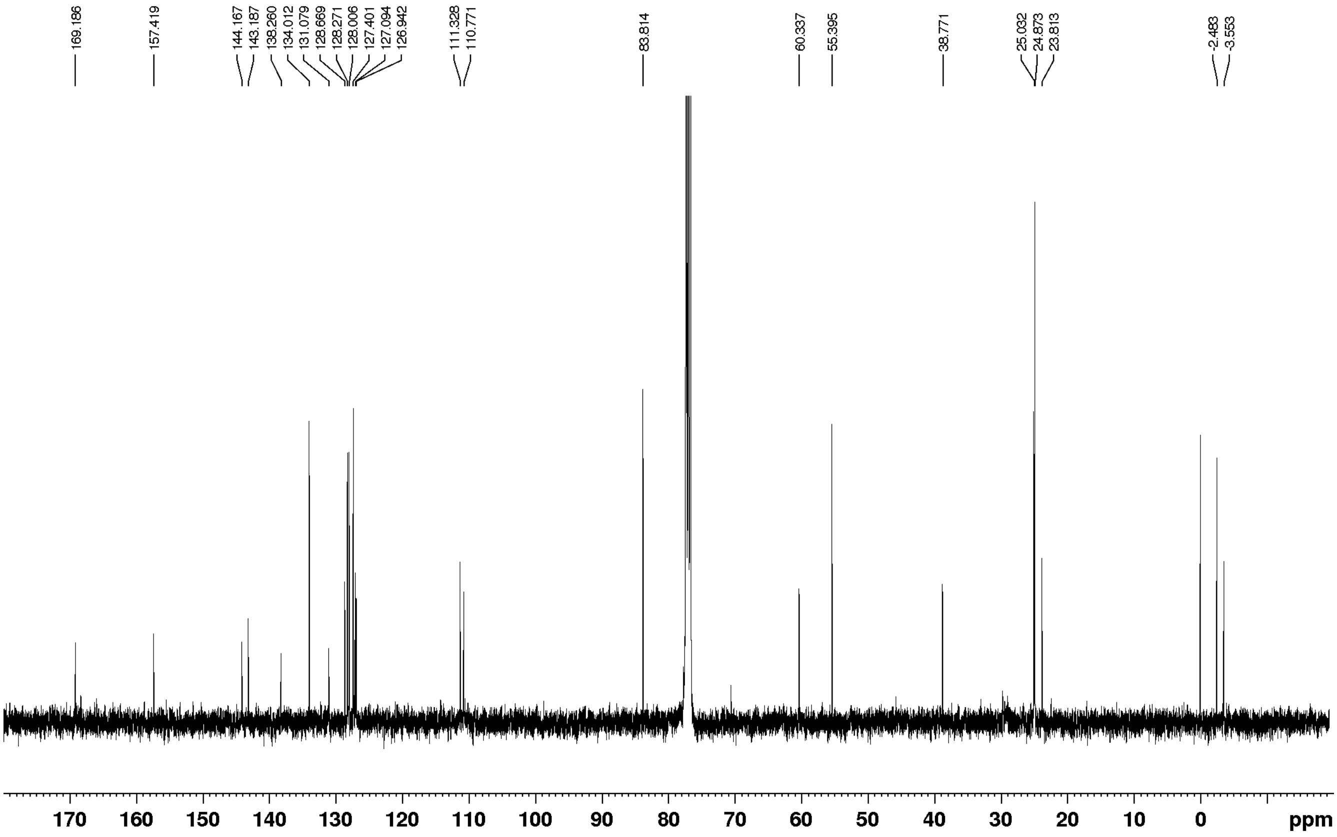

Supplementary Fig 52. ^{13}C **NMR spectrum (100 MHz,** $CDCl_3$**, r.t.) of 3af**.

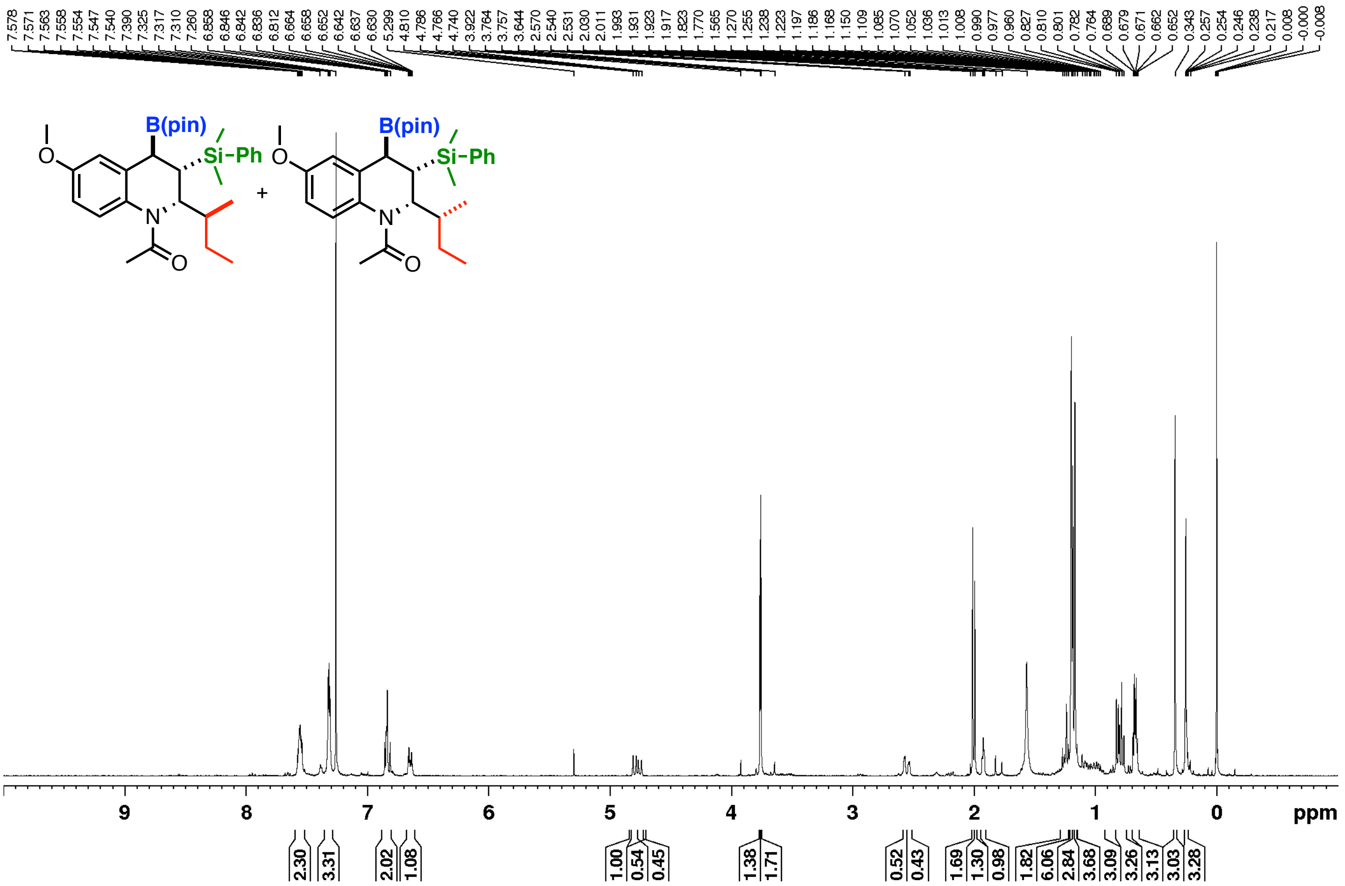

Supplementary Fig 53. ^{1}H NMR spectrum (400 MHz, $CDCl_3$, r.t.) of 3ag.

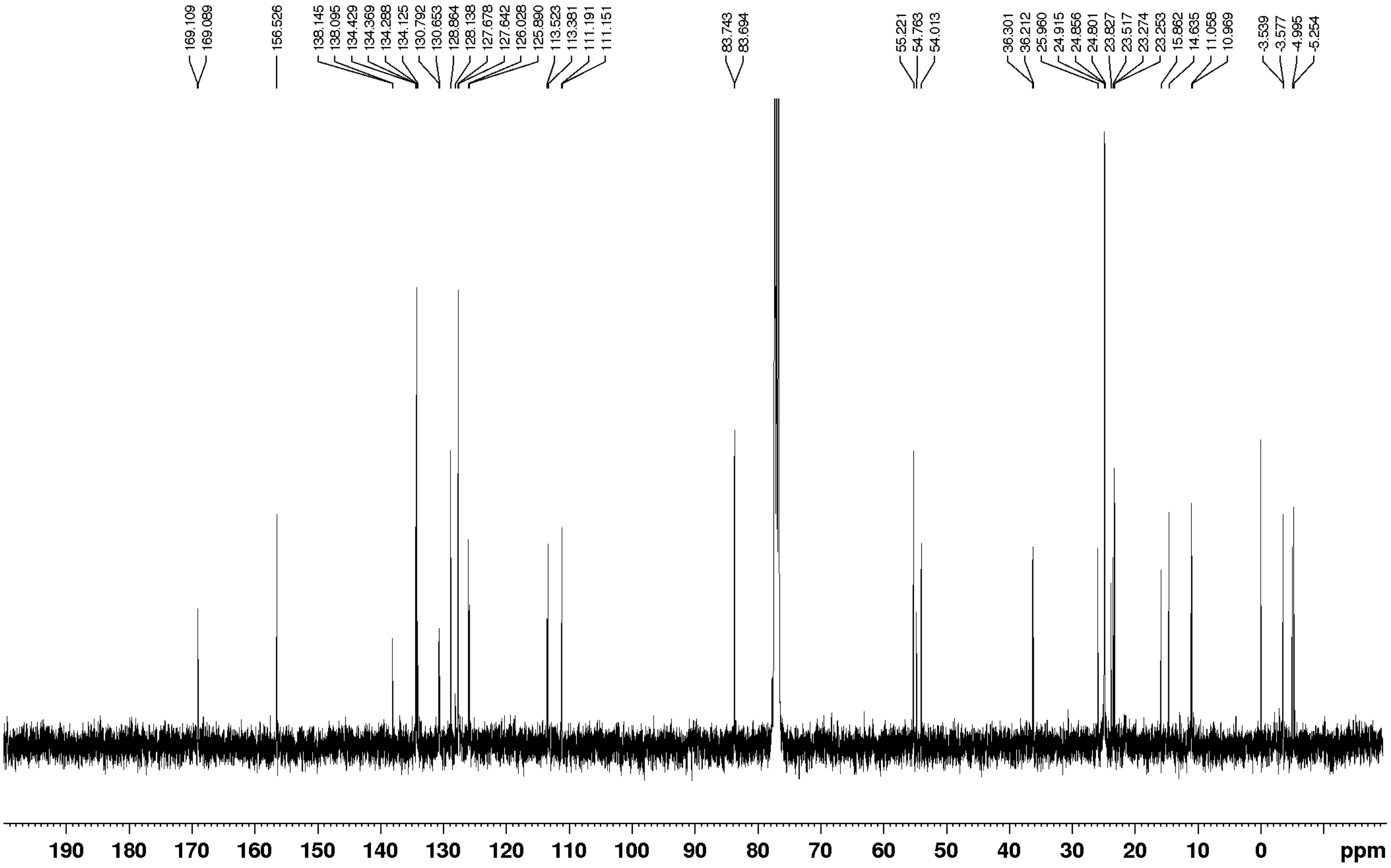

Supplementary Fig 54. ^{13}C NMR spectrum (100 MHz, $CDCl_3$, r.t.) of 3ag.

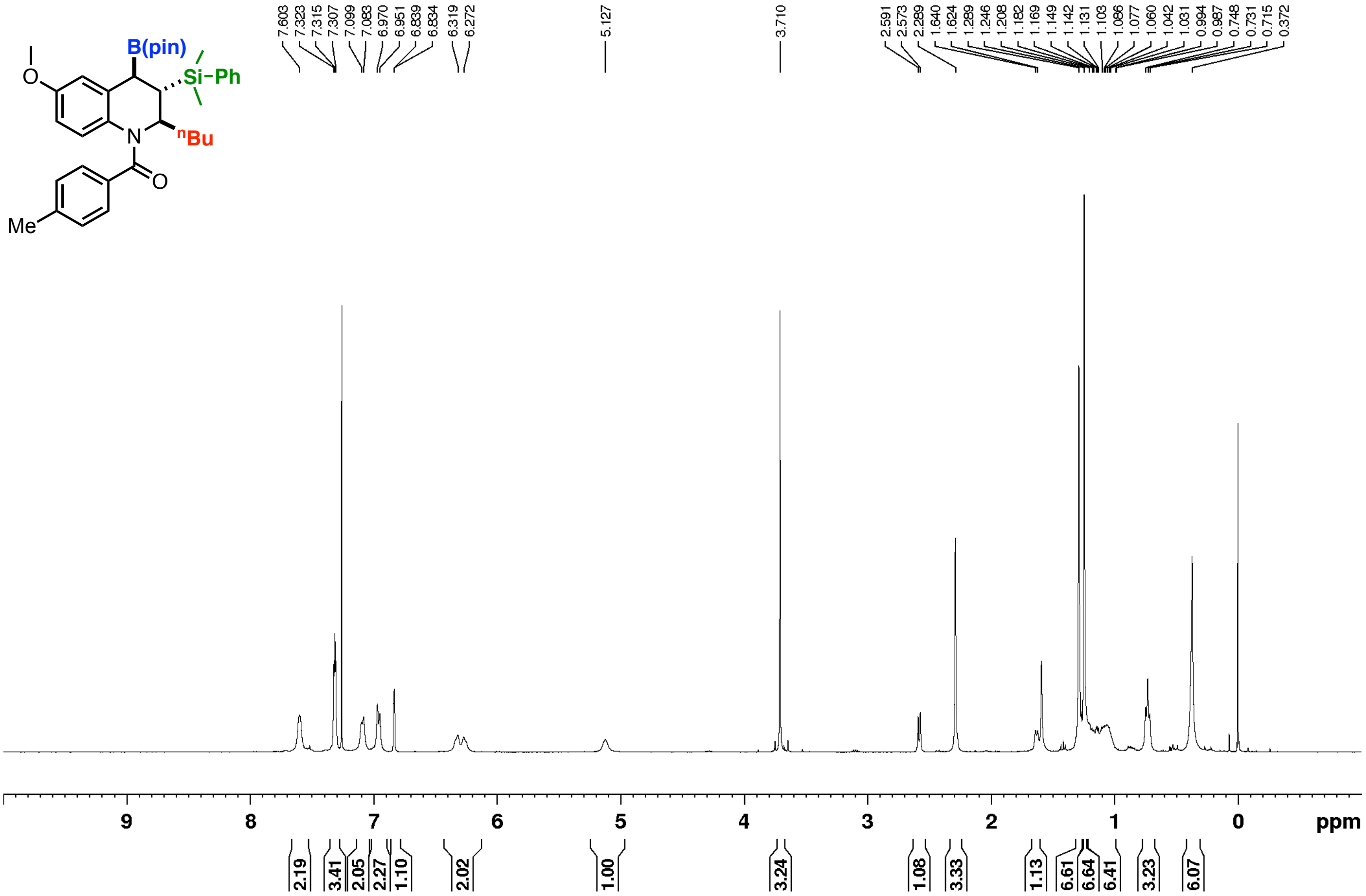

Supplementary Fig 55. ^{1}H NMR spectrum (400 MHz, $CDCl_3$, r.t.) of 3ah.

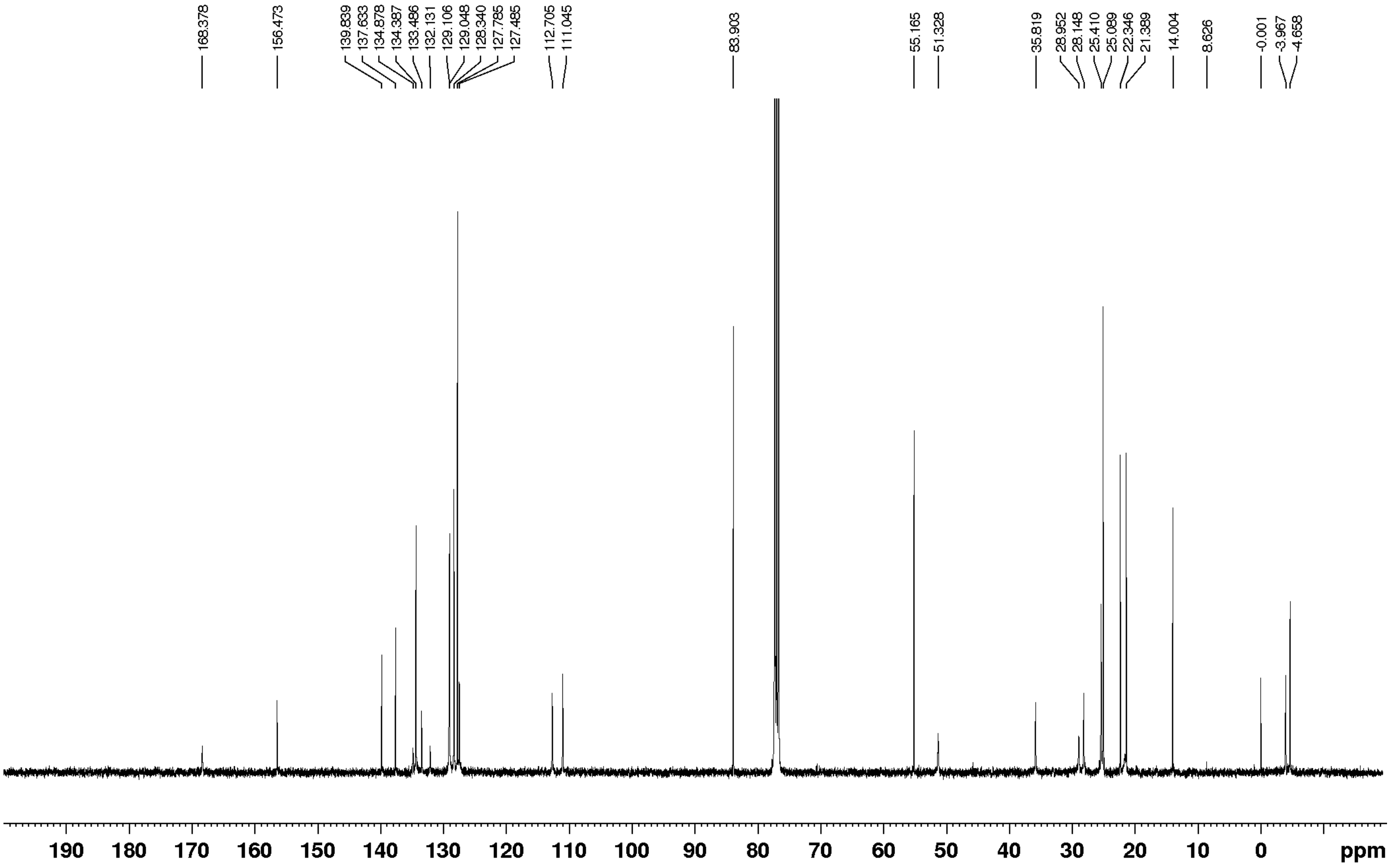

Supplementary Fig 56. ^{13}C NMR spectrum (100 MHz, $CDCl_3$, r.t.) of 3ah.

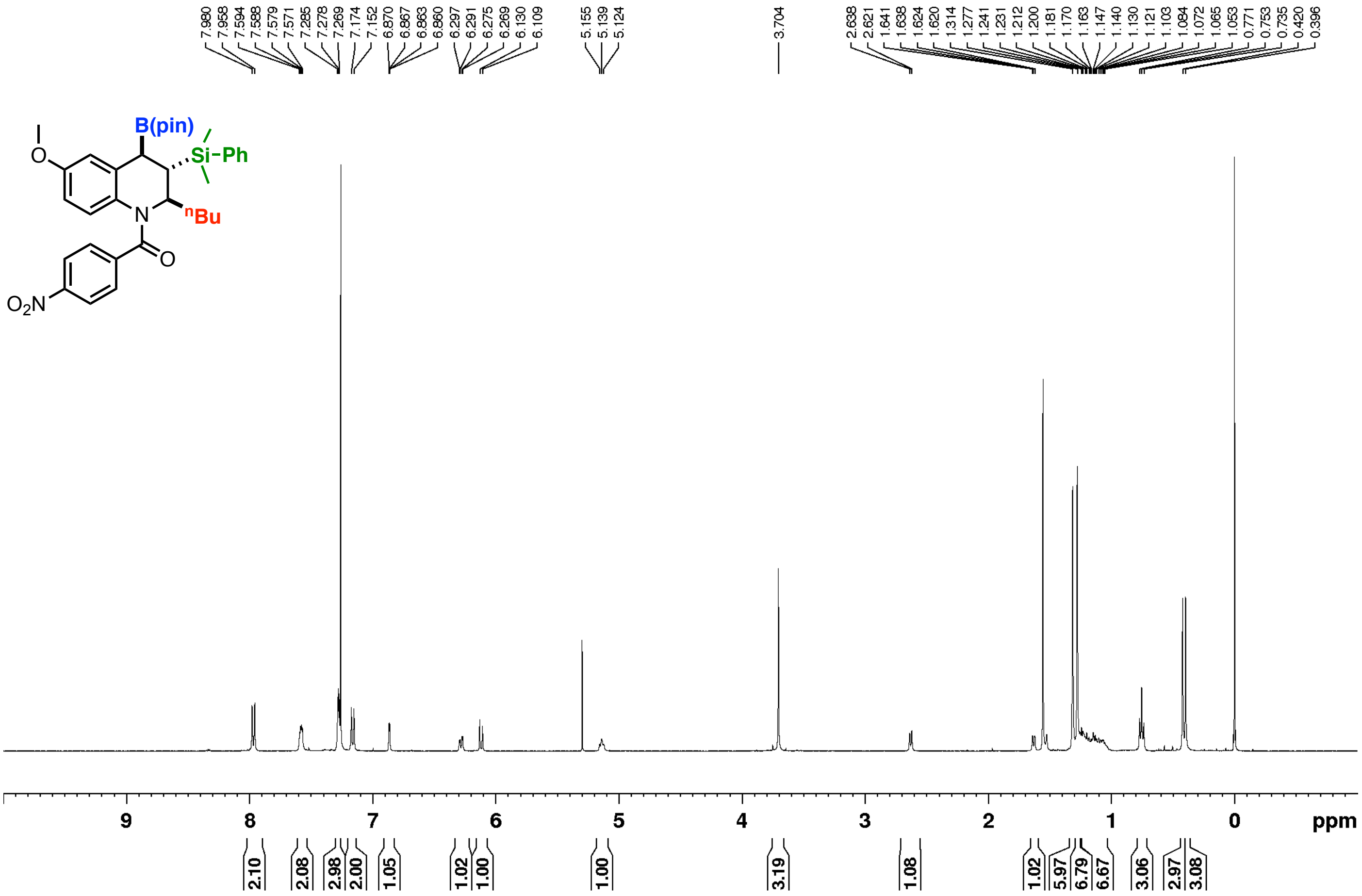

Supplementary Fig 57. ^{1}H **NMR spectrum (400 MHz,** $CDCl_3$**, r.t.) of 3ai**.

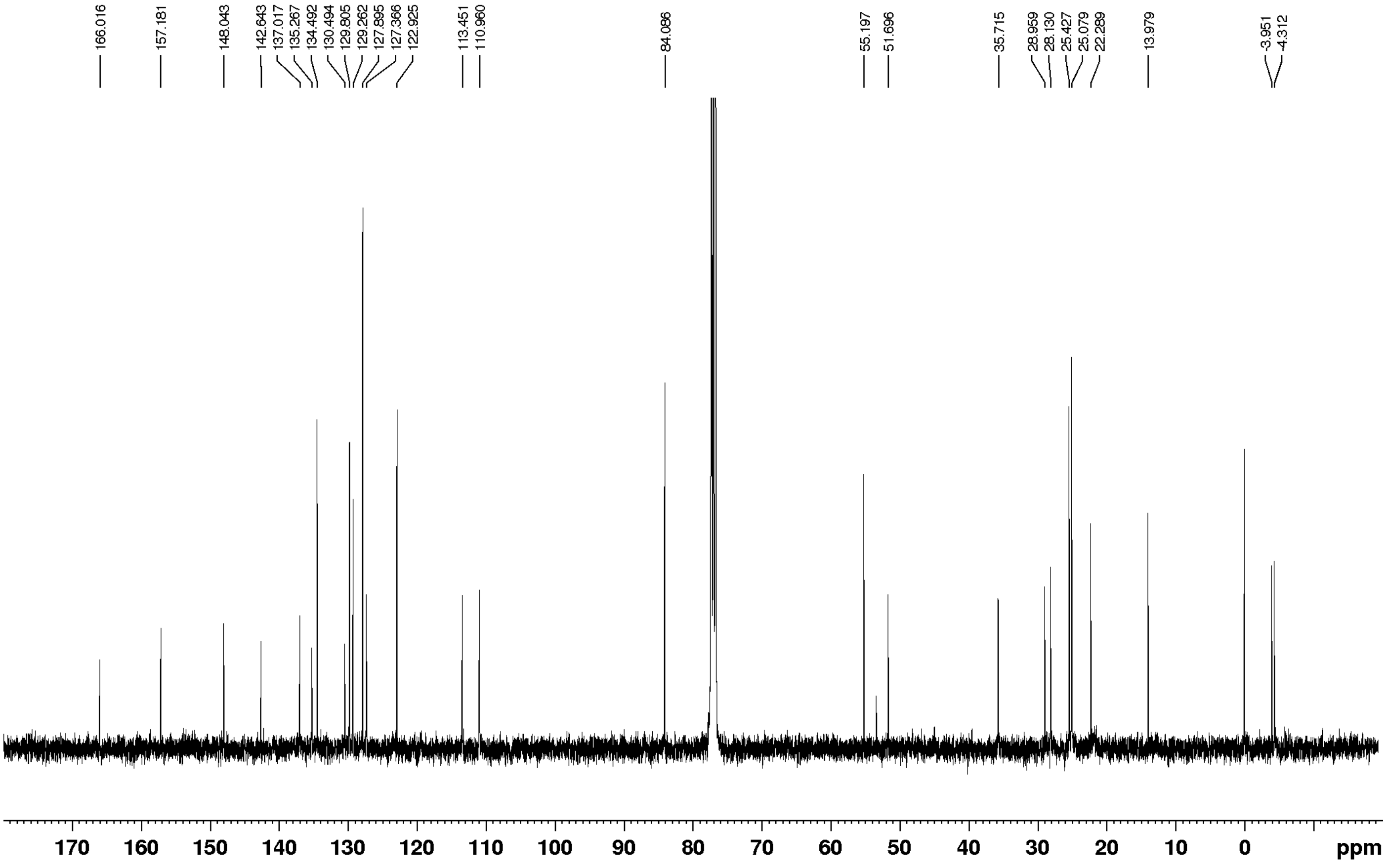

Supplementary Fig 58. ^{13}C **NMR spectrum (100 MHz,** $CDCl_3$**, r.t.) of 3ai**.

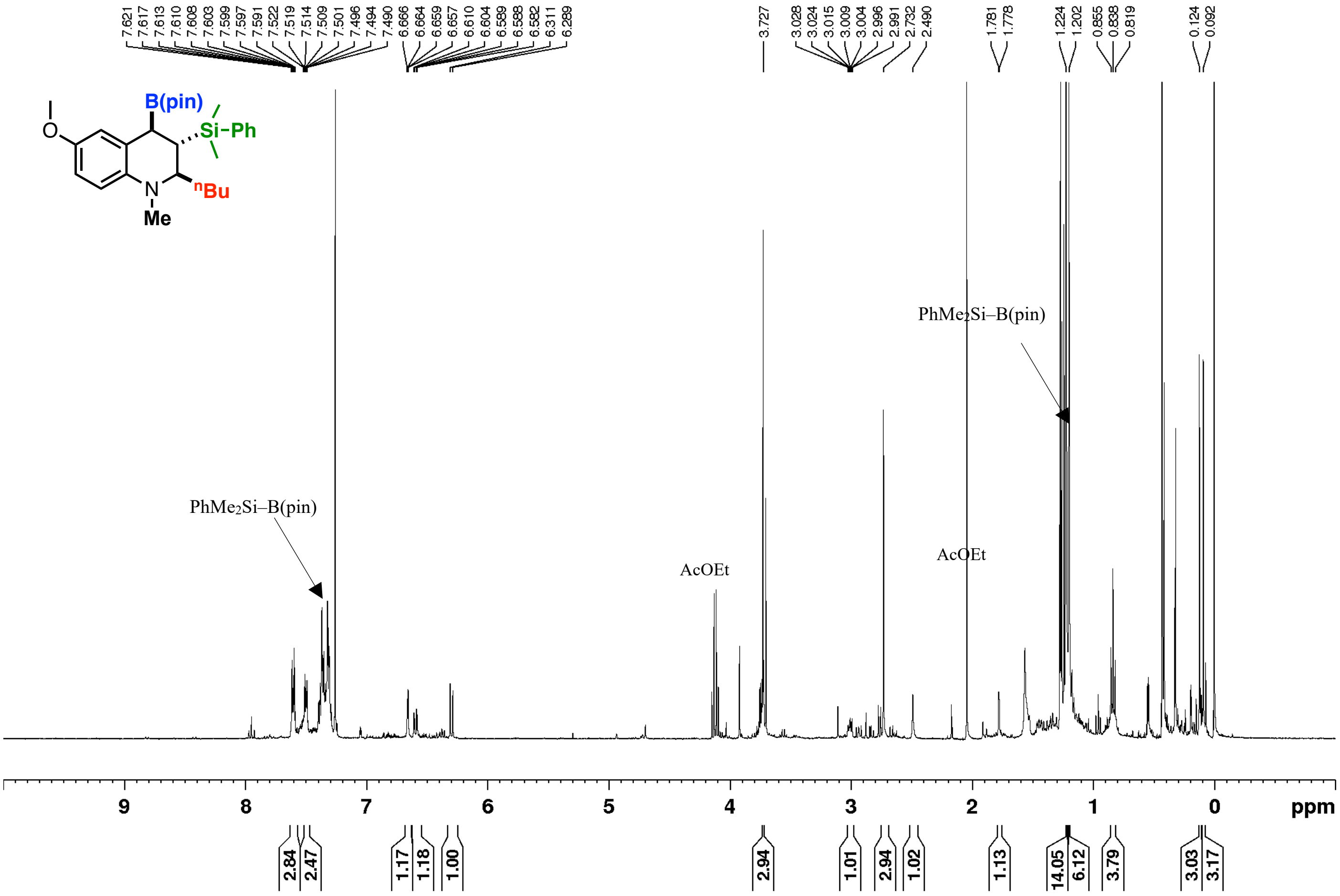

Supplementary Fig 59. ^{1}H NMR spectrum (400 MHz, $CDCl_3$, r.t.) of the crude mixture of 3ak.

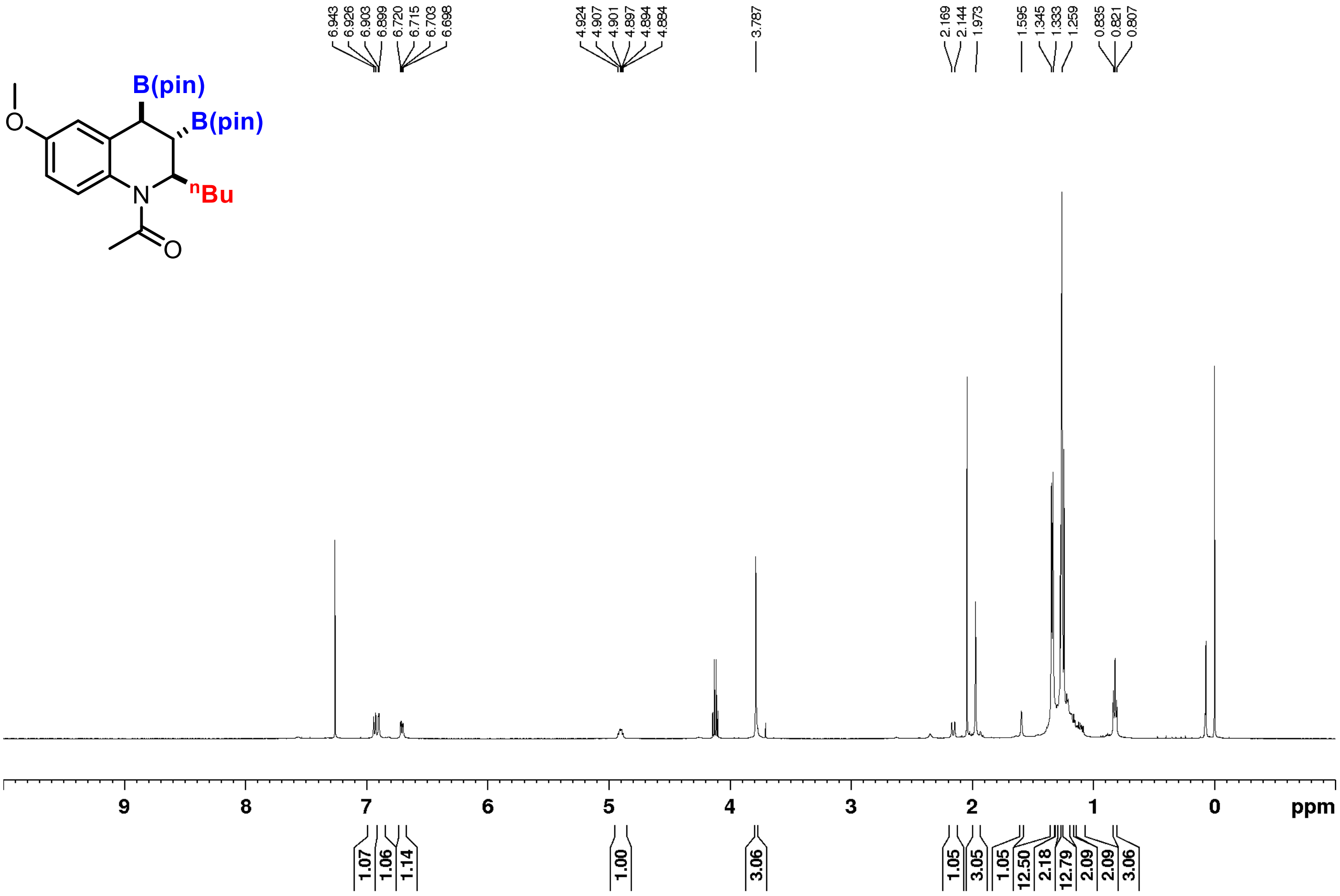

Supplementary Fig 60. ^{1}H NMR spectrum (500 MHz, $CDCl_3$, r.t.) of 5a-1.

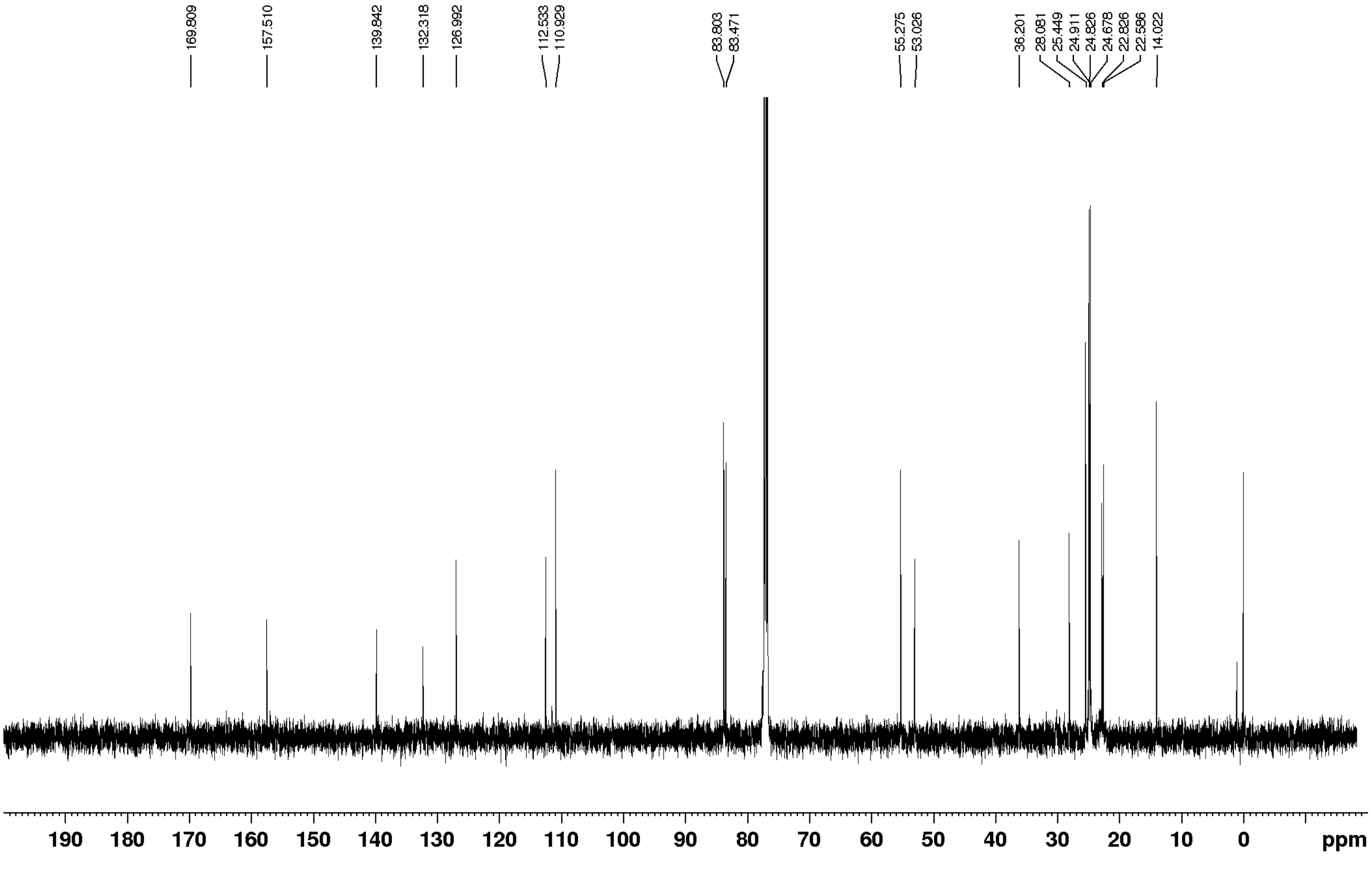

Supplementary Fig 61. ^{13}C NMR spectrum (125 MHz, $CDCl_3$, r.t.) of 5a-1.

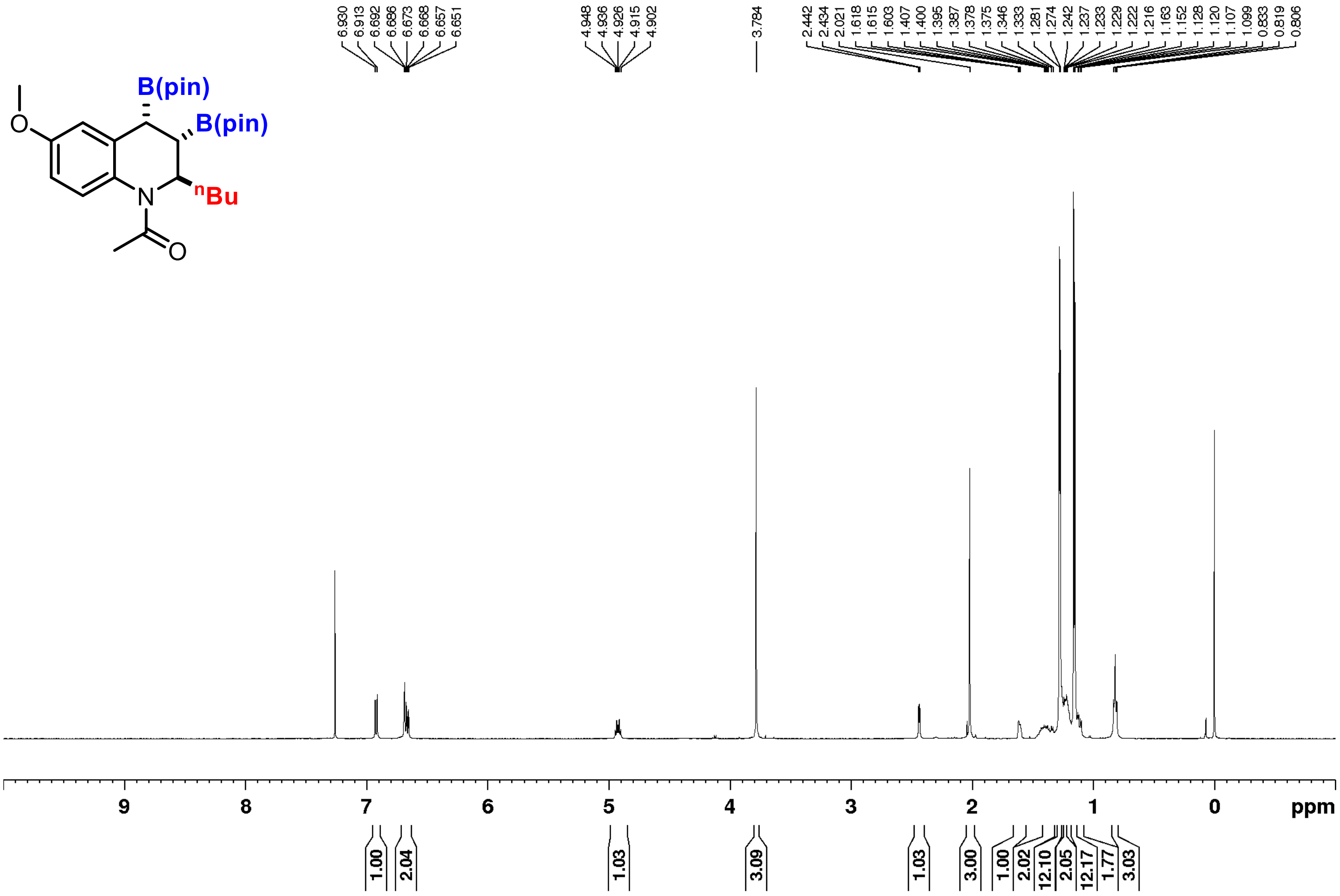

Supplementary Fig 62. ^{1}H NMR spectrum (500 MHz, $CDCl_3$, r.t.) of 5a-2.

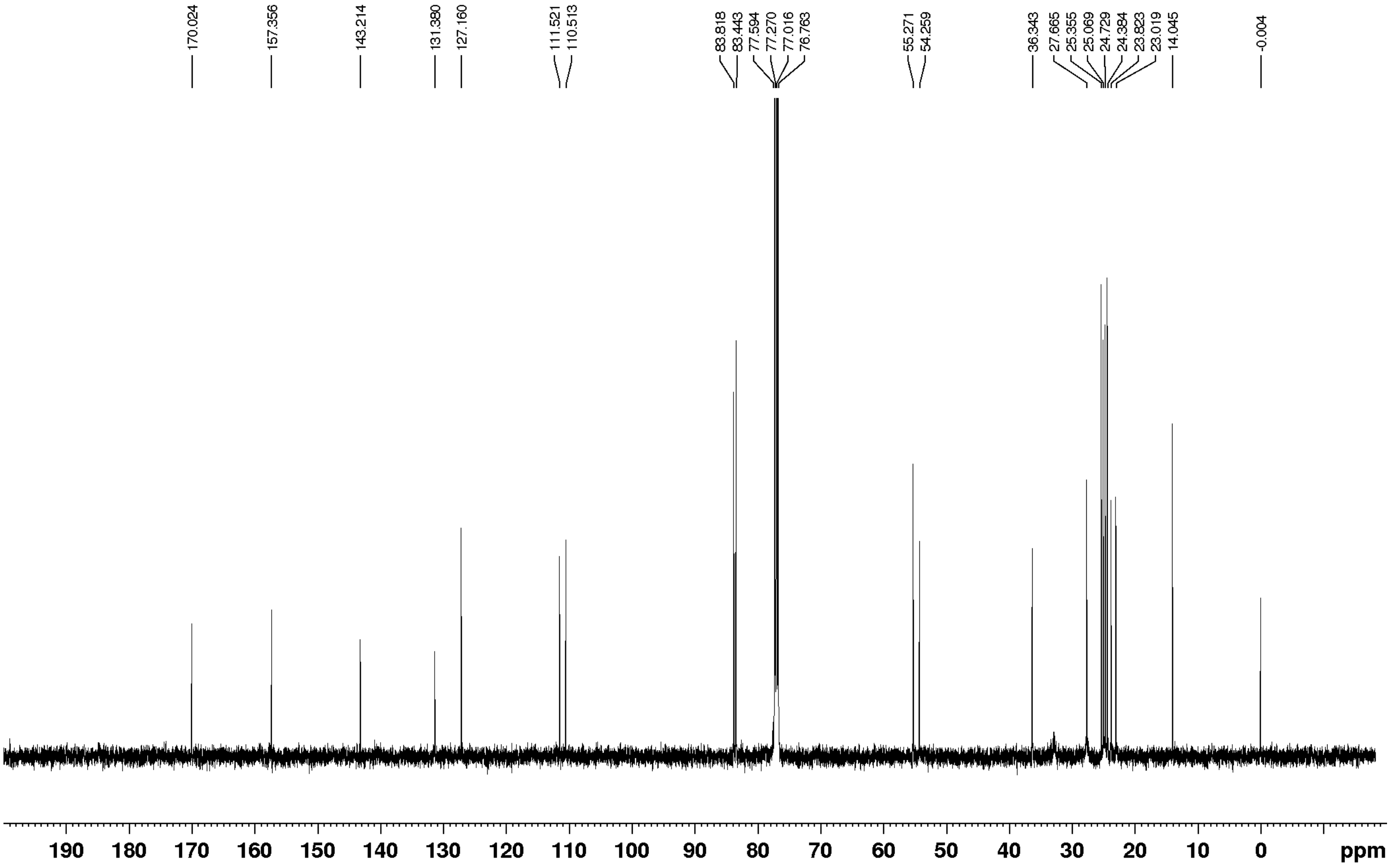

Supplementary Fig 63. ^{13}C NMR spectrum (125 MHz, $CDCl_3$, r.t.) of 5a-2.

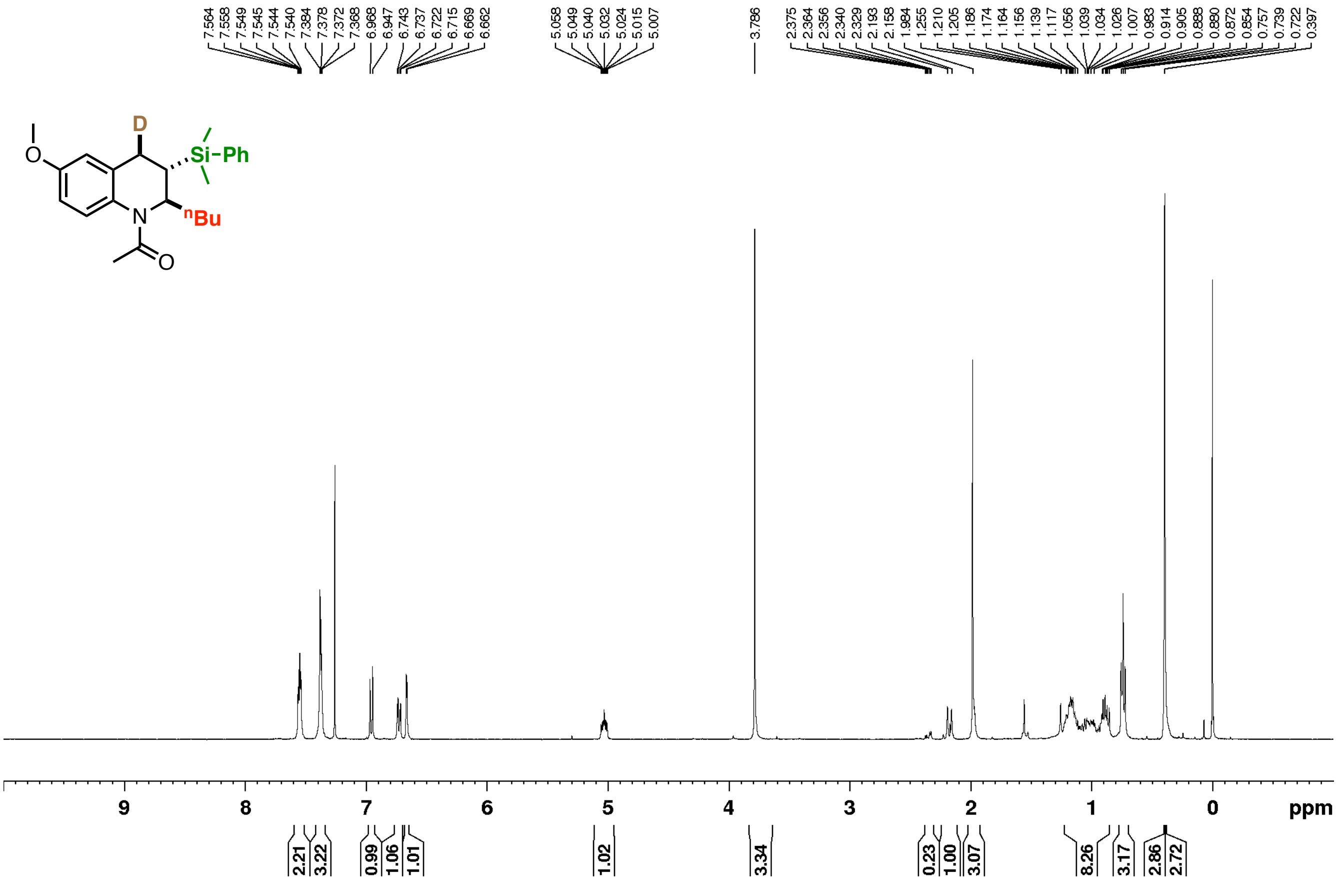

Supplementary Fig 64. **^{1}H NMR spectrum (400 MHz, $CDCl_3$, r.t.) of 7-D.**

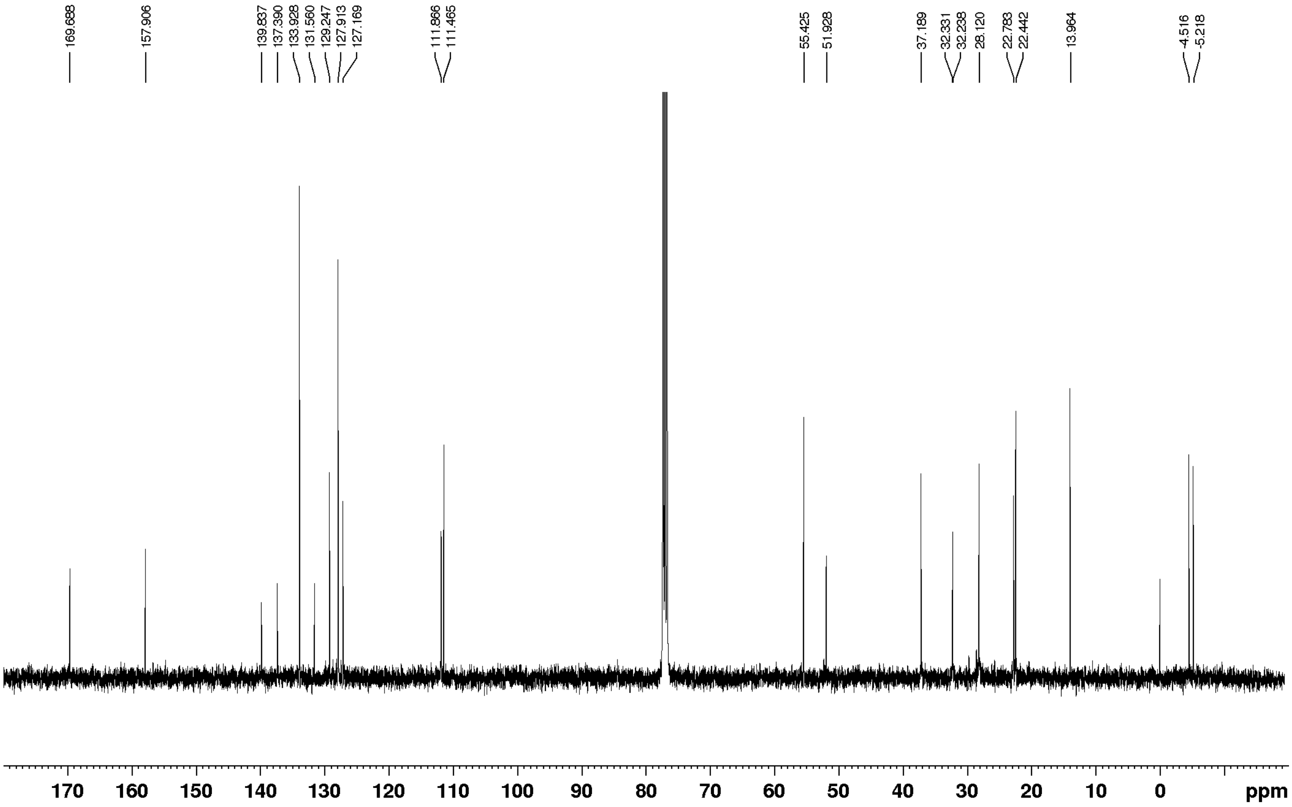

Supplementary Fig 65. **^{13}C NMR spectrum (100 MHz, $CDCl_3$, r.t.) of 7-D.**

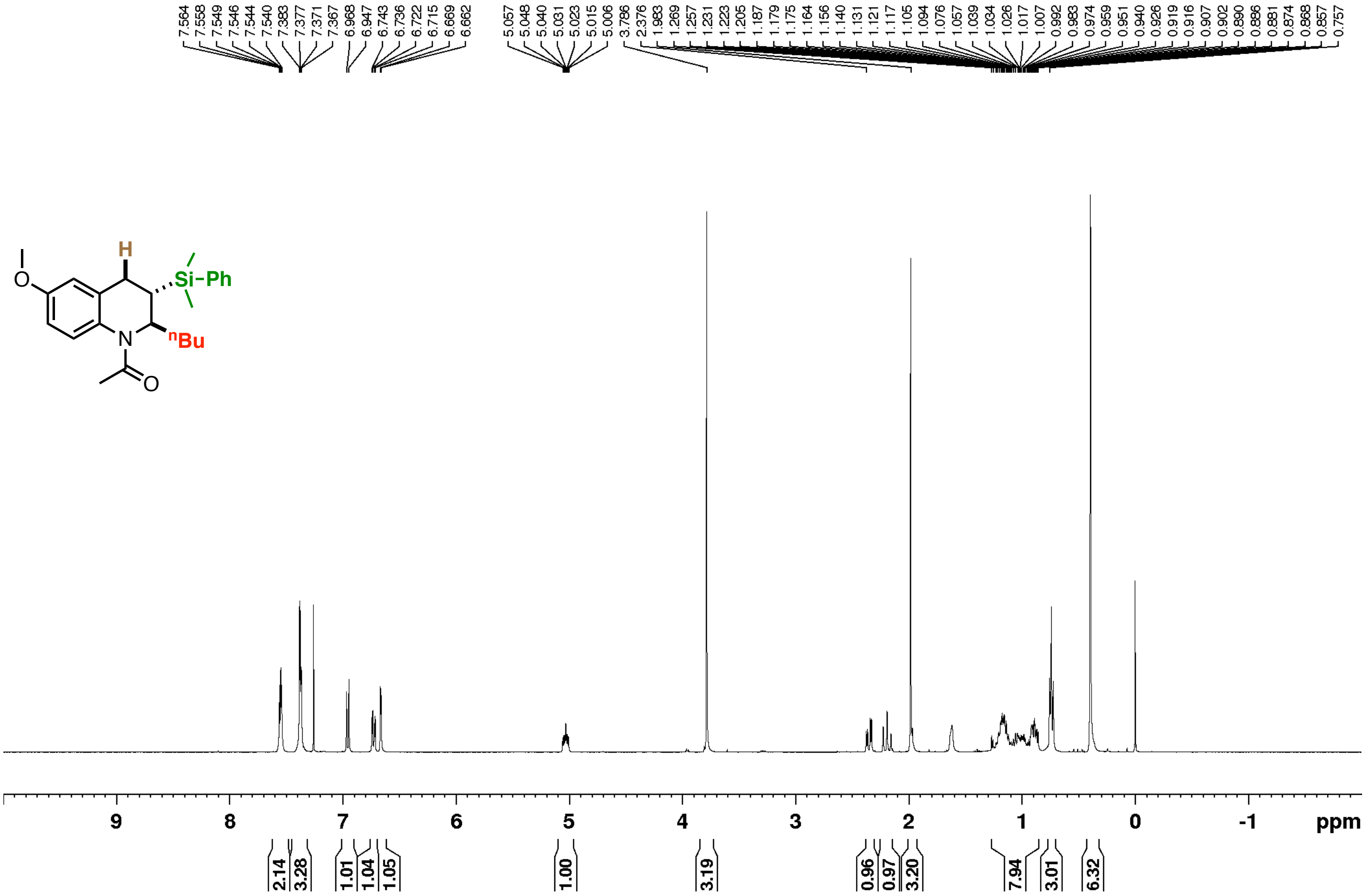

Supplementary Fig 66. **^{1}H NMR spectrum (400 MHz, $CDCl_3$, r.t.) of 7-H**.

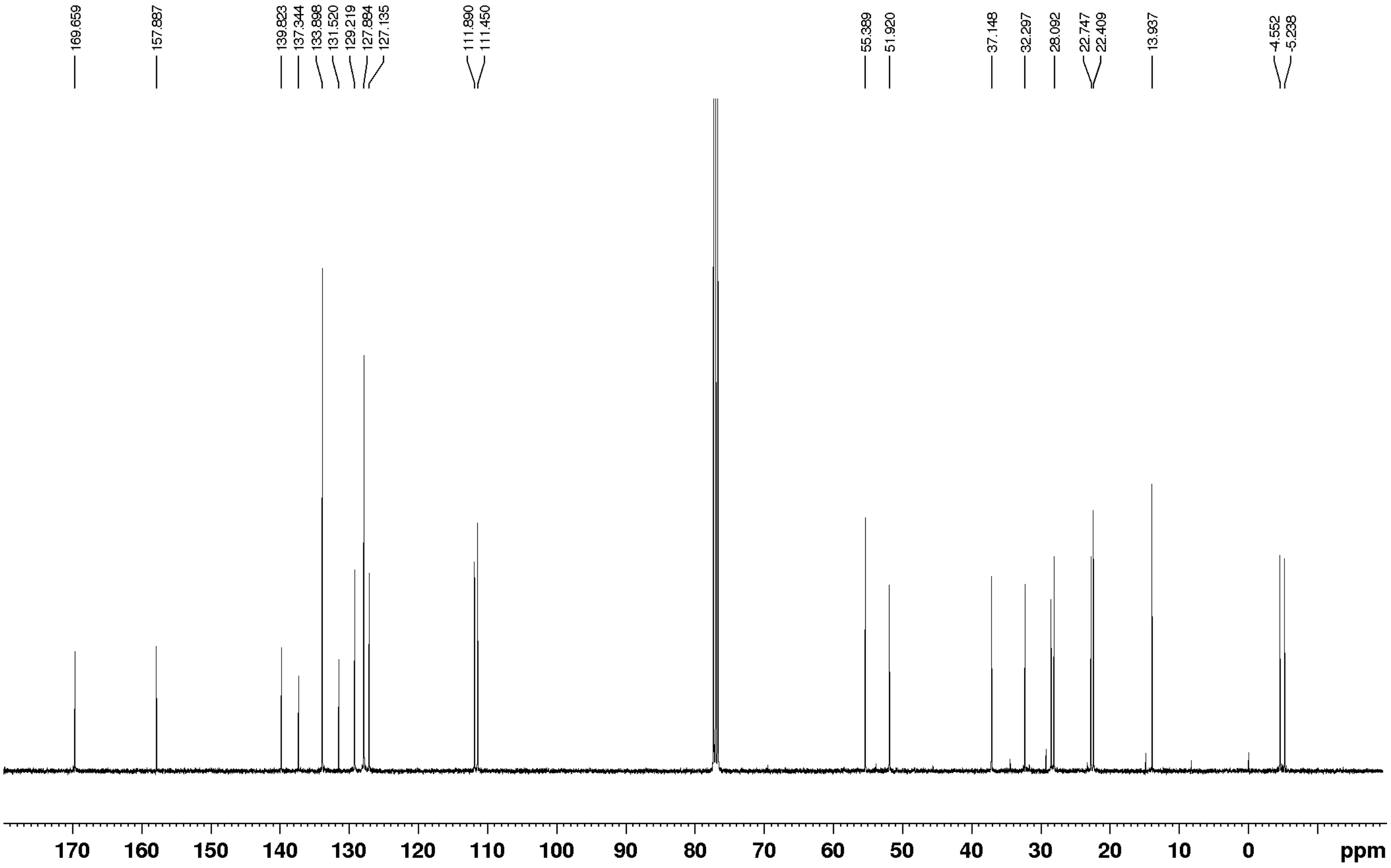

Supplementary Fig 67. **^{13}C NMR spectrum (100 MHz, $CDCl_3$, r.t.) of 7-H**.

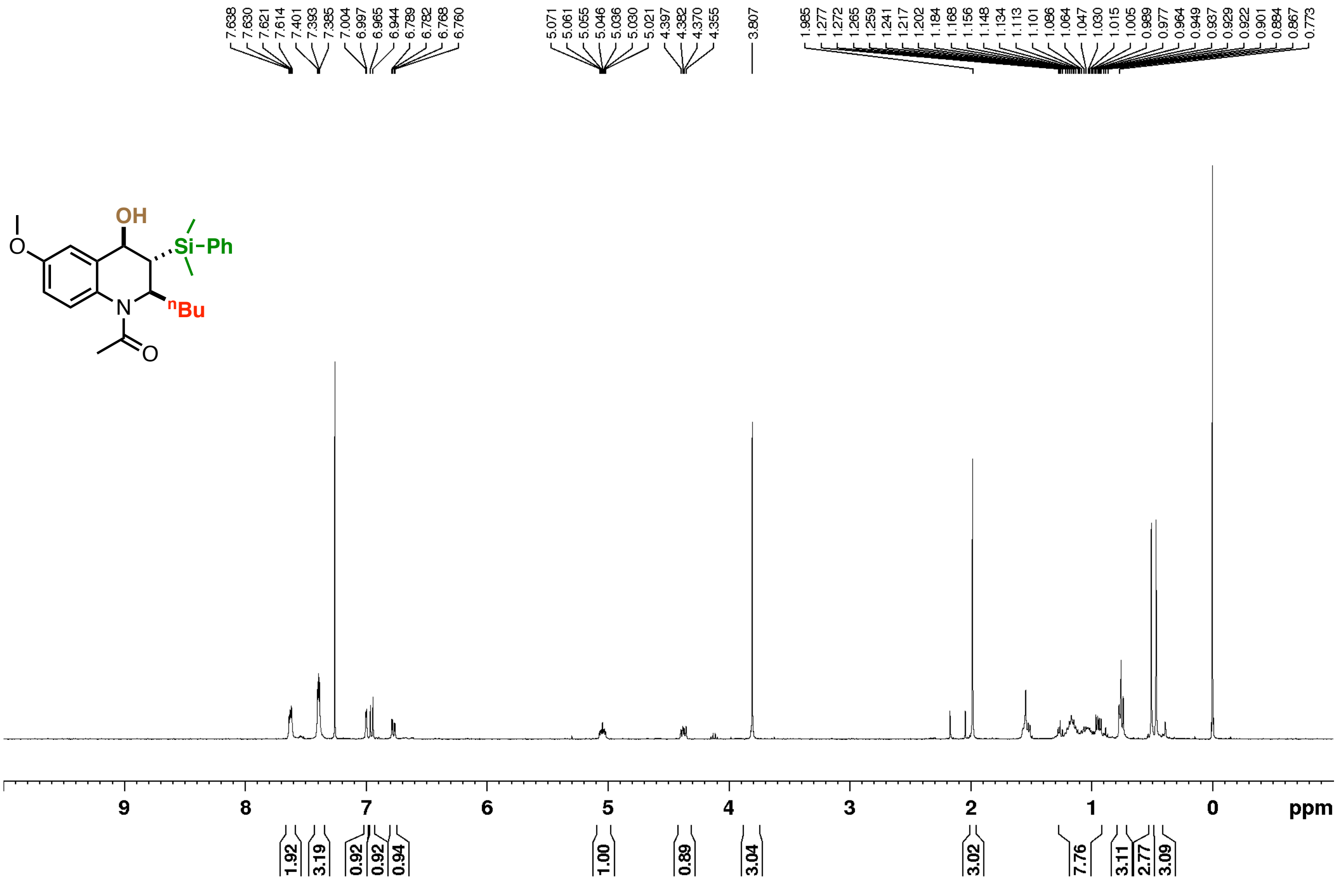

Supplementary Fig 68. ^{1}H NMR spectrum (400 MHz, $CDCl_3$, r.t.) of **8**.

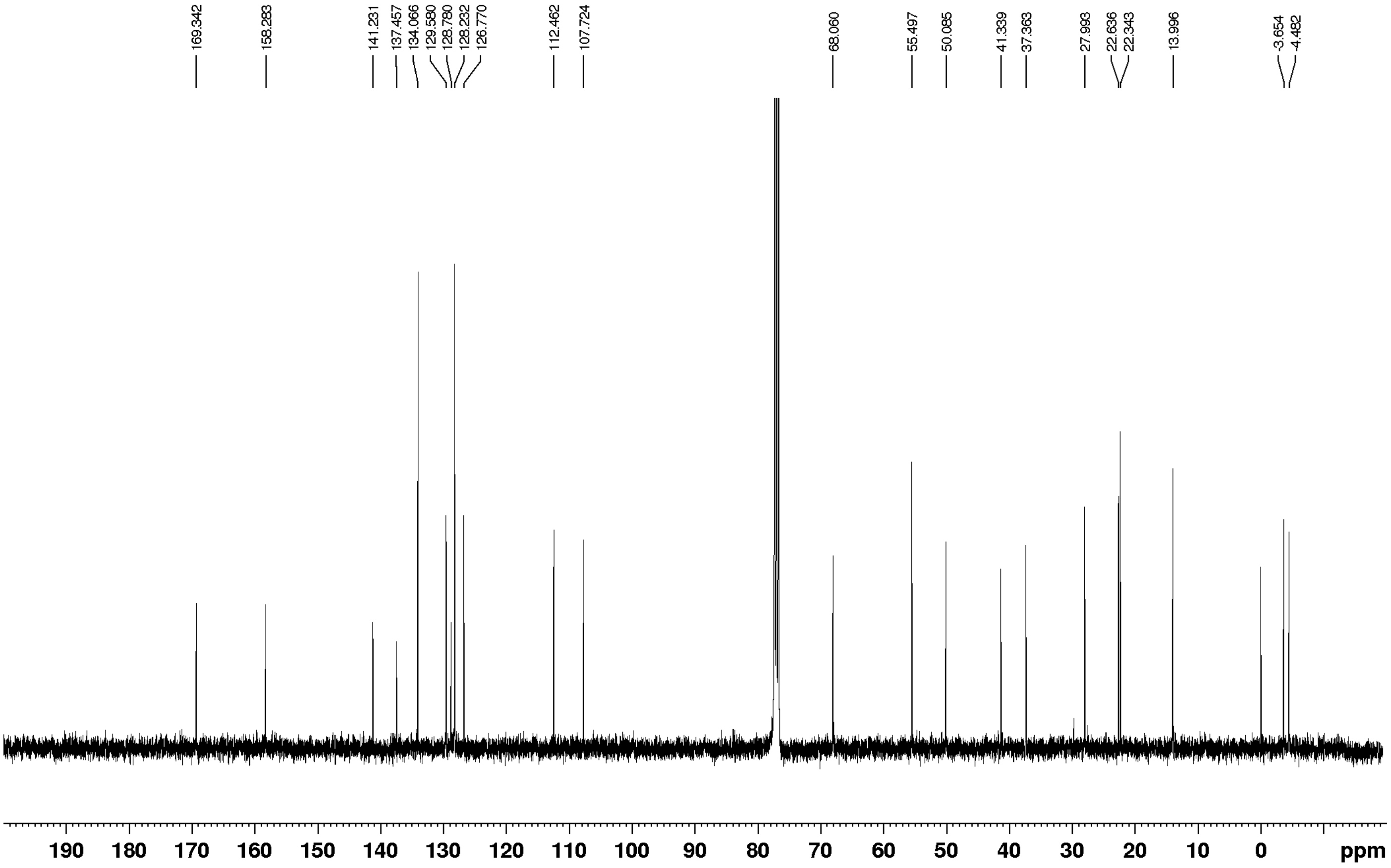

Supplementary Fig 69. ^{13}C NMR spectrum (100 MHz, $CDCl_3$, r.t.) of **8**.

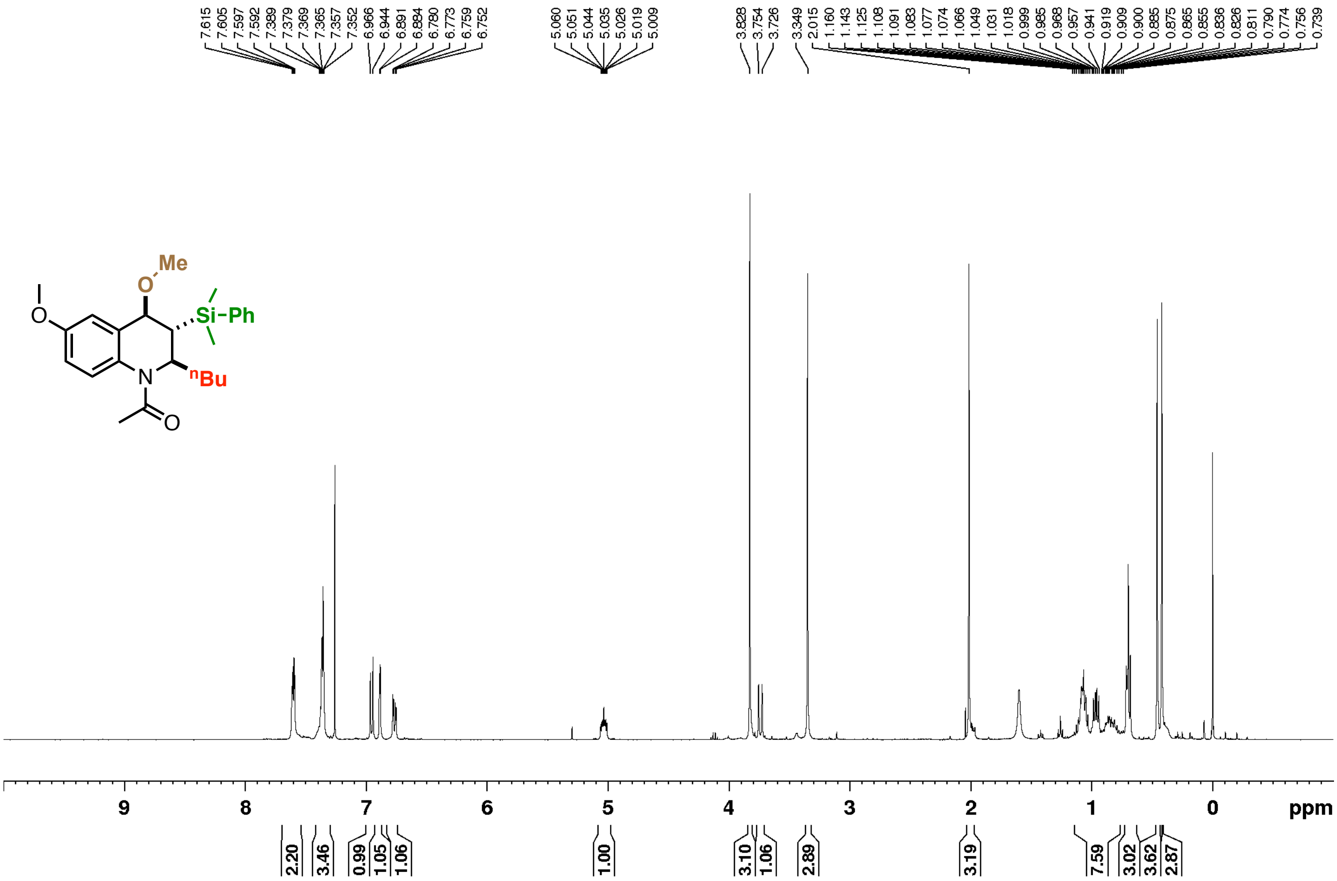

Supplementary Fig 70. ^{1}**H NMR spectrum (400 MHz, CDCl**$_3$**, r.t.) of 9**.

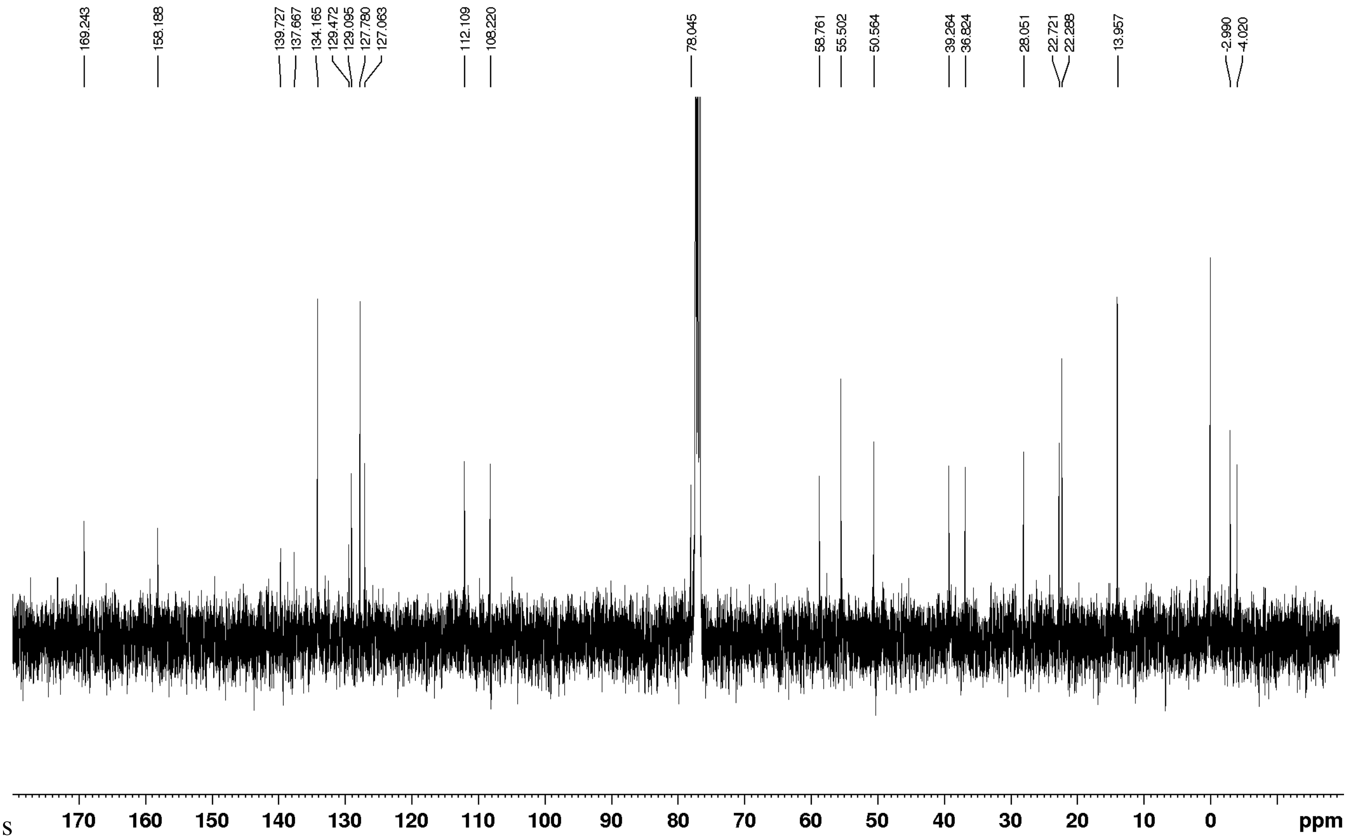

S

Supplementary Fig 71. ^{13}**C NMR spectrum (100 MHz, CDCl**$_3$**, r.t.) of 9**.

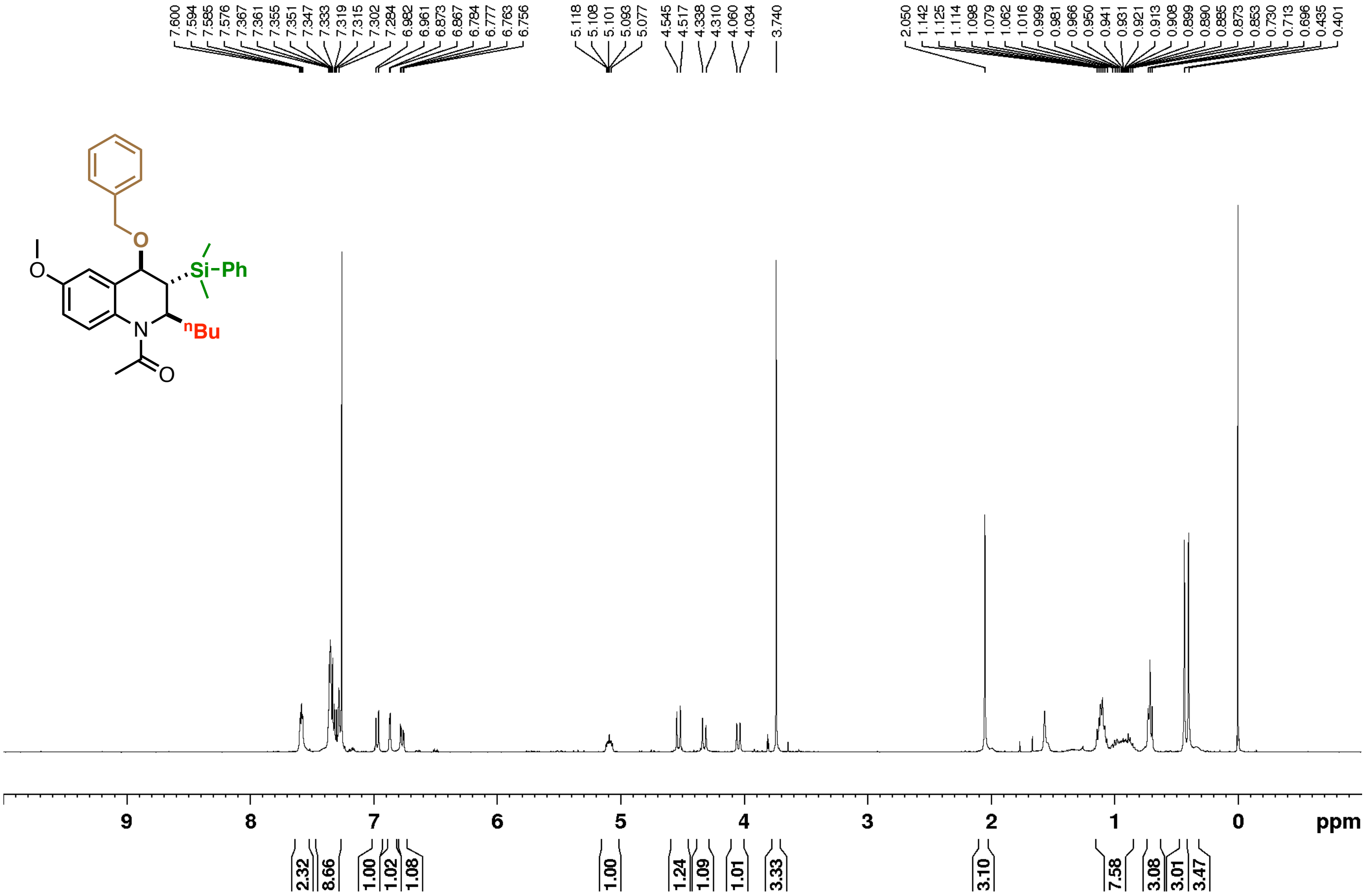

Supplementary Fig 72. ^{1}H NMR spectrum (400 MHz, $CDCl_3$, r.t.) of 10.

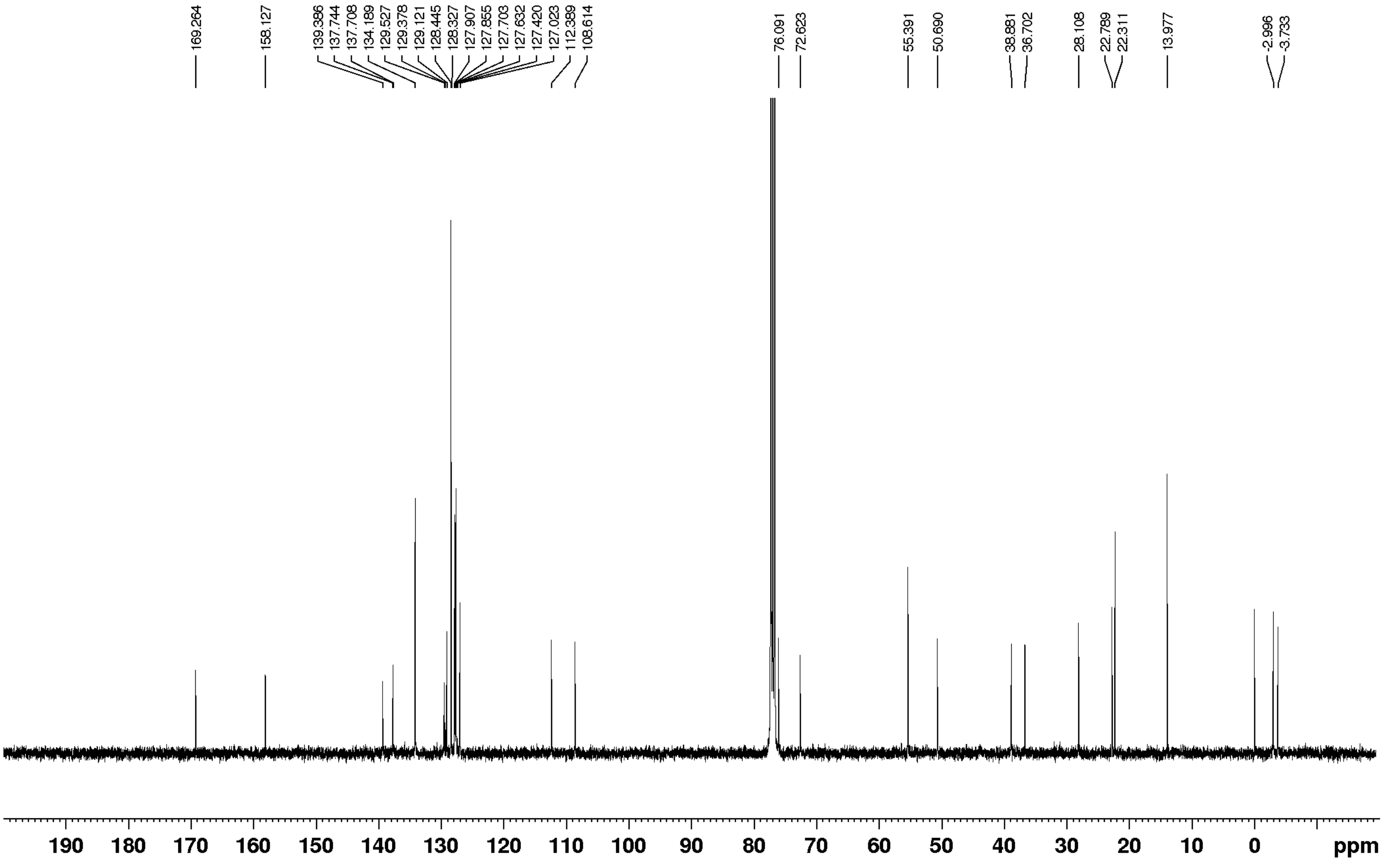

Supplementary Fig 73. ^{13}C NMR spectrum (100 MHz, $CDCl_3$, r.t.) of 10.

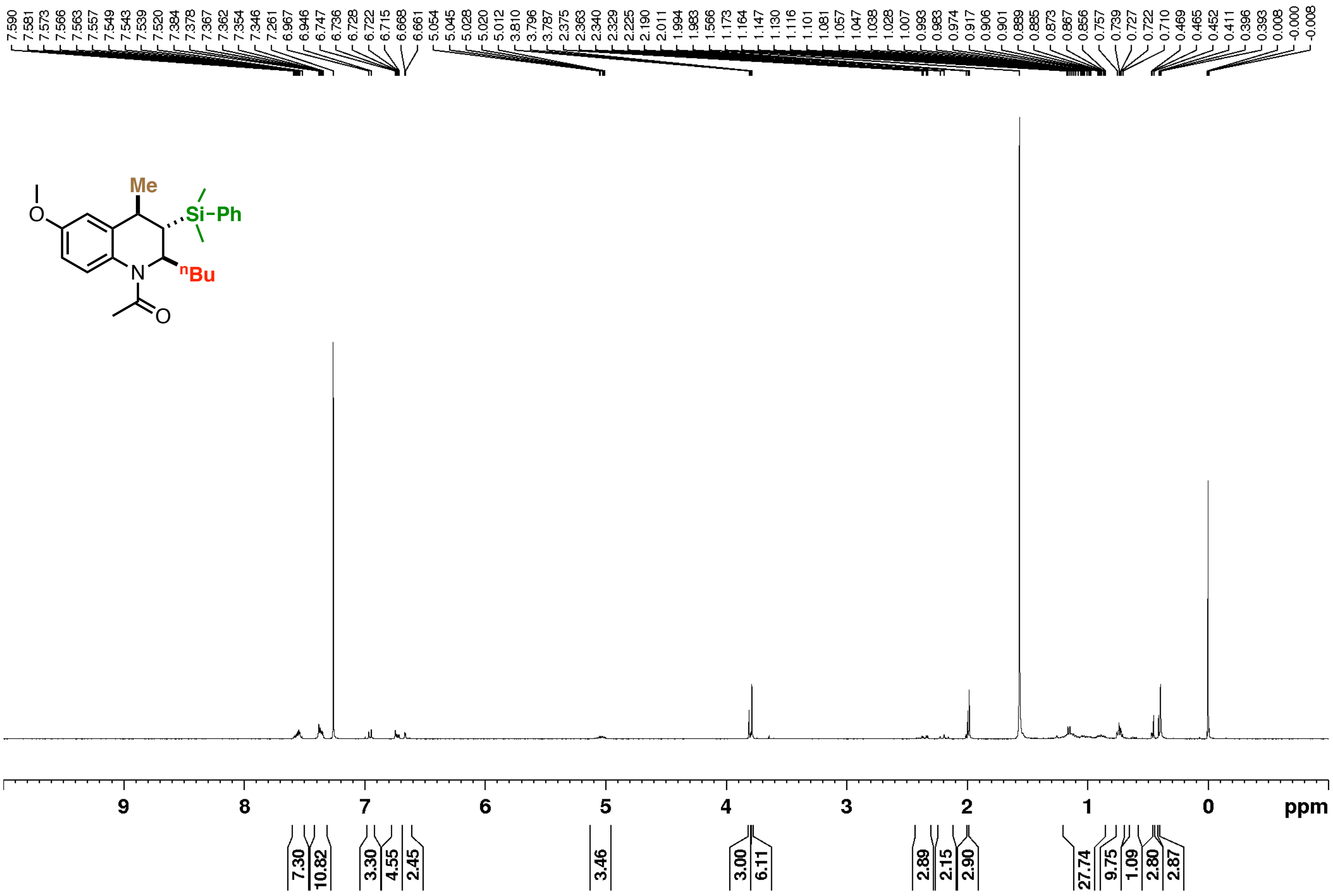

Supplementary Fig 74. **^{1}H NMR spectrum (400 MHz, $CDCl_3$, r.t.) of 11**.

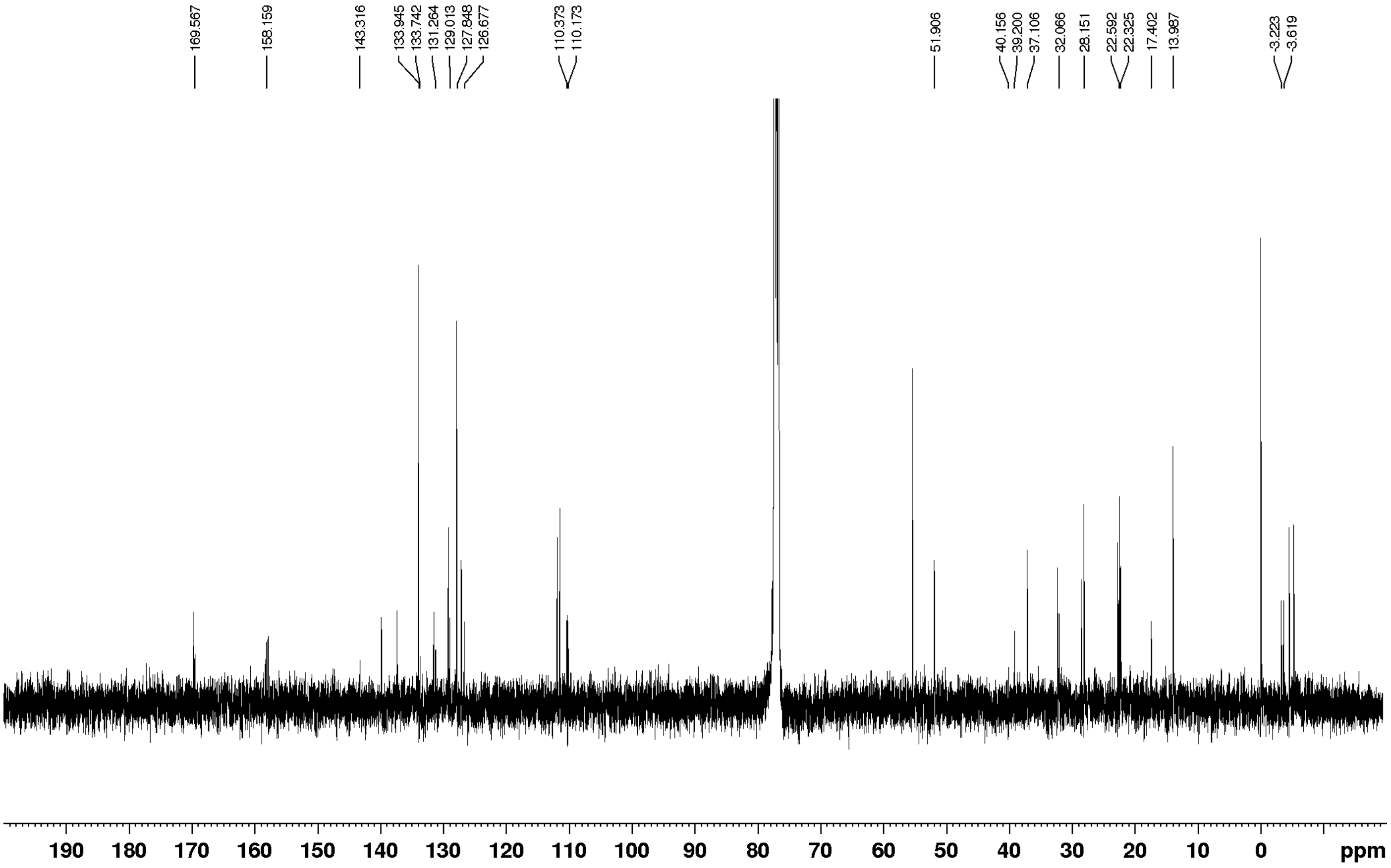

Supplementary Fig 75. **^{13}C NMR spectrum (100 MHz, $CDCl_3$, r.t.) of 11**.

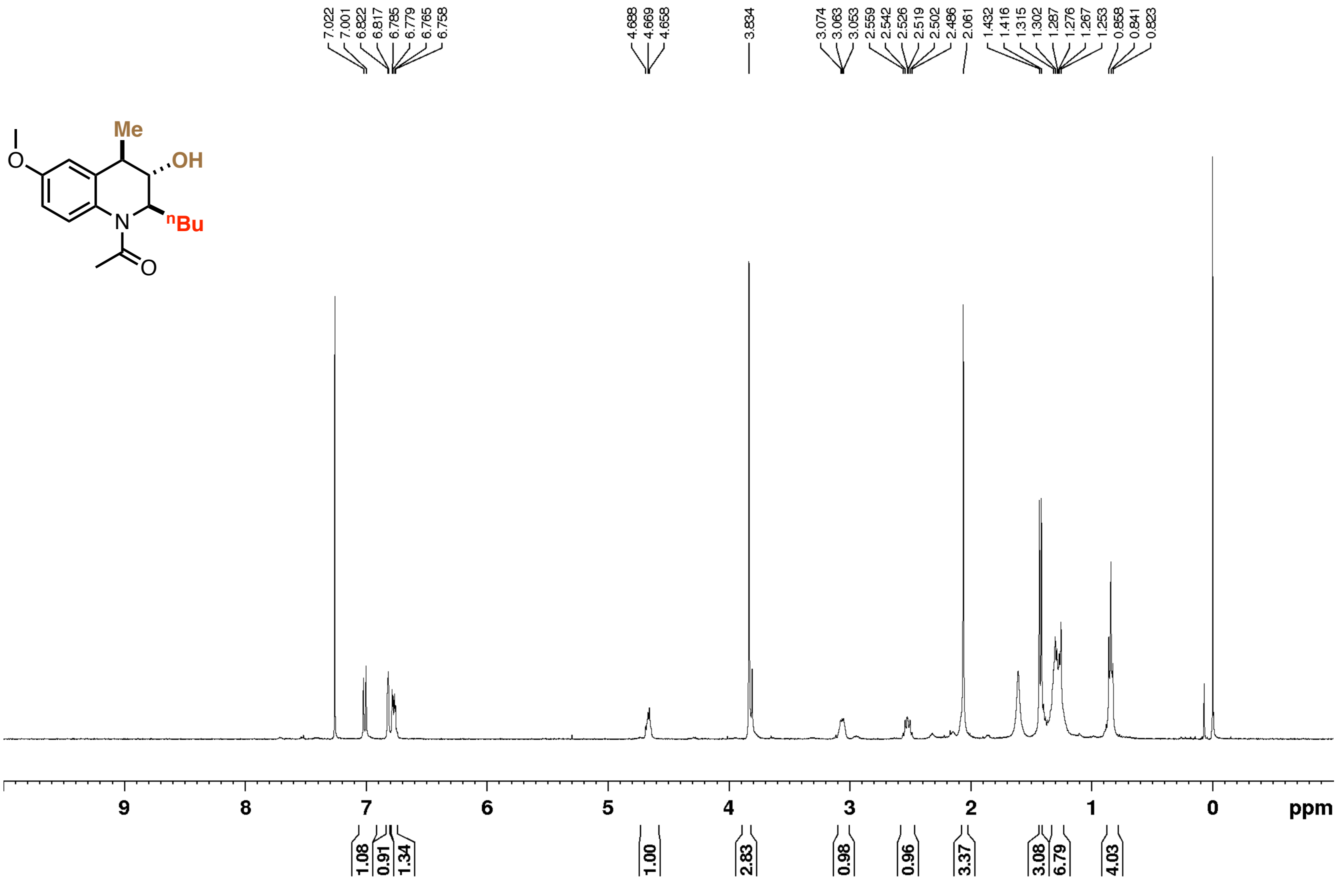

Supplementary Fig 76. ^{1}H NMR spectrum (400 MHz, $CDCl_3$, r.t.) of 13.

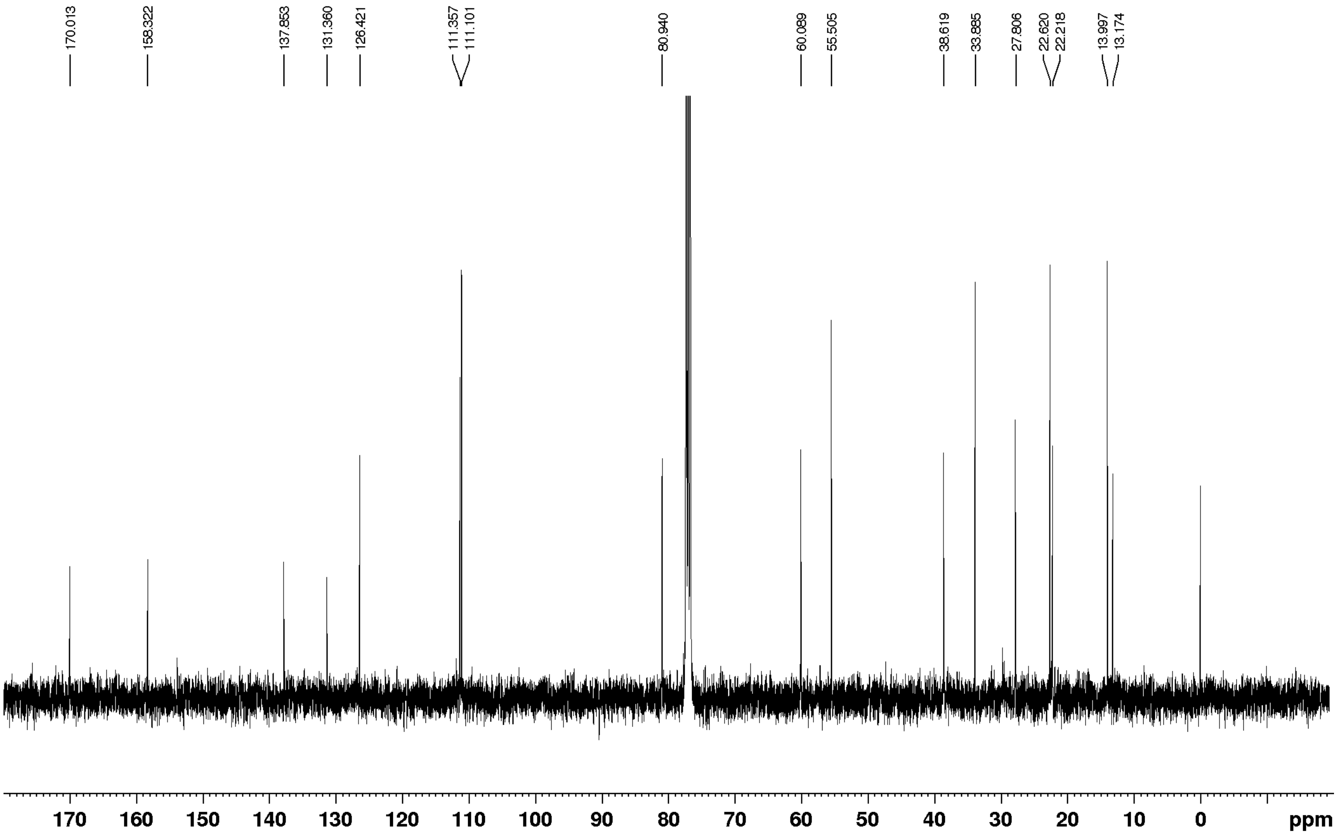

Supplementary Fig 77. ^{13}C NMR spectrum (100 MHz, $CDCl_3$, r.t.) of 13.

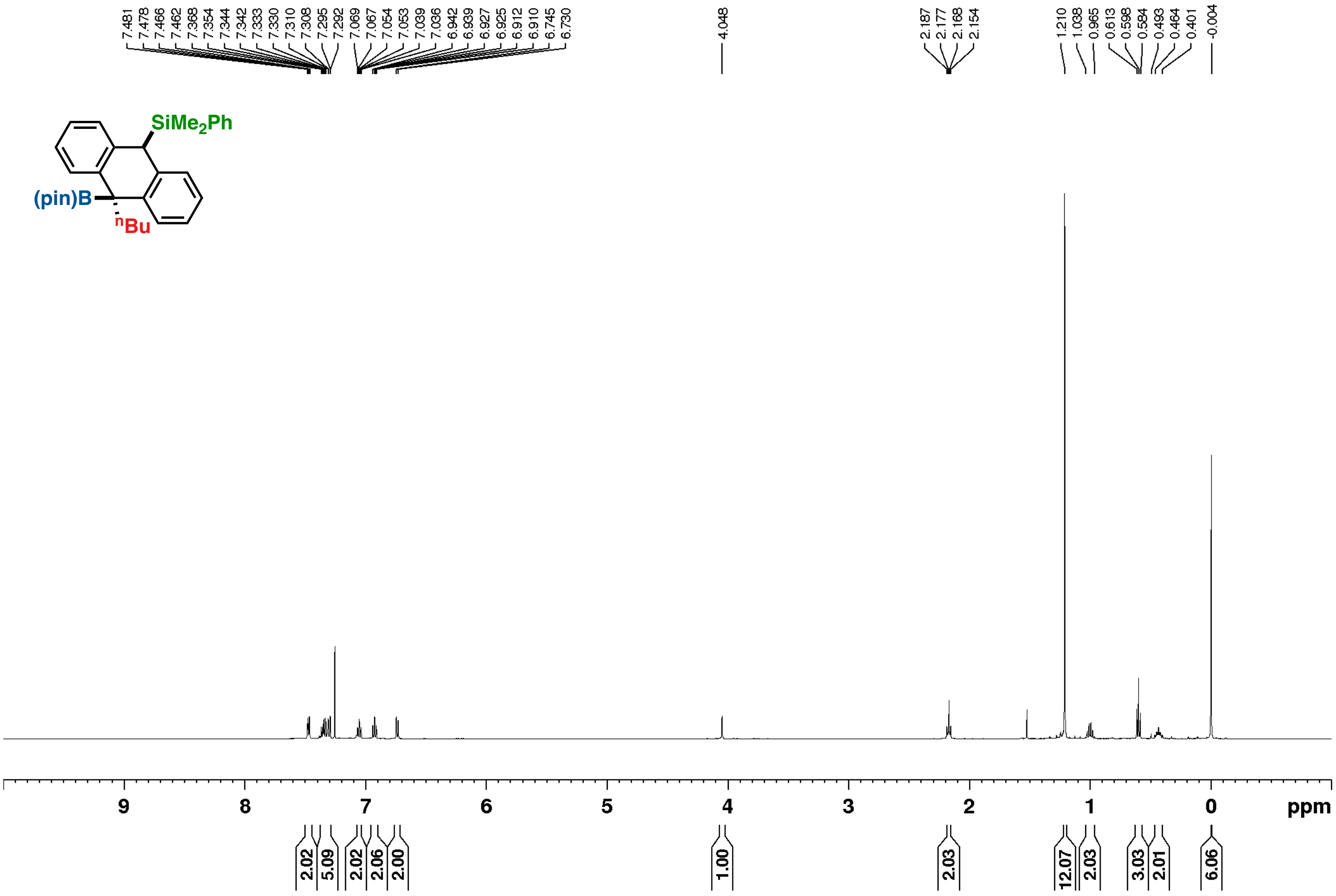

Supplementary Fig 78. ^{1}H NMR spectrum (500 MHz, $CDCl_3$, r.t.) of 19.

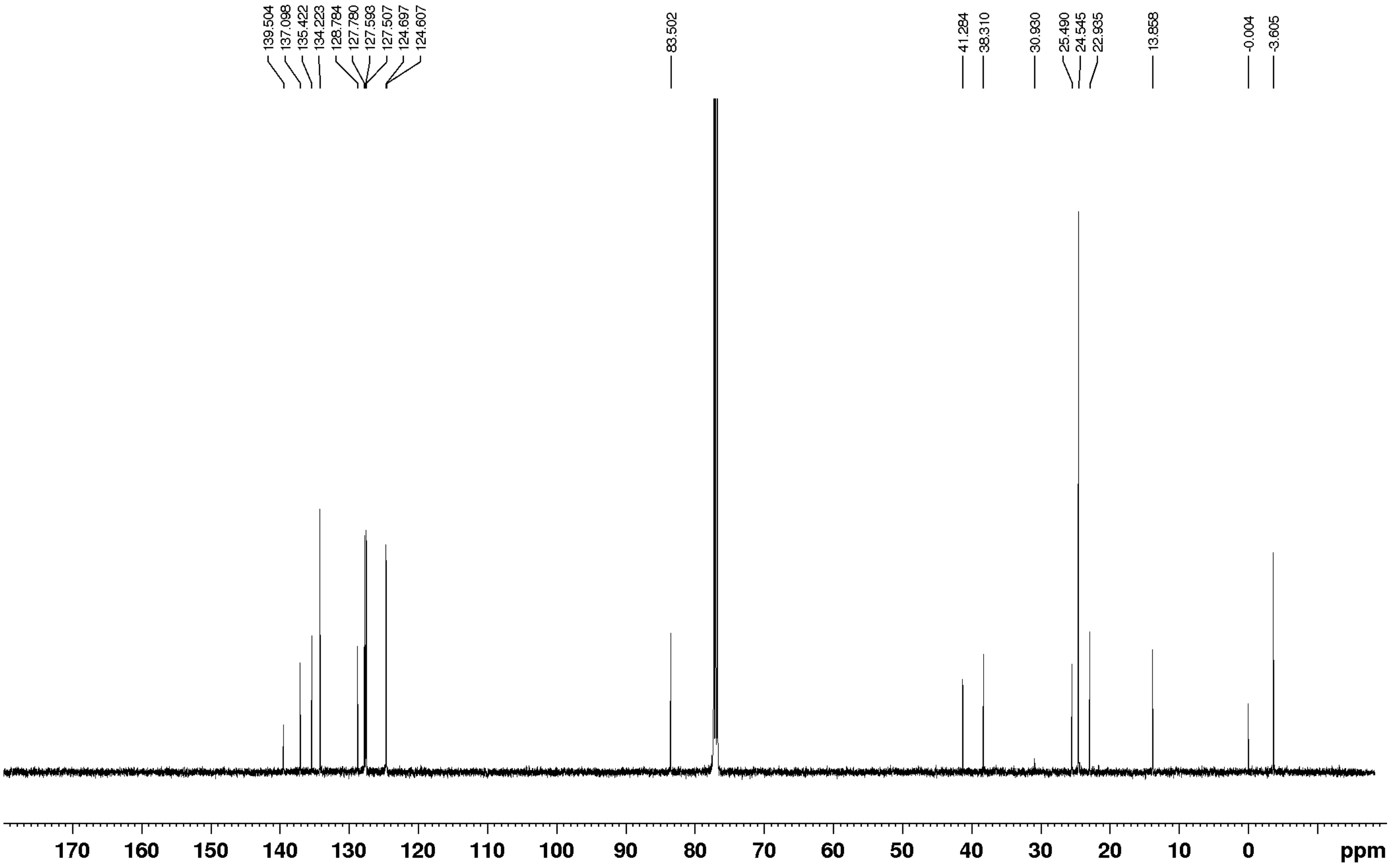

Supplementary Fig 79. ^{13}C NMR spectrum (125 MHz, $CDCl_3$, r.t.) of 19.

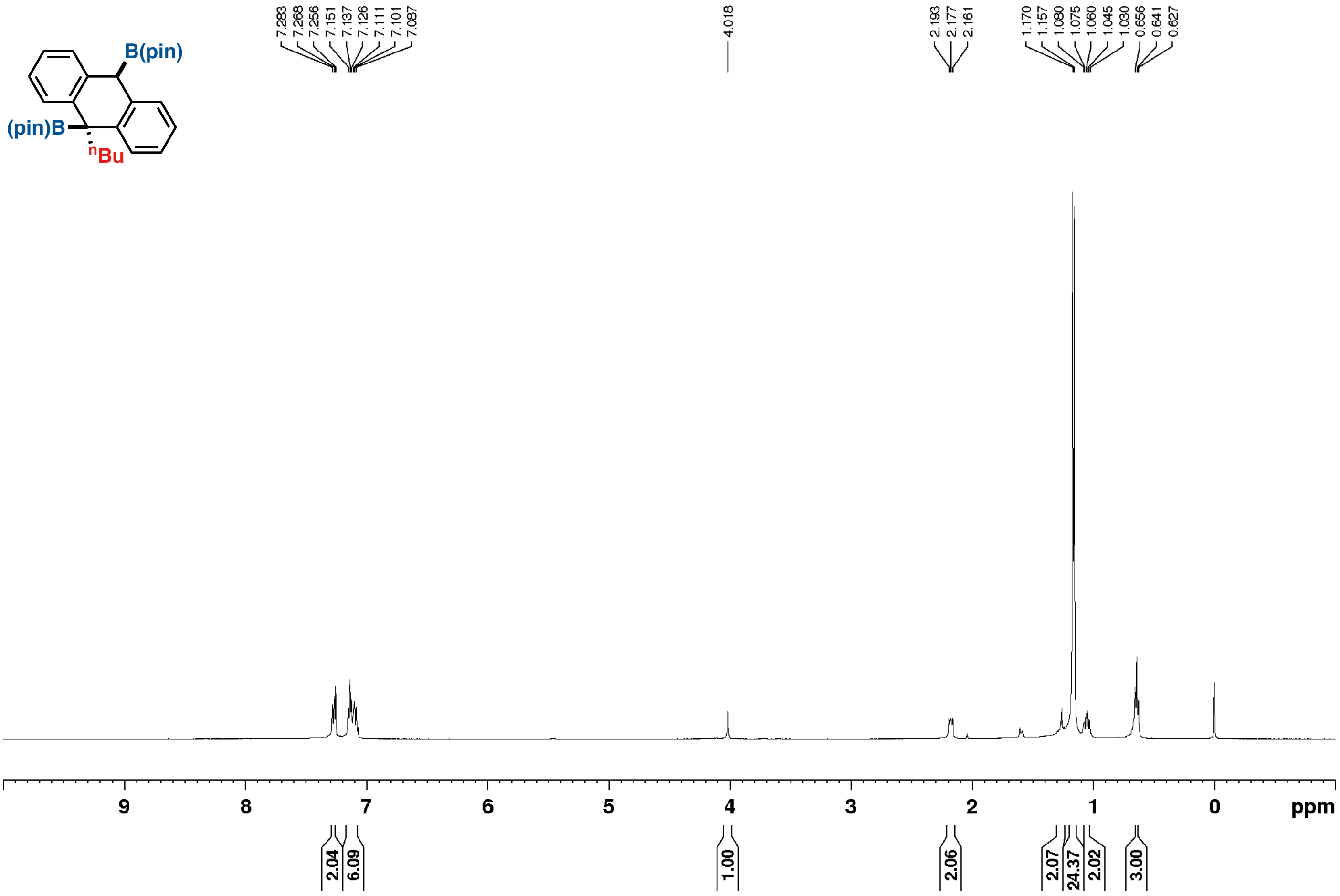

Supplementary Fig 80. ^{1}H NMR spectrum (500 MHz, $CDCl_3$, r.t.) of 20-1.

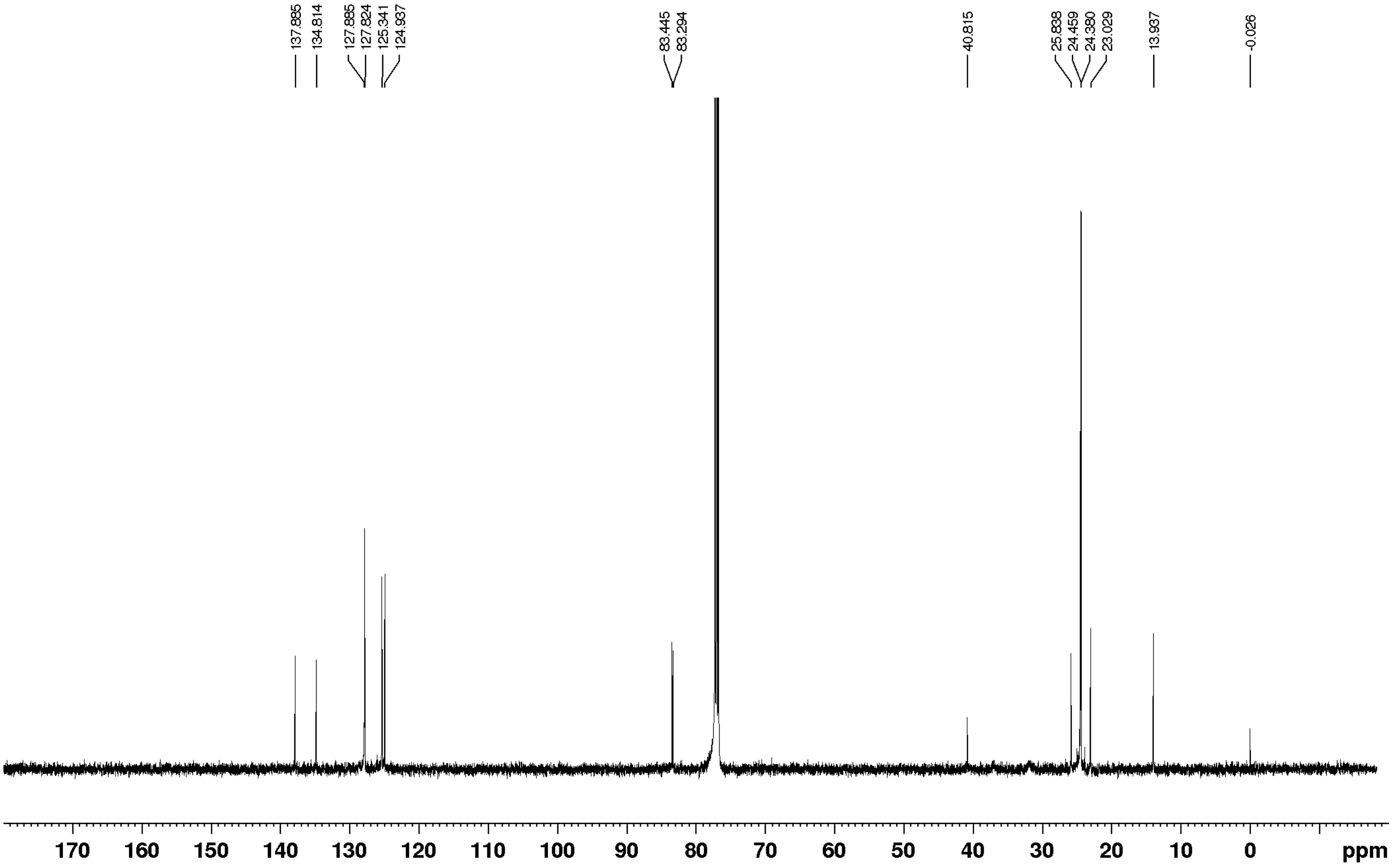

Supplementary Fig 81. ^{13}C NMR spectrum (125 MHz, $CDCl_3$, r.t.) of 20-1.

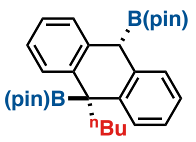

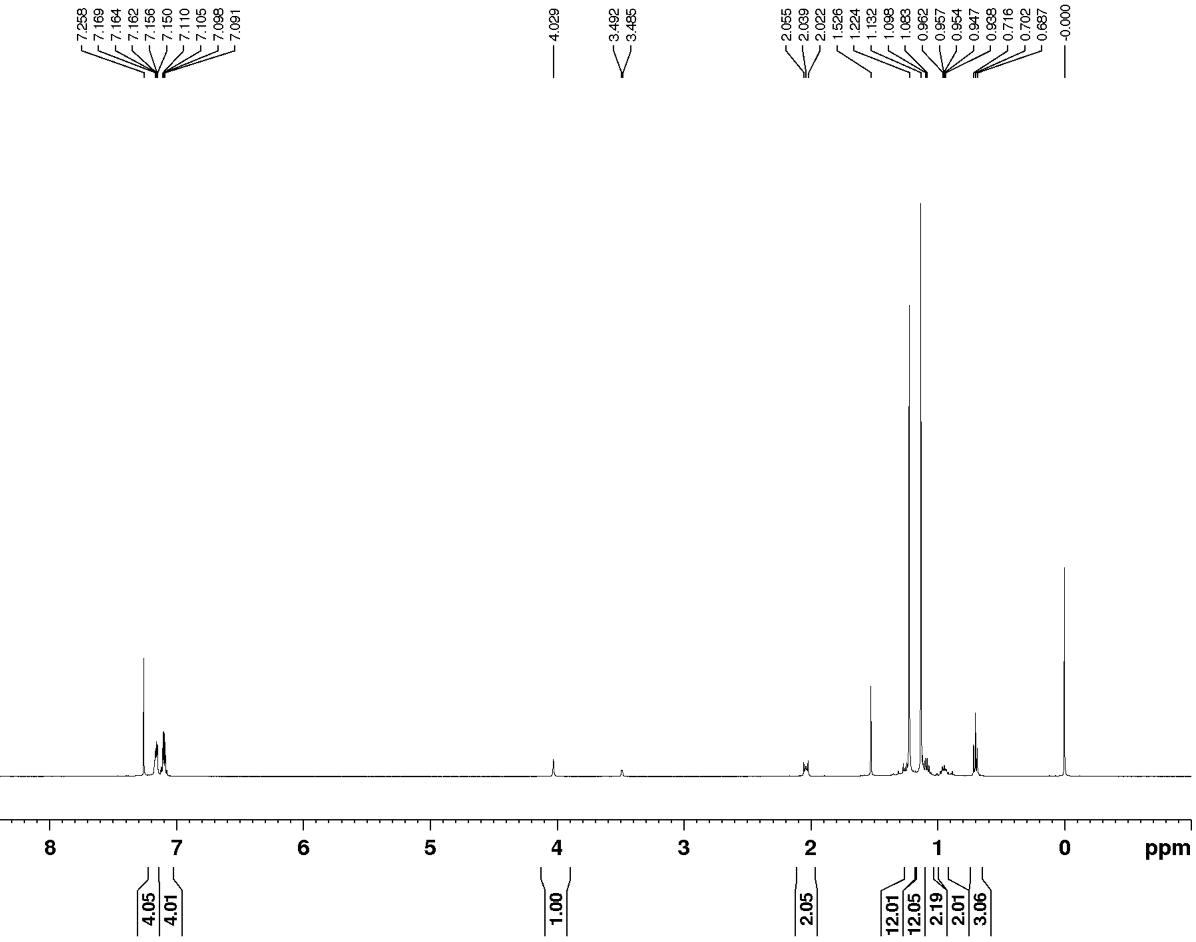

Supplementary Fig 82. ^{1}H NMR spectrum (500 MHz, $CDCl_3$, r.t.) of 20-2.

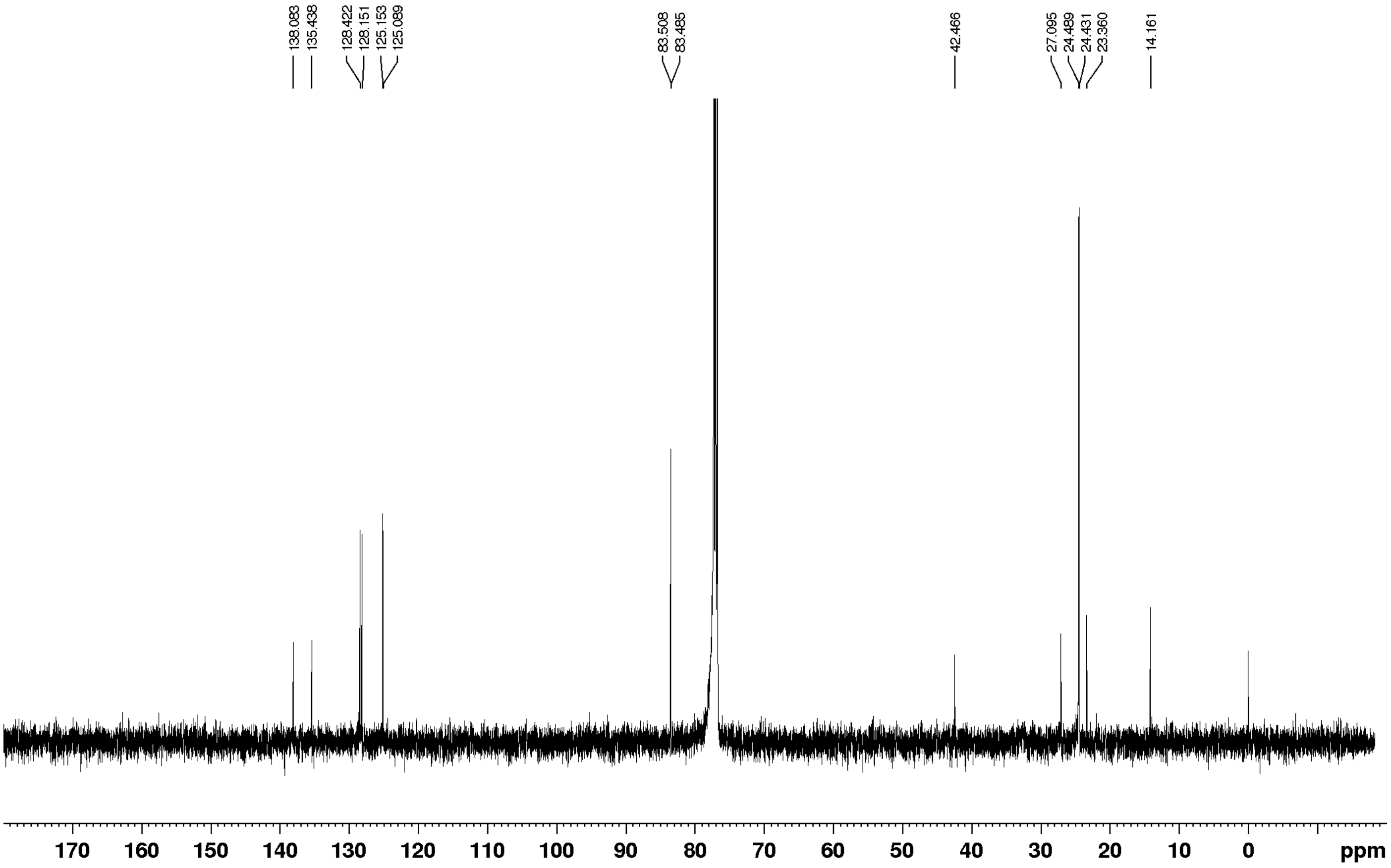

Supplementary Fig 83. ^{13}C NMR spectrum (125 MHz, $CDCl_3$, r.t.) of 20-2.

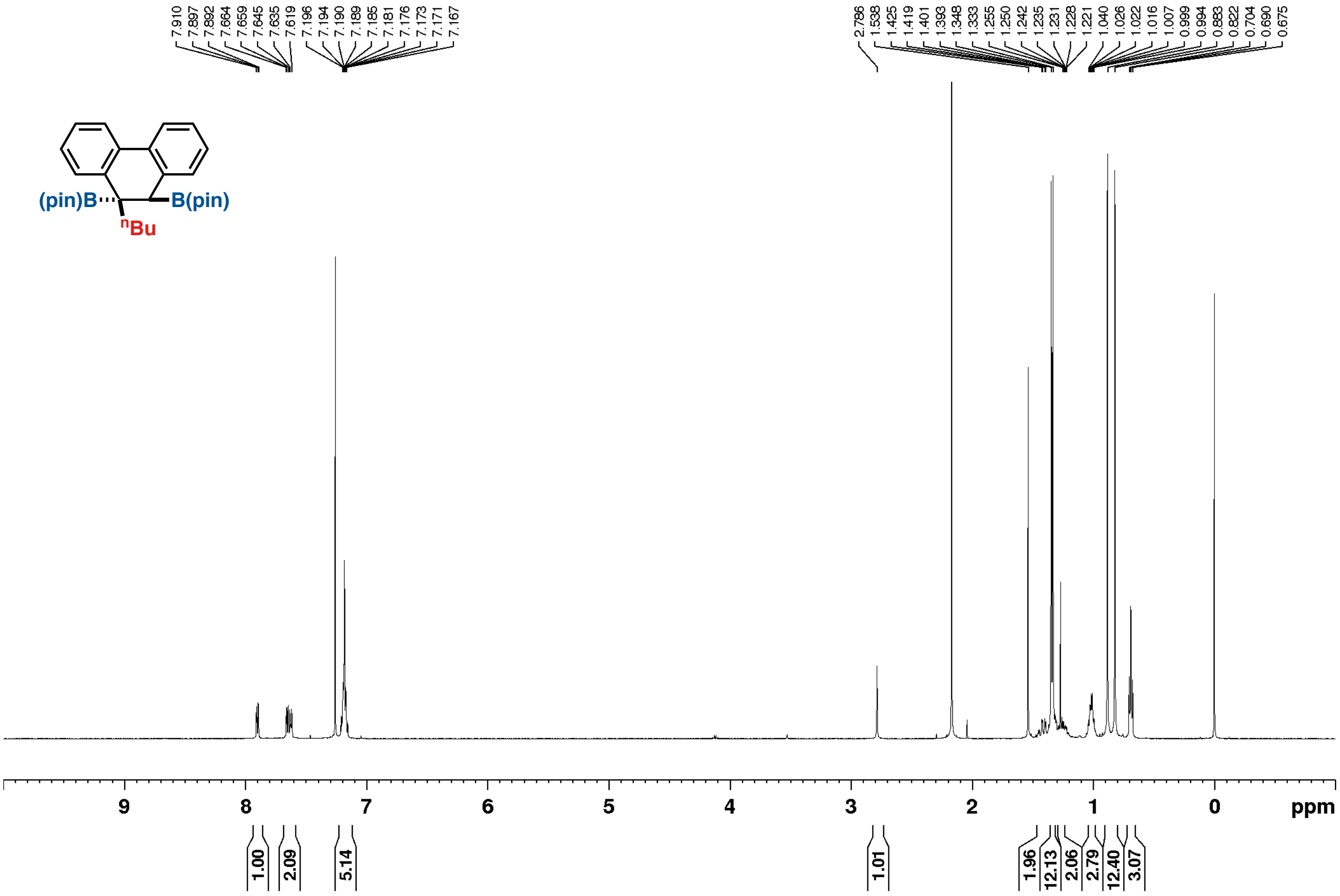

Supplementary Fig 84. ^{1}**H NMR spectrum (500 MHz, CDCl$_3$, r.t.) of 22**.

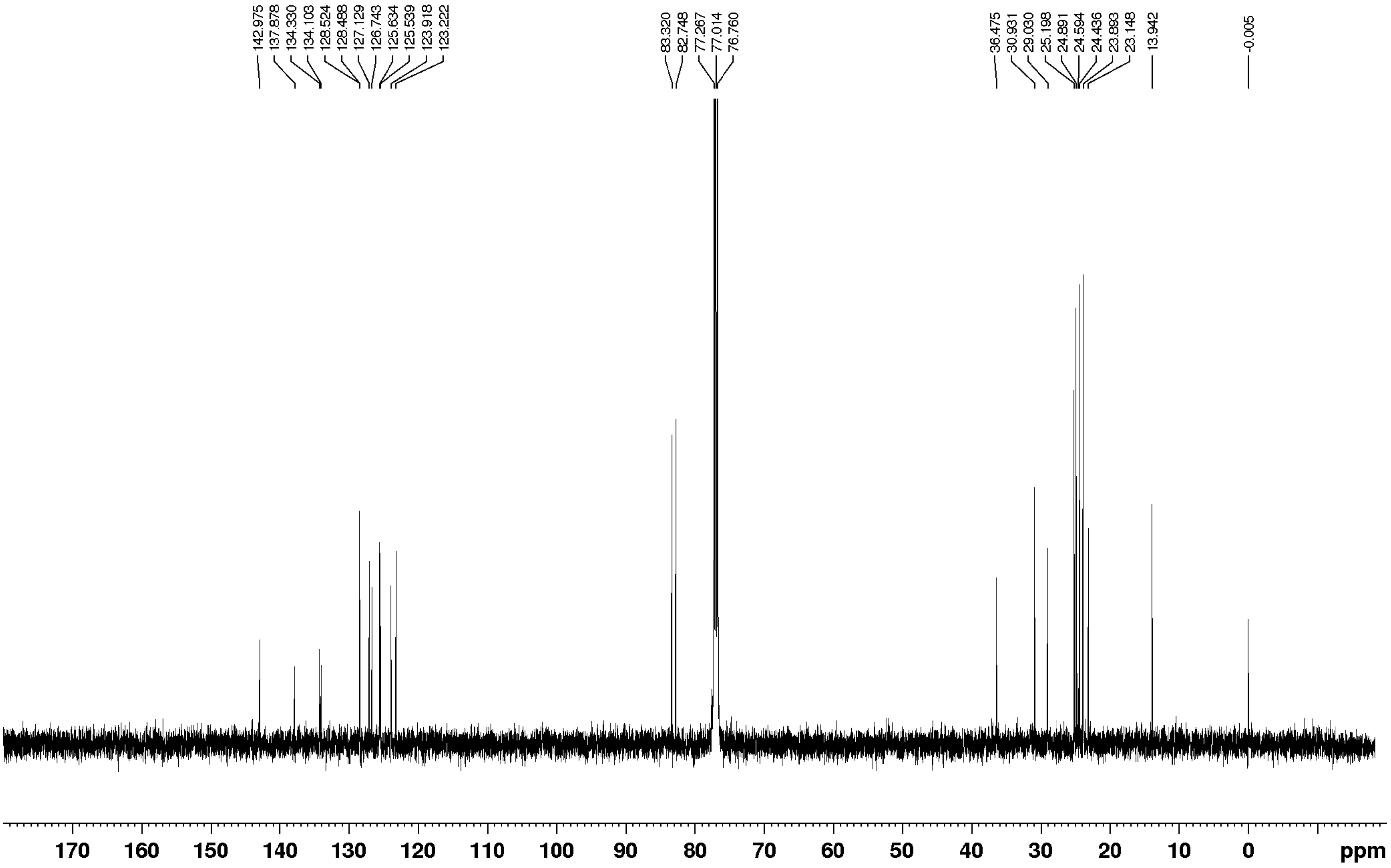

Supplementary Fig 85. ^{13}**C NMR spectrum (125 MHz, CDCl$_3$, r.t.) of 22**.

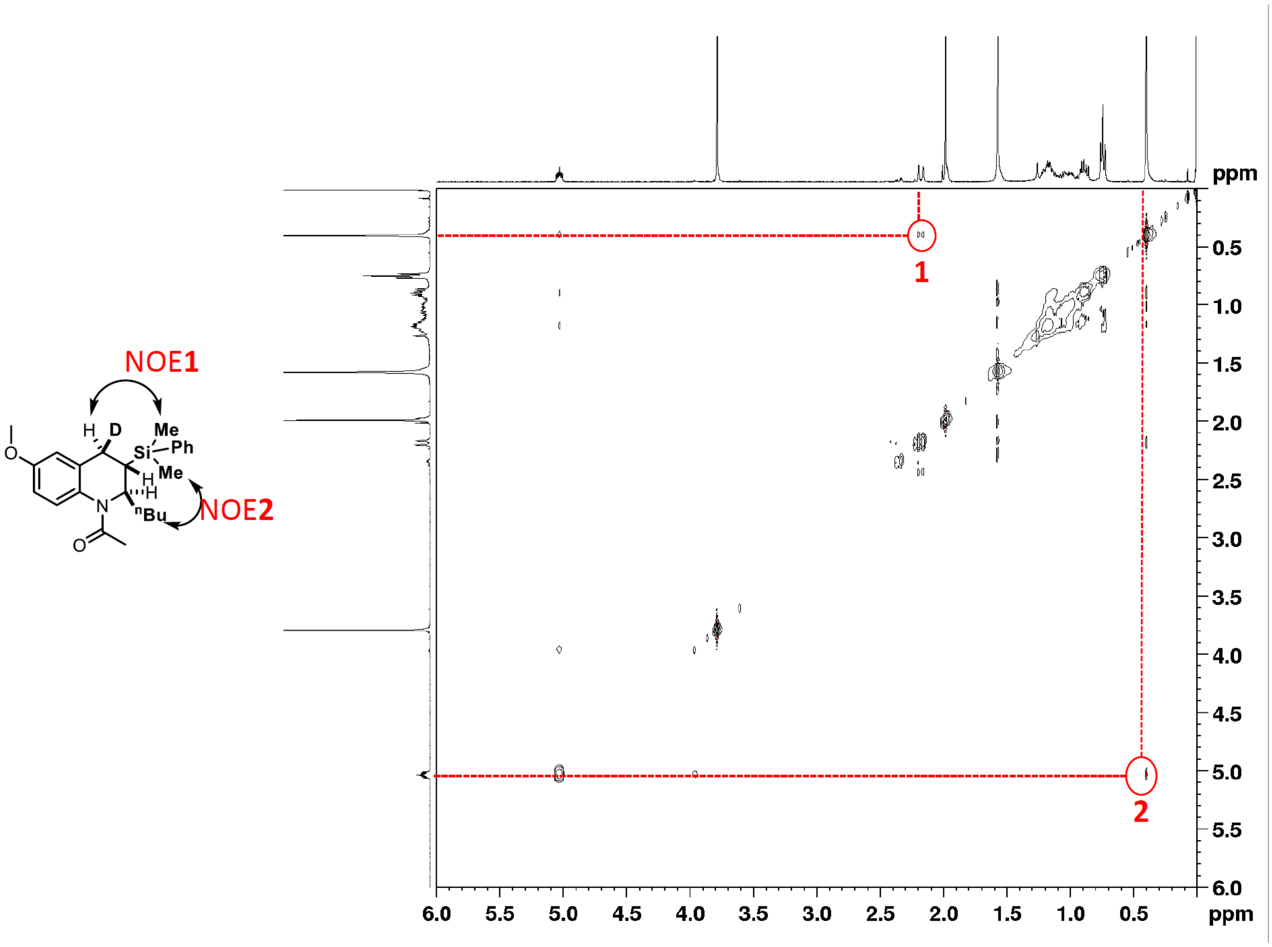

Supplementary Fig 86. NOESY spectrum (400 MHz, CDCl₃, r.t.) of 7-D.

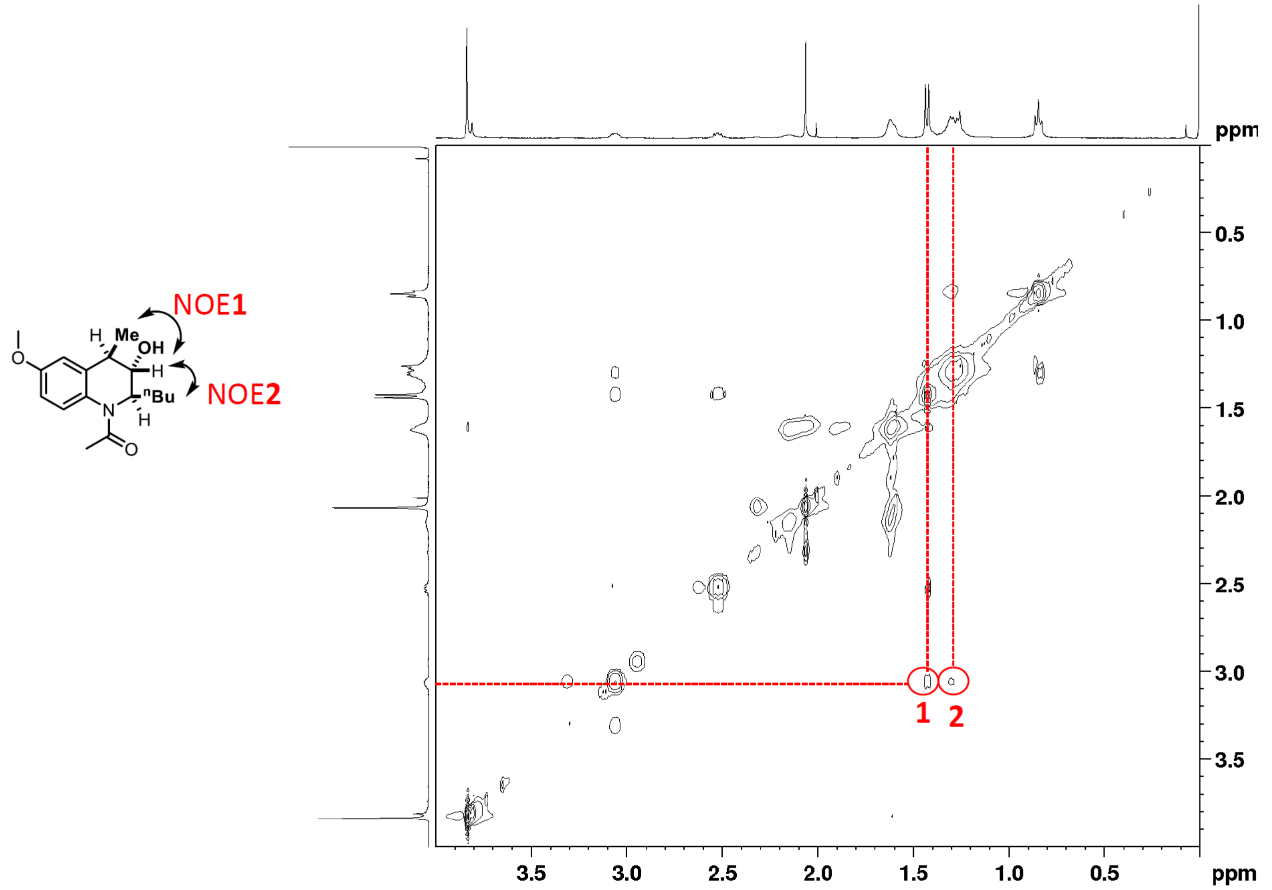

Supplementary Fig 87. NOESY spectrum (400 MHz, CDCl₃, r.t.) of 13.

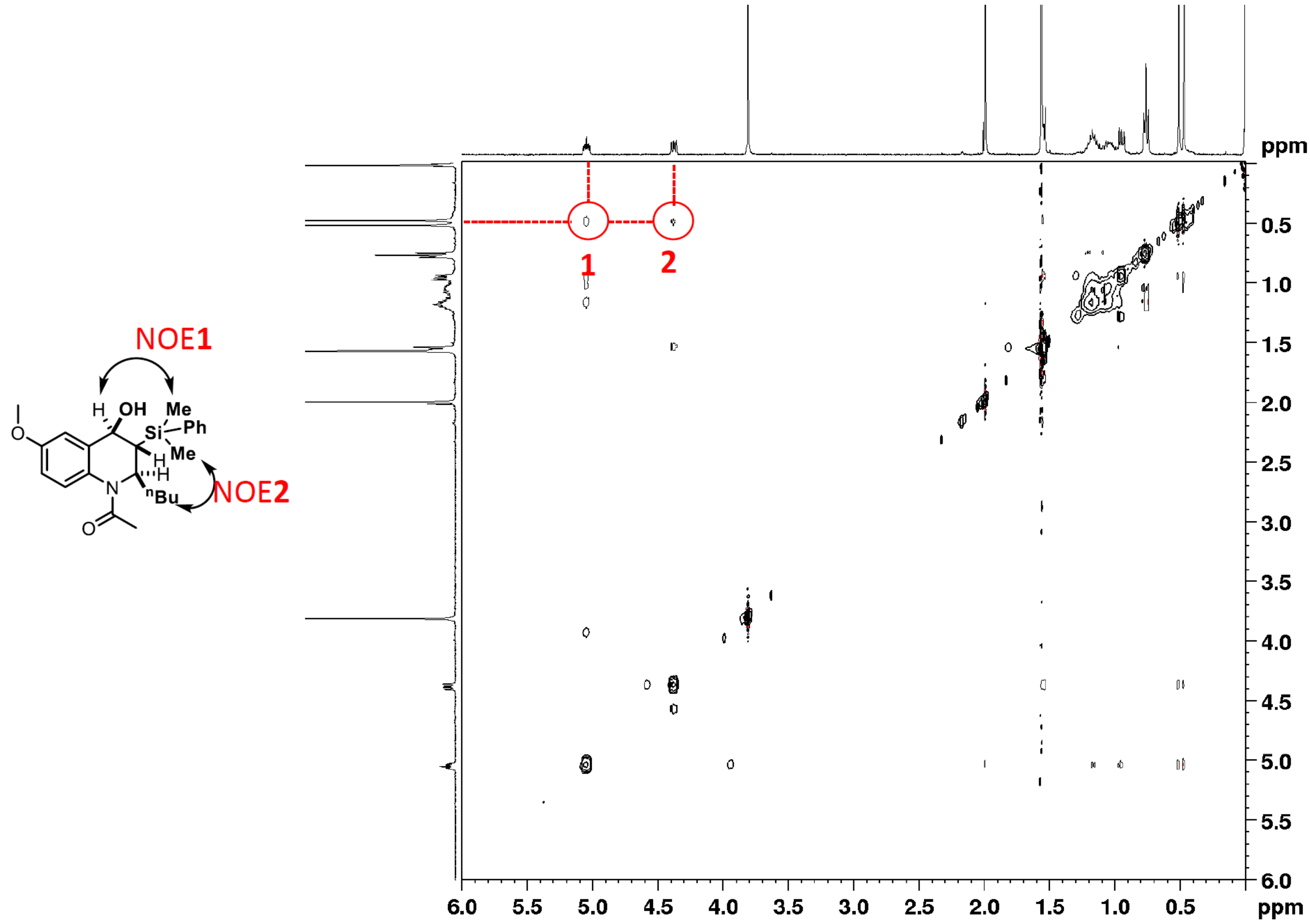

Supplementary Fig 88. NOESY spectrum (400 MHz, $CDCl_3$, r.t.) of 8.

7. Supplementary References

(1) (a) Takeuchi, T.; Shishido, R.; Kubota, K.; Ito, H. Synthesis of hydrosilylboronates via the monoborylation of a dihydrosilane Si–H bond and their application for the generation of dialkylhydrosilyl anions. *Chem. Sci.* **2021**, *12*, 11799–11804. (b) Shishido, R.; Uesugi, M.; Takahashi, R.; Mita, T.; Ishiyama, T.; Kubota, K.; Ito, H. General Synthesis of Trialkyl- and Dialkylarylsilylboranes: Versatile Silicon Nucleophiles in Organic Synthesis. *J. Am. Chem. Soc.* **2020**, *142*, 14125–14133.

(2) Gaussian 16, Revision A.03, Frisch, M. J.; Trucks, G. W.; Schlegel, H. B.; Scuseria, G. E.; Robb, M. A.; Cheeseman, J. R.; Scalmani, G.; Barone, V.; Petersson, G. A.; Nakatsuji, H.; Li, X.; Caricato, M.; Marenich, A. V.; Bloino, J.; Janesko, B. G.; Gomperts, R.; Mennucci, B.; Hratchian, H. P.; Ortiz, J. V.; Izmaylov, A. F.; Sonnenberg, J. L.; Williams-Young, D.; Ding, F.; Lipparini, F.; Egidi, F.; Goings, J.; Peng, B.; Petrone, A.; Henderson, T.; Ranasinghe, D.; Zakrzewski, V. G.; Gao, J.; Rega, N.; Zheng, G.; Liang, W.; Hada, M.; Ehara, M.; Toyota, K.; Fukuda, R.; Hasegawa, J.; Ishida, M.; Nakajima, T.; Honda, Y.; Kitao, O.; Nakai, H.; Vreven, T.; Throssell, K.; Montgomery, J. A.; Jr., Peralta, J. E.; Ogliaro, F.; Bearpark, M. J.; Heyd, J. J.; Brothers, E. N.; Kudin, K. N.; Staroverov, V. N.; Keith, T. A.; Kobayashi, R.; Normand, J.; Raghavachari, K.; Rendell, A. P.; Burant, J. C.; Iyengar, S. S.; Tomasi, J.; Cossi, M.; Millam, J. M.; Klene, M.; Adamo, C.; Cammi, R.; Ochterski, J. W.; Martin, R. L.; Morokuma, K.; Farkas, O.; Foresman, J. B.; Fox, D. J. Gaussian, Inc., Wallingford CT, 2016.

(3) (a) Beche, A. D. *Phys. Rev.* **1988**, *A38*, 3098-3100. (b) Beche, A. D. J. *Chem. Phys.* **1993**, *98*, 1372-1377. (c) Beche, A. D. J. *Chem. Phys.* **1993**, *98*, 5648-5652. (d) Lee, C; Yang, W.; Parr, R. G. *Phys. Rev.* **1988**, *B37*, 785-788. (d) Krishnan, R.; Binkley, J. S.; Seeger, R.; Pople, J. A. Self-consistent molecular orbital methods. XX. A basis set for correlated wave functions. *J. Chem. Phys.* **1980**, 72, 650-654. (e) McLean, A. D.; Chandler, G. S. Contracted Gaussian basis sets for molecular calculations. I. Second row atoms, *Z* = 11–18. *J. Chem. Phys.* **1980**, *72*, 5639–5648.

(4) Tomasi, J.; Persico, M. Molecular Interactions in Solution: An Over-view of Methods Based on Continuous Distributions of the Solvent. *Chem. Rev.* **1994**, *94*, 2027–2094.

(5) (a) Fukui, K. The path of chemical reactions - the IRC approach. *Acc. Chem. Res.* **1981**, *14*, 363–368. (b) Ishida, K.; Morokuma, K.; Ko-mornicki, A. The intrinsic reaction coordinate. An ab initio calcula-tion for HNC -> HCN and H^- $+CH_4$ -> CH_4+H^- *J. Chem. Phys.* **1977**, *66*, 2153–2156. (c) Gonzalez, C.; Schlegel, H. B. An improved algorithm for reaction path following. *J. Chem. Phys.* **1989**, *90*, 2154–2161. (d) Schlegel, H. B.; Gonzalez, C. Reaction path following in mass-weighted internal coordinates. *J. Phys. Chem.* **1990**, *94*, 5523–5527.

(6) (a) Matsumoto, T.; Yamano, A.; Sato, T.; Ferrara, J. D.; White, F. J.; Meyer, M. *J. Chem. Crystallogr.* **2021**, *51*, 438-450. (b) Sheldrick, G. M. *Acta Cryst.* **2015**, *A71*, 3–8.

(7) Dolomanov, O. V.; Bourhis, L. J.; Gildea, R. J.; Howard, J. A. K.; Puschmann, H. *J. Appl. Crystallogr.* **2009**, *42*, 339–341.
