# Supplementary material for: Dearomative triple elementalization of quinolines driven by visible light
Source: Nat Commun. 2023 Feb 6;14:652. doi: 10.1038/s41467-023-36161-4 (PMC9902486; doi:10.1038/s41467-023-36161-4)
Supplement: Supplementary file 2 — Description of Additional Supplementary Files [file 41467_2023_36161_MOESM2_ESM.pdf]

Description of Additional Supplementary Files

Supplementary Data 1: Cartesian Coordinates of the structures.
