# Supplementary material for: Dearomative triple elementalization of quinolines driven by visible light
Source: Nat Commun. 2023 Feb 6;14:652. doi: 10.1038/s41467-023-36161-4 (PMC9902486; doi:10.1038/s41467-023-36161-4)
Supplement: Supplementary file 3 — Supplementary Data 1 [file 41467_2023_36161_MOESM3_ESM.pdf]

Supplementary data 1

Dearomative triple elementalization of quinolines driven by visible light

Shiho Ishigaki,[†] Yuki Nagashima,*[,†,‡] Daiki Yukimori,[‡] Jin Tanaka,[†] Takashi Matsumoto,[§] Kazunori Miyamoto,[‡] Masanobu Uchiyama*[,‡,⊥], and Ken Tanaka*[,†]

[†] Department of Chemical Science and Engineering, Tokyo Institute of Technology, O-okayama, Meguro-ku, Tokyo 152-8550, Japan
[‡] Graduate School of Pharmaceutical Sciences, The University of Tokyo, 7-3-1 Hongo, Bunkyo-ku, Tokyo 113-0033, Japan
[§] Rigaku Corporation, 3-9-12 Matsubara-cho, Akishima, Tokyo 196-8666, Japan
[⊥] Research Initiative for Supra-Materials (RISM), Shinshu University, 3-15-1 Tokida, Ueda, Nagano 386-8567, Japan

Table of Contents

1. Cartesian Coordinates of the Computed Structures S-1

quinoline

C	-2.40613700	0.69969600	0.00002000
C	-1.22715800	1.40358900	0.00019400
C	0.01408000	0.72259500	0.00013100
C	0.02905200	-0.70216000	-0.00011500
C	-1.20231600	-1.40129000	-0.00029600
C	-2.39167600	-0.71469100	-0.00022900
H	1.28387100	2.47953200	0.00051000
H	-3.35796900	1.22830100	0.00007500
H	-1.22769600	2.49439500	0.00038900
C	1.26130700	1.38895800	0.00030500
H	-1.16481000	-2.48928400	-0.00049100
H	-3.33450700	-1.25956100	-0.00036600
C	2.31851100	-0.75963000	-0.00006000
C	2.41738400	0.65208800	0.00021500
H	3.23067600	-1.36103600	-0.00009100
H	3.39583500	1.12794800	0.00035400
N	1.18518800	-1.42217500	-0.00019500

INT-A’

C	-4.32964000	-1.24079600	0.03985900
C	-3.96522600	-0.09849700	0.73605500
C	-2.66436400	0.41323700	0.64786600
C	-1.69542400	-0.23971800	-0.16427800
C	-2.07956900	-1.40039300	-0.85573500
C	-3.37697300	-1.88521000	-0.75358700
H	-2.88419300	1.95819800	2.19382700
H	-5.34525500	-1.62642200	0.10659500
H	-4.69162700	0.42298600	1.36185100
C	-2.24008400	1.58965400	1.39279300
H	-1.34561600	-1.88541100	-1.49454800
H	-3.65521800	-2.77574200	-1.31624700
C	-1.08785100	2.21081800	1.07764200
H	-0.76143800	3.10167700	1.61653100
C	-0.26806200	1.70395300	-0.08237200
N	-0.37749000	0.24375700	-0.14941300
C	0.69942400	-3.02102300	0.41337900
C	1.25499200	-2.85851700	-1.00416300
H	1.32397900	-3.67485300	1.04013100
H	-0.32582100	-3.43032600	0.39740800
H	2.35377700	-2.97664100	-1.00487100
H	0.83814200	-3.59858400	-1.70358600
Si	2.65691100	0.22343200	0.21025900
C	4.03223200	-1.08432500	0.24240600
H	4.97522400	-0.64065000	0.59593900
H	3.78156100	-1.91617800	0.91653100
H	4.21732500	-1.50088500	-0.75786700
C	3.30561400	1.60853200	-0.91931600
H	4.32963800	1.88997900	-0.63099800
H	3.33703300	1.26738100	-1.96371100
H	2.69443800	2.52225200	-0.89237700
C	2.61272000	0.91100100	1.99776200
H	1.87790600	1.71752300	2.15029000
H	2.38860800	0.09972400	2.70970200
H	3.59337300	1.31808000	2.28681000
B	0.86873200	-0.70502500	-0.24080400
O	0.68942100	-1.70903800	0.93566600
O	0.88666600	-1.56760300	-1.40355200
C	-0.62364300	2.37851100	-1.40932500
H	0.01804700	1.92265300	-2.18028500
H	-1.66313000	2.12805700	-1.67457100
H	0.78914100	1.93022100	0.11653800
C	-0.43401000	3.88655000	-1.37703300
H	0.58605400	4.15292400	-1.06129100
H	-0.59762100	4.33166100	-2.36586400
H	-1.13422000	4.36962500	-0.68145600
Li	-0.34919700	-0.48200000	1.79772500

anthracene

C	3.66504400	0.71384900	-0.00002800
C	2.48186600	1.40823800	0.00005800
C	1.22509000	0.72296900	0.00004800
C	1.22509100	-0.72297000	0.00002600
C	2.48186800	-1.40823700	-0.00003200
C	3.66504500	-0.71384500	-0.00008100
C	0.00000000	1.40450900	0.00004000
C	0.00000100	-1.40451000	0.00003500
C	-1.22508900	-0.72297200	0.00003500
C	-1.22509000	0.72296900	0.00001300
C	-2.48186700	1.40823800	-0.00004200
H	-2.48046100	2.49615200	-0.00009700
C	-3.66504400	0.71384800	-0.00007400
C	-3.66504400	-0.71384600	-0.00001200
C	-2.48186800	-1.40823800	0.00004200
H	-0.00000700	2.49328400	0.00005900
H	4.61162000	1.24819600	-0.00006100
H	2.48045800	2.49615200	0.00012300
H	2.48046100	-2.49615100	-0.00003800
H	4.61161500	-1.24820300	-0.00015300
H	-0.00000600	-2.49328600	0.00005300
H	-4.61161700	1.24820200	-0.00011700
H	-4.61161700	-1.24820000	-0.00003200
H	-2.48046600	-2.49615200	0.00010100

INT-G’

C	-4.26963200	-3.14128500	-0.33506300
C	-4.64924300	-1.82979600	-0.44641200
C	-3.71307100	-0.76891500	-0.22985600
C	-2.34087400	-1.07143600	0.13286500
C	-2.00036400	-2.46360600	0.23413400
C	-2.92434500	-3.45457200	0.00491000
H	-5.16110000	0.77963600	-0.60156700
H	-4.98687400	-3.93952500	-0.50945400
H	-5.67082800	-1.56705400	-0.71328100
C	-4.12233300	0.56184000	-0.35905500
H	-0.98800900	-2.72837700	0.50408500
H	-2.62120600	-4.49577000	0.08363900
C	-3.22474800	1.61589200	-0.18837400
C	-1.83755400	1.32353700	0.13033400
C	1.65353200	0.02922600	2.63943600
C	1.56051400	-1.47055900	2.25912700
H	2.65526700	0.43867300	2.46901600
H	1.37823700	0.20765300	3.68893700
H	2.43981800	-1.77153500	1.67215300
H	1.49060700	-2.12380400	3.13694500
Si	1.36971300	-0.36628200	-1.03466700
C	1.19773200	-2.06509000	-1.88569100
H	1.83420500	-2.13903000	-2.77762500
H	1.45712300	-2.89126400	-1.21207700
H	0.16026500	-2.22593400	-2.20563900
C	0.90081000	0.94196200	-2.34781300
H	-0.16416900	0.88246000	-2.60595700
H	1.10000800	1.96844500	-2.01732700
H	1.47852800	0.77695600	-3.26710800
B	0.20119000	-0.34938000	0.72629300
O	0.70971200	0.69127300	1.78792500
O	0.37523200	-1.59244900	1.49856900
Li	-0.96162800	1.15206200	2.19820900
C	-1.38289000	-0.02397000	0.34463700
C	-3.65695600	2.97346600	-0.32795600
H	-4.70466300	3.15353000	-0.56016500

C	-0.95824800	2.46753600	0.22753200
H	0.10200600	2.30340700	0.38185700
C	-2.78687800	4.02286000	-0.18377600
H	-3.13194900	5.04733300	-0.29453000
C	-1.41510100	3.75826200	0.08507700
H	-0.71069100	4.58408200	0.14847100
C	3.23297600	-0.09045200	-0.67995100
C	4.18194400	-1.12777800	-0.76916200
C	3.71490200	1.17978400	-0.30179200
C	5.53739300	-0.91392600	-0.49501700
H	3.86284300	-2.12545200	-1.06388700
C	5.06653400	1.40602600	-0.02713000
H	3.02152200	2.01578400	-0.22018000
C	5.98541700	0.35569900	-0.12088000
H	6.24313000	-1.73807400	-0.57774900
H	5.40375200	2.40076800	0.25764000
H	7.03847400	0.52693400	0.09034700

IM1

C	-3.90921300	1.91300200	-1.11296700
C	-3.48883000	1.94942400	0.21589400
C	-2.17030800	1.63108000	0.57463400
C	-1.22325100	1.24617000	-0.42743800
C	-1.66868000	1.21769100	-1.76563000
C	-2.98361400	1.54772900	-2.09718100
H	-2.48305500	1.72110000	2.74823800
H	-4.92840200	2.18134200	-1.37853000
H	-4.18472800	2.24290600	1.00085200
C	-1.72942300	1.66175600	1.96363700
H	-0.95461300	0.99214900	-2.54429400
H	-3.27947900	1.54052400	-3.14434500
C	-0.41583000	1.63597100	2.25079700
H	-0.06013700	1.68089000	3.27826000
C	0.59248400	1.64306000	1.12157000
N	0.07591600	0.86138600	-0.02654600
C	0.42142100	-1.49059100	-2.67337900
C	1.23297900	-0.32046800	-3.28313200
H	0.86779700	-2.46825900	-2.90118100
H	-0.61401700	-1.49133100	-3.04700300
H	2.25675500	-0.64065100	-3.52598800
H	0.77483100	0.05895600	-4.20721300
Si	3.01477700	-0.25542300	-0.18932600
C	3.92289700	-1.47334000	-1.36113500
H	4.88635000	-1.77066100	-0.92330700
H	3.34392800	-2.38937900	-1.53742600
H	4.13711800	-1.02045000	-2.33726200
C	4.15267300	1.28362800	-0.12153300
H	5.19585200	0.98618600	0.05621600
H	4.12001900	1.83214600	-1.07183000
H	3.87460200	1.98765600	0.67267000
C	3.17966700	-1.10508600	1.53024600
H	2.76504900	-0.51295500	2.35657800
H	2.67423500	-2.07934900	1.53371100
H	4.23962200	-1.28488400	1.75959900
B	1.09113700	0.10351300	-0.96590200
O	0.43032200	-1.26122300	-1.27090300
O	1.23508700	0.69630200	-2.30486600
C	0.98862600	3.08672300	0.71614300
H	1.67939000	3.01427700	-0.13237900
H	0.09406300	3.60839800	0.35242200
H	1.49916000	1.13578800	1.45973000
C	1.63370900	3.89406600	1.84954700
H	2.52132800	3.38631700	2.25019300
H	1.95434800	4.87918000	1.49068300
H	0.93729800	4.05796000	2.68086400
C	-3.19652800	-2.16464100	-1.22746900
H	-3.80063200	-3.08285400	-1.23531500
H	-3.83274100	-1.29569900	-1.43348600
H	-2.41749800	-2.23261000	-1.98731500
C	-3.46801800	-1.95228600	1.11727400
H	-4.07314500	-2.86901600	1.16042200
H	-2.88542100	-1.85839700	2.03525200
H	-4.12605500	-1.08086700	1.00720900
C	-0.03032900	-2.09828700	3.17842900
H	-0.70867900	-2.70970200	3.79128200
H	1.00575600	-2.27296200	3.49486500
H	-0.27550300	-1.04133400	3.29724900
C	0.10393800	-3.77506400	1.49824900
H	-0.00994900	-3.90313900	0.42031400
H	1.13527100	-4.01712600	1.78668800
H	-0.59084900	-4.44355800	2.02608700
O	-0.19108000	-2.41196400	1.79690500
O	-2.54254000	-2.02050100	0.03475800
Li	-0.68412600	-1.27202000	0.22896700

TS1_2

C	-4.58742600	0.10493800	1.05627300
C	-4.15194800	0.26337500	-0.25879900
C	-2.84718700	-0.08033800	-0.64269200
C	-1.92934700	-0.58246800	0.32645100
C	-2.38583900	-0.74635200	1.64723800
C	-3.69410000	-0.40949500	2.00109600
H	-3.02058000	0.61997500	-2.71632600
H	-5.60517400	0.36347400	1.33580800
H	-4.83286100	0.64580500	-1.01742600
C	-2.40302100	0.04643400	-2.02673100
H	-1.70675600	-1.12831000	2.39726600
H	-4.01674400	-0.56034200	3.02897400
C	-1.27831200	-0.56242000	-2.43625600
H	-0.95524200	-0.51065000	-3.47294600
C	-0.48787300	-1.41661900	-1.47213400
N	-0.59913900	-0.85102700	-0.09506500
C	1.35693100	-1.67461700	2.90569600
C	1.59427100	-2.88200600	1.97380200
H	2.28616900	-1.13465900	3.11928600
H	0.88293200	-1.95424500	3.85347600
H	2.63787800	-3.21364500	1.96681200
H	0.95771300	-3.73733100	2.24087800
Si	2.81247000	0.13904100	-0.19303500
C	4.12411100	-0.45897000	1.09721900
H	5.13495900	-0.27222500	0.70407600
H	4.04685300	0.07650500	2.05384800
H	4.05382800	-1.53285000	1.30781400
C	3.35617100	-0.81342500	-1.78703900
H	4.43767400	-0.70352900	-1.96117500
H	3.14190000	-1.88669000	-1.69870000
H	2.84608000	-0.45094200	-2.69161200
C	3.51006400	1.93060900	-0.53667600
H	3.00516000	2.44029400	-1.36691500
H	3.42227700	2.58486700	0.34309500
H	4.58061500	1.87391300	-0.78926300
B	0.54475300	-1.19196400	0.81711800
O	0.46723200	-0.82414800	2.17493800
O	1.22367400	-2.42070400	0.67201800
C	-0.92919600	-2.90177600	-1.54978400
H	-0.38376800	-3.45109500	-0.77667900
H	-1.99919200	-2.96562800	-1.31193200
H	0.57691100	-1.36695200	-1.72481500
C	-0.66081800	-3.53948200	-2.91928200
H	0.40249900	-3.47545200	-3.18494200
H	-0.93401800	-4.60124900	-2.90989000
H	-1.23885100	-3.06026900	-3.71871200

C	0.76118300	2.44480000	2.53430200
H	0.78963600	3.48245500	2.89564900
H	0.48024600	1.76843400	3.34981900
H	1.73990000	2.15782500	2.14870800
C	-1.49709600	2.67991700	1.84333300
H	-1.53666700	3.73749800	2.14237600
H	-2.14940800	2.51140400	0.98460500
H	-1.83419400	2.04935800	2.67382300
C	0.62704100	2.27394600	-2.92958100
H	-0.21155700	2.43313500	-3.62091700
H	1.46930600	2.91704300	-3.21756900
H	0.94149500	1.23115200	-2.96734800
C	-0.10146300	3.92730700	-1.40392100
H	-0.34407500	4.06242100	-0.35024800
H	0.75523800	4.56363400	-1.66569400
H	-0.96364300	4.20431500	-2.02719200
O	0.22655200	2.55279100	-1.58900200
O	-0.16880400	2.32870000	1.45393600
Li	0.33099100	1.17991600	-0.10128800

IM2

C	-0.48271500	-1.67460700	-3.32596300
C	0.01951800	-1.93110200	-2.05042900
C	-0.68618700	-1.54166800	-0.90162700
C	-1.92692100	-0.86939700	-1.04117100
C	-2.42525200	-0.61249400	-2.32648800
C	-1.70745700	-1.01156600	-3.45661500
H	0.86768400	-2.10100900	0.54573400
H	0.06880000	-1.99371100	-4.20635100
H	0.97213300	-2.44177700	-1.92826900
C	-0.17170400	-1.79664500	0.44202200
H	-3.37302400	-0.09937700	-2.44081800
H	-2.11745600	-0.81146300	-4.44357400
C	-0.97360100	-1.66867200	1.51184400
H	-0.60860800	-1.87382700	2.51535500
C	-2.43205700	-1.30789300	1.34374100
C	-4.62292200	2.55561600	-0.31293400
C	-4.69730600	2.31376000	1.20999600
H	-4.31381300	3.57201000	-0.57409100
H	-5.57307900	2.33398900	-0.81466300
H	-4.10387400	3.04359900	1.77492300
H	-5.72034900	2.31381000	1.59740300
Si	4.47322500	-0.60289100	0.40994000
C	5.56558400	-0.34609100	1.99750500
H	6.36511800	-1.10112700	2.06825100
H	6.04732800	0.64269300	2.00119800
H	4.96676000	-0.41765500	2.91771200
C	3.99226900	-2.47978700	0.57819700
H	4.87996200	-3.11373900	0.73397500
H	3.31626800	-2.65249600	1.42882700
H	3.48186200	-2.85129300	-0.32286700
C	5.82616300	-0.67866400	-0.98350500
H	5.39541600	-0.96682200	-1.95410300
H	6.31583600	0.29612600	-1.12370000
H	6.61445900	-1.41131500	-0.74666400
B	-3.43392700	0.69221100	0.22941000
O	-3.62904800	1.62665800	-0.77589900
O	-4.12344300	1.01049700	1.39189000
C	-3.34859500	-2.55556500	1.29335400
H	-4.37310900	-2.20555300	1.10916200
H	-3.05966100	-3.17379200	0.43360600
H	-2.75184500	-0.69779400	2.19354700
C	-3.31384400	-3.39405700	2.57674700
H	-3.58262300	-2.79095100	3.45427300
H	-4.02727000	-4.22387600	2.51573100
H	-2.32223300	-3.82579000	2.75429000

N	-2.61112800	-0.46382100	0.13343700
Li	2.56588000	1.09612700	0.06029800
O	1.83143200	1.80380800	-1.57901600
O	1.84065000	2.26699300	1.48908200
C	0.51650300	2.34756500	-1.71546900
H	-0.16673600	1.60472400	-2.14445600
H	0.17935200	2.62206400	-0.71413900
H	0.54034200	3.24049400	-2.35554000
C	2.40613700	1.40979700	-2.82927400
H	2.51345400	2.28404800	-3.48596500
H	3.38686700	0.98515400	-2.60772600
H	1.77596500	0.65497800	-3.31617300
C	2.60526200	3.45068000	1.72332500
H	3.54293100	3.21061900	2.24171700
H	2.82822000	3.88928100	0.74782700
H	2.02440400	4.16563500	2.32190100
C	1.46952400	1.60404500	2.70204100
H	0.87875600	0.72972600	2.42184900
H	2.36194200	1.28385400	3.25446800
H	0.86345600	2.27518200	3.32594800

TS2_3

C	-3.14672500	-3.28054200	-0.02541900
C	-1.79642700	-3.30115900	0.30690600
C	-1.06756300	-2.10976800	0.58835500
C	-1.79228100	-0.87757700	0.48094100
C	-3.15076900	-0.87106300	0.17529100
C	-3.84049600	-2.06477900	-0.09129700
H	0.81766300	-3.07535200	1.01749400
H	-3.66544600	-4.21686700	-0.22268600
H	-1.27203100	-4.25242500	0.38582900
C	0.29614200	-2.12096000	0.98874800
H	-3.68307000	0.07116700	0.11686600
H	-4.89945700	-2.03636100	-0.33248200
C	0.97812500	-0.93752600	1.38925900
H	1.73169400	-1.08642000	2.16460200
C	0.04429600	0.22204700	1.75372300
C	-2.83553600	3.35585000	-0.33795900
C	-1.66797900	3.93150300	0.49719700
H	-2.91092700	3.79974400	-1.33546100
H	-3.80175400	3.46462700	0.16941300
H	-0.94713500	4.48261800	-0.11866300
H	-2.00271800	4.58264500	1.31033100
Si	3.03419100	-0.33325900	0.34170700
C	3.78395700	0.91177500	1.59377100
H	4.87756700	0.80609600	1.64218800
H	3.56844000	1.95116900	1.31224700
H	3.39832800	0.76286300	2.61096900
C	3.83435600	-2.03307000	0.72950400
H	4.92370900	-1.98334100	0.59344800
H	3.64592700	-2.36857300	1.75744600
H	3.45766300	-2.81991200	0.06135700
C	3.86335000	0.10290100	-1.35979500
H	3.51211800	-0.54597000	-2.17301700
H	3.68496500	1.13985200	-1.66441500
H	4.95298300	-0.03681500	-1.28068400
B	-1.53360400	1.64778400	0.43614000
O	-2.53778900	1.95849400	-0.46650400
O	-1.01923300	2.78094600	1.05619000
C	-0.59221100	0.06964800	3.15581200
H	-1.30884700	0.89360800	3.27670400
H	-1.16588900	-0.86320500	3.19109300
H	0.59401600	1.16645400	1.73958900
C	0.41371200	0.11133300	4.31218500
H	1.02880100	1.02042800	4.27754400
H	-0.11005600	0.10336100	5.27566800

H	1.08753000	-0.75273800	4.30142000
N	-1.02477400	0.33796400	0.70081400
Li	0.54756000	-0.11912100	-0.85855100
O	0.09078900	-1.26918600	-2.40512600
O	0.86799200	1.65387000	-1.92667700
C	-1.25116100	-1.25578200	-2.91502200
H	-1.87570000	-1.97536700	-2.37587500
H	-1.64683600	-0.25013100	-2.76049700
H	-1.24116000	-1.49082700	-3.98875900
C	0.70679800	-2.55282400	-2.54127100
H	0.77361900	-2.82518000	-3.60374700
H	1.71100200	-2.47809100	-2.11972200
H	0.13302600	-3.31089500	-1.99588500
C	0.98844500	1.67501500	-3.34906700
H	2.03322000	1.84569400	-3.64246300
H	0.66393300	0.70129900	-3.71410500
H	0.35679500	2.46817400	-3.77351600
C	1.34650800	2.86218000	-1.33769100
H	1.22333300	2.77513200	-0.25806400
H	2.40653200	3.01633400	-1.57837000
H	0.76691000	3.71932100	-1.70853300

IM3

C	2.27317700	-3.06485400	-2.02378300
C	1.16126300	-3.04262700	-1.18227400
C	0.48465900	-1.83312100	-0.87287800
C	1.00945400	-0.64431400	-1.46489500
C	2.10005000	-0.67338500	-2.33290600
C	2.75026100	-1.88349300	-2.61112500
H	-1.04445700	-2.62829700	0.36998300
H	2.75799800	-4.01385600	-2.24774400
H	0.77957500	-3.97347200	-0.76396100
C	-0.64313500	-1.66975400	0.01936900
H	2.45795800	0.25128800	-2.77769300
H	3.60072300	-1.90317200	-3.28752900
C	-1.74708500	-0.77769200	-0.61111000
H	-2.15438600	-1.31107200	-1.49478500
C	-1.15380700	0.55445100	-1.18236200
C	2.67862300	3.34737900	-0.67597600
C	1.31771000	4.00368300	-0.36186600
H	3.43674500	3.53853900	0.09014500
H	3.07368500	3.66486200	-1.64968100
H	1.20568800	4.22805900	0.70735300
H	1.13779900	4.91953700	-0.93319300
Si	-3.26698900	-0.64258300	0.56254300
C	-4.04994500	1.09395800	0.64093900
H	-4.88488600	1.08632400	1.35474200
H	-3.34388200	1.86448200	0.97668800
H	-4.44965900	1.41577200	-0.32725100
C	-4.59951200	-1.84981700	-0.05491200
H	-5.46815200	-1.86765800	0.61642900
H	-4.95954500	-1.57860600	-1.05581900
H	-4.20557000	-2.87282700	-0.11560600
C	-2.85217100	-1.12845900	2.35405100
H	-2.48594100	-2.15957900	2.41506600
H	-2.07889100	-0.48514800	2.78998900
H	-3.75005300	-1.05011100	2.98254500
B	1.01759300	1.79730700	-0.87085200
O	2.39586100	1.94272600	-0.72508800
O	0.35015600	3.01448900	-0.73569600
C	-1.53119900	0.80440700	-2.66021400
H	-0.98123000	1.69108400	-3.00305200
H	-1.16574400	-0.04396300	-3.25418700
H	-1.49175700	1.41955600	-0.60195600
C	-3.02345900	1.02089500	-2.93454900
H	-3.40744400	1.89187200	-2.38928700
H	-3.19221400	1.20091600	-4.00346500
H	-3.62656700	0.15088400	-2.65071700
N	0.34619700	0.56757900	-1.06747400
Li	0.66720600	-0.52587300	1.24170800
O	2.39627400	-1.32243600	1.86331100
O	0.51335900	0.94143600	2.63721800
C	3.58063600	-0.97154000	1.13305600
H	3.66237300	-1.57657400	0.22302400
H	3.48984100	0.08027500	0.85615200
H	4.46599800	-1.11960400	1.76738600
C	2.38014400	-2.69913100	2.24546700
H	3.23661100	-2.92019200	2.89807400
H	1.44921500	-2.87328600	2.79013000
H	2.41002500	-3.34287500	1.35860200
C	1.08061200	0.76863300	3.93607400
H	0.29892200	0.52029000	4.66766400
H	1.80050400	-0.04667100	3.86314300
H	1.59313400	1.68782000	4.25287300
C	-0.41953300	2.02174700	2.59752700
H	-0.76802800	2.11802400	1.56933500
H	-1.26998800	1.82404400	3.26371100
H	0.07082600	2.95601500	2.90498100

[1]IM1*

C	3.75819100	2.11228900	1.03269800
C	3.38319400	1.99758200	-0.30033200
C	2.05622600	1.66091500	-0.66558000
C	1.07054300	1.41842300	0.36682600
C	1.47692600	1.56642900	1.70626500
C	2.79279300	1.90655600	2.03173000
H	2.32023300	1.41906900	-2.83661100
H	4.77702900	2.38448900	1.29746400
H	4.10635800	2.18099600	-1.09344200
C	1.61491900	1.48736200	-2.01100700
H	0.73656300	1.44002400	2.48457400
H	3.06218600	2.03209700	3.07825100
C	0.20426800	1.96312300	-2.23729500
H	0.12710700	2.98142800	-2.63073300
C	-0.81994700	1.61451600	-1.16561600
N	-0.20165800	0.93239200	-0.01049900
C	-0.20452000	-1.34317500	2.71551100
C	-1.18196600	-0.28523500	3.28446300
H	-0.50023100	-2.36734600	2.98110900
H	0.81753000	-1.17175600	3.08564900
H	-2.14707400	-0.74362300	3.54598400
H	-0.78471500	0.19251200	4.19093300
Si	-2.94631900	-0.63131600	0.21149300
C	-3.55944600	-2.02361100	1.38226400
H	-4.45710600	-2.50064500	0.96396400
H	-2.80336400	-2.80695100	1.52326100
H	-3.83044100	-1.63967200	2.37374500
C	-4.36162800	0.65885300	0.22380600
H	-5.33532100	0.16205800	0.10819300
H	-4.37903800	1.20944000	1.17327100
H	-4.27509400	1.39675900	-0.58308700
C	-3.02800200	-1.46670900	-1.52088300
H	-2.78945400	-0.78829300	-2.35015500
H	-2.34120700	-2.32069100	-1.57979200
H	-4.04241500	-1.85119600	-1.69895200
B	-1.09846200	0.06571100	0.95074300
O	-0.24300400	-1.17088600	1.30489200
O	-1.33434200	0.68155100	2.26845200
C	-1.58114200	2.88880700	-0.70591000
H	-2.21367100	2.61067300	0.14278100
H	-0.84845700	3.61299100	-0.32420400
H	-1.56255000	0.92373200	-1.58388500

C	-2.44434500	3.53469600	-1.79595200
H	-3.17823700	2.82364700	-2.19660100
H	-3.00093100	4.38756300	-1.38919700
H	-1.84991800	3.90603000	-2.63956600
C	3.37855100	-1.92994900	1.35118400
H	3.98229600	-2.84739800	1.39470300
H	4.01142500	-1.05773700	1.55641500
H	2.57950400	-1.98216600	2.09071000
C	3.71584400	-1.76596100	-0.98808700
H	4.31285000	-2.68903100	-1.00093400
H	3.15911500	-1.67625900	-1.92182300
H	4.37993800	-0.90010100	-0.87021100
C	0.28063600	-1.74742900	-3.18200300
H	1.01734800	-2.17826800	-3.87545300
H	-0.73030800	-2.03246700	-3.49771700
H	0.37101300	-0.66031500	-3.17783800
C	0.42924800	-3.61226900	-1.71316200
H	0.61651800	-3.85160500	-0.66474300
H	-0.57597500	-3.95355900	-1.99281700
H	1.17470200	-4.11621100	-2.34484900
O	0.53366100	-2.19658600	-1.85048900
O	2.75999300	-1.80979800	0.06919000
Li	0.87787900	-1.13020900	-0.18980400

^{3}IM1*

C	2.81428500	2.89358100	0.96796800
C	1.96043300	3.34640800	0.00041100
C	0.64823500	2.75694400	-0.19930700
C	0.32139200	1.54476500	0.60586300
C	1.22161000	1.15228100	1.61971800
C	2.44449100	1.80232900	1.82237300
H	-0.00875000	4.11004400	-1.71978900
H	3.77639900	3.38256800	1.10975000
H	2.23318400	4.19535600	-0.62371700
C	-0.27519500	3.25473800	-1.10073700
H	0.91426600	0.37476100	2.30612800
H	3.08349900	1.51416700	2.65205700
C	-1.56261100	2.67858000	-1.20339800
H	-2.32675400	3.11351800	-1.83816000
C	-1.95535000	1.57968800	-0.27714200
N	-0.82966100	0.84165200	0.32137500
C	0.35157400	-2.16759400	1.96341000
C	-0.97970600	-1.92457500	2.71133600
H	0.52583500	-3.23171600	1.75458600
H	1.20443900	-1.78343100	2.54392500
H	-1.67006100	-2.76914000	2.56119700
H	-0.83071400	-1.79676000	3.79084100
Si	-2.38475100	-1.72157600	-0.53084900
C	-2.16493500	-3.60780800	-0.25590900
H	-2.73755800	-4.17227400	-1.00545300
H	-1.11362400	-3.91107200	-0.34498400
H	-2.52121900	-3.92840100	0.73151400
C	-4.25561900	-1.41070300	-0.26026400
H	-4.85511700	-2.12643100	-0.84024800
H	-4.51990200	-1.53641100	0.79792500
H	-4.57490100	-0.40349300	-0.55629900
C	-2.04835600	-1.48897500	-2.40731500
H	-2.15743000	-0.44915000	-2.74135300
H	-1.02842300	-1.81031400	-2.65769900
H	-2.73918900	-2.10152000	-3.00361100
B	-1.09057200	-0.66398300	0.75288200
O	0.23503000	-1.44468500	0.73676300
O	-1.49410000	-0.73381200	2.16157600
C	-2.85639300	2.10374900	0.90507200
H	-3.07190700	1.25088300	1.55746000
H	-2.25779500	2.81387900	1.48848200

H	-2.56735400	0.85256700	-0.81979800
C	-4.16240100	2.75819200	0.44609500
H	-4.75155900	2.08203300	-0.18704800
H	-4.78144500	3.01409500	1.31474300
H	-3.98288800	3.68002100	-0.11856300
C	3.96885000	-2.21929800	1.19569600
H	4.82174600	-2.81206100	0.83747300
H	4.25239600	-1.69393800	2.11777700
H	3.12448900	-2.88049200	1.39549600
C	4.61058600	-0.40691300	-0.19615400
H	5.45367900	-0.98100800	-0.60455700
H	4.20825000	0.25523900	-0.96399900
H	4.94635700	0.19026300	0.66064800
C	1.35049600	0.42278000	-3.01595000
H	2.12796800	0.69070000	-3.74477400
H	0.46102400	0.05664500	-3.54242900
H	1.08536700	1.29420400	-2.41698700
C	2.20415300	-1.78463600	-2.80305900
H	2.58041900	-2.48383700	-2.05422700
H	1.32960300	-2.21581900	-3.30731300
H	2.99022900	-1.58469500	-3.54482400
O	1.84727500	-0.58201200	-2.12261100
O	3.55686300	-1.29179300	0.19344200
Li	1.68355000	-0.65045100	-0.14397700

^{3}TS1_4*

C	-1.30259100	-3.44849000	-1.04979900
C	-0.08969400	-3.18614200	-1.65459600
C	0.86546200	-2.26663300	-1.09280200
C	0.43552100	-1.54655700	0.09324900
C	-0.75622700	-1.88226500	0.73266000
C	-1.67000000	-2.81188800	0.17232200
H	2.45342500	-2.51231800	-2.53311600
H	-1.97796000	-4.17464500	-1.50021700
H	0.17876300	-3.70713700	-2.57311700
C	2.17245200	-2.04867100	-1.58707500
H	-0.95454500	-1.44652200	1.71126500
H	-2.55385200	-3.12272400	0.72237400
C	3.10042500	-1.29534500	-0.88273200
H	4.11429600	-1.15618200	-1.24604900
C	2.74755100	-0.79855600	0.49551800
N	1.28285100	-0.52323100	0.58752100
C	-0.75409100	2.04741500	2.16058500
C	0.66939300	2.56777600	2.44127200
H	-1.44741200	2.83660400	1.85006400
H	-1.17267600	1.53549200	3.03727000
H	0.85964000	3.51616400	1.92225300
H	0.85782100	2.71371000	3.51028900
Si	1.42557800	2.07730400	-1.10396200
C	0.53920900	3.77157600	-0.97824800
H	0.65870500	4.31424100	-1.92838700
H	-0.53676600	3.67097000	-0.79047500
H	0.96301900	4.40342900	-0.18873200
C	3.29305000	2.42028300	-1.15335000
H	3.51477000	3.18222900	-1.91565200
H	3.65922100	2.79307700	-0.18945300
H	3.85160300	1.51287200	-1.40525200
C	0.85904500	1.26069800	-2.71936100
H	1.28545100	0.25803500	-2.82054600
H	-0.23195300	1.16979400	-2.75993100
H	1.17926400	1.86864800	-3.57898600
B	0.83012500	0.79686100	0.97434800
O	-0.59474000	1.09642700	1.09417900
O	1.53218600	1.55192400	1.95000900
C	3.13387000	-1.78270700	1.63415500
H	2.74827900	-1.37866100	2.58120400

H	2.62734400	-2.73956500	1.45732200
H	3.25770400	0.14814800	0.70555700
C	4.64581300	-2.00546700	1.74792500
H	5.17658800	-1.05883300	1.91948600
H	4.87929800	-2.67266800	2.58711400
H	5.05080600	-2.45847000	0.83583900
C	-3.55256800	-0.52224900	2.78825900
H	-4.31862400	-0.02059800	3.39517700
H	-3.69504000	-1.60972800	2.84358000
H	-2.56284600	-0.26728600	3.16921700
C	-4.88787800	-0.36335400	0.84269400
H	-5.68926600	0.15034800	1.39144700
H	-4.85261600	-0.00299700	-0.18552500
H	-5.07623300	-1.44534200	0.84364300
C	-2.75299600	-0.40067300	-2.60991200
H	-3.79968000	-0.52872300	-2.91950500
H	-2.16606800	-0.01411900	-3.45199000
H	-2.33668300	-1.35357200	-2.27934200
C	-3.12184900	1.82025100	-1.82118000
H	-3.03000600	2.42777200	-0.91808000
H	-2.50210000	2.25407800	-2.61647000
H	-4.17147000	1.80498400	-2.14840400
O	-2.67817300	0.50506700	-1.49938000
O	-3.62062700	-0.07419400	1.43418600
Li	-1.96047400	-0.03408100	0.24924800

^{3}IM4*

C	-1.87190700	-2.26132000	-2.49459800
C	-0.54810100	-1.91667300	-2.69884500
C	0.33518600	-1.59462900	-1.61389300
C	-0.24193300	-1.63409600	-0.28643300
C	-1.55193500	-2.05474400	-0.08834800
C	-2.41330900	-2.33231400	-1.18178200
H	2.11784600	-1.18135000	-2.75323100
H	-2.50029200	-2.50739800	-3.34883100
H	-0.14714300	-1.89318000	-3.71175100
C	1.71272300	-1.29662100	-1.74806800
H	-1.91170600	-2.17971200	0.93284000
H	-3.41697000	-2.71069800	-1.00970800
C	2.55612700	-1.18224700	-0.64488500
H	3.62897200	-1.08143800	-0.77171200
C	2.02836500	-1.56922000	0.71466500
C	-1.28017400	1.20991500	2.81094800
C	0.15462300	1.34949600	3.35078400
H	-1.74239900	2.16584600	2.54810100
H	-1.93289500	0.67849000	3.51545200
H	0.63621700	2.26806300	2.99085900
H	0.20350900	1.32732800	4.44352600
Si	2.79672800	1.81964800	-0.59118900
C	2.21177100	3.65519400	-0.43979500
H	3.07414300	4.34140800	-0.46258500
H	1.54655800	3.94026500	-1.26540100
H	1.67494300	3.83574900	0.50104600
C	4.04253200	1.57435500	0.83832500
H	4.78376800	2.38833600	0.82871400
H	3.54615600	1.58667300	1.81617100
H	4.58478200	0.62627000	0.75423700
C	3.73555600	1.72570600	-2.25166100
H	4.20251800	0.74675600	-2.40391000
H	3.06660500	1.91002500	-3.10147700
H	4.52852100	2.48862500	-2.28137300
B	0.13923900	-0.24029300	1.71537200
O	-1.12673600	0.40743200	1.62442600
O	0.84463200	0.21661900	2.81958100
C	2.19111900	-3.06960600	1.06141900
H	1.68886900	-3.25418200	2.02313700
H	1.66617700	-3.66656000	0.30477200
H	2.53659900	-0.99369000	1.49707700
C	3.65374400	-3.51826400	1.15345700
H	4.20501100	-2.93073600	1.90063500
H	3.72107700	-4.57330200	1.44659100
H	4.16682500	-3.40486500	0.19166700
N	0.57967700	-1.19836700	0.79747900
Li	-2.20812800	0.14972600	-0.03677600
O	-4.11462000	0.31311100	0.55109500
O	-2.04006600	1.68381600	-1.25873000
C	-4.60770800	-0.51764300	1.60123500
H	-4.94061900	-1.48649800	1.20537600
H	-3.78674200	-0.67438400	2.30313000
H	-5.44418200	-0.02593000	2.11639400
C	-5.10700300	0.58226400	-0.44209400
H	-5.96062100	1.10703200	0.00836800
H	-4.63758000	1.21389400	-1.19736300
H	-5.45027900	-0.35202400	-0.90572600
C	-1.94037500	2.99177400	-0.69751100
H	-2.38562000	3.73201300	-1.37675300
H	-2.49772800	2.98623800	0.24242900
H	-0.89187700	3.25379800	-0.50824300
C	-1.39474700	1.58603700	-2.53803400
H	-1.52106400	0.55943000	-2.88337700
H	-1.86296900	2.28758700	-3.24192100
H	-0.32514200	1.80588300	-2.43969200

^{3}TS4_5*

C	-0.41083500	-3.91991300	-1.39774700
C	0.56287900	-3.05328000	-1.86554100
C	1.06409500	-1.98134500	-1.07241900
C	0.48415800	-1.79738400	0.23981600
C	-0.48215800	-2.69024000	0.70399300
C	-0.94720900	-3.74574700	-0.09750200
H	2.53790300	-1.26533500	-2.47636100
H	-0.74393900	-4.74902800	-2.01842400
H	0.99358100	-3.20396200	-2.85466800
C	2.14979200	-1.15701900	-1.46418400
H	-0.86402700	-2.57985400	1.71672300
H	-1.66434300	-4.45676800	0.30480500
C	2.72199500	-0.21486800	-0.59055100
H	3.71672200	0.15725600	-0.82242600
C	2.39942900	-0.35127300	0.88166900
C	-1.68759300	0.70596600	3.13118200
C	-0.42651800	1.42753200	3.63522800
H	-2.52521900	1.37883200	2.91992800
H	-2.01947000	-0.08048000	3.81852400
H	-0.38918500	2.47183700	3.29607400
H	-0.32854700	1.40334800	4.72418800
Si	1.78363700	2.04241700	-1.45389100
C	1.62293000	3.90684100	-0.95640100
H	1.44054100	4.52933500	-1.85128200
H	0.80847100	4.08628300	-0.24563900
H	2.54842400	4.26453400	-0.48700800
C	3.20106800	2.07972700	-2.74094600
H	3.00372600	2.85330100	-3.49776900
H	4.16548300	2.31866300	-2.27370700
H	3.31274600	1.12074000	-3.26099400
C	0.19785800	1.60103800	-2.44238900
H	0.21134500	0.55019200	-2.75454500
H	-0.71394800	1.77345200	-1.85659500
H	0.12744900	2.22619800	-3.34805000
B	0.17280900	0.00600700	1.94993600
O	-1.25563600	0.08043300	1.89901100
O	0.65372400	0.70255100	3.04579800
C	3.27951900	-1.37427500	1.63734600

H	2.89404400	-1.45416400	2.66450800
H	3.16330900	-2.36200500	1.17327400
H	2.51838900	0.62251900	1.37393800
C	4.76385100	-0.99204600	1.68463700
H	4.90368700	0.00583100	2.12194300
H	5.32854900	-1.70474900	2.29818100
H	5.21331500	-0.98619900	0.68508000
N	0.95290900	-0.71245800	1.03104200
Li	-2.12402500	0.24525300	0.20211100
O	-3.30048700	-0.87964600	-0.81410700
O	-2.84271800	2.04344700	-0.02582700
C	-4.15895300	-1.84684800	-0.19973700
H	-3.66011100	-2.82190000	-0.15528200
H	-4.37337200	-1.49496700	0.81311100
H	-5.09793200	-1.92582800	-0.76439600
C	-2.94946100	-1.23183800	-2.16417600
H	-3.85989200	-1.31817600	-2.77166700
H	-2.32437600	-0.42563600	-2.55480600
H	-2.38758600	-2.17115100	-2.17543000
C	-4.09359300	2.31911200	-0.66459100
H	-3.92920800	2.88146500	-1.59322400
H	-4.55748700	1.35773800	-0.89061500
H	-4.74084600	2.90030100	0.00555000
C	-2.13262600	3.23975800	0.31867500
H	-1.17192900	2.93888600	0.74368200
H	-1.95466100	3.84804600	-0.57665300
H	-2.70568100	3.82134900	1.05402600

^{3}IM5*

C	0.27214300	-4.16667100	0.48660400
C	-0.80561800	-3.47744600	1.01742200
C	-1.18274700	-2.18896300	0.53656500
C	-0.39063100	-1.60112800	-0.51130400
C	0.68373700	-2.32024700	-1.04315500
C	1.01918200	-3.58703700	-0.55277100
H	-2.87566700	-1.97489600	1.85077100
H	0.52776900	-5.15457300	0.86134400
H	-1.40306500	-3.92167300	1.81096000
C	-2.31276300	-1.51653500	1.04041100
H	1.26224700	-1.89731600	-1.85763100
H	1.85022200	-4.12695700	-0.99915200
C	-2.74399200	-0.19807400	0.50273300
H	-3.84440400	-0.16545400	0.46930800
C	-2.22334400	-0.00106700	-0.94082000
C	1.82966200	1.34629600	-2.97748000
C	0.62559900	2.27383700	-3.14979800
H	2.77844100	1.85646300	-2.76519500
H	1.97211300	0.66984300	-3.82752900
H	0.75537500	3.22722400	-2.62276700
H	0.38724200	2.47164800	-4.19842700
Si	-2.34075600	1.21999200	1.76707000
C	-2.63054200	2.90555200	0.94749700
H	-2.50837100	3.71282900	1.68178800
H	-1.92900900	3.09817100	0.12660300
H	-3.64650000	2.98555800	0.53929900
C	-3.55025100	1.02028700	3.21361300
H	-3.38437300	1.79896000	3.96933200
H	-4.59424200	1.10031600	2.88399300
H	-3.43187200	0.05036400	3.71297100
C	-0.57292000	1.10063300	2.42939900
H	-0.40737500	0.13967900	2.93174000
H	0.17554100	1.19336600	1.63494700
H	-0.38059600	1.89600300	3.16170300
B	0.06151800	0.55003700	-1.76124500
O	1.46843300	0.53104400	-1.81448900
O	-0.46805500	1.55355800	-2.54848800

C	-3.00238000	-0.82179300	-1.99485900
H	-2.45335100	-0.73932400	-2.94152100
H	-2.99734300	-1.88315800	-1.71730800
H	-2.31298300	1.05035100	-1.22141900
C	-4.44365900	-0.34550400	-2.21556800
H	-4.47423800	0.71893700	-2.48250700
H	-4.91065400	-0.90484800	-3.03458100
H	-5.07164700	-0.48783400	-1.32808400
N	-0.76665500	-0.32510300	-1.00670200
Li	2.98188200	0.53356500	-0.46254400
O	3.57200100	-0.96464200	0.60618800
O	2.95712700	2.03039600	0.81690600
C	4.58336900	-1.87676500	0.16416600
H	4.14956600	-2.87136800	-0.00466200
H	4.98522000	-1.47631300	-0.77154300
H	5.38629900	-1.93994200	0.91209100
C	2.96160900	-1.35849700	1.83814300
H	3.72198000	-1.44552800	2.62602700
H	2.24563700	-0.57879400	2.10735800
H	2.43880900	-2.31439000	1.71708300
C	4.16234300	2.23974200	1.56672400
H	3.91569200	2.63096800	2.56404300
H	4.65969200	1.27179100	1.65360300
H	4.82183800	2.93913900	1.03674300
C	2.23787000	3.24929100	0.61783700
H	1.32685400	3.00561500	0.06591700
H	1.97120400	3.69229200	1.58736800
H	2.84182300	3.95950200	0.03605900

IM6

C	1.14933500	1.93981200	3.46134600
C	-0.15103900	1.65274300	3.09936900
C	-0.48762600	0.76341500	2.00427900
C	0.66906600	0.20675000	1.32447100
C	1.97606900	0.50043600	1.71547900
C	2.25484300	1.38348500	2.77301600
H	1.32612300	2.60773900	4.30385100
H	-0.97459000	2.08768800	3.66426000
H	2.80338300	-0.02161700	1.23263900
H	3.27686200	1.57169900	3.08664200
C	-2.07947700	-0.43803600	0.43003300
H	-2.19945400	-1.50925400	0.69723200
C	-0.86253900	-0.36886400	-0.52732600
N	0.40214600	-0.72121800	0.21960400
C	0.89288900	-4.35968000	0.20601700
C	1.92269900	-3.88112500	1.24813300
H	0.23803500	-5.15377500	0.57635600
H	1.36733500	-4.69623300	-0.72531600
H	1.60746700	-4.10465900	2.27471300
H	2.92498500	-4.29049500	1.08852000
Si	-3.76961400	0.07615100	-0.32793200
C	-5.02613200	0.39036500	1.06079400
H	-6.02719000	0.54008800	0.63456000
H	-5.08408300	-0.46127500	1.75081300
H	-4.77896300	1.27889700	1.65232800
C	-3.61337400	1.66798500	-1.36022300
H	-4.60249100	2.02929600	-1.67110500
H	-3.14092800	2.46204300	-0.76823300
H	-3.01852600	1.52195400	-2.27158000
C	-4.53560200	-1.29701100	-1.40663700
H	-3.97919800	-1.49564900	-2.32966900
H	-4.59356400	-2.24151100	-0.84913400
H	-5.56064900	-1.02453800	-1.69234700
B	0.79368000	-2.08737800	0.41236600
O	0.10581600	-3.19124800	-0.07050200
O	1.95813400	-2.45685600	1.07532700

C	-1.01751100	-1.14438200	-1.84756000
H	-1.30226700	-2.18058800	-1.64738600
H	-1.85772500	-0.68574000	-2.38090900
H	-0.74030000	0.69236000	-0.79282900
C	0.19951800	-1.11716100	-2.78151200
H	1.05690700	-1.65520000	-2.36381100
H	-0.04418100	-1.58735000	-3.74234200
H	0.52272100	-0.08900700	-2.99775200
Li	1.69028700	0.87344400	-0.52566000
O	3.38327300	0.54543100	-1.46406100
O	1.13859800	2.68834100	-1.03513300
C	0.51179400	3.09030700	-2.25159400
H	-0.58120500	3.05065000	-2.15773700
H	0.81634300	4.11196200	-2.51732400
H	0.83629100	2.40010200	-3.03442500
C	0.79029600	3.54258500	0.07389900
H	-0.28596900	3.48295700	0.26992500
H	1.33809100	3.18205700	0.94589400
H	1.07940500	4.57598200	-0.15870000
C	4.18102500	1.60906300	-1.98621600
H	5.07914300	1.75104500	-1.37044400
H	4.47808500	1.39037300	-3.02096500
H	3.57073700	2.51254000	-1.95539000
C	4.08923200	-0.69740300	-1.41217400
H	3.43487100	-1.42600500	-0.93193900
H	4.34601700	-1.03026100	-2.42675900
H	5.00624900	-0.58907300	-0.81790700
C	-1.78071800	0.43782400	1.63243700
H	-2.60050000	0.81977700	2.23401500

TS6_7

C	-0.34879100	-0.39615800	4.02156900
C	-1.27696000	-0.89539600	3.11671600
C	-1.09807200	-0.74639800	1.70480000
C	0.11181300	-0.08680200	1.30121200
C	1.03113100	0.40890600	2.21344400
C	0.81004800	0.28133000	3.59639500
H	-0.52974800	-0.52090000	5.08840300
H	-2.16388000	-1.41184500	3.48101200
H	1.96237000	0.87920700	1.88785700
H	1.52344600	0.68084100	4.31161800
C	-2.10625600	-0.44268200	-0.61076100
H	-2.23371300	-1.12110200	-1.47314600
C	-0.84798500	0.43136700	-0.87021800
N	0.35003600	-0.17962900	-0.14252700
C	0.70542200	-3.53784900	-1.67162700
C	1.09126800	-3.78555800	-0.19599600
H	-0.09171600	-4.20239400	-2.01963400
H	1.56201800	-3.63124000	-2.35376300
H	0.29403200	-4.30424200	0.34999800
H	2.02697200	-4.34271900	-0.07861700
Si	-3.73545900	0.56814700	-0.48188300
C	-5.18160100	-0.64094500	-0.24503100
H	-6.13903200	-0.10324600	-0.22867500
H	-5.22802500	-1.37322200	-1.06237300
H	-5.09516900	-1.19864900	0.69462600
C	-3.67908900	1.77367700	0.98375100
H	-4.66146000	2.24061500	1.13731000
H	-3.39642200	1.25995600	1.90957700
H	-2.95530700	2.58357000	0.82288700
C	-4.13818700	1.58067400	-2.05460400
H	-3.42860300	2.40245400	-2.21824400
H	-4.13557500	0.95410900	-2.95664200
H	-5.13816600	2.02910900	-1.97275300
B	0.48800100	-1.62163600	-0.44576700
O	0.24032500	-2.18190500	-1.69766200
O	1.24672700	-2.47290100	0.35318200
C	-0.59212100	0.66409000	-2.36891200
H	-0.43631900	-0.29894100	-2.86375400
H	-1.51645400	1.08069400	-2.78573400
H	-0.96507300	1.41223500	-0.39087000
C	0.56238500	1.61408900	-2.71014700
H	1.54367100	1.17638500	-2.47683600
H	0.57772900	1.83947700	-3.78357200
H	0.48067200	2.56816100	-2.17325100
Li	2.06598300	0.84036800	-0.23783300
O	3.76542600	-0.09597200	-0.53805300
O	2.46518500	2.71993000	0.06975200
C	3.60636100	3.37710500	-0.48145700
H	3.30435800	4.07041500	-1.27803900
H	4.14072900	3.93207600	0.30102300
H	4.25850200	2.60283900	-0.89008900
C	1.54224700	3.62724500	0.68777600
H	1.14288700	4.32707300	-0.05785500
H	0.73641400	3.02840400	1.11450300
H	2.04488000	4.18627200	1.48716300
C	4.32318200	-0.72548200	0.62898300
H	3.77160400	-1.64297400	0.86196500
H	5.38591000	-0.94199700	0.46069100
H	4.22192000	-0.02014100	1.45569200
C	3.95486700	-0.88550900	-1.71611900
H	3.49083300	-0.35100000	-2.54794700
H	5.02729700	-1.01142000	-1.91507200
H	3.48085900	-1.86746000	-1.60204800
C	-1.91074300	-1.26201900	0.66878100
H	-2.74903700	-1.89529600	0.96189900

IM7

C	0.83890600	-0.10124600	3.82448700
C	1.46783100	0.71205600	2.86655100
C	1.06720500	0.63999100	1.53447600
C	0.01757600	-0.23462300	1.16668300
C	-0.59732400	-1.05256500	2.10933700
C	-0.17838900	-0.98192500	3.45058600
H	1.14960100	-0.04767500	4.86565000
H	2.25898000	1.39608300	3.16894100
H	-1.39701500	-1.73702100	1.82983000
H	-0.65260000	-1.61414100	4.19801200
C	2.07278300	0.36596500	-0.73847000
H	2.22001400	0.94455800	-1.66401400
C	0.88887800	-0.64508700	-1.00255500
N	-0.31431800	-0.09435800	-0.24250000
C	-0.90503600	3.18397500	-1.81329900
C	-1.15690600	3.54421200	-0.33866300
H	-0.41619400	3.99363200	-2.37252100
H	-1.84825500	2.93788100	-2.33352100
H	-0.38371900	4.23927500	0.03019900
H	-2.13802700	4.00703300	-0.15996100
Si	3.77840700	-0.39635900	-0.35297300
C	4.96148300	0.97460500	0.21999000
H	5.97785400	0.58412900	0.36294500
H	5.01861700	1.78670000	-0.51666800
H	4.63752900	1.41514500	1.17092600
C	3.72585200	-1.75998200	0.96838000
H	4.73828800	-2.14246600	1.15758400
H	3.31830900	-1.39739700	1.91850200
H	3.11042800	-2.61147600	0.65016300
C	4.50002400	-1.16580500	-1.93910700
H	3.89326400	-2.00704800	-2.29915800
H	4.56060400	-0.43122200	-2.75288600
H	5.51471000	-1.54757300	-1.76251200
B	-0.03972600	1.53297700	-0.38204700

O	-0.06296900	2.04855300	-1.75543500
O	-1.07357400	2.30103800	0.33401600
C	0.62457000	-0.83818000	-2.50473300
H	0.28028400	0.11348900	-2.92078700
H	1.59349500	-1.05341500	-2.97634400
H	1.11023000	-1.62975000	-0.56595600
C	-0.34835500	-1.96347600	-2.87234600
H	-1.35735100	-1.78277300	-2.48146500
H	-0.43624400	-2.05921700	-3.96202000
H	-0.00977200	-2.93362400	-2.48372100
Li	-2.22488900	-0.50962100	-0.32013300
O	-3.85030600	0.52208900	0.11025700
O	-2.98247700	-2.35365900	-0.29422000
C	-4.37917300	-2.64404800	-0.37270800
H	-4.60417300	-3.19355100	-1.29701300
H	-4.69264800	-3.24524500	0.49164200
H	-4.90506700	-1.68899700	-0.36406200
C	-2.18337300	-3.53938400	-0.23784500
H	-2.32681200	-4.14017100	-1.14518400
H	-1.14188200	-3.22697700	-0.16620500
H	-2.45358500	-4.13490500	0.64438000
C	-3.83441800	1.03953800	1.45386900
H	-3.11044700	1.85712800	1.52745600
H	-4.84351600	1.36960500	1.73364300
H	-3.52370200	0.22095900	2.10660400
C	-4.36004000	1.47178400	-0.82765900
H	-4.32520900	1.00645200	-1.81637800
H	-5.40090900	1.72274600	-0.58163500
H	-3.74908200	2.38046700	-0.82782200
C	1.47136600	1.38083100	0.30001300
H	2.10368200	2.25888700	0.46426600

TS7_8

C	0.72335900	-0.08595400	3.85969300
C	1.39926600	0.67889800	2.89400500
C	1.04058600	0.57858700	1.55411200
C	-0.00935700	-0.28369100	1.14670800
C	-0.67286200	-1.04883100	2.11780700
C	-0.30756100	-0.94368900	3.46995600
H	1.00231100	-0.00920100	4.90806900
H	2.19006000	1.36322000	3.19895100
H	-1.46478400	-1.74054700	1.82969500
H	-0.82512500	-1.54419600	4.21583200
C	2.07158700	0.34570400	-0.71799400
H	2.26701100	0.94607200	-1.62111000
C	0.86074000	-0.62194700	-1.03420200
N	-0.33166000	-0.21936000	-0.23348700
C	-0.93519000	3.23265700	-1.81468500
C	-1.30617000	3.67312700	-0.39034100
H	-0.68230000	4.06680200	-2.47844200
H	-1.74216300	2.64621200	-2.28410500
H	-0.81619500	4.62079700	-0.12158100
H	-2.38671900	3.79537400	-0.23874900
Si	3.73933800	-0.50367100	-0.34487000
C	5.01672600	0.80710500	0.16831200
H	6.01293400	0.36018000	0.28628200
H	5.09798700	1.60373000	-0.58295400
H	4.75187200	1.27944700	1.12254200
C	3.64726400	-1.83255200	1.00738700
H	4.63666400	-2.28511800	1.16105500
H	3.30953600	-1.42071800	1.96471800
H	2.95483400	-2.63973600	0.73684100
C	4.36712500	-1.34142300	-1.93557000
H	3.68824100	-2.13534200	-2.27339300
H	4.46851000	-0.62175600	-2.75860000
H	5.35207000	-1.79992200	-1.77409200

B	0.18988300	1.92903600	-0.29341300
O	0.20871400	2.40902900	-1.62216600
O	-0.80730500	2.62711400	0.44003400
C	0.55860900	-0.65091700	-2.54359100
H	0.20044500	0.34012200	-2.84328900
H	1.51041700	-0.80645600	-3.07029300
H	1.13399500	-1.65346600	-0.74133000
C	-0.42351100	-1.73348700	-3.00522100
H	-1.42360300	-1.60137000	-2.57367300
H	-0.53745600	-1.71784600	-4.09688200
H	-0.07386700	-2.73663600	-2.72567400
Li	-2.18866600	-0.62981200	-0.33841100
O	-3.76482000	0.47021600	0.12285900
O	-2.94814500	-2.45820300	-0.35314600
C	-4.32839000	-2.81338800	-0.31725000
H	-4.60420400	-3.37980500	-1.21760700
H	-4.54581500	-3.42003400	0.57264300
H	-4.89794900	-1.88401900	-0.27100800
C	-2.08198200	-3.59763500	-0.35083600
H	-2.24941600	-4.20590800	-1.24939000
H	-1.05917500	-3.22114800	-0.34265700
H	-2.26094000	-4.20658300	0.54528000
C	-3.69176300	1.09838300	1.41590600
H	-3.08272200	2.00625400	1.36318700
H	-4.70477200	1.32181700	1.77559200
H	-3.20547400	0.38988500	2.08823600
C	-4.48937100	1.24751400	-0.82713000
H	-4.48801300	0.69733000	-1.77183700
H	-5.52595000	1.39061300	-0.49193100
H	-4.01786400	2.22716700	-0.97482600
C	1.53260300	1.34211300	0.36333400
H	2.26729600	2.11725800	0.61632700

IM8

C	-0.01648100	0.04058000	3.79157900
C	0.92264000	0.47176100	2.83885900
C	0.81250500	0.16708500	1.48595000
C	-0.28454900	-0.62851700	1.01047600
C	-1.19907600	-1.10265300	2.00803200
C	-1.07422300	-0.76250500	3.35616200
H	0.10031900	0.29689000	4.84100400
H	1.77060300	1.07551700	3.16648500
H	-1.96597000	-1.81964600	1.70859700
H	-1.79111100	-1.15832300	4.07435600
C	1.91347000	-0.39491200	-0.70204800
H	2.52517900	0.04731800	-1.50372400
C	0.50037900	-0.68334600	-1.29615600
N	-0.53905900	-0.92085500	-0.29683400
C	2.13554300	4.04418200	-1.18423900
C	0.90864300	4.27802700	-0.27445400
H	2.93478000	4.77433400	-1.02414700
H	1.87169600	4.03723400	-2.24892400
H	1.13136300	4.94437400	0.56754700
H	0.04212600	4.67231500	-0.81524600
Si	2.87233300	-2.00465400	-0.26830500
C	4.39588400	-1.58783500	0.78811200
H	5.02723400	-2.47726900	0.91617400
H	5.01474300	-0.80864900	0.32388400
H	4.11451500	-1.23990400	1.78976700
C	1.83817700	-3.31454800	0.62691800
H	2.41849000	-4.23908900	0.75204200
H	1.52295200	-2.97255200	1.61893700
H	0.92873600	-3.55566300	0.06393500
C	3.48586900	-2.74953100	-1.90947000
H	2.65403900	-3.00111600	-2.58007200
H	4.14416000	-2.05351500	-2.44620700

H	4.05441000	-3.67225500	-1.73236400
B	1.64134500	2.12281900	-0.04184500
O	2.60608900	2.73915500	-0.81751400
O	0.59024900	2.97497600	0.24599700
C	0.07465600	0.38794900	-2.33710900
H	-0.16269800	1.32717100	-1.81848300
H	0.92772700	0.59318000	-3.00106400
H	0.59018200	-1.61417300	-1.89091800
C	-1.11891500	-0.03169200	-3.20470000
H	-2.01276000	-0.23358000	-2.60260400
H	-1.37668200	0.74589900	-3.93553900
H	-0.89006200	-0.94896500	-3.76373800
Li	-2.37214000	-0.45365200	-0.06872200
O	-3.22435900	1.30988100	0.21867700
O	-3.76474700	-1.70632700	-0.62050900
C	-5.17056500	-1.64171800	-0.40987400
H	-5.71113600	-1.78840400	-1.35567800
H	-5.48937300	-2.40762900	0.31084700
H	-5.39067400	-0.65015300	-0.00865900
C	-3.31787800	-2.96988700	-1.12318900
H	-3.74604600	-3.15594300	-2.11762400
H	-2.22946000	-2.90926100	-1.17820900
H	-3.61399900	-3.77686200	-0.43919900
C	-2.74530600	2.10154800	1.32076600
H	-1.85105400	2.66153400	1.02552600
H	-3.53780400	2.78629900	1.65240300
H	-2.48480300	1.40898300	2.12184900
C	-3.61513600	2.10910400	-0.89345000
H	-3.96421400	1.43353100	-1.67825600
H	-4.42990800	2.78976800	-0.60877200
H	-2.76583400	2.69582200	-1.26911700
C	1.82733500	0.63946500	0.46844300
H	2.81508600	0.67398000	0.95783900

[3]IM9*

C	0.05935400	3.29507200	2.77691000
C	-1.11139800	2.65454300	2.39504800
C	-1.08552000	1.46740200	1.60412100
C	0.19240400	0.96781700	1.19732400
C	1.35769600	1.62237800	1.58987600
C	1.30129800	2.78087000	2.37757800
H	0.01335200	4.19316200	3.38737600
H	-2.07711300	3.04372600	2.70962200
H	2.33057900	1.21262700	1.33002100
H	2.22398200	3.27075800	2.67563700
C	-2.28070800	-0.37319300	0.30051200
H	-2.34219600	-1.33752800	0.84128600
C	-0.96045200	-0.35864000	-0.50875700
N	0.23328800	-0.25211400	0.41399000
C	1.08507500	-3.59291400	1.70207400
C	1.86442400	-2.65917800	2.65333200
H	0.44197900	-4.30550400	2.22621700
H	1.74447700	-4.13683700	1.01522700
H	1.44865700	-2.64616100	3.66694500
H	2.93171400	-2.89642200	2.70492300
Si	-3.92165800	-0.32363400	-0.74805600
C	-5.34544400	0.19441800	0.39355000
H	-6.30028200	0.06446800	-0.13221000
H	-5.39012400	-0.41865300	1.30317000
H	-5.28295700	1.24555100	0.69871300
C	-3.77118900	0.93184900	-2.15895500
H	-4.72518100	1.01534000	-2.69600700
H	-3.52139500	1.93079700	-1.77887800
H	-3.00735700	0.65485200	-2.89630800
C	-4.35267700	-2.04638500	-1.41298200
H	-3.65097100	-2.41520500	-2.16845800
H	-4.38512100	-2.78567900	-0.60191900
H	-5.34914300	-2.02495200	-1.87355100
B	0.71054300	-1.42446900	1.13357100
O	0.25434900	-2.70333600	0.92366300
O	1.70548200	-1.34418400	2.08084200
C	-0.84497600	-1.49709400	-1.53466800
H	-1.03207900	-2.46191700	-1.05647000
H	-1.65021900	-1.33933400	-2.26031100
H	-0.94373500	0.58336800	-1.07475300
C	0.47773400	-1.55211500	-2.30476600
H	1.31244700	-1.87020300	-1.66863100
H	0.41987600	-2.28278500	-3.11990000
H	0.71799300	-0.58502100	-2.77512500
Li	1.96684100	0.33721800	-0.89249500
O	3.63936300	-0.61504900	-0.96065700
O	1.99406300	1.99724200	-1.85343100
C	3.13514300	2.40123300	-2.62686100
H	2.85609800	2.50628000	-3.68412000
H	3.52756700	3.35316300	-2.24451900
H	3.89717500	1.62697700	-2.52036400
C	0.95877200	2.98852600	-1.87511200
H	0.58102400	3.11632300	-2.89797500
H	0.15691200	2.64506900	-1.21969100
H	1.34748300	3.94355600	-1.49946600
C	4.56792700	-0.70313000	0.13223100
H	4.79748600	-1.75704400	0.34268600
H	5.49110100	-0.16221800	-0.11575600
H	4.09366200	-0.25424600	1.00757800
C	4.14597900	-1.25316000	-2.14475100
H	3.42235700	-1.08994300	-2.94849800
H	5.11188900	-0.81231400	-2.42614000
H	4.26580500	-2.33099200	-1.96696800
C	-2.26335300	0.76047700	1.27928800
H	-3.18537800	1.06325500	1.76782300

[3]TS9_10*

C	-0.48979900	-1.79220300	3.43683100
C	-1.27999700	-1.98972400	2.33974000
C	-1.14238800	-1.15837200	1.18719100
C	0.00015900	-0.15044300	1.14789800
C	0.79831800	0.02448200	2.26351300
C	0.54171000	-0.70575900	3.45095200
H	-0.57912600	-2.43621200	4.30788700
H	-2.00780800	-2.80067400	2.32963800
H	1.59425900	0.77009100	2.26353900
H	1.03113500	-0.44414900	4.38390400
C	-2.17194600	-0.06264700	-0.79667500
H	-2.34136300	-0.23648100	-1.87111100
C	-1.01121300	1.00705900	-0.66667800
N	0.22990900	0.32533600	-0.15465400
C	1.10172100	-1.89067300	-2.87096300
C	1.47567900	-2.88082600	-1.75894900
H	0.93404800	-2.36696000	-3.84403700
H	1.87720700	-1.11342700	-2.99469600
H	1.01259400	-3.86487900	-1.93275100
H	2.55871100	-3.02857100	-1.65415100
Si	-3.86457800	0.45648300	-0.08681700
C	-5.06904800	-1.00724100	-0.20566200
H	-6.07790500	-0.71435800	0.11421500
H	-5.14361900	-1.38260900	-1.23475700
H	-4.75082100	-1.84389800	0.42865500
C	-3.78223200	1.04474400	1.71578400
H	-4.77252300	1.38559300	2.04858500
H	-3.45376000	0.24707300	2.39131500
H	-3.08773100	1.88531700	1.84068500
C	-4.56640100	1.88507600	-1.13250400

H	-3.93347600	2.78056200	-1.07901600
H	-4.65544100	1.60651200	-2.19074600
H	-5.56693400	2.17119700	-0.78105700
B	-0.12502600	-1.41221800	-0.97872400
O	-0.10756100	-1.31157400	-2.40729000
O	0.94252800	-2.29311200	-0.57994000
C	-0.76939000	1.73152600	-2.00077600
H	-0.39808400	1.00201800	-2.72784200
H	-1.74986700	2.06459000	-2.36911000
H	-1.28410300	1.77188400	0.07853300
C	0.16273700	2.94632300	-1.94007800
H	1.17901300	2.66927400	-1.63610900
H	0.23433000	3.43024900	-2.92257200
H	-0.20411400	3.69918200	-1.22899000
Li	2.14655000	0.58984100	-0.07572400
O	3.76167000	-0.53260300	-0.17118300
O	2.88863300	2.33290600	0.58227300
C	4.26434500	2.68110400	0.71455200
H	4.48928500	3.58889900	0.13772100
H	4.51885600	2.85056700	1.76991600
H	4.84929900	1.84399300	0.33167700
C	2.01803300	3.33989600	1.11067700
H	2.13838900	4.27767800	0.55283500
H	0.99852600	2.97140000	1.00186500
H	2.23946200	3.51086100	2.17251900
C	3.88422300	-1.58508600	0.79846300
H	3.78309000	-2.56170000	0.31273800
H	4.85811100	-1.51378900	1.30170200
H	3.07275700	-1.46173100	1.51645800
C	4.71764000	-0.63233400	-1.22185500
H	4.54273000	0.19977700	-1.90850100
H	5.73999300	-0.56861700	-0.82245500
H	4.60120300	-1.57993400	-1.76382300
C	-1.55699000	-1.38654700	-0.22445900
H	-2.19922200	-2.26033700	-0.37676400

^{3}IM10*

C	0.08024400	-0.29197300	3.74313600
C	-0.95089000	-0.61624200	2.83666700
C	-0.83717700	-0.18341200	1.46949200
C	0.29733400	0.54648500	1.05763200
C	1.38486800	0.85196800	2.00722000
C	1.21550200	0.43121300	3.36893500
H	-0.02073900	-0.60425400	4.78285000
H	-1.83715700	-1.15161400	3.16626900
H	1.97471000	1.75376400	1.79808900
H	1.95760900	0.69901800	4.11786400
C	-1.91268600	0.49333200	-0.69574800
H	-2.50166000	0.08734500	-1.53457500
C	-0.48719700	0.79877100	-1.24276200
N	0.55696000	0.90800500	-0.22470100
C	-2.47108600	-4.01303800	-1.04236500
C	-1.00652700	-4.15694700	-0.57148100
H	-3.12858100	-4.79211200	-0.64499300
H	-2.56081400	-3.99866200	-2.13539200
H	-0.90752100	-4.83126500	0.28762700
H	-0.33177000	-4.49382000	-1.36518800
Si	-2.87760800	2.09022200	-0.22088400
C	-4.54262300	1.62003600	0.55869100
H	-5.17521500	2.51068800	0.67154500
H	-5.09371600	0.89871800	-0.05870800
H	-4.41779600	1.17922400	1.55514200
C	-1.93379800	3.23198400	0.95762500
H	-2.50124400	4.15670000	1.13074900
H	-1.76102400	2.75158500	1.92733500
H	-0.95260500	3.51188800	0.55460100

C	-3.23294200	3.03823200	-1.83386000
H	-2.31370300	3.34995700	-2.34656400
H	-3.81557100	2.43060700	-2.53897600
H	-3.81275500	3.94744600	-1.62660800
B	-1.77099200	-2.05928400	-0.07393500
O	-2.88616200	-2.73749300	-0.52870900
O	-0.62705100	-2.83225500	-0.15719600
C	-0.03477600	-0.17654700	-2.36449800
H	0.19371400	-1.15396800	-1.92335600
H	-0.88672300	-0.32453200	-3.04275200
H	-0.51378800	1.78921700	-1.73043600
C	1.15872500	0.32501000	-3.18693700
H	2.04907900	0.48149400	-2.56804400
H	1.42144400	-0.39102500	-3.97600800
H	0.92565300	1.28139600	-3.67368000
Li	2.48160000	0.42186400	0.09350700
O	3.20555000	-1.41402200	0.02692000
O	3.89889800	1.62377600	-0.52026000
C	5.27876900	1.45119700	-0.20701700
H	5.90297000	1.68513700	-1.08061900
H	5.56875300	2.09941000	0.63132200
H	5.41461300	0.40552200	0.07555500
C	3.56736900	2.96343400	-0.89183600
H	4.10369600	3.24834000	-1.80740700
H	2.49029000	2.98507800	-1.06408700
H	3.82504200	3.66065600	-0.08322100
C	2.76985200	-2.27479800	1.09565700
H	1.85384600	-2.80123600	0.80465400
H	3.56626600	-2.99202800	1.33737100
H	2.55955700	-1.63321300	1.95246700
C	3.50980900	-2.13211100	-1.16450700
H	3.83229600	-1.40467700	-1.91339400
H	4.31985300	-2.85255000	-0.98235300
H	2.62431100	-2.66800200	-1.53234100
C	-1.87409300	-0.56933100	0.45097800
H	-2.85531000	-0.55216700	0.95353000

IM11

C	0.69001200	-3.53405500	-1.79242300
C	1.31214900	-2.30692000	-1.58262200
C	0.71679000	-1.28124700	-0.83258400
C	-0.56552900	-1.50343400	-0.28086300
C	-1.18404600	-2.75461900	-0.47903300
C	-0.56894900	-3.75363400	-1.22725800
H	1.18060700	-4.30598800	-2.37955200
H	2.29792900	-2.12865700	-2.00768900
H	-2.16158500	-2.93540300	-0.05128100
H	-1.07672800	-4.70545800	-1.36390400
C	0.55411600	1.16204000	-0.09470900
H	1.19396000	1.92285400	0.37444900
C	-0.36736200	0.60152700	1.01036900
C	-4.83742700	-0.91776100	0.87307500
C	-4.58301300	0.48136200	1.47130600
H	-5.63166800	-0.92865100	0.12076600
H	-5.06921900	-1.66265000	1.64486000
H	-4.96164800	1.28268100	0.82400300
H	-5.00615200	0.60646200	2.47260900
Si	-0.35103300	2.15279900	-1.47297100
C	-1.57786200	3.36736600	-0.68339200
H	-2.03431700	4.00448100	-1.45244700
H	-2.38862300	2.85387200	-0.15285400
H	-1.07990600	4.02988300	0.03696800
C	0.97163500	3.16058200	-2.39245000
H	0.51173100	3.78068000	-3.17320800
H	1.51260800	3.83115700	-1.71242500
H	1.71573900	2.52018500	-2.88297000

C	-1.25171900	1.06196000	-2.73321400
H	-0.57520900	0.33629400	-3.20053900
H	-2.07230100	0.49451000	-2.28017500
H	-1.67417600	1.68389600	-3.53407400
B	-2.61581700	-0.41699000	0.73743200
O	-3.59392400	-1.27168400	0.24952000
O	-3.15433100	0.58145600	1.54620200
C	0.35401400	0.11487500	2.29033400
H	-0.40547700	-0.36268700	2.92312700
H	1.07846800	-0.67296800	2.04597800
H	-1.04684700	1.39657100	1.32562800
C	1.03129600	1.23643300	3.08877900
H	0.31079000	2.02316000	3.34853700
H	1.44426100	0.84655700	4.02728100
H	1.85263800	1.70367900	2.53468200
N	-1.22198100	-0.48137200	0.46280800
C	4.82773400	0.55856500	1.04351100
C	4.64795700	-0.96789300	1.22540800
H	5.63053200	0.80751400	0.34000400
H	5.00869000	1.08533700	1.98530000
H	5.47569700	-1.55112100	0.81125000
H	4.50467700	-1.25350400	2.27365300
B	2.83396100	-0.10439800	0.14014800
O	3.57794500	1.00531100	0.48760700
O	3.44547700	-1.28146500	0.49975300
C	1.46896200	0.02971100	-0.65552200
H	1.80925200	0.33658300	-1.66087600

IM12

C	-0.14373200	2.75862100	-3.33057700
C	0.98888900	2.30691100	-2.64408900
C	0.93490900	1.22472600	-1.72655200
C	-0.34664200	0.57669100	-1.53734700
C	-1.45884600	1.03063100	-2.26651400
C	-1.38031400	2.12558600	-3.14710700
H	2.96184400	1.24570500	-1.07593000
H	-0.05412700	3.58545600	-4.03411200
H	1.95088500	2.78952300	-2.82178800
C	1.99968100	0.76431900	-0.87310600
H	-2.40686100	0.49397200	-2.16910200
H	-2.25742900	2.44415500	-3.70690100
C	2.10565900	-0.76980000	-0.75061300
H	2.54595200	-1.17710700	-1.68858200
C	0.67062700	-1.41265200	-0.62227700
Si	3.37532500	-1.22984200	0.59388200
C	3.86565300	-3.07417900	0.55948000
H	4.62038800	-3.27930000	1.33131100
H	3.01315400	-3.73848200	0.74879300
H	4.29833300	-3.36368200	-0.40675900
C	4.97926800	-0.24226800	0.33332500
H	5.75280300	-0.55366600	1.04838600
H	5.38075700	-0.39498200	-0.67724000
H	4.81332700	0.83371500	0.46214000
C	2.75756800	-0.88752800	2.36326700
H	2.40868300	0.14565500	2.47420600
H	1.93249500	-1.55714300	2.63839400
H	3.56716100	-1.05211200	3.08736500
C	0.39121600	-2.40030700	-1.78923700
H	-0.66704700	-2.69647100	-1.72206600
H	0.48727600	-1.84611200	-2.73265200
H	0.60712400	-2.00404500	0.31087400
C	1.25384200	-3.66683600	-1.84553200
H	1.18517600	-4.24466100	-0.91283500
H	0.93364500	-4.32343900	-2.66611400
H	2.31198900	-3.43508300	-2.00540200
N	-0.43024900	-0.41592100	-0.54023100
Li	0.42232500	1.29844700	0.53261600
O	0.40632900	3.43148300	0.75656100
O	-0.32833500	1.13567000	2.52902900
C	-0.68663000	4.15284000	0.18959300
H	-0.66212600	4.09862500	-0.90540400
H	-1.60302600	3.68606400	0.55888200
H	-0.66229000	5.20315300	0.51704700
C	1.66507800	4.03348400	0.44751500
H	1.71601000	5.04250000	0.88373000
H	2.43789900	3.39535000	0.87820200
H	1.80817000	4.08748500	-0.63774700
C	-0.15598300	2.09385500	3.56939700
H	0.62704500	1.76774300	4.26913700
H	0.13585900	3.02786300	3.08954900
H	-1.09589000	2.23429800	4.12466200
C	-0.73481600	-0.13247500	3.02403400
H	-0.73143500	-0.81303300	2.17061800
H	-0.03286900	-0.49958600	3.78503800
H	-1.74326600	-0.07240500	3.46203000
Li	-2.17285700	-0.95558800	-0.03382700
O	-2.90462200	-2.82287700	0.08638600
O	-3.72940000	0.06811500	0.62615100
C	-3.78909900	1.49222800	0.48509200
H	-3.64183500	1.97514300	1.46036600
H	-2.99379400	1.77989200	-0.20391200
H	-4.76192700	1.78924900	0.07075300
C	-2.15481600	-3.88605400	0.68016500
H	-2.83407500	-4.66237000	1.05764700
H	-1.45841500	-4.32220800	-0.04590700
H	-1.58894900	-3.45963400	1.51085800
C	-3.68635500	-3.26086600	-1.02789400
H	-4.40363700	-4.03007100	-0.71093500
H	-4.22824400	-2.39085000	-1.40582000
H	-3.04069500	-3.66194000	-1.81948500
C	-4.76009000	-0.44349800	1.46757500
H	-5.74878900	-0.20687700	1.05050700
H	-4.62907600	-1.52562500	1.51621600
H	-4.68356200	-0.01369800	2.47628600
